# Neutral anion-detecting organic cages based on anion–π interactions

**DOI:** 10.1039/d5sc08157b

**Published:** 2026-01-23

**Authors:** Yuyang Lu, Ping Zhou, Hua Tang, Yating Wu, Yueyan Kuang, Ze Cao, Jiyong Liu, Guangcheng Wu, Hongliang Chen, Hao Li

**Affiliations:** a Stoddart Institute of Molecular, Science Department of Chemistry, Zhejiang University Hangzhou 310058 China lihao2015@zju.edu.cn hongliang.chen@zju.edu.cn; b Zhejiang-Israel Joint Laboratory of Self-Assembling Functional Materials, ZJU-Hangzhou Global Scientific and Technological Innovation Center, Zhejiang University Hangzhou 311215 China; c Beijing National Laboratory for Molecular Sciences Beijing 100871 China; d Department of Chemistry, Zhejiang University Hangzhou 310058 China; e Department of Chemistry, The University of Hong Kong Hong Kong SAR 999077 China gcwu2023@hku.hk

## Abstract

A series of neutral tetrahedral molecular cages were self-assembled in relatively high yields by condensing a triamino linker with triangular tris-aldehyde precursors. Each tris-aldehyde features a central triazine core, which imparts an electron-deficient cavity that facilitates anion encapsulation through fourfold anion–π interactions. The anion binding affinity is significantly influenced by substituents on the tris-aldehyde precursors: electron-donating groups (*e.g.*, Ph) diminish binding by compromising the electron-deficient nature of the cage, whereas more electron-withdrawing substituents (*e.g.*, Cl, Br, and CF_3_-Ph) enhance it. Interestingly, the strongly electron-withdrawing fluorine (F) substituents, in close proximity to the binding pocket, unexpectedly diminish binding affinity due to a repulsive field effect. Within each corner of the tetrahedral framework, intramolecular CH–π interactions occur between a phenyl proton *ortho* to the imine bond and an adjacent phenyl plane. The encapsulation of anionic guests within the cavity perturbed or reinforced these CH–π interactions to varying degrees, producing distinct NMR responses that serve as signatures for different anions.

## Abstract 2

A series of tetrahedral molecular cages were synthesized *via* imine condensation reactions. Each face features a central triazine core, which imparts an electron-deficient cavity that facilitates anion encapsulation through anion–π interactions.

## Graphical abstract

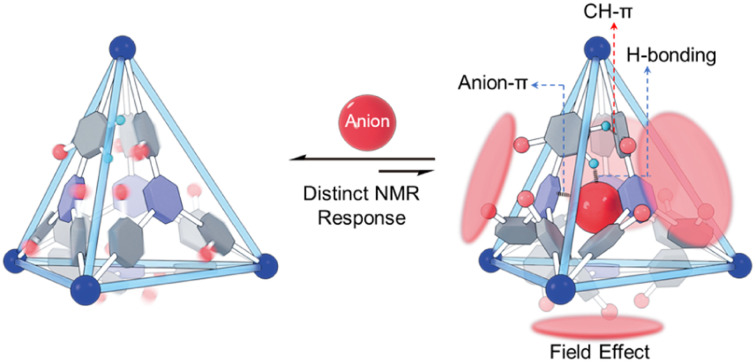

## Introduction

**Fig. 1:**
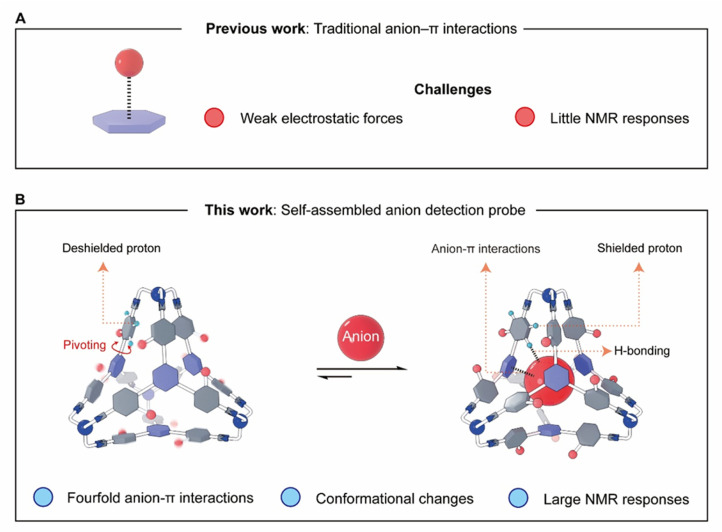
Graphical representations of (A) a traditional electron-deficient π system engaging in an anion–π interaction, and (B) a self-assembled cage encapsulating an anion *via* fourfold anion–π interactions in CDCl_3_. The preorganized cage architecture in (B) enhances binding affinity relative to the open system in (A), *via* a combination of many supramolecular forces including anion–π interactions and hydrogen bonds. Anion encapsulation induces conformational changes in the cage, resulting in significant NMR chemical shift changes for protons that experience altered chemical environments.

The recognition and detection of anions represent a major focus in supramolecular chemistry due to their critical roles in nature, such as metabolic regulation,^1,2^ aquatic eutrophication,^3,4^ and the maintenance of physiological homeostasis.^5,6^ A common approach of anion detection involves designing artificial anion acceptors that can bind with anionic targets by leveraging hydrogen bonding,^7–16^ halogen bonding,^17–26^ electrostatic forces,^27^ hydrophobic effects,^28–32^ and coordination to metal ions^33–36^ or boron atoms^37^ developed more recently. However, another type of weak supramolecular force—anion–π interaction^38–41^—was long overlooked in host–guest chemistry. This changed a few decades ago when some theoretical chemists proposed^42–44^ the feasibility of using electron-deficient π systems to bind negatively charged species (Fig. 1A). Typically, the so-called anion–π interactions are weaker in strength than commonly employed interactions including hydrogen bonds.^45,46^ As a consequence, anion–π interactions can hardly be used as the primary binding forces that solely drive guest recognition in the absence of other noncovalent forces. For example, many groups obtained^47–50^ various coordination cages bearing triazine walls, whose cavities can encapsulate anions. Here, electrostatic forces resulting from metal cations and anion–π interactions act as the primary and secondary driving forces, respectively, as inferred from the results that anion recognition can still occur even after the triazine units were replaced with electron rich phenyls.^51,52^ Developing neutral anion-detecting probes, in which anion–π interactions play the predominant roles, still needs to be exploited. Additionally, unlike hydrogen bonds, which exhibit partial covalent character and induce noticeable shifts in proton NMR spectra, anion–π interactions arise from electrostatic forces and usually result in minimal or no shifts^53^ in the corresponding ^1^H NMR spectra, unless hydrogen bonding is also involved.^54^ Other techniques for probing anion–π interactions also face limitations, including (i) crystallography,^55^ which only confirms binding in the solid state; (ii) mass spectrometry,^56^ which provides limited quantitative information; and (iii) UV-Vis absorption and fluorescence^57^ spectroscopies, which require dramatically different spectra for hosts and the corresponding host–anion complexes, a condition not always met. Therefore, it remains a key challenge to develop artificial hosts with high synthetic efficiency and the ability to produce distinct NMR responses for different anions as their signatures.

In our group, a variety of complex molecules^58^ were synthesized through reversible organic reactions, including imine bond formation, which allows for error correction. For example, a series of tetrahedral molecules^59–63^ were self-assembled by condensing four equivalents of the trisamino linker tris(2-aminoethyl)amine (TREN) as the vertices with four equivalents of various trisformyl precursors as the faces. In some cases, the yields of these tetrahedral cages are nearly quantitative, partially due to the stabilizing intramolecular CH–π interactions at each tetrahedral corner between an *ortho*-phenyl proton (relative to an imine bond) and the adjacent phenyl moiety. The critical role that CH–π interactions play in driving the formation of tetrahedra was confirmed by a control experiment in which, when one of the *ortho*-phenyl protons was replaced by other nonacidic unit, including F, the tetrahedral cages cannot form.^61^ None of these tetrahedral cages exhibit the ability to accommodate anions, due to the electron rich nature of the phenyl building blocks. We propose that replacing the phenyl units on the faces with electron-deficient triazine moieties could generate a tetrahedron whose cavity is capable of anion recognition *via* fourfold anion–π interactions (Fig. 1B) in the absence of electrostatic attraction.

## Results and discussion

**Fig. 2:**
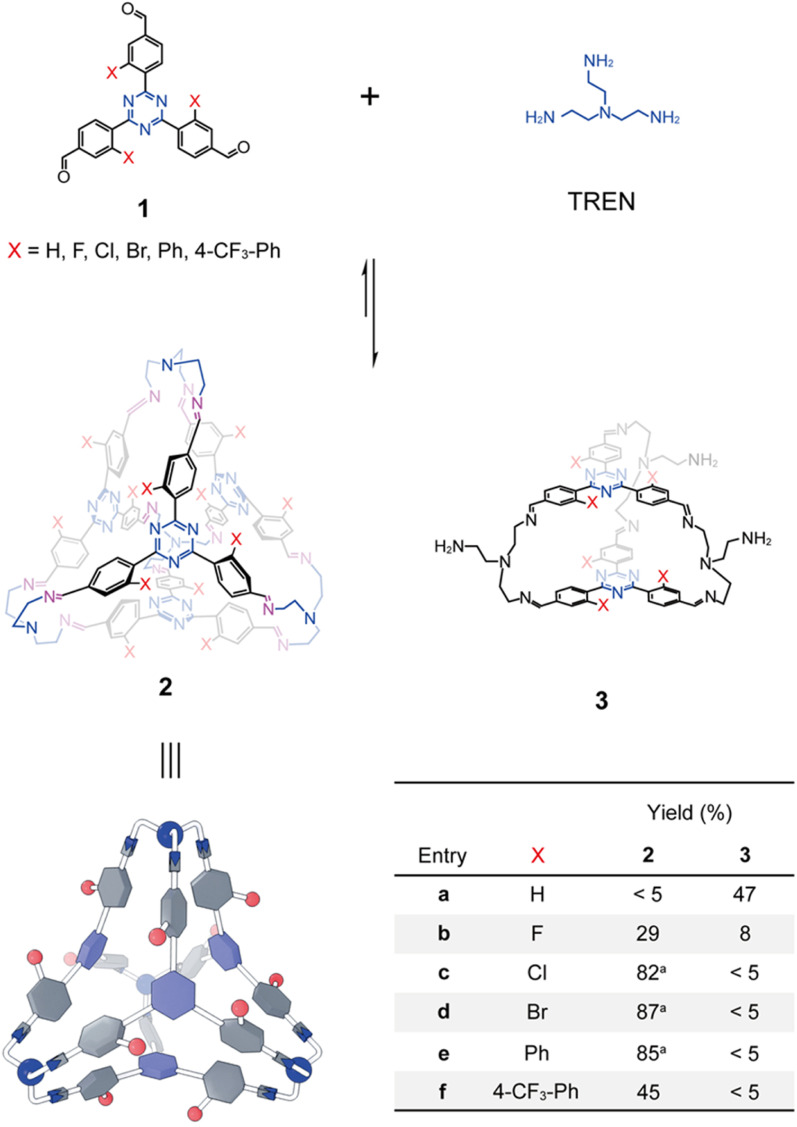
Structural formulae of tetrahedral cages (2a–2f) and prisms (3a–3f) obtained from the condensation of triformyl precursors (1a–1f) with the triamino linker, TREN. Yields are provided in the table at the bottom right. ^*a*^Yields for 2c, 2d, and 2e are isolated; all others were determined by integration of product resonances in their *in situ*^1^H NMR spectra relative to an internal standard. A yield of <5% indicates the product was not observed in the corresponding ^1^H NMR spectrum.

A series of trisformyl precursors each with a central triazine core 1a–1f (Fig. 2) were obtained either through commercial purchase or synthesis. The commercially available 1a was first combined with TREN in CDCl_3_ in a 1 : 1 ratio. The solution was heated at 55 °C for 12 h to allow imine formation to reach equilibrium. However, the ^1^H NMR spectrum indicated that the expected tetrahedron 2a was not produced. Instead, a triangular prism-shaped molecule (3a) was self-assembled within a library of mixture, consistent with our previously reported results.^64^ The failure of tetrahedron formation stems from the fact that the tetrahedron formation requires the occurrence of CH–π interactions as the driving force, which necessitates that each benzaldehyde unit adopts an edge-in conformation. Given that the central triazine core orientates in a face-in manner within the tetrahedron framework, this in turn forces each benzaldehyde unit to twist into a specific dihedral angle relative to the central triazine unit. Unfortunately, 1a has a planar conformation, stabilized by intramolecular CH–N hydrogen bonds and phenyl-triazine conjugation. This conformational difference between the precursor 1a and the putative product 2a implies an energetic penalty that disfavors tetrahedron formation.

1b–1f are derivatives of 1a, each bearing a functional group at the *meta*-position relative to the formyl unit of the benzaldehyde function, including F (1b), Cl (1c), Br (1d), Ph (1e), and 4-CF_3_Ph (1f). These substituents introduce either steric hindrance or coulombic repulsion, thereby forcing each benzaldehyde unit to adopt a twisted dihedral angle relative to the triazine core. We thus proposed that the more twisted conformations of the trisformyl precursors 1b–1f relative to 1a might favor the formation of the corresponding tetrahedral products. The F-substituents in 1b, being the smallest non-hydrogen atom, endow this precursor with the smallest twisting dihedral angle. It is thus predictable that the formation of the tetrahedron 2b would be favored to the least extent. This hypothesis was confirmed, as a 1 : 1 mixture of 1b and TREN produced both tetrahedron 2b and prism 3b in a 2 : 1 manner, which was indicated by the ^1^H NMR spectrum (see Fig. S32 in the SI). By comparing the integrations of the corresponding resonances of the products and the formyl precursor relative to an internal standard, the cyclization yields of 2b and 3b were calculated to be around 29% and 8%, respectively.

As a comparison, combination of each of 1c–1f with TREN in CDCl_3_ produced only the corresponding tetrahedra 2c–2f as the sole observable products in their ^1^H NMR spectra (see the SI), further confirming that their preorganized twisted conformations are of importance in self-assembly, by favoring the occurrence of the CH–π interactions. 2c, 2d and 2e were isolated as solid-state compounds *via* precipitation by adding petroleum ether into the corresponding self-assembly solutions (see the detailed procedures in the SI). The isolated yields of 2c, 2d and 2e were determined to be 82%, 87% and 85%, respectively. These cages were re-dissolved in CDCl_3_ and their ^1^H NMR spectra were recorded, confirming their purity and indicating that 2c, 2d and 2e are rather stable or kinetically inert during precipitation, despite the dynamic nature of imine formation. Attempts to isolate 2f using the same procedure were unsuccessful. The ^1^H NMR spectrum recorded after precipitation showed a few resonances corresponding to impurities that were not detected in the *in situ* self-assembly solution, suggesting partial degradation of 2f during precipitation. Therefore, the *in situ* self-assembly solution of 2f was used for the following host–guest binding investigations. The cyclization yield of 2f was determined to be 45% by using an internal standard. The relatively low yield of 2f compared with other cage products was due to the production of some insoluble byproducts, which might be oligomers or polymers. Luckily, 2f is the only observable product in the ^1^H NMR spectrum, which can be used for host–guest recognition without further purification.

**Fig. 3:**
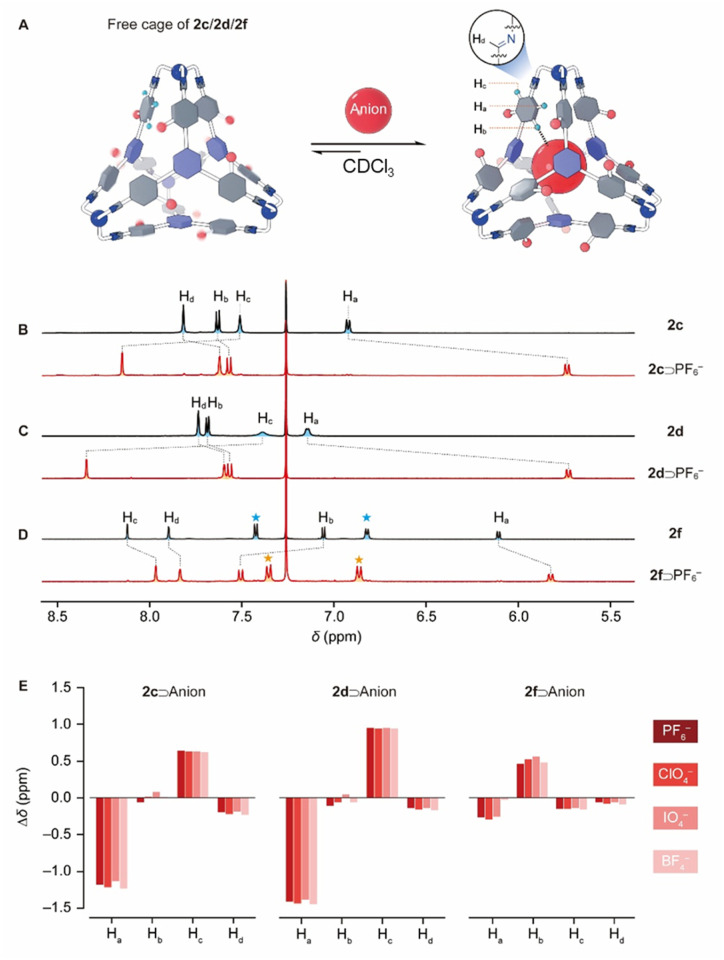
(A) Graphical representation of cages 2c, 2d, and 2f encapsulating an anionic guest. Partial ^1^H NMR spectra (400 MHz, CDCl_3_, 298 K) of (B) 2c, (C) 2d, and (D) 2f before (top, black trace) and after (bottom, red trace) encapsulation of a PF_6_^−^ guest. In (D), resonances marked with blue and orange asterisks correspond to protons on the 4-CF_3_Ph substituents of 2f. (E) Chemical shift changes (Δ*δ*, ppm) for protons H_a_–H_d_ in the host–guest complexes of anion⊂2c (left), anion⊂2d (middle), and anion⊂2f (right) relative to the corresponding “free” cages namely 2c, 2d and 2f, respectively. Proton assignments are provided in (A).

In the ^1^H NMR spectrum of 2c (Fig. 3B), the resonances corresponding to the protons a and c in the *ortho* positions relative to the imine bond were observed at around 6.93 and 7.82 ppm, which shifted upfield by 1.04 and 0.59 ppm, respectively, compared to the precursor 1c. These substantial upfield shifts for both protons confirm that each experiences a shielded magnetic environment due to intramolecular CH–π interactions with an adjacent phenyl moiety within the cage framework. The ^1^H NMR spectra of 2b and 2d exhibited similar patterns, except that in 2d, the resonances corresponding to protons a and c became relatively broad (Fig. 3C), indicating that rotation within 2d occurs at a slower rate compared to 2c. This is attributed to the bulkier Br atom in 2d, which introduces a higher energy barrier for rotation. In contrast, no rotation was observed in the framework of either 2e or 2f. In the ^1^H NMR spectrum of 2e (Fig. S47), the resonances corresponding to a and c appeared at 6.10 and 8.10 ppm, respectively, which are located in significantly upfield and downfield positions compared with their counterparts in 2c or 2d. This observation indicated that in 2e, proton a is oriented inward toward the cage cavity while proton c is oriented outward, away from the cavity at all times – a conformation driven by the large phenyl substituent, which cannot fit within the cage cavity. The ^1^H NMR spectrum of 2f (Fig. 3D) is similar to that of 2e, which is predictable given that its 4-CF_3_Ph substituent is even bulkier.

Given that each tetrahedral cage contains four triazine units, we hypothesized that they are capable of recognizing anionic guests *via* fourfold anion–π interactions. Various commercially available anions, all introduced as tetrabutylammonium (TBA^+^) salts, were added to CDCl_3_ solutions of 2b. Little or no changes were observed in the ^1^H NMR spectra (Fig. S69), indicating that 2b is incapable of recognizing any of these anions. In the case of 2c and 2d, addition of F^−^, Cl^−^, Br^−^, I^−^, H_2_PO_4_^−^, NO_3_^−^, AcO^−^ and SiF_6_^2−^, led to little or no changes in the corresponding ^1^H NMR spectra (Fig. S57 and S62) either. These observations indicated that the cage either 2c or 2d has little or no binding towards these anions, probably because of these less complementary sizes or weaker anion–π interactions. Addition of HSO_4_^−^ led to degradation of 2c. To our delight, addition of PF_6_^−^ to 2d led to a concomitant attenuation of the resonances corresponding to the “free” cage and an increase of a new set of resonances corresponding to the complex PF_6_^−^⊂2d. After approximately six hours, no further change in the composition of the reaction mixture was observed, indicating that complexation of PF_6_^−^⊂2d had reached equilibrium. In the case of the complex PF_6_^−^⊂2d, the resonances corresponding to protons a and c were observed at 5.74 and 8.34 ppm, which shifted upfield and downfield by 1.41 and 0.95 ppm compared with those of the “free” cage 2d, respectively (Fig. 3C, red trace). Apparently, these pronounced resonance shifts resulted from the conformational rearrangements upon guest encapsulation: the anionic guest forced the Br atoms and proton c to orient outward, while forcing proton a to reside inward at all times. The binding constant (*K*_a_) for PF_6_^−^⊂2d was evaluated to be (5.6 ± 0.5) ×10^3^ M^−1^ (Table 1) by integrating the resonances corresponding to the “free” cage and the complex in the ^1^H NMR spectrum of a mixture of PF_6_^−^ and 2d. By performing similar experiments, a series of anionic complexes including ClO_4_^−^⊂2d, IO_4_^−^⊂2d and BF_4_^−^⊂2d were obtained. In their corresponding ^1^H NMR spectra (Fig. S63–S66), the resonances corresponding to the proton a were observed to undergo upfield shifts by 1.44, 1.38 and 1.45 ppm, respectively (Fig. 3E, middle). The binding constants of ClO_4_^−^⊂2d, IO_4_^−^⊂2d and BF_4_^−^⊂2d were determined to be (1.0 ± 0.1) ×10^3^, (2.5 ± 0.1) ×10^2^ and (1.6 ± 0.1) ×10^2^ M^−1^, respectively (Table 1). All the above anionic guests were considered having little or no association with their counterion TBA^+^ in chlorinated solvents due to their lower basicity.^65–67^

**Table 1:** Association constants *K*_a_ for each of the hosts 2b–2f to recognize the anions. N. D. = not determined *via*^1^H NMR spectroscopy, because the corresponding *K*_a_ values are too small to be measured

*K*_a_ (M^−1^)	2b	2c	2d	2e	2f
PF_6_^−^	N. D.	8.2 (±0.3) × 10^2^	5.6 (±0.5) × 10^3^	N. D.	5.7 (±0.4) × 10^3^
ClO_4_^−^	N. D.	2.2 (±0.1) × 10^2^	1.0 (±0.1) × 10^3^	N. D.	1.1 (±0.3) × 10^3^
IO_4_^−^	N. D.	7.0 (±0.7) × 10^1^	2.5 (±0.1) × 10^2^	N. D.	1.2 (±0.2) × 10^3^
BF_4_^−^	N. D.	3.0 (±0.4) × 10^1^	1.6 (±0.1) × 10^2^	N. D.	9.0 (±0.7) × 10^1^

To investigate the influence of the solvophobic effect on binding constants, we also performed binding experiments in more polar solvents, namely CDCl_3_/CD_3_SOCD_3_ (5 : 1, v/v). The NMR titration results (Fig. S64) revealed that the binding constant of PF_6_^−^⊂2d was significantly reduced from 5.6 (±0.5) × 10^3^ M^−1^ in pure CDCl_3_ to 1.5 × 10^2^ M^−1^ in CDCl_3_/CD_3_SOCD_3_ (5 : 1, v/v). This experiment indicated that more polar solvent disfavored host–guest binding by shielding its charge and thus suppressing anion–π interactions. We thus reasonably hypothesize that the solvophobic effect might play a less dominant role compared with anion–π interactions in anion recognition. The cage 2c bearing Cl atoms exhibited similar binding behaviors. In the ^1^H NMR spectra of the complexes PF_6_^−^⊂2c, ClO_4_^−^⊂2c, IO_4_^−^⊂2c and BF_4_^−^⊂2c (Fig. S58–S61), the resonances corresponding to proton a underwent upfield shifts by 1.18, 1.22, 1.14 and 1.23 ppm, respectively (Fig. 3E, left), relative to the “free” 2c. The corresponding binding constants for these complexes were determined to be (8.2 ± 0.3) ×10^2^, (2.2 ± 0.1) ×10^2^, (7.0 ± 0.7) ×10^1^ and (3.0 ± 0.4) ×10^1^ M^−1^, respectively (Table 1).

The addition of these anions with TBA^+^ counterions to the CDCl_3_ solutions of 2e led to little or no shifts (Fig. S70). This outcome is somewhat surprising considering that the framework of 2e is identical to that of either 2c or 2d, aside from the different substituents. One possible explanation is that the phenyl substituents in the framework of 2e introduced steric hindrance to the anionic guests, increasing the energy barriers for host–guest association. This possibility was ruled out by the experiments that the combination of 1e and TREN in the presence of the putative anionic guests produced the “free” cage 2e exclusively (Fig. S71). Another explanation is that the phenyl substituents in 2e are relatively electron-rich, which jeopardized the electron deficient nature of the cage 2e. We thus synthesized 2f, an analogue of 2e with more electron-withdrawing 4-CF_3_Ph units, and tested its anion recognition ability. After adding these anions to the solution of 2f, we observed the formation of host–guest complexes, as evidenced by shifts in the ^1^H NMR spectra, although the complexation did not reach equilibrium until a few days. Therefore, the complexes PF_6_^−^⊂2f, ClO_4_^−^⊂2f, IO_4_^−^⊂2f and BF_4_^−^⊂2f were successfully obtained by self-assembling the cage 2f in the presence of these anionic guests as templates. In the ^1^H NMR spectra (Fig. 3D and S71–S74) of these complexes, the resonances corresponding to proton a underwent upfield shifts by 0.27, 0.29, 0.26 and 0.30 ppm (Fig. 3E, right), respectively, relative to the “free” 2f. These upfield shifts imply an enhanced shielded magnetic effect occurred to the protons a in the complexes compared with the “free” cage 2f. One possible explanation is that the accommodation of anionic guests leads to the expulsion of a solvent molecule (CDCl_3_) from the cage cavity, causing the cage framework to shrink slightly and enhanced CH–π interactions. It is noteworthy that the upfield shifts in 2f were less pronounced than those observed in 2c and 2d. This can be attributed to the fact that 2f undergoes fewer conformational changes during anion accommodation, *i.e.*, the protons a are forced to reside inside the cage cavity by the 4-CF_3_Ph substituents regardless of whether anions are present. *K*_a_ values of PF_6_^−^⊂2f, ClO_4_^−^⊂2f, IO_4_^−^⊂2f and BF_4_^−^⊂2f were determined to be (5.7 ± 0.4) ×10^3^, (1.1 ± 0.3) ×10^3^, (1.2 ± 0.2) ×10^3^ and (9.0 ± 0.7) ×10^1^ M^−1^, respectively (Table 1), by integrating the corresponding resonances for both the complexes and the “free” cage.

**Fig. 4:**
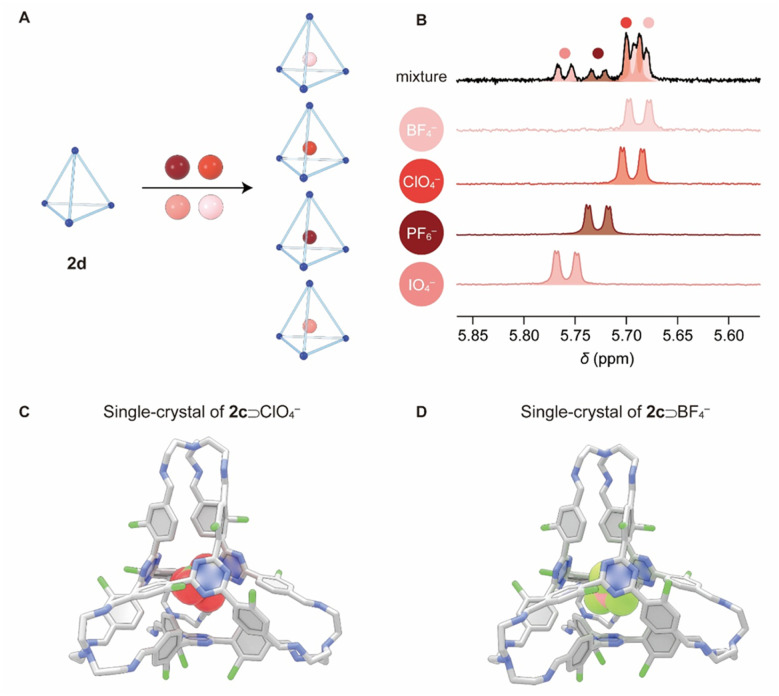
(A) Graphical representation of cage 2d encapsulating four different anions. (B) Partial ^1^H NMR spectra (400 MHz, CDCl_3_, 298 K) of 2d (0.5 mM) after the addition of a mixture of PF_6_^−^, ClO_4_^−^, IO_4_^−^ and BF_4_^−^ (top), each at a concentration of 3 mM, and after the addition of each individual anion (below). The full spectra are shown in the SI. All anions were added as their TBA^+^ salts. Side views of the core structures from single-crystal X-ray diffraction analysis for (C) ClO_4_^−^⊂2c and (D) BF_4_^−^⊂2c. Color code: carbon, gray; nitrogen, blue; chlorine, green; oxygen, red; boron, orange; fluorine, light green. TBA^+^ counterions, hydrogen atoms, and solvent molecules are omitted for clarity.

The ability of cages 2c, 2d, and 2f to produce distinct NMR responses for different anions encouraged us to test their utility as probes to detect multiple anions simultaneously when they are present as a mixture (Fig. 4A). A mixture of TBA^+^ salts of PF_6_^−^, BF_4_^−^, ClO_4_^−^, and IO_4_^−^ (each at 3 mM) was added to a CDCl_3_ solution of 2d (0.5 mM). After the host–guest complexes reached equilibrium, the ^1^H NMR spectrum was recorded. The resonances corresponding to all four complexes namely PF_6_^−^⊂2d, ClO_4_^−^⊂2d, IO_4_^−^⊂2d and BF_4_^−^⊂2d were clearly observed (Fig. 4B). However, for some unknown reasons, the NMR integral ratios corresponding to different complexes do not match the binding constants. The probing ability is a key advantage resulting from the confined cavity of 2d, which led to slow exchange between the “free” cage and each of the different complexes on the timescale of ^1^H NMR spectroscopy. Such discrimination is typically unattainable in traditional systems with “open” structures, such as those reported by Wang,^68,69^ where complexes undergo fast association/dissociation exchange on the NMR timescale, leading to averaged signals for mixtures. The ability of our cage systems to detect multiple anions within a library of mixture simultaneously is reminiscent of the bambusuril rings reported by Sindelar *et al.*^70^

To gain a more quantitative understanding of the kinetic parameters controlling the formation and dissociation of the cage–anion complexes, we employed ^1^H NMR spectroscopy (Fig. S76–S108) to monitor the change in the composition of CDCl_3_ solutions initially containing the “free” cages (0.3 mM) after the addition of an excess of anionic guests (3–9 mM). The observed pseudo-first-order rate constants (*k*_obs_) were first calculated from plots of ln([A]_0_/[A]) *versus* time, where [A]_0_ and [A] represent the concentrations of the “free” cage at the start and at the given time after anion addition, respectively. Plots of *k*_obs_*versus* the initial anion concentrations were then constructed. The rates of association (*k*_f_) were defined by the slopes of these plots, while the rates of dissociation (*k*_b_) were calculated using the equation *k*_b_ = *k*_f_/*K*_a_. For PF_6_^−^⊂2d, ClO_4_^−^⊂2d, IO_4_^−^⊂2d and BF_4_^−^⊂2d, *k*_f_ values were determined to be 30.8, 18.3, 2.3, and 11.3 M^−1^ h^−1^, and *k*_b_ values were determined to be 5.5 × 10^−3^, 18.3 × 10^−3^, 9.2 × 10^−3^, and 70.6 × 10^−3^ h^−1^, respectively. In the case of PF_6_^−^⊂2f, ClO_4_^−^⊂2f, IO_4_^−^⊂2f and BF_4_^−^⊂2f, *k*_f_ values were determined to be 1.06, 1.11, 0.33, and 1.28 M^−1^ h^−1^, and *k*_b_ values were determined to be 0.19 × 10^−3^, 1.0 × 10^−3^, 0.03 × 10^−3^, and 14.2 × 10^−3^ h^−1^, respectively. For each anion, both *k*_f_ and *k*_b_ of 2d are approximately one order of magnitude larger than those of 2f, which is consistent with the fact that, compared with the Br units in 2d, the bulkier 4-CF_3_Ph substituents in 2f impose greater steric hindrance on the anionic guests.

Diffraction grade single crystals of ClO_4_^−^⊂2c·TBA^+^ (Fig. 4C) and BF_4_^−^⊂2c·TBA^+^ (Fig. 4D) were obtained by vapor diffusion of diisopropyl ether into their corresponding chloroform solutions, which unambiguously confirmed the formation of cage–anion complexes. The solid-state structures align with the ^1^H NMR spectroscopic results recorded in solution. As expected, all the Cl substituents in the framework of 2c are positioned outside the cage cavity, endowing the cage with a “free” cavity to accommodate the anionic guests. In the case of ClO_4_^−^⊂2c and BF_4_^−^⊂2c, close contacts were observed between the triazine units in the cages and the oxygen atoms (3.06 Å) in ClO_4_^−^, as well as the fluorine atoms (3.06 Å) of BF_4_^−^, indicating the occurrence of anion–π interactions. The distances between the protons in the *meta* positions relative to imine bonds and the oxygen atoms in ClO_4_^−^ and fluorine atoms in BF_4_^−^ were measured to be in the range of 2.55–2.83 Å, implying the formation of hydrogen bonds as secondary interactions.

**Fig. 5:**
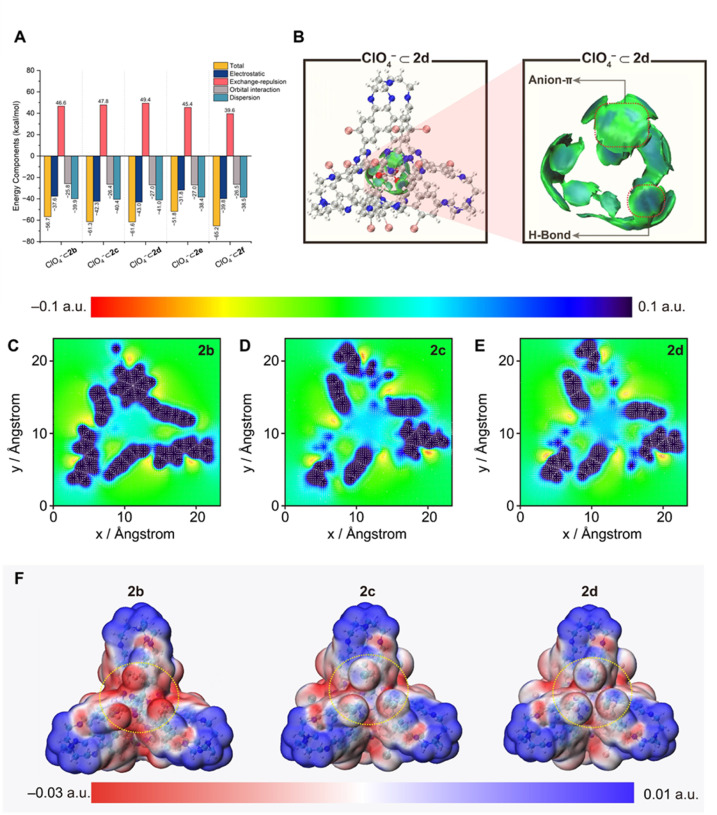
(A) Energy decomposition analysis of ClO_4_^−^⊂2b, ClO_4_^−^⊂2c, ClO_4_^−^⊂2d, ClO_4_^−^⊂2e, and ClO_4_^−^⊂2f. (B) IGMH map showing intermolecular interactions of ClO_4_^−^⊂2d. (C–E) The 2D electrostatic potential distribution and the electric field gradient diagram of 2b (C), 2c (D), and 2d (E). (F) Electrostatic potential distribution color vdW surface maps of 2b (left), 2c (middle), and 2d (right).

The unexpected trend in anion binding affinity prompted a comprehensive computational investigation to elucidate the physical nature of host–guest interactions. DFT calculations were initially employed to optimize the geometries of both the empty cages and their complexes with ClO_4_^−^. Subsequently, energy decomposition analysis (EDA) using the sobEDAw^71^ method was applied to partition the total interaction energy into electrostatic, exchange-repulsion, orbital, and dispersion terms (Fig. 5A and Table S2). The analysis reveals that electrostatic components are the predominant contributions, which govern the observed total interaction energy trend of |Δ*E*_int_(2b)| < |Δ*E*_int_(2c)| < |Δ*E*_int_(2d)| and |Δ*E*_int_(2e)| < |Δ*E*_int_(2f)| (Table S2). These computational trends align closely with the experimental binding affinity. Notably, although the molecular cages are charge-neutral, the dominant electrostatic attraction likely arises from their pronounced electron-deficient character. This finding motivated us to probe deeper into the origin of the observed affinity trend.

The independent gradient model based on Hirshfeld partition of molecular density (IGMH)^72^ analysis using Multiwfn 3.8(dev)^73^ was employed to visualize the interfragmental interactions within the complexes. The resulting isosurfaces (Fig. 5B and S112) display broad, mainly green regions between ClO_4_^−^ and the triazine rings, indicative of attractive anion–π interactions with a substantial dispersion character. Additionally, stronger CH–O hydrogen bond interactions are also evident as the predominately blue isosurface regions between ClO_4_^−^ and the inward-pointing protons at the *meta*-position of the substituent groups. To quantify the contributions of these interactions, we performed quantitative assessments based on calculated quadrupole moment (for anion–π interactions) and AIM topology analysis^74^ (for hydrogen bonds) (see SI, Section 6). The average quadrupole moments (Table S3) of the triazine rings for the empty cages do not correlate with the experimental affinity trend. In contrast, the cumulative hydrogen-bond energies derived from AIM analysis (Table S4) show better agreement, yet they account for only about 30% of the total interaction energy. These findings suggest that neither anion–π interactions nor hydrogen bonding serves as the primary determinant of the observed binding trend.

In an attempt to further probe the electrostatic character of the cage cavity, we analyzed the electrostatic potential (ESP) distribution^75^ of the empty cages. The ESP map (Fig. 5C–E and S109), visualized on a cross-section 3 Å above the triazine plane, reveals a pronounced electropositive region within the cage cavity. Intriguingly, the intensity of this electropositive potential exhibits a clear inverse correlation with the electronegativity of the substituent groups, with the most electronegative F substituent cage 2b having the weakest electropositive cavity. In order to clarify this counterintuitive observation, we derived restrained electrostatic potential (RESP) charges^76^ for each atom. The analysis (Table S6) reveals a slight increase in the net positive charge on the triazine fragments from 2d to 2b, consistent with the expected inductive effect applied by the electronegative halogen substituents. Concomitantly, the twelve halogen atoms exhibit substantial negative charges, with average values increasing in the order: 2d (−0.054 a.u.) < 2c (−0.083 a.u.) < 2b (−0.22 a.u.), as observed (Fig. 5F) on the ESP maps, where electron-rich regions exist over the surface of the halogen atoms.

The significant negative charge localized on the fluorine atoms in 2b motivated us to investigate their electrostatic influence by computing the electrostatic potential energy (Table S7) between cage components and an imaginary unit (−1) charge placed at the center of the cage. While the triazine units generated a notable attractive potential, it is effectively overshadowed by the repulsive potentials arising from the halogen atoms. Consequently, the net electrostatic repulsion follows the order: 2b > 2c > 2d, which aligns with the experimental anion affinity series and rationalizes the primacy of the repulsive field effect exerted by the adjacent halogen atoms in governing anion binding trends.

Finally, the affinity trends for cages 2e and 2f are explained by complementary mechanisms. The absence of observable anion affinity of 2e results from the electron-donating inductive effect of the Ph groups that significantly weaken the overall electrostatic term of attraction (Fig. 5A). In contrast, the strong binding affinity of 2f, where remote CF_3_ groups induce a negligible field effect, cannot be explained solely by an electron-withdrawing inductive effect. Instead, the enhanced affinity of 2f is also attributable to the marked reduction in Pauli repulsion (Table S2). This effect arises from the decreased overlap between the occupied orbitals of the anion and 2f, a consequence of orbital polarization induced by the electron-withdrawing CF_3_ groups.

## Conclusions

To summarize, condensation of a trisamino linker with a series of trisformyl precursors yielded five tetrahedral cages in modest to high yields. Each cage features four electron-deficient faces each containing a triazine unit. Three of these cages utilize their cavities to accommodate anions of complementary sizes through fourfold anion–π interactions. The substituents—all grafted at the *meta* positions relative to the formyl groups—played crucial roles in both self-assembly and anion recognition. First, they preorganized each benzaldehyde unit into a twisted dihedral angle relative to the central triazine core in the precursors. This conformation favored intramolecular CH–π interactions, which served as the key driving force for tetrahedral cage formation. Second, the electronic nature of these substituents has significant impact on anion affinity. Generally, electron-donating groups (*e.g.*, Ph) diminished binding affinity by reducing electrostatic attraction with the anionic guests. In contrast, these attractive forces were increased in cages with electron-withdrawing substituents (*e.g.*, Br, Cl, and CF_3_-Ph), leading to enhanced anion binding. Notably, strongly electron-withdrawing substituents (*e.g.*, F) in close proximity to the binding pocket also weaken the binding affinity through a repulsive field effect. The encapsulation of anionic guests induced conformational changes in the cage framework. This, in turn, perturbed or reinforced the intramolecular CH–π interactions at each corner to a varying extent for each anion. These perturbations produced distinct NMR responses that provided characteristic signatures for differentiating between anions.

## Author contributions

H. L. and Y. L. conceived the concept. Y. L. and H. T. performed the experiments and analyzed the data. P. Z. and G. W. carried out the computational studies. H. C. provided the image models. The manuscript was written through contributions of all authors. All authors have given approval to the final version of the manuscript.

## Conflicts of interest

There is no conflict of interest to report.

## Supplementary Material

SC-017-D5SC08157B-s001

SC-017-D5SC08157B-s002

## Data Availability

CCDC 2483576 and 2483577 contain the supplementary crystallographic data for this paper.^77*a*,*b*^ Supplementary information (SI): synthetic procedures, NMR spectra, ESI-MS spectra, computational details, and X-ray structure details. See DOI: https://doi.org/10.1039/d5sc08157b.
