# Supplementary material for: Neutral anion-detecting organic cages based on anion–π interactions
Source: Chem Sci. 2026 Jan 23;17(12):5993–6004. doi: 10.1039/d5sc08157b (PMC12854773; doi:10.1039/d5sc08157b)
Supplement: SC-017-D5SC08157B-s001 [file SC-017-D5SC08157B-s001.pdf]

Supplementary Information (SI) for Chemical Science.
This journal is © The Royal Society of Chemistry 2026

Neutral Anion-Detecting Organic Cages based on Anion-π Interactions

Yuyang Lu[a+], Ping Zhou[a,b+], Hua Tang[a+], Yating Wu[a], Yueyan Kuang[a], Ze Cao[a], Jiyong Liu[d], Guangcheng Wu[e]*, Hongliang Chen[a,b,c]*, Hao Li[a,b]*

Supporting Information Contents

Supporting Information Contents 2
1. Materials and General Methods 1
2. Synthesis of **1b-1f**...2
3. NMR Spectroscopic Characterization...11
4. Synthesis and Characterization of the Cage Products...27
5. Kinetic and thermodynamic Studies of binding anionic guests based on ^{1}H NMR Spectroscopic results 41
6. Computational Studies 93
7. X-Ray Crystallography 102
8. References...106

1. Materials and General Methods

All reagents and solvents were purchased from commercial sources and used without further purification. Manipulations were performed under a normal atmosphere unless otherwise indicated. Nuclear magnetic resonance (NMR) spectra were recorded at ambient temperature using Bruker AVANCE III 400/500 and Agilent DD2 600 spectrometers, with working frequencies of 400/500/600 and 100/125/150 MHz for ^{1}H and ^{13}C, respectively. Chemical shifts are reported in ppm relative to the residual internal non-deuterated solvent signals ($CDCl_3$: δ = 7.26 ppm). High-resolution mass spectra (HRMS) were performed on an Agilent 6545 Quadruple-Time-Of-flight (Q-TOF) mass spectrometer and TIMS-TOF-FLEX MALDI2, Bruker. X-ray crystallographic data were collected on a Bruker D8 Venture diffractometer. All the reactions that require elevated temperature used oil bath to provide heat. Structural assignments were made with additional information from gCOSY, NOESY experiments.

2. Synthesis of 1b-1f

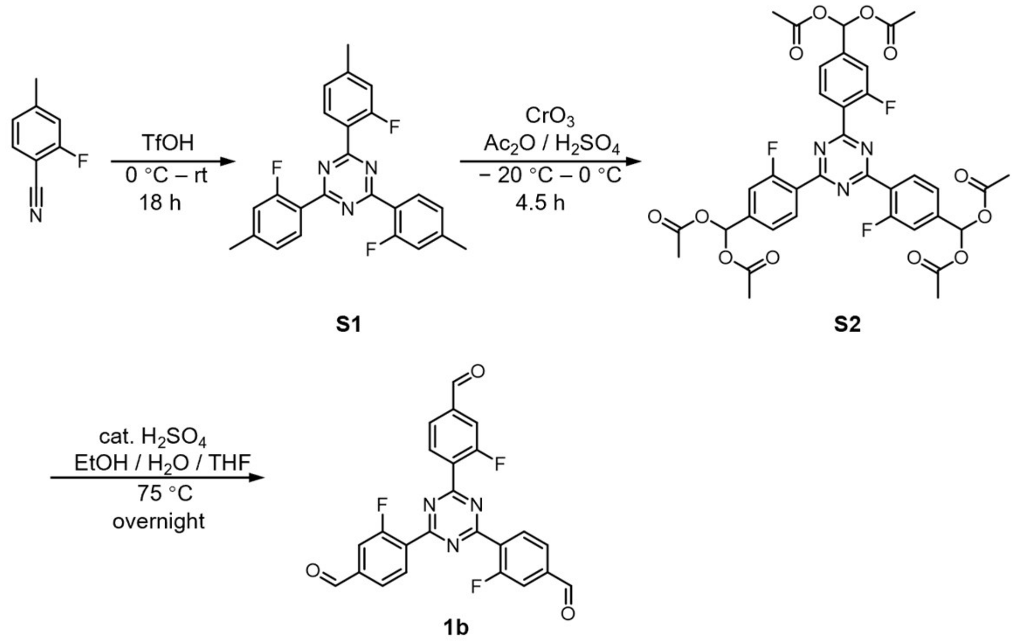

Scheme S1. The synthetic procedure of **1b**[S1]

Compound S1: 5 mL of trifluoromethanesulfonic acid was added dropwise to 2-fluoro-4-methylbenzonitrile (5.00 g, 37.0 mmol) under nitrogen atmosphere at 0 °C. The mixture was kept at 0 °C for an hour and gradually warmed to room temperature and stirred overnight. 30 mL of saturated aqueous $NaHCO_3$ solution was added slowly to neutralize the mixture. The resulting precipitate was extracted with DCM (3 × 20 mL) and wash with saturated brine for three times. The organic phase was dried with anhydrous Na_2SO_4 and the solvent was evaporated to obtain pure **S1** (4.95 g, 99%) as white solid. **^{1}H NMR** (400 MHz, $CDCl_3$): δ = 8.35 (t, J = 8.0 Hz, 3H), 7.12 (dd, J = 8.2, 1.6 Hz, 3H), 7.05 (dd, J = 12.0, 1.6 Hz, 3H), 2.44 (s, 9H). **^{13}C NMR** (100 MHz, $CDCl_3$): δ = 170.5 (d, J = 5.6 Hz), 162.4 (d, J = 260.1 Hz), 144.9 (d, J = 9.0 Hz), 132.1, 125.1 (d, J = 3.2 Hz), 121.8 (d, J = 8.1 Hz), 117.6 (d, J = 21.9 Hz), 21.5. **^{19}F NMR** (376 MHz, $CDCl_3$): δ = 111.2. **MS (ESI-HRMS):** m/z $[M+H]^+$ calcd for $C_{24}H_{19}F_3N_3^+$: 406.1531; found: 406.1528.

Compound S2: **S1** (500.0 mg, 1.23 mmol) was dissolved in acetic anhydride (6.0 mL) and sulfuric acid (1.0 mL; 98%) under −20 °C. A solution of CrO_3 (1.11 g, 11.1 mmol)

in acetic anhydride (5.0 mL) was added slowly to the mixture over a period of 3.5 hours. Then the reaction was stirred under 0 °C for an hour and was subsequently poured into 50 mL of iced water. The resulting yellow solid was filtered off and wash with water. The residue was purified by column chromatography (dichloro-methane/ethyl acetate (80:1); silica gel, 200-300 mesh) to yield **S2** (57.0 mg, 6 %) as colorless solid. **^{1}H NMR** (400 MHz, $CDCl_3$): δ = 8.46 (t, J = 7.8 Hz, 3H), 7.73 (s, 3H), 7.48 (dd, J = 8.1, 1.6 Hz, 3H), 7.42 (dd, J = 11.3, 1.6 Hz, 3H), 2.17 (s, 18H). **^{13}C NMR** (100 MHz, $CDCl_3$): δ = 170.4, 168.6, 162.3 (d, J = 262.0 Hz), 141.4 (d, J = 8.2 Hz), 132.8, 125.4, 122.6, 115.7 (d, J = 23.9 Hz), 88.4, 20.8. **^{19}F NMR** (376 MHz, $CDCl_3$): δ = 109.1. **MS (ESI-HRMS)**: m/z $[M+H]^+$ calcd for $C_{36}H_{31}F_3N_3O_{12}^+$: 754.1860; found: 754.1856.

Compound 1b: 98% sulfuric acid (0.1 mL) was added dropwise to the solution of **S2** (50.0 mg, 0.066 mmol) in a mixture of EtOH (1.0 mL) and H_2O (0.8 mL). The reaction was refluxed for 12 h and then cooled to room temperature. Saturated aqueous $NaHCO_3$ solution (2 mL) was added to neutralize the mixture. The resulting precipitation was filtered off and washed with water. The residue was dried under vaccum to obtain pure **1b** (28.0 mg, 95%) as yellow solid. **^{1}H NMR** (600 MHz, $CDCl_3$): δ = 10.12 (d, J = 1.4 Hz, 3H), 8.64 (t, J = 7.5 Hz, 3H), 7.88 (dd, J = 8.0, 1.5 Hz, 3H), 7.79 (dd, J = 10.4, 1.5 Hz, 3H). **^{13}C NMR** (150 MHz, $CDCl_3$): δ = 190.3, 170.4 (d, J = 5.5 Hz), 162.7 (d, J = 264.2 Hz), 140.6 (d, J = 7.1 Hz), 133.3, 129.1 (d, J = 8.3 Hz), 125.4 (d, J = 3.9 Hz), 117.6 (d, J = 22.9 Hz). **^{19}F NMR** (376 MHz, $CDCl_3$): δ = 108.4. **MS (ESI-HRMS):** m/z $[M+H]^+$ calcd for $C_{24}H_{13}F_3N_3O_3^+$: 448.0909; found: 448.0901.

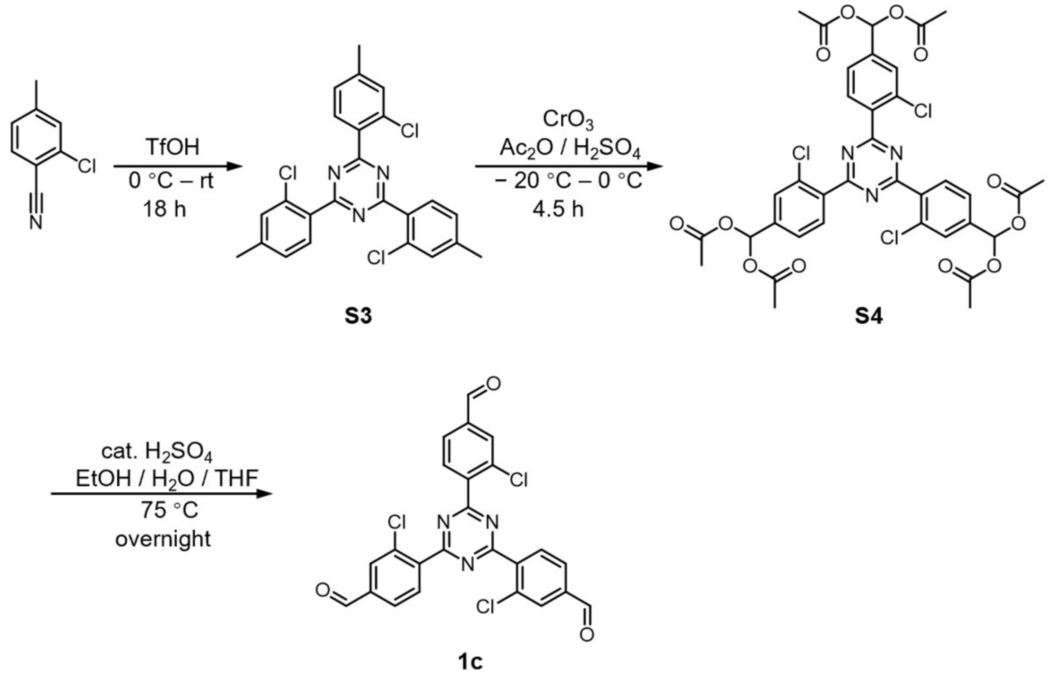

Scheme S2. Synthesis of **1c**.

Compound S3: 5 mL of trifluoromethanesulfonic acid was added dropwise to 2-chloro-4-methylbenzonitrile (5.00 g, 33.0 mmol) under nitrogen atmosphere at 0 °C. The mixture was kept at 0 °C for an hour and gradually warmed to room temperature and stirred overnight. 30 mL of saturated aqueous $NaHCO_3$ solution was added slowly to neutralize the mixture. The resulting precipitate was extracted with DCM (3 × 20 mL) and wash with saturated brine for three times. The organic phase was dried with anhydrous Na_2SO_4 and the solvent was evaporated, yielding a white solid. The latter was purified by column chromatography (petroleum ether/ethyl acetate (8:1); silica gel, 200-300 mesh) to yield **S3** (3.76 g, 75%) as a white solid. **^{1}H NMR** (400 MHz, $CDCl_3$): δ = 8.06 (d, *J* = 7.9 Hz, 3H), 7.38 (d, *J* = 1.6 Hz, 3H), 7.23 (dd, *J* = 7.9, 1.7 Hz, 3H), 2.41 (s, 9H). **^{13}C NMR** (100 MHz, $CDCl_3$): δ = 172.4, 142.7, 133.5, 132.7, 132.6, 131.7, 127.8, 21.2. **MS (ESI-HRMS):** m/z $[M+H]^+$ calcd for $C_{24}H_{19}Cl_3N_3^+$: 454.0645; found: 454.0639.

Compound S4: **S3** (360.0 mg, 0.79 mmol) was dissolved in 2.6 mL of acetic anhydride and 0.8 mL of sulfuric acid (98%) under −20 °C. The solution of CrO_3 (713.0 mg, 7.13 mmol) in 3.3 mL of acetic anhydride was added slowly to the mixture over a period of

3.5 hours. Then the reaction was stirred under 0 °C for an hour and was subsequently poured into 50 mL of iced water. The resulting yellow solid was filtered off and wash with water. The residue was purified by column chromatography (dichloro-methane/ethyl acetate, 80∶1); silica gel, 200-300 mesh) to yield **S4** (50.0 mg, 8%) as a colorless solid. **^{1}H NMR** (400 MHz, $CDCl_3$): δ = 8.16 (d, *J* = 8.1 Hz, 3H), 7.73 (d, *J* = 1.6 Hz, 3H), 7.71 (s, 3H), 7.58 (dd, *J* = 8.1, 1.7 Hz, 3H), 2.17 (s, 18H). **^{13}C NMR** (100 MHz, $CDCl_3$): δ = 172.2, 168.6, 139.6, 136.1, 134.2, 133.0, 129.5, 125.4, 88.4, 20.8. **MS (ESI-HRMS):** m/z $[M+Na]^+$ calcd for $C_{36}H_{30}Cl_3N_3NaO_{12}{}^+$: 824.0793; found: 824.0787.

Compound 1c: Sulfuric acid (0.15 mL; 98%) was added dropwise to a solution of **S6** (100.0 mg, 0.12 mmol) in 2.5 mL of EtOH and 2 mL of H_2O. The reaction was refluxed for 12 h and then cooled to room temperature. 2.5 mL of saturated aqueous $NaHCO_3$ solution was added to neutralize the mixture. The resulting precipitation was filtered off and washed with water. The residue was dried under vaccum to obtain pure **1b** (56.0 mg, 94%) as pale solid. **^{1}H NMR** (600 MHz, $CDCl_3$): δ = 10.10 (s, 3H), 8.29 (d, *J* = 7.9 Hz, 3H), 8.10 (d, *J* = 1.5 Hz, 3H), 7.97 (dd, *J* = 8.0, 1.5 Hz, 3H). **^{13}C NMR** (151 MHz, $CDCl_3$): δ = 190.3, 172.3, 139.5, 138.8, 135.0, 133.4, 132.1, 127.7. **MS (ESI-HRMS):** m/z $[M+H]^+$ calcd for $C_{24}H_{13}Cl_3N_3O_3{}^+$: 824.0793; found: 824.0787.

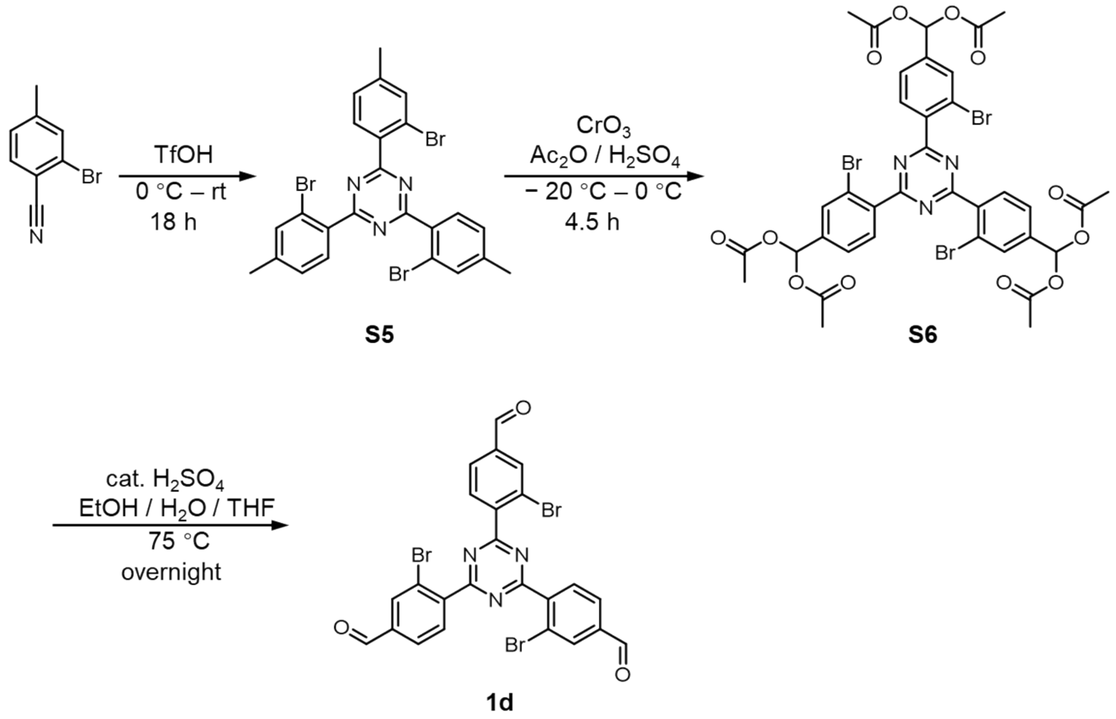

Scheme S3. Synthesis of **1d**.

Compound S5: Trifluoromethanesulfonic acid (5 mL) was added dropwise to 2-bromo-4-methylbenzonitrile (5.00 g, 25.5 mmol) under nitrogen atmosphere at 0 °C. The mixture was kept at 0 °C for an hour and gradually warmed to room temperature and stirred overnight. 30 mL of saturated aqueous $NaHCO_3$ solution was added slowly to neutralize the mixture. The resulting precipitate was extracted with DCM (3 × 20 mL) and wash with saturated brine for three times. The organic phase was dried with anhydrous Na_2SO_4 and the solvent was evaporated to yield a white solid, which was purified by column chromatography (petroleum ether/ethyl acetate (10:1); silica gel, 200-300 mesh) to yield **S5** (3.12 g, 62%) as a white solid. **^{1}H NMR** (400 MHz, $CDCl_3$): δ = 8.07 (d, J = 7.9 Hz, 3H), 7.59 (s, 3H), 7.28 (d, J = 8.2 Hz, 3H), 2.41 (s, 9H). **^{13}C NMR** (101 MHz, $CDCl_3$): δ = 172.9, 142.7, 135.0, 134.3, 132.7, 128.4, 122.0, 21.1. **MS (ESI-HRMS):** m/z $[M+H]^+$ calcd for $C_{24}H_{19}Br_3N_3^+$: 587.9109; found: 587.9107.

Compound S6: **S5** (500.0 mg, 0.85 mmol) was dissolved in 3 mL of acetic anhydride and 0.6 mL of sulfuric acid (98%) under −20 °C. The solution of CrO_3 (765.0 mg, 7.65 mmol) in 3.75 mL of acetic anhydride was added slowly to the mixture over a period

of 3.5 hours. Then the reaction was stirred under 0 °C for an hour and was subsequently poured into 50 mL of iced water. The resulting yellow solid was filtered off and wash with water. The residue was purified by column chromatography (dichloro-methane/ethyl acetate, 100:1); silica gel, 200-300 mesh) to yield **S6** (198.0 mg, 25%) as colorless solid. **^{1}H NMR** (400 MHz, $CDCl_3$): δ = 8.16 (d, *J* = 8.1 Hz, 3H), 7.93 (d, *J* = 1.7 Hz, 3H), 7.70 (s, 3H), 7.63 (dd, *J* = 8.1, 1.7 Hz, 3H), 2.16 (s, 18H). **^{13}C NMR** (101 MHz, $CDCl_3$): δ = 172.9, 168.6, 139.5, 137.9, 132.9, 132.8, 126.0, 122.3, 88.3, 20.8. **MS (ESI-HRMS):** m/z $[M+Na]^+$ calcd for $C_{36}H_{30}Br_3N_3NaO_{12}^+$: 955.9277; found: 955.9274.

Compound 1d: Sulfuric acid (0.25 mL; 98%) was added dropwise to the solution of **S6** (250.0 mg, 0.27 mmol) in 2.5 mL of EtOH and 2mL of H_2O. The reaction was refluxed for 12 h and then cooled to room temperature. 5 mL of saturated $NaHCO_3$ solution was added to neutralize the mixture. The resulting precipitation was filtered off and washed with water. The residue was dried under vaccum to obtain pure **1d** (165.0 mg, 97%) as yellow solid. **^{1}H NMR** (600 MHz, $CDCl_3$): δ = 10.09 (s, 3H), 8.29 (d, *J* = 1.5 Hz, 3H), 8.27 (d, *J* = 7.9 Hz, 3H), 8.02 (dd, *J* = 7.9, 1.5 Hz, 3H). **^{13}C NMR** (150 MHz, $CDCl_3$): δ = 190.1, 173.0, 141.3, 138.7, 135.4, 133.3, 128.3, 123.0. Mass spectrum was recorded. However, molecular ion peaks were not found, probably due to the molecule is difficult to undergo ionization.

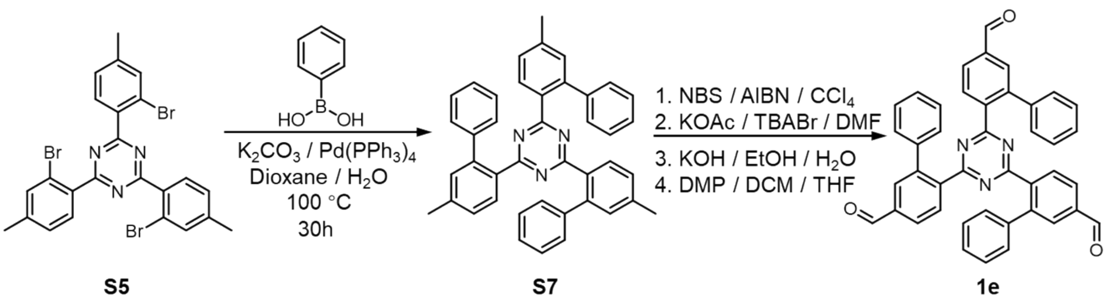

Scheme S4. Synthesis of **1e**.

Compound S7: **S5** (520.0 mg, 0.88 mmol) and phenylboronic acid (539.0 mg, 4.42 mmol) were dissolved in a mixture solvent consisting of dioxane (10 mL) and H_2O (2

mL). The reaction mixture was degassed for 30 min, before $Pd(PPh_3)_4$ (51.0 mg, 0.044 mmol) and K_2CO_3 (1.10 g, 8.0 mmol) were added. The reaction mixture was stirred at 100 °C under an N_2 atmosphere for 36 h. After cooling to room temperature, the solvent was removed under vacuum and the residue was poured into water and then extracted with CH_2Cl_2 (3 × 20 mL). The resulting organic extract was washed with water (2 × 15 mL) and brine (3 × 10 mL), dried over anhydrous Na_2SO_4, and then concentrated to give the crude product, which was purified by column chromatography (petroleum ether/ethyl acetate (10:1); silica gel, 200-300 mesh) to yield **S7** (500.0 mg, 98%) as white solid. **^{1}H NMR** (400 MHz, $CDCl_3$): δ = 7.37 – 7.27 (m, 9H), 7.17 (dt, *J* = 6.0, 1.6 Hz, 9H), 7.02 (dd, *J* = 8.0, 1.8 Hz, 3H), 6.77 (d, *J* = 7.9 Hz, 3H), 2.40 (s, 9H). **^{13}C NMR** (100MHz, $CDCl_3$): δ = 173.4, 142.7, 142.5, 140.5, 133.2, 131.6, 131.4, 129.2, 128.0, 127.8, 126.4, 21.4. **MS (ESI-HRMS):** m/z $[M+H]^+$ calcd for $C_{42}H_{34}N_3^+$: 580.2753; found: 580.2747.

Compound 1e: A mixture of **S7** (600.0 mg, 1.04 mmol), N-bromosuccinimide (NBS, 833.0 mg, 4.68 mmol), and azodiisobutyronitrile (AIBN, 16.0 mg, 0.1 mmol) in CCl_4 (30 mL) was stirred and heated to reflux for 12 h. When the reaction was complete, the solvent was removed under reduced pressure and the residue was poured into water. The aqueous mixture was extracted with CH_2Cl_2. The organic phase was washed with brine and dried over anhydrous Na_2SO_4. The solvent was removed under reduced pressure to give a white solid, to which KOAc (1.53 g, 15.6 mmol), TBABr (503.0 mg, 1.56 mmol) and DMF (20 mL) was added. The reaction mixture was then stirred at 100ºC overnight. After cooling down to room temperature, the mixture was extracted with ethyl acetate and water. The organic phase was washed with brine and dried over anhydrous Na_2SO_4, and then concentrated to give yellow liquid, which was dissolved in 10 mL of MeOH. KOH (0.88 g, 15.6 mmol) was added to the solution and the reaction was refluxed overnight. After cooling down to room temperature, the mixture was extracted with ethyl acetate and water, dried over anhydrous Na_2SO_4, and the solvent was evaporated under reduced pressure. The residue was dissolved in 10 mL of THF and 15 mL of CH_2Cl_2. Dess-Martin periodinane (1.76 g, 4.16 mmol) was added

in small portions under 0 °C and then the reaction was stirred for another 4 h under room temperature. The resultant mixture was filtered and the filtrate was concentrated to give crude product, which was then purified by column chromatography on silica gel (Petroleum Ether / CH_2Cl_2, 1:9); silica gel, 200-300 mesh) to give **1e** as white solid (126.0 mg, 20%). **^{1}H NMR** (400 MHz, $CDCl_3$): δ = 10.10 (s, 3H), 7.90 (d, J = 1.6 Hz, 3H), 7.77 (dd, J = 8.0, 1.6 Hz, 3H), 7.38 (dd, J = 5.2, 1.9 Hz, 9H), 7.19 – 7.12 (m, 6H), 7.02 (d, J = 7.9 Hz, 3H). **^{13}C NMR** (100 MHz, $CDCl_3$): δ = 191.7, 173.4, 143.6, 140.8, 140.3, 137.5, 132.2, 131.9, 129.1, 128.5, 127.8, 127.4. **MS (ESI-HRMS):** m/z $[M+Na]^+$ calcd for $C_{42}H_{27}N_3NaO_3^+$: 644.1950; found: 644.1945.

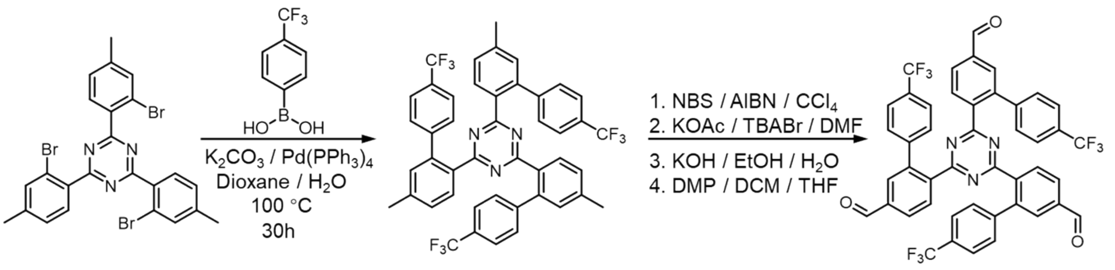

Scheme S5. Synthesis of **1f**.

Compound S8: **S5** (550.0 mg, 0.94 mmol) and 4-(trifluoromethyl)phenylboronic acid (888 mg, 4.68 mmol) were dissolved in a mixture solvent consisting of dioxane (10 mL) and H_2O (2 mL). The reaction mixture was degassed for 30 min, before $Pd(PPh_3)_4$ (54.0 mg, 0.047 mmol) and K_2CO_3 (1.16 g, 8.42 mmol) were added. The reaction was stirred at 100 °C under an N_2 atmosphere for 48 h. After cooling to room temperature, the solvent was removed under vacuum and the residue was poured into water and then extracted with CH_2Cl_2 (3 × 20 mL). The resulting organic extract was washed with water (2 × 15 mL) and brine (3 × 10 mL), dried over anhydrous Na_2SO_4, and then concentrated to give the crude product, which was purified by column chromatography (petroleum ether/ethyl acetate (15:1); silica gel, 200-300 mesh) to yield **S8** (552.0 mg, 75%) as white solid. **^{1}H NMR** (400 MHz, $CDCl_3$): δ = 7.59 (d, J = 8.1 Hz, 6H), 7.26 (d, J = 8.0 Hz, 6H), 7.13 (d, J = 1.7 Hz, 3H), 7.05 (dd, J = 8.2, 1.8 Hz, 3H), 6.73 (d, J = 7.9 Hz, 3H), 2.41 (s, 9H). **^{13}C NMR** (100 MHz, $CDCl_3$): δ = 172.4, 146.9, 141.5,

141.3, 132.3, 131.6, 131.1, 129.3, 128.6, 124.9, 124.9, 21.4. **^{19}F NMR** (376 MHz, $CDCl_3$): δ = 62.24. **MS (ESI-HRMS):** m/z $[M+H]^+$ calcd for $C_{45}H_{31}F_9N_3^+$: 784.2374; found: 784.2369.

Compound 1f: A mixture of **S8** (1.05 g, 1.34 mmol), NBS (1.07 g, 6.03 mmol), and AIBN (16.0 mg, 0.1 mmol) in CCl_4 (50 mL) was stirred and heated to reflux for 12 h. When the reaction was complete, the solvent was removed under reduced pressure and the residue was extracted with CH_2Cl_2 and water. The organic phase was washed with brine and dried over anhydrous Na_2SO_4. The solvent was removed under reduced pressure to give white solid, to which KOAc (1.97 g, 20.1 mmol), TBABr (648.0 mg, 2.01 mmol) and DMF (25 mL) was added. The reaction mixture was then stirred at 100 ºC overnight. After cooling down to room temperature, the mixture was extracted with ethyl acetate and water. The organic phase was washed with brine and dried over anhydrous Na_2SO_4, and then concentrated to give yellow liquid, which was dissolved in 15 mL of MeOH. KOH (1.13 g, 20.1 mmol) was added to the solution and the reaction was refluxed overnight. After cooling down to room temperature, the mixture was extracted with ethyl acetate and water, dried over anhydrous Na_2SO_4, and the solvent was evaporated under reduced pressure. The residue was dissolved in 20 mL of THF and 30 mL of CH_2Cl_2. Dess-Martin periodinane (2.56 g, 6.03 mmol) was added in small portions under 0 °C and then the reaction was stirred for another 4 h under room temperature. The resultant mixture was filtered and the filtrate was concentrated to give crude product, which was then purified by column chromatography on silica gel (Petroleum Ether / CH_2Cl_2, 1:4); silica gel, 200-300 mesh) to give **1f** as white solid (260.0 mg, 23%). **^{1}H NMR** (400 MHz, $CDCl_3$): δ = 10.11 (s, 3H), 7.89 (d, *J* = 1.5 Hz, 3H), 7.79 (dd, *J* = 8.0, 1.6 Hz, 3H), 7.65 (d, *J* = 8.1 Hz, 6H), 7.29 – 7.26 (m, 6H), 7.02 (d, *J* = 7.9 Hz, 3H). **^{13}C NMR** (100 MHz, $CDCl_3$): δ = 191.1, 172.6, 144.9, 142.2, 139.5, 137.9, 131.7, 131.4, 129.4, 129.1, 125.4, 125.3, 125.3. **^{19}F NMR** (376 MHz, $CDCl_3$): δ = 62.43. **MS (ESI-HRMS):** m/z $[M+Na]^+$ calcd for $C_{45}H_{24}F_9N_3NaO_3^+$: 848.1572; found: 848.1566.

3. NMR Spectroscopic Characterization

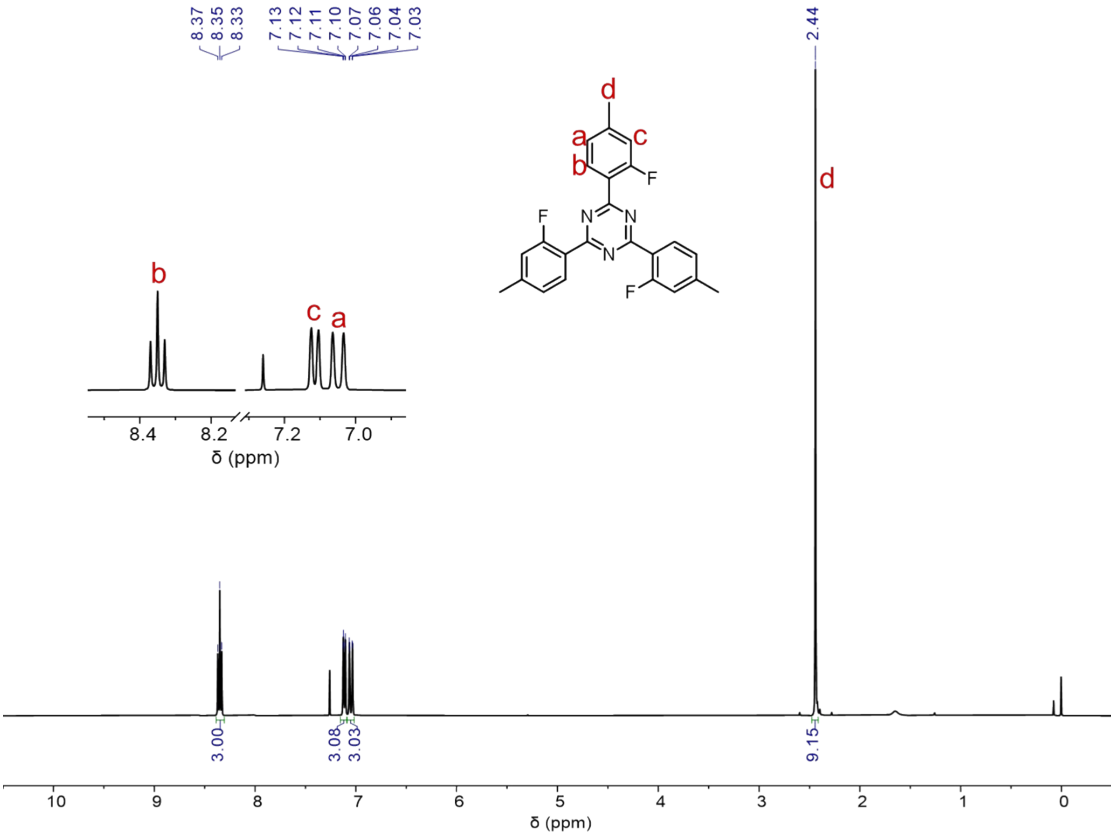

Figure S1. ^{1}H NMR spectrum of **S1** (400 MHz, $CDCl_3$, 298 K).

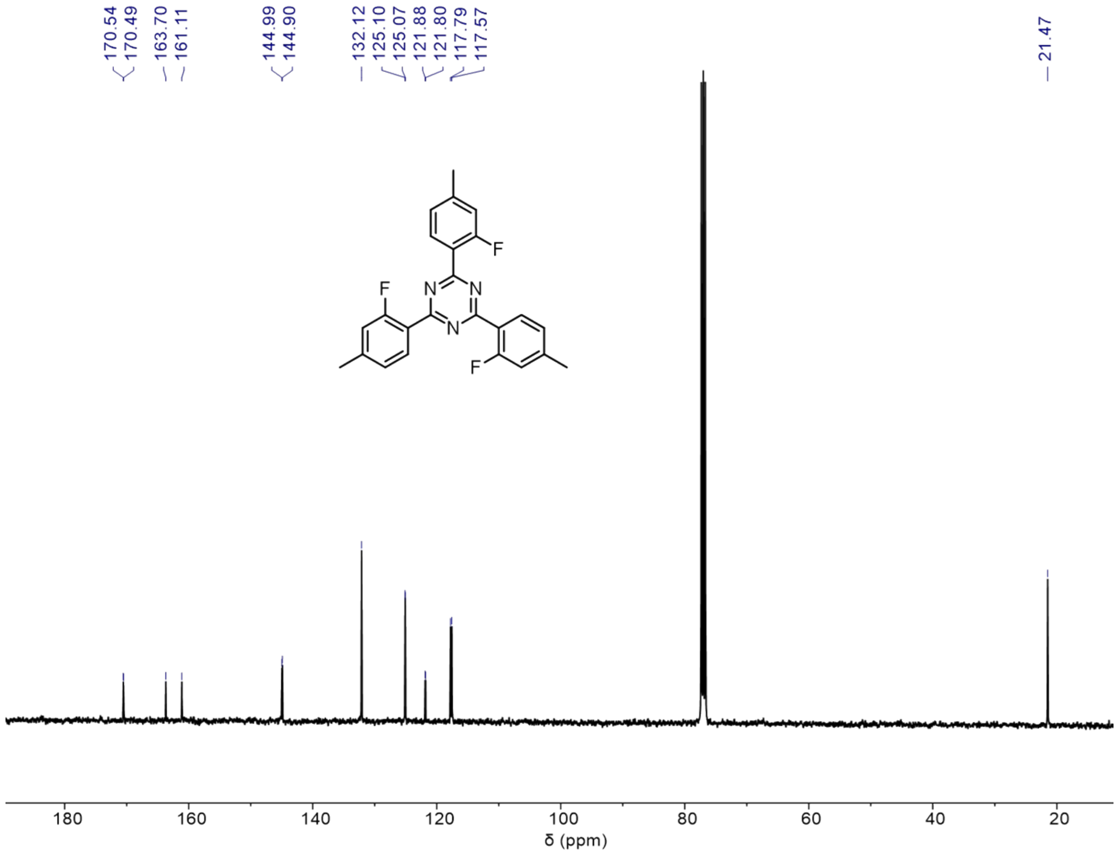

Figure S2. ^{13}C NMR spectrum of **S1** (100 MHz, $CDCl_3$, 298 K).

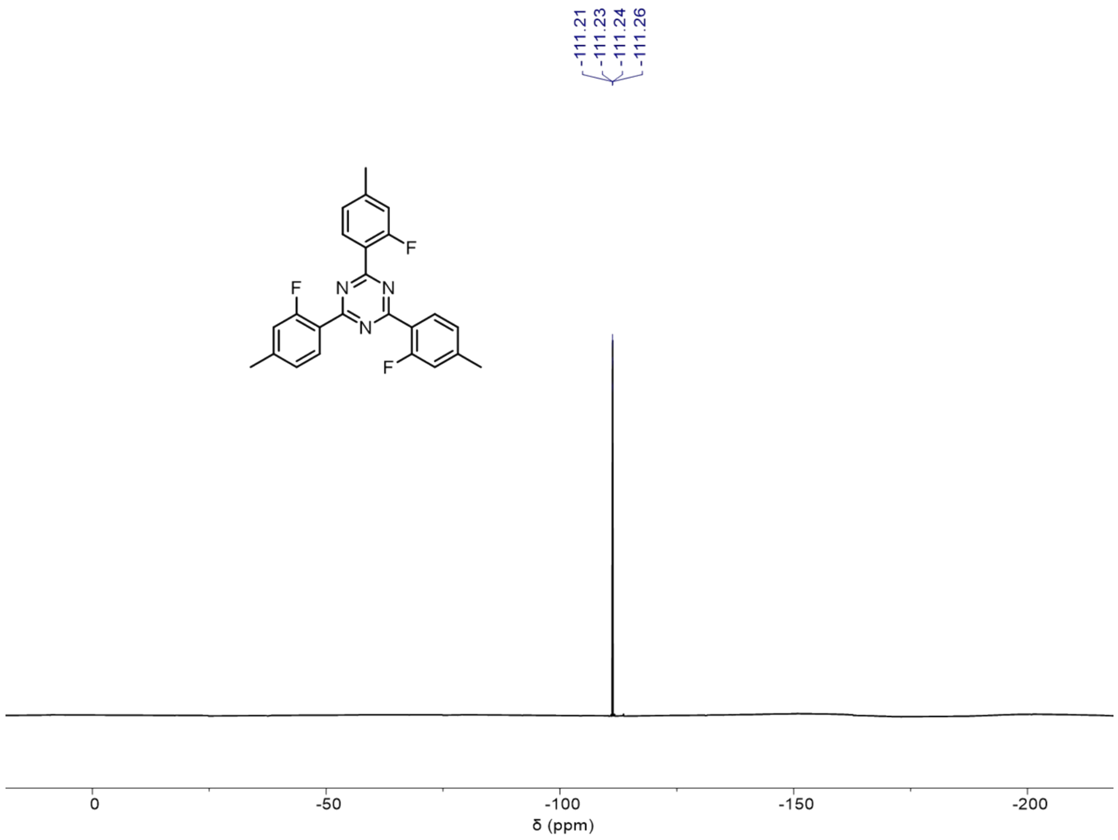

Figure S3. ^{19}F NMR spectrum of **S1** (376 MHz, $CDCl_3$, 298K).

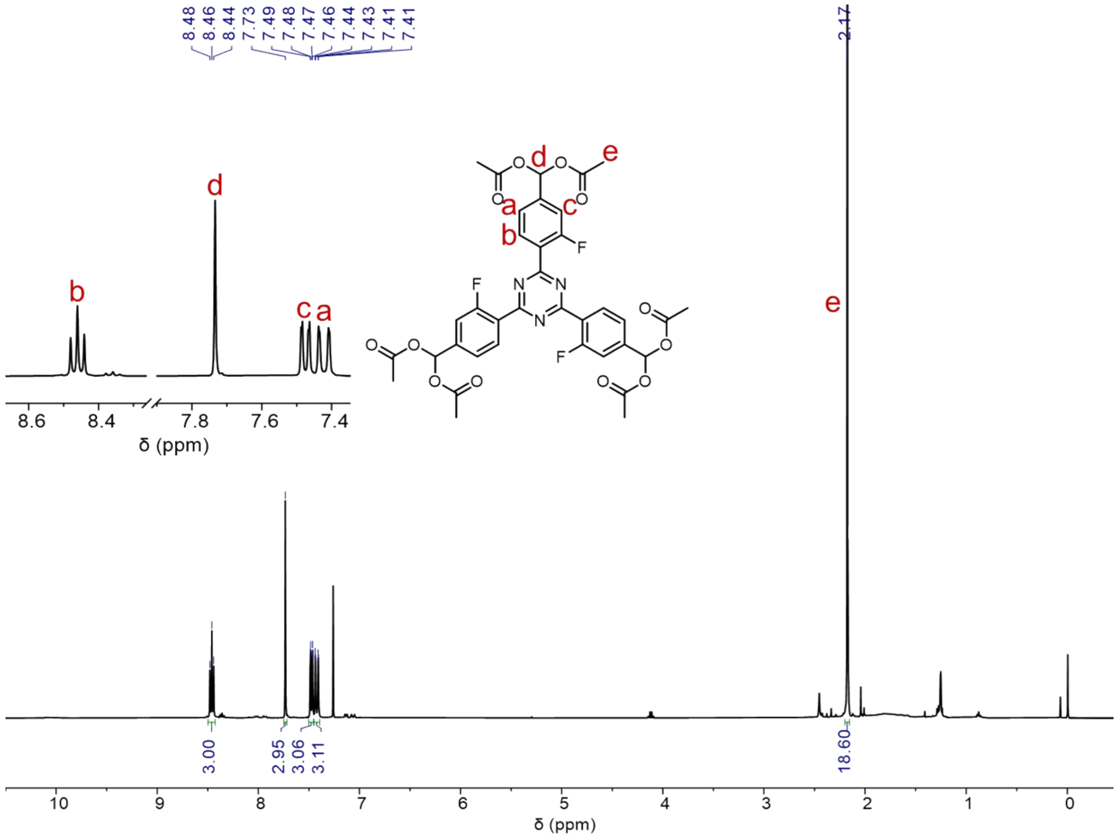

Figure S4. ^{1}H NMR spectrum of **S2** (400 MHz, $CDCl_3$, 298 K).

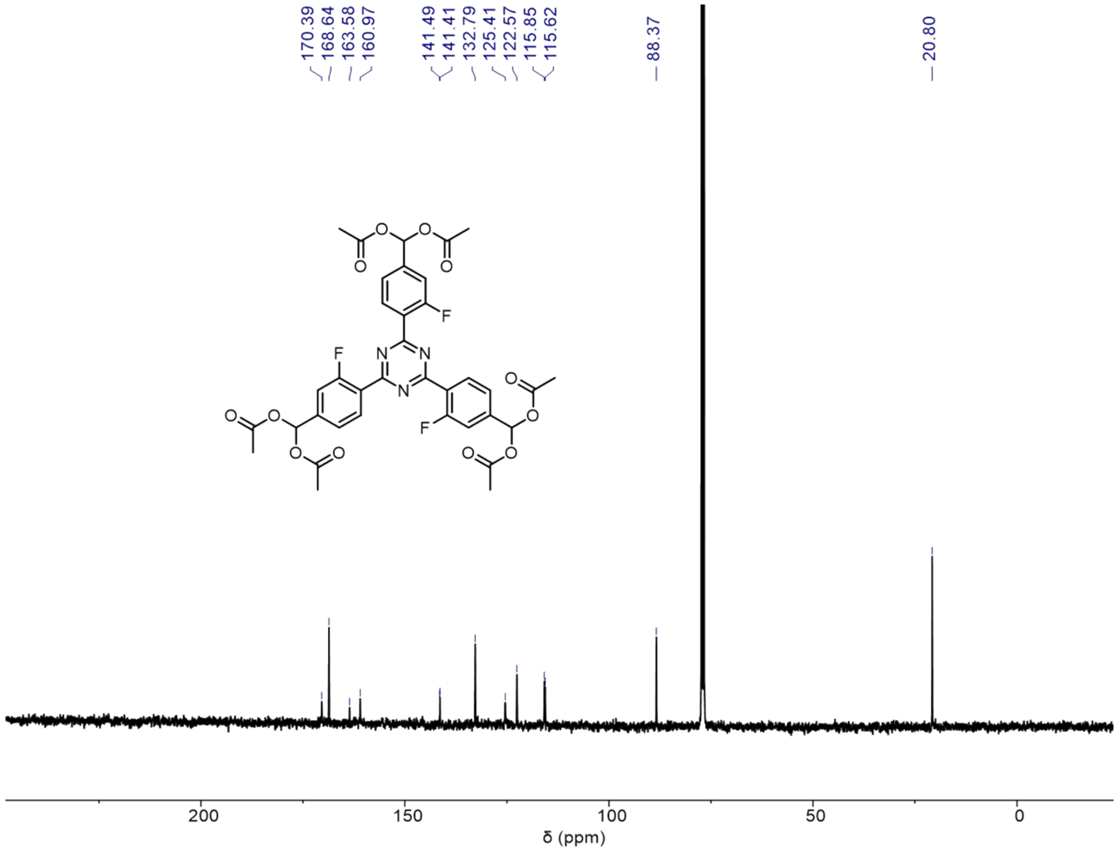

Figure S5. ^{13}C NMR spectrum of **S2** (100 MHz, $CDCl_3$, 298 K).

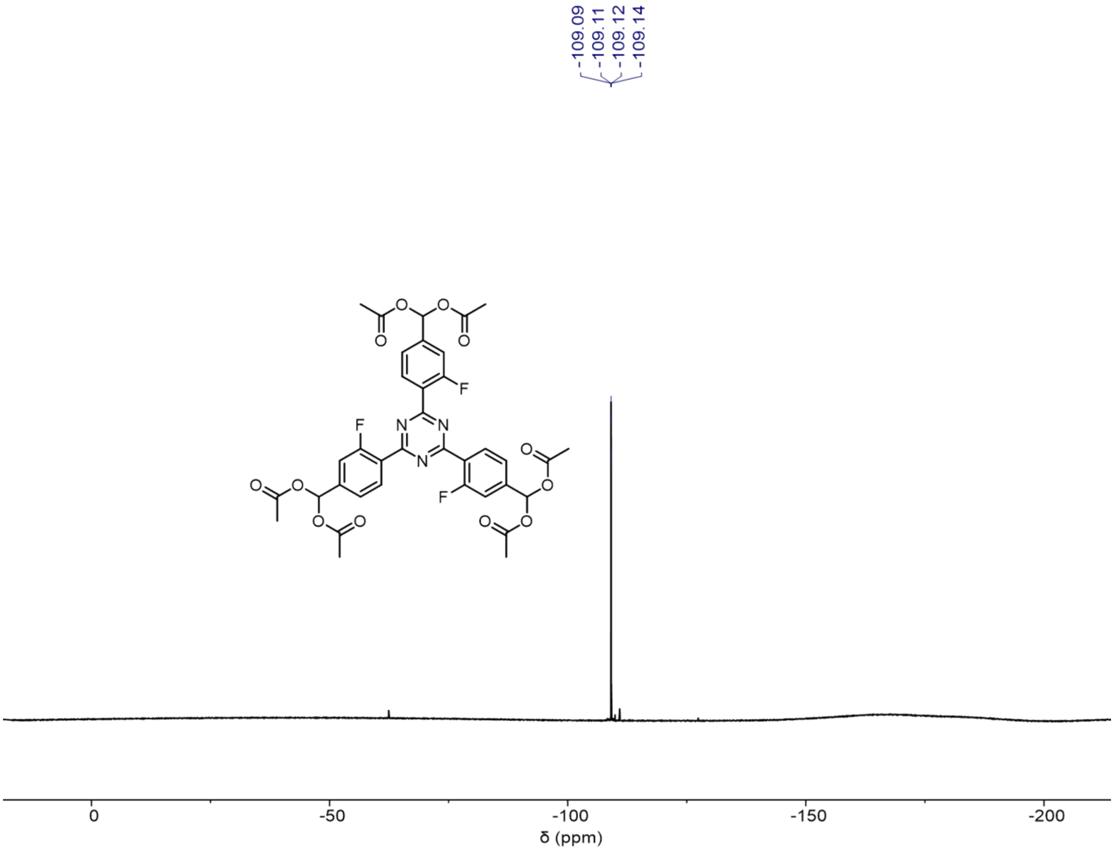

Figure S6. ^{19}F NMR spectrum of **S2** (376 MHz, $CDCl_3$, 298K).

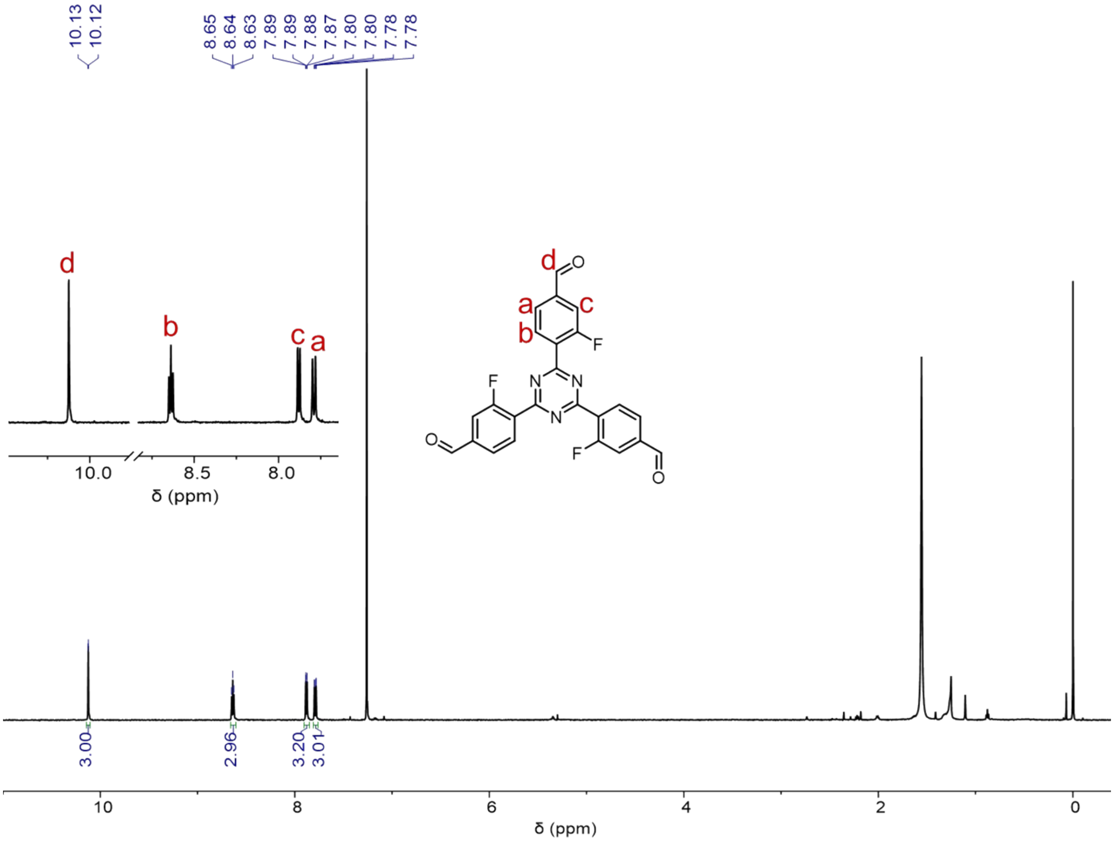

Figure S7. ^{1}H NMR spectrum of **1b** (600 MHz, $CDCl_3$, 298 K).

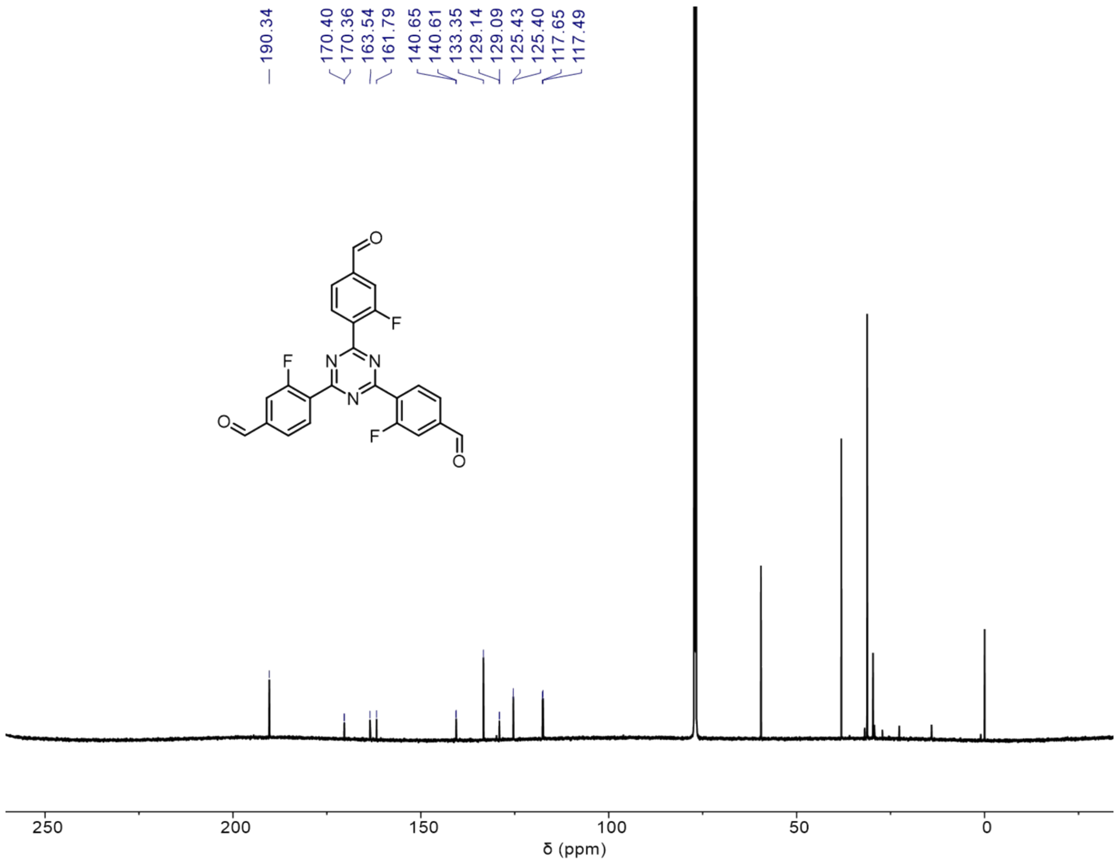

Figure S8. ^{13}C NMR spectrum of **1b** (150 MHz, $CDCl_3$, 298 K).

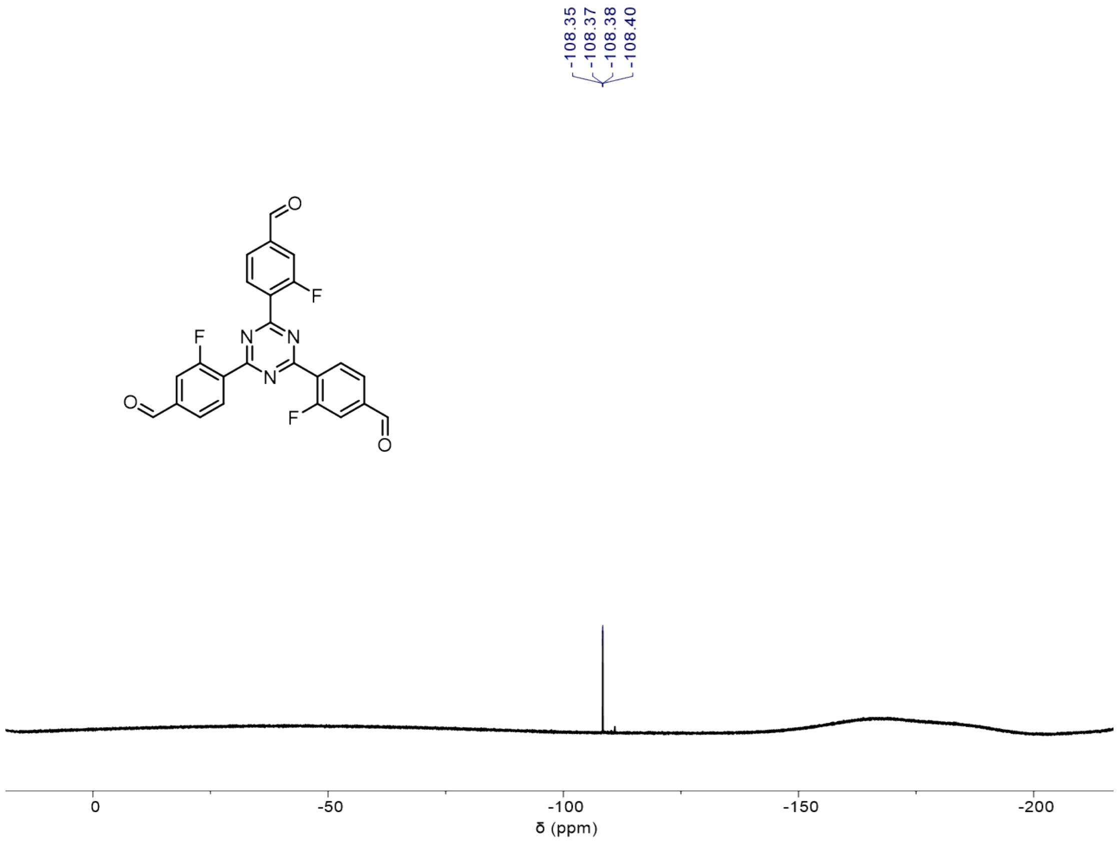

Figure S9. ^{19}F NMR spectrum of **1b** (376 MHz, $CDCl_3$, 298K).

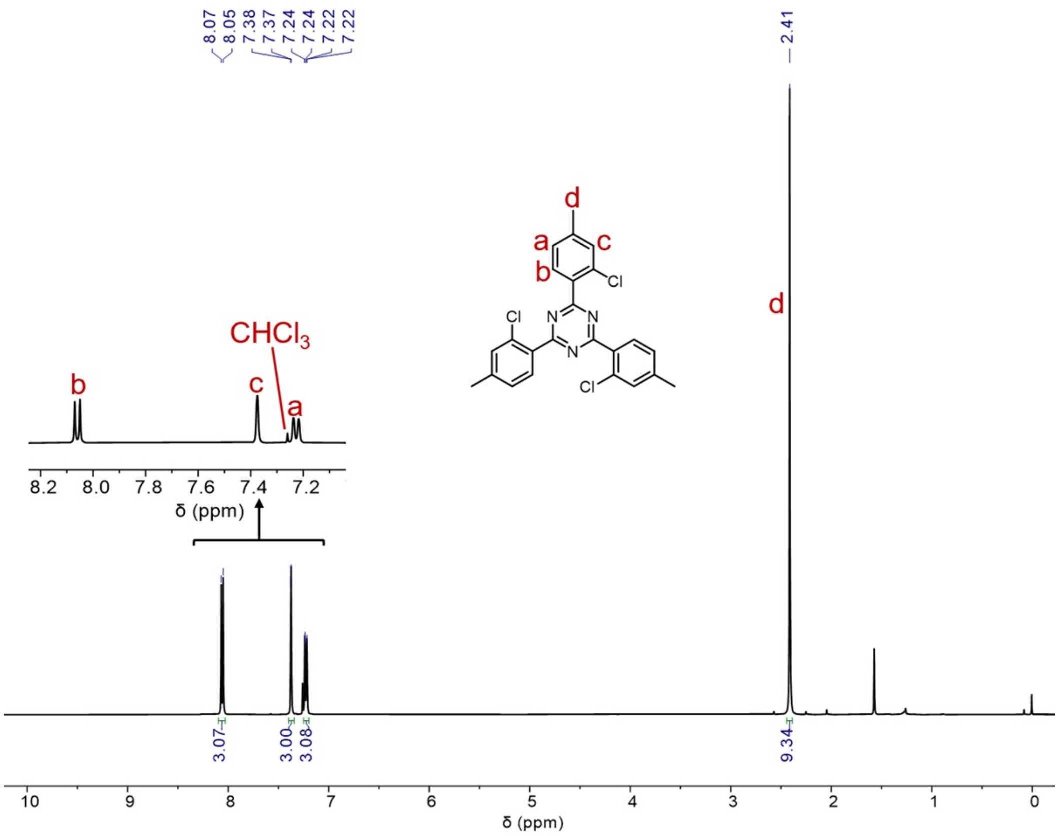

Figure S10. ^{1}H NMR spectrum of **S3** (400 MHz, $CDCl_3$, 298 K).

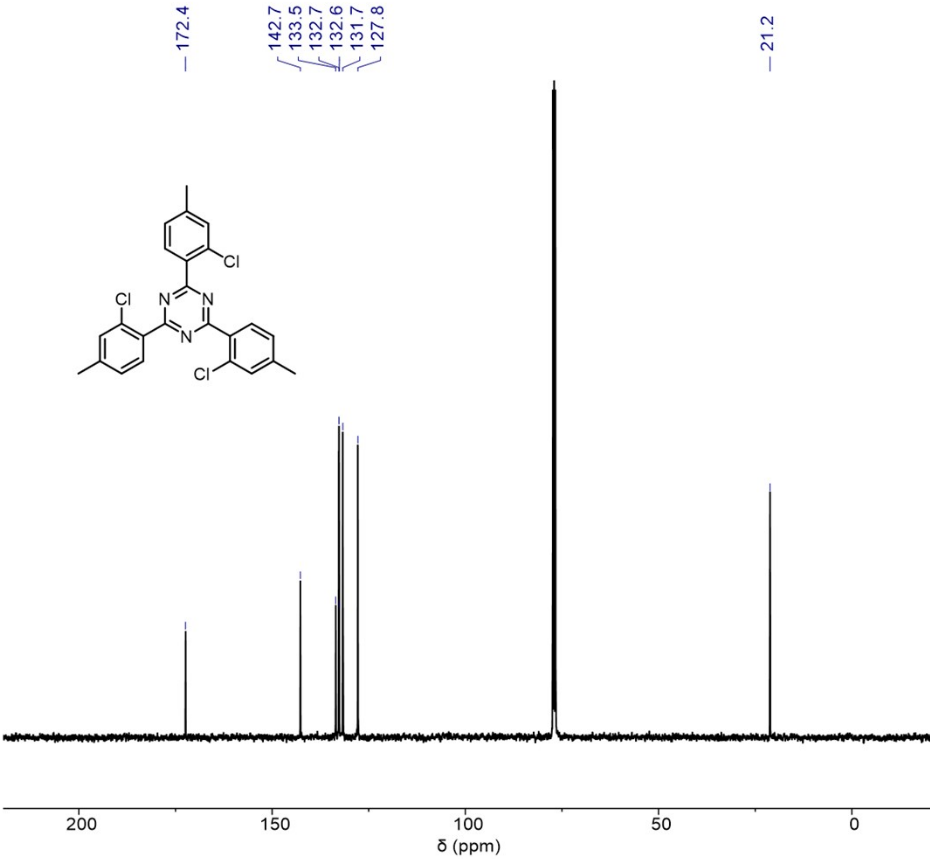

Figure S11. ^{13}C NMR spectrum of **S3** (100 MHz, $CDCl_3$, 298 K).

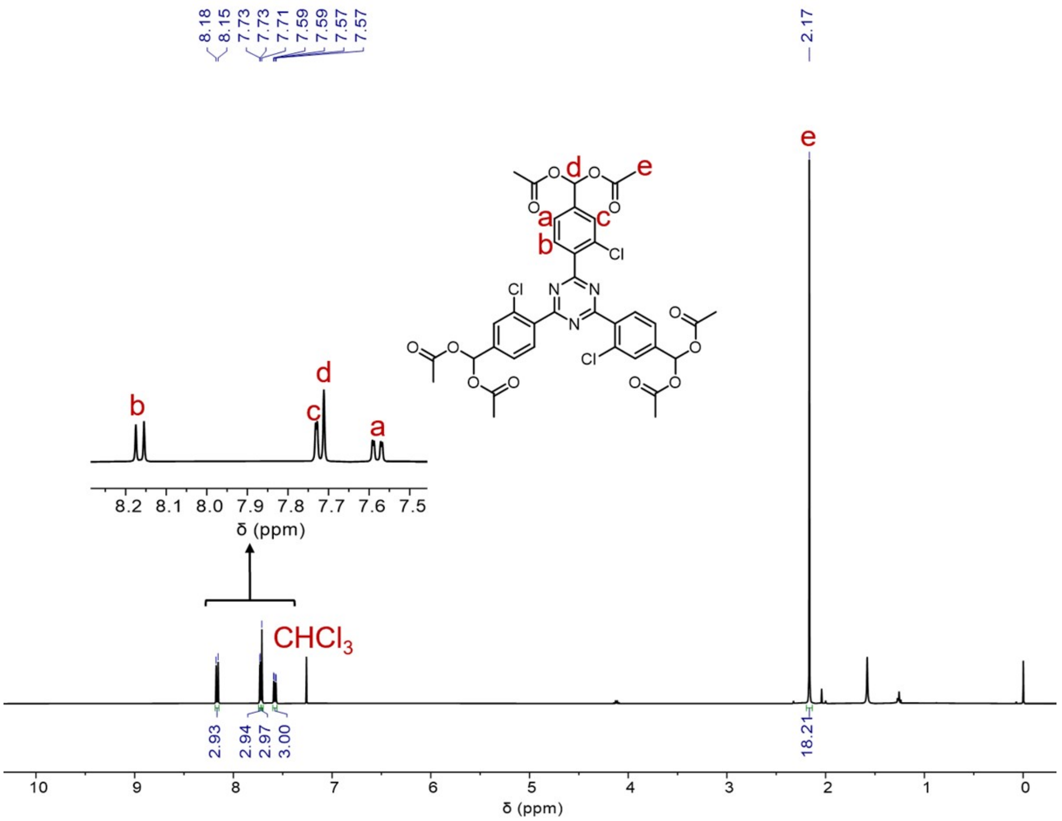

Figure S12. ^{1}H NMR spectrum of **S4** (400 MHz, $CDCl_3$, 298 K).

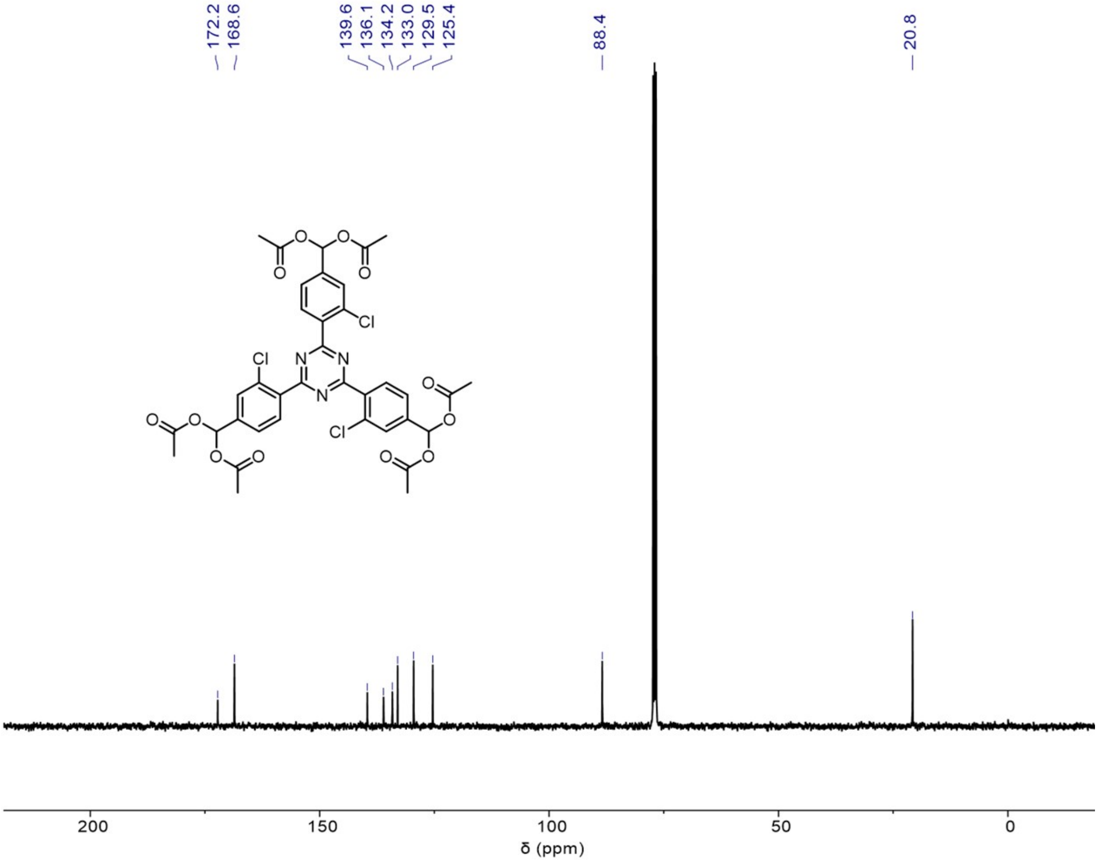

Figure S13. ^{13}C NMR spectrum of **S4** (100 MHz, $CDCl_3$, 298 K).

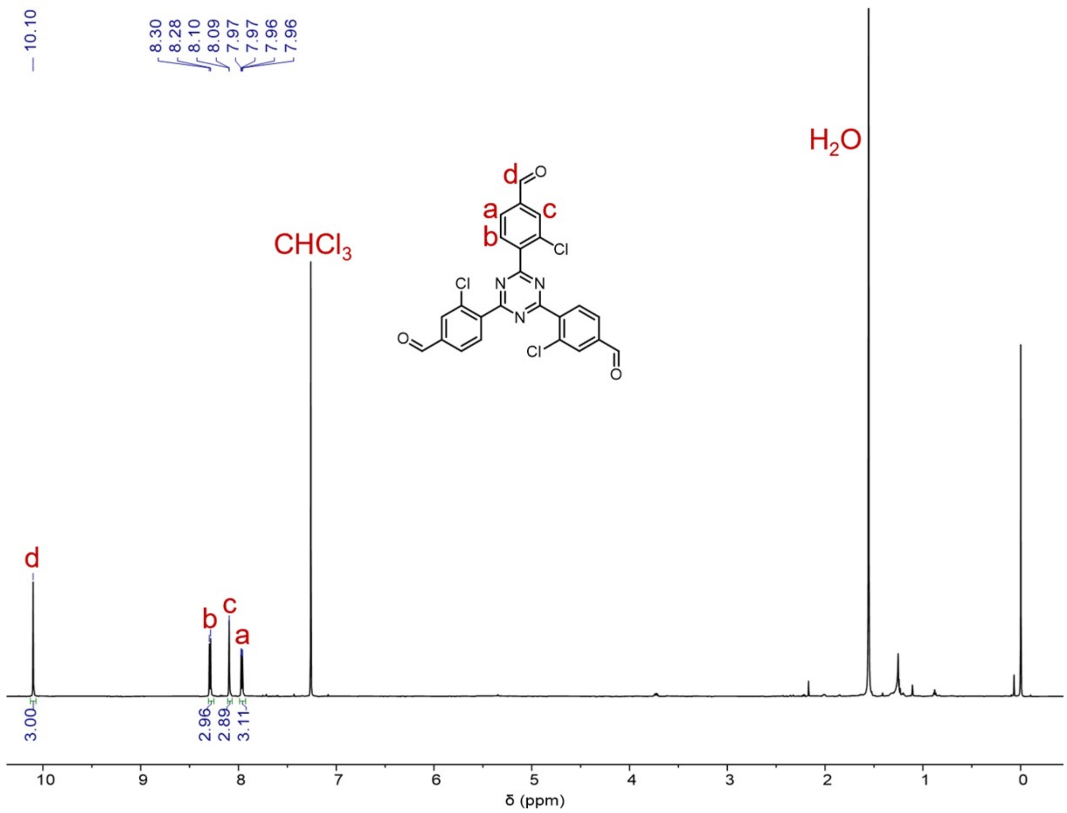

Figure S14. ^{1}H NMR spectrum of **1c** (600 MHz, $CDCl_3$, 298 K).

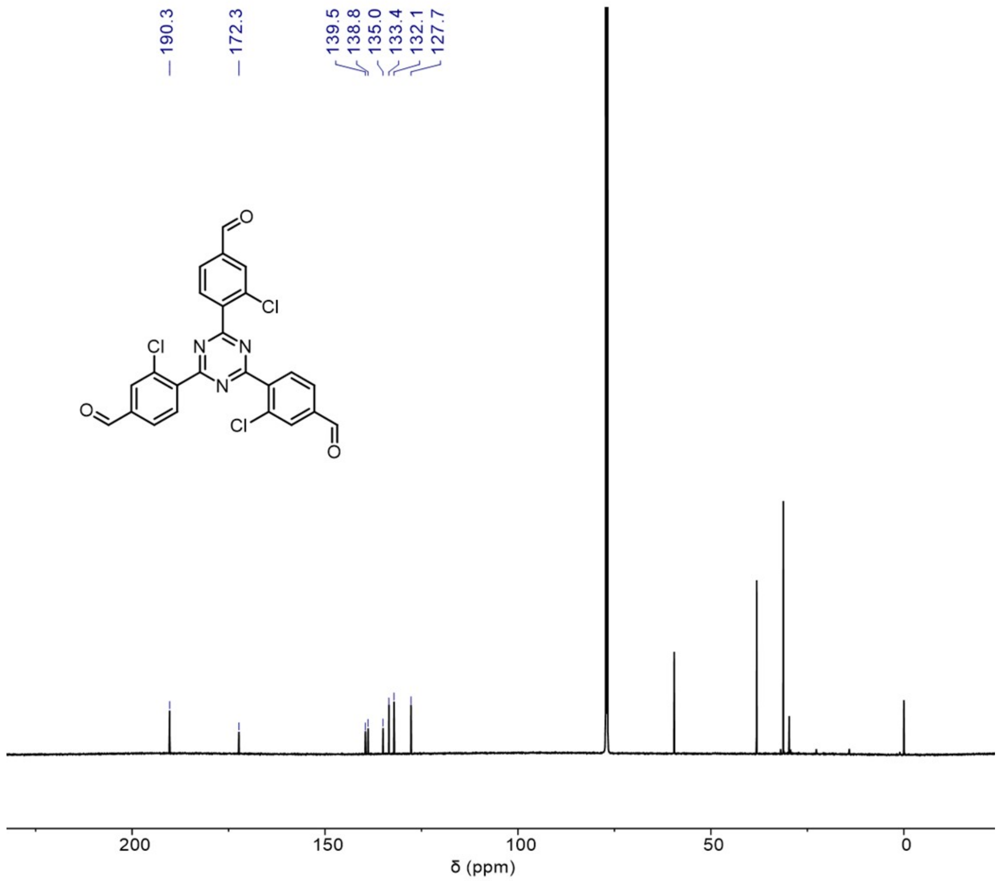

Figure S15. ^{13}C NMR spectrum of **1c** (150 MHz, $CDCl_3$, 298 K).

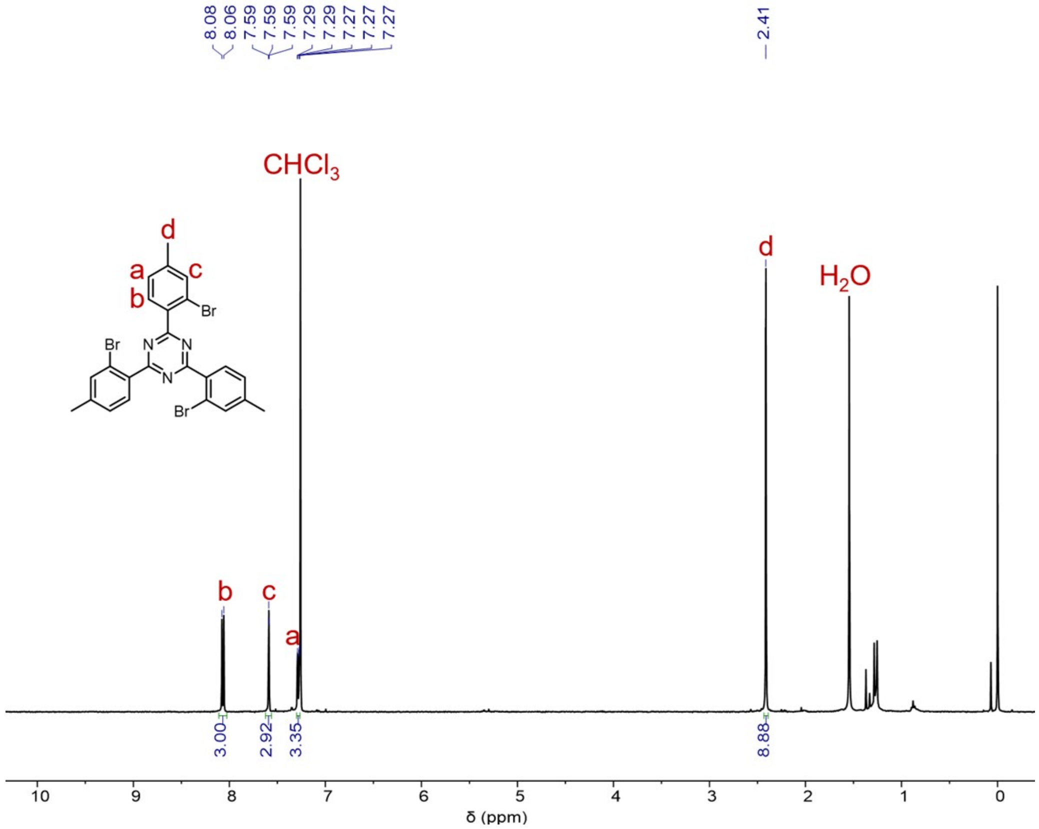

Figure S16. ^{1}H NMR spectrum of **S5** (400 MHz, $CDCl_3$, 298 K).

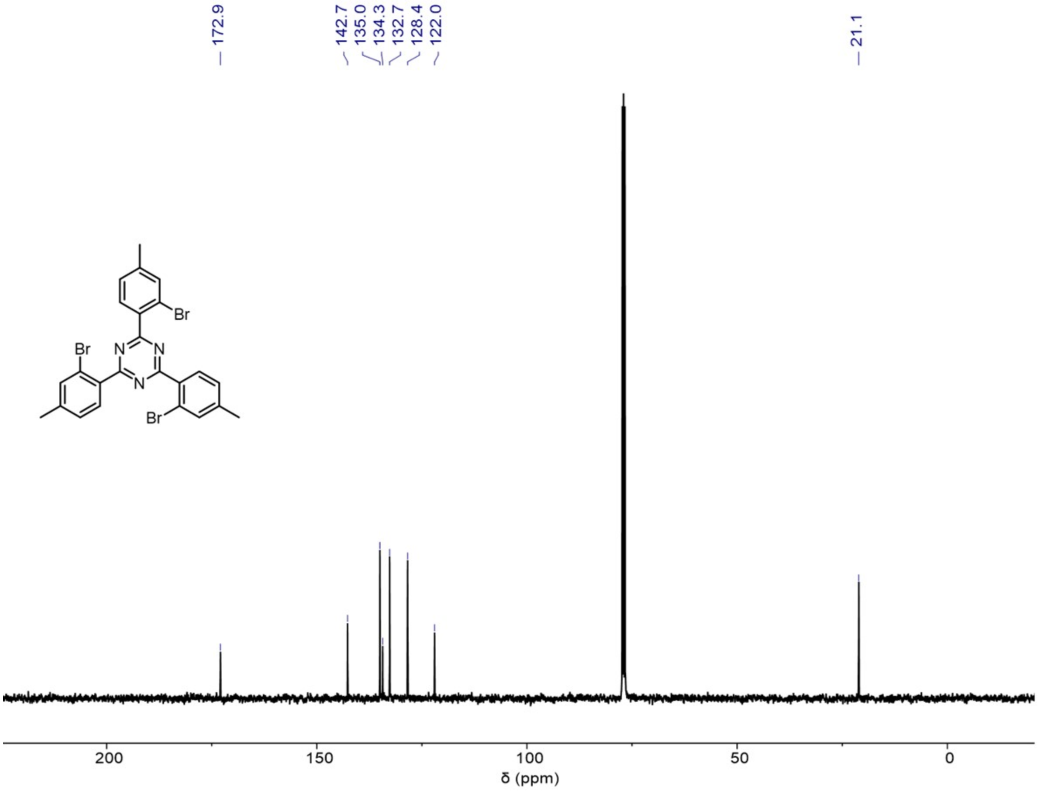

Figure S17. ^{13}C NMR spectrum of **S5** (100 MHz, $CDCl_3$, 298 K).

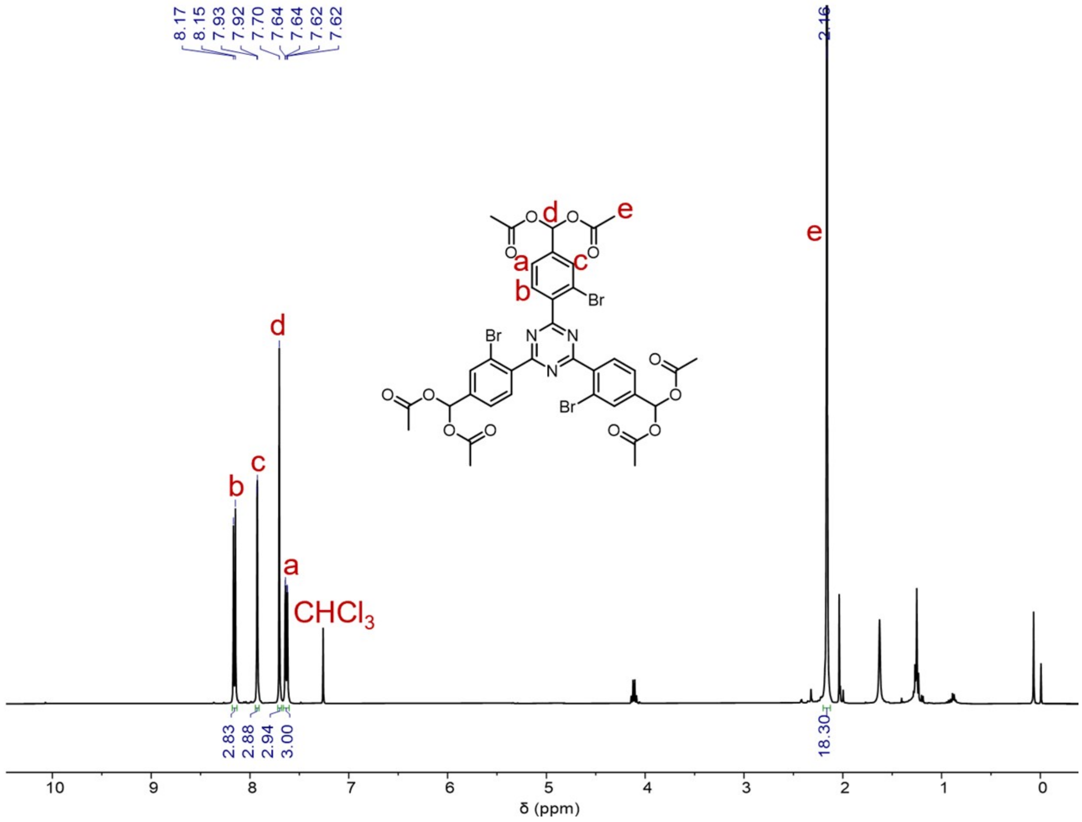

Figure S18. ^{1}H NMR spectrum of **S6** (400 MHz, $CDCl_3$, 298 K).

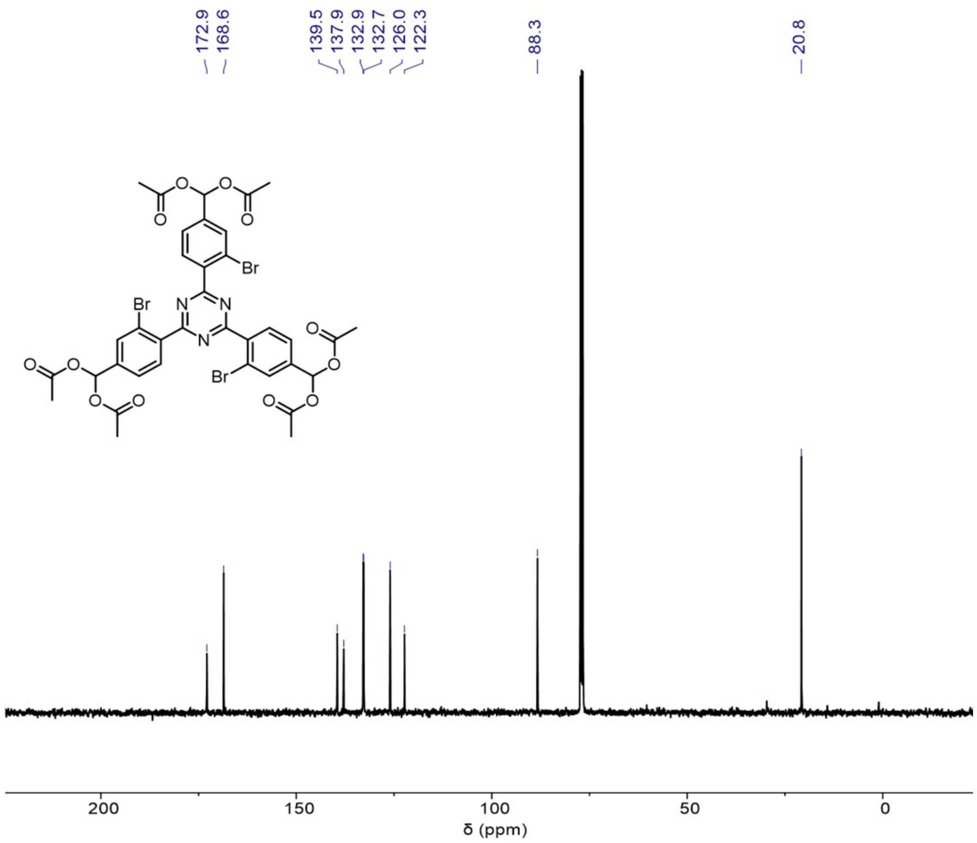

Figure S19. ^{13}C NMR spectrum of **S6** (100 MHz, $CDCl_3$, 298 K).

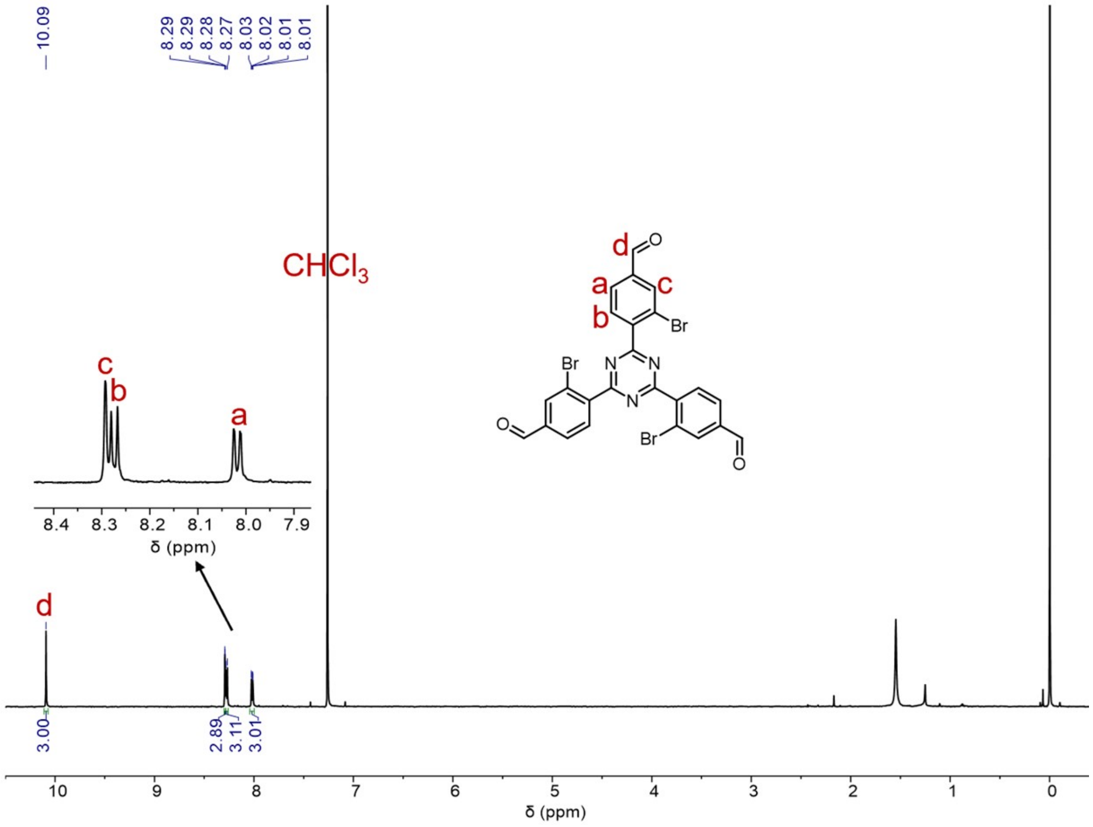

Figure S20. ^{1}H NMR spectrum of **1d** (600 MHz, CDCl3, 298 K).

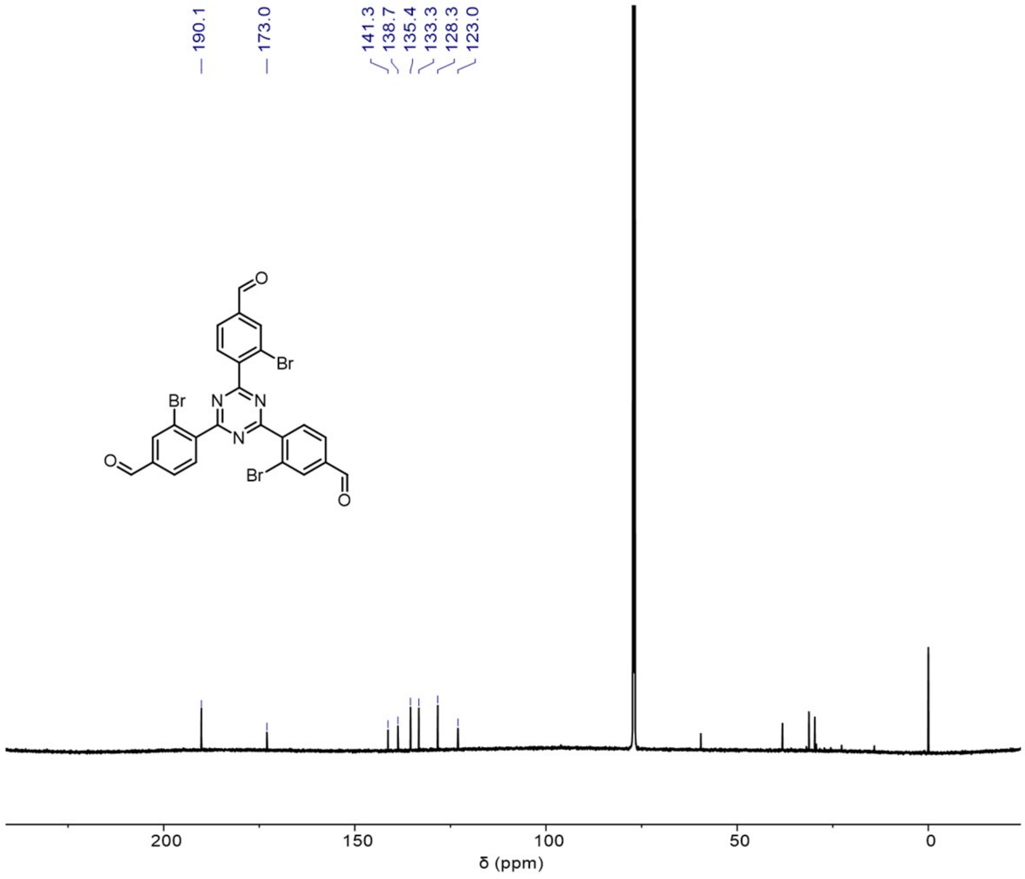

Figure S21. ^{13}C NMR spectrum of **1d** (150 MHz, $CDCl_3$, 298 K).

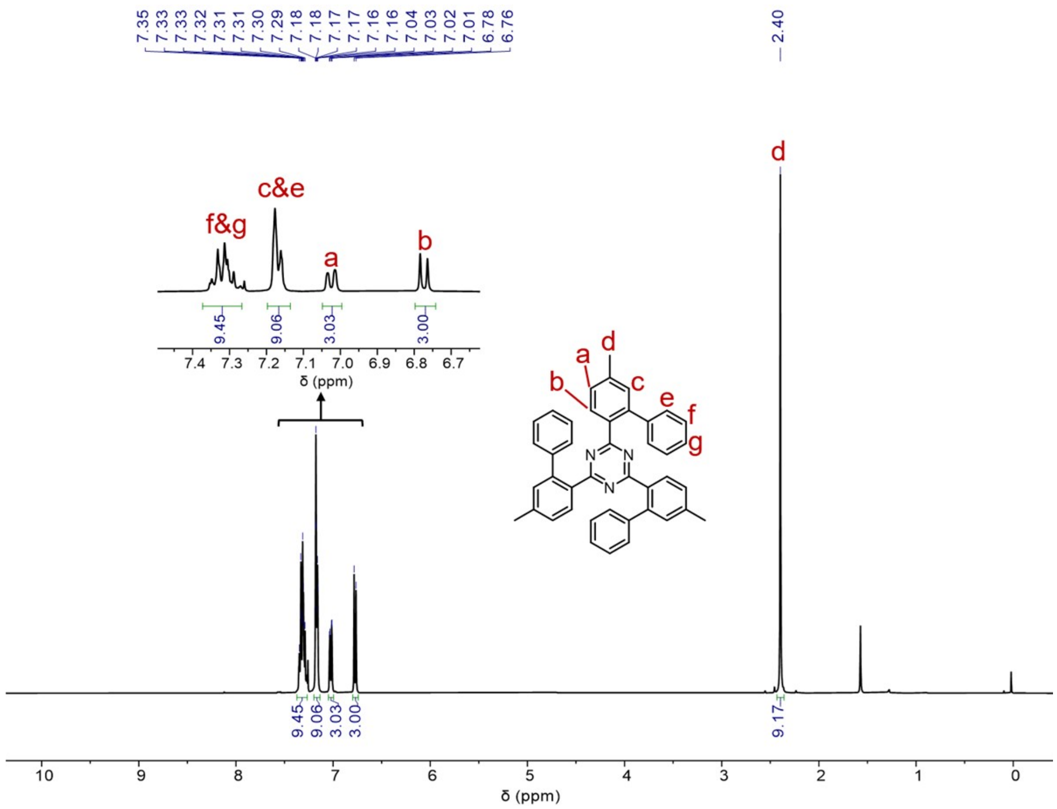

Figure S22. ^{1}H NMR spectrum of **S7** (400 MHz, $CDCl_3$, 298 K).

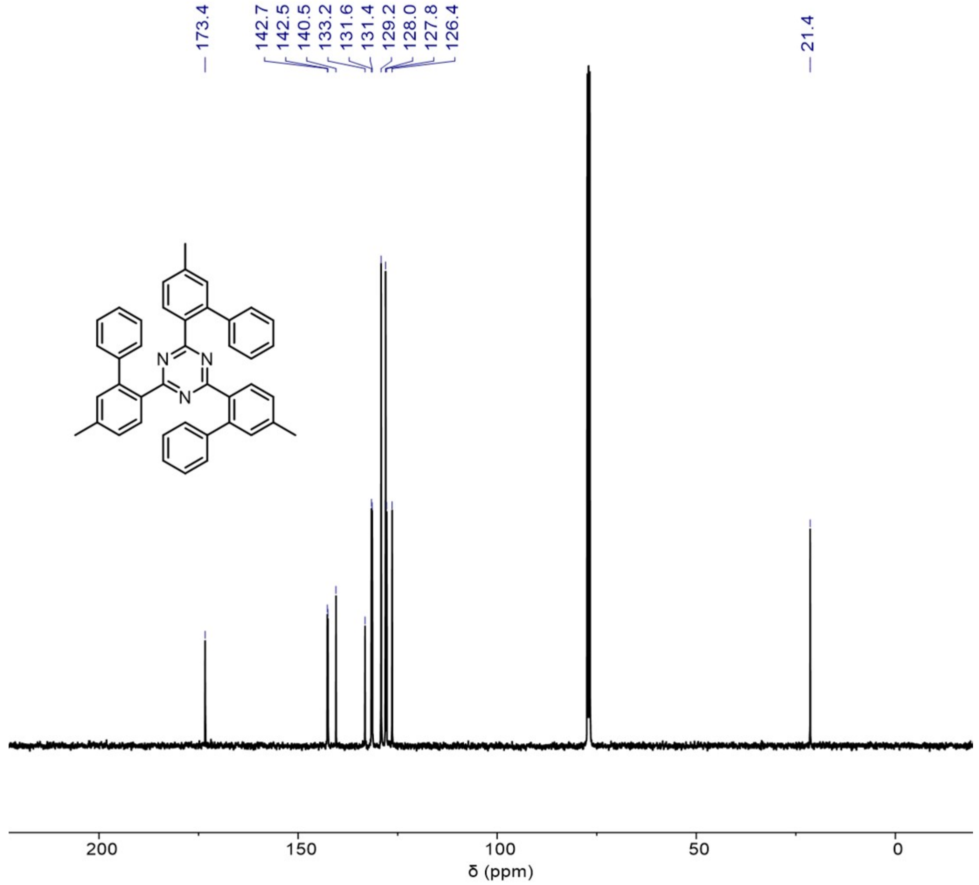

Figure S23. ^{13}C NMR spectrum of **S7** (100 MHz, $CDCl_3$, 298 K).

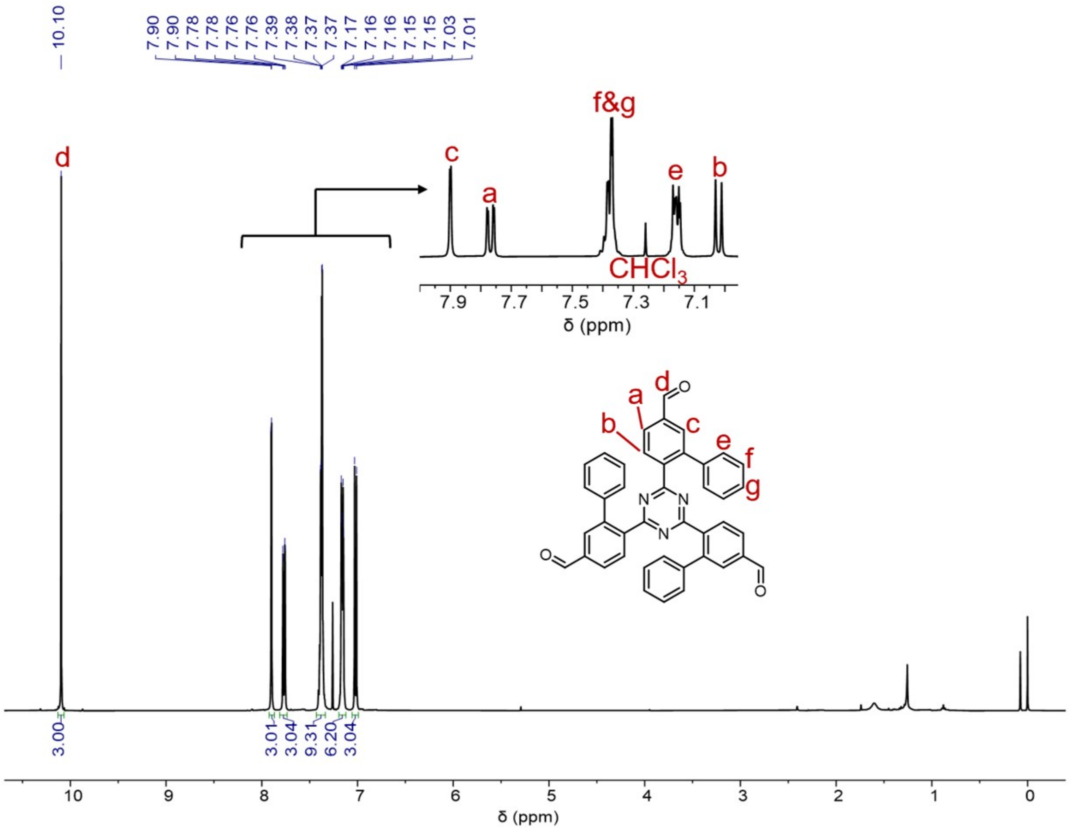

Figure S24. ^{1}H NMR spectrum of **1e** (400 MHz, CDCl3, 298 K).

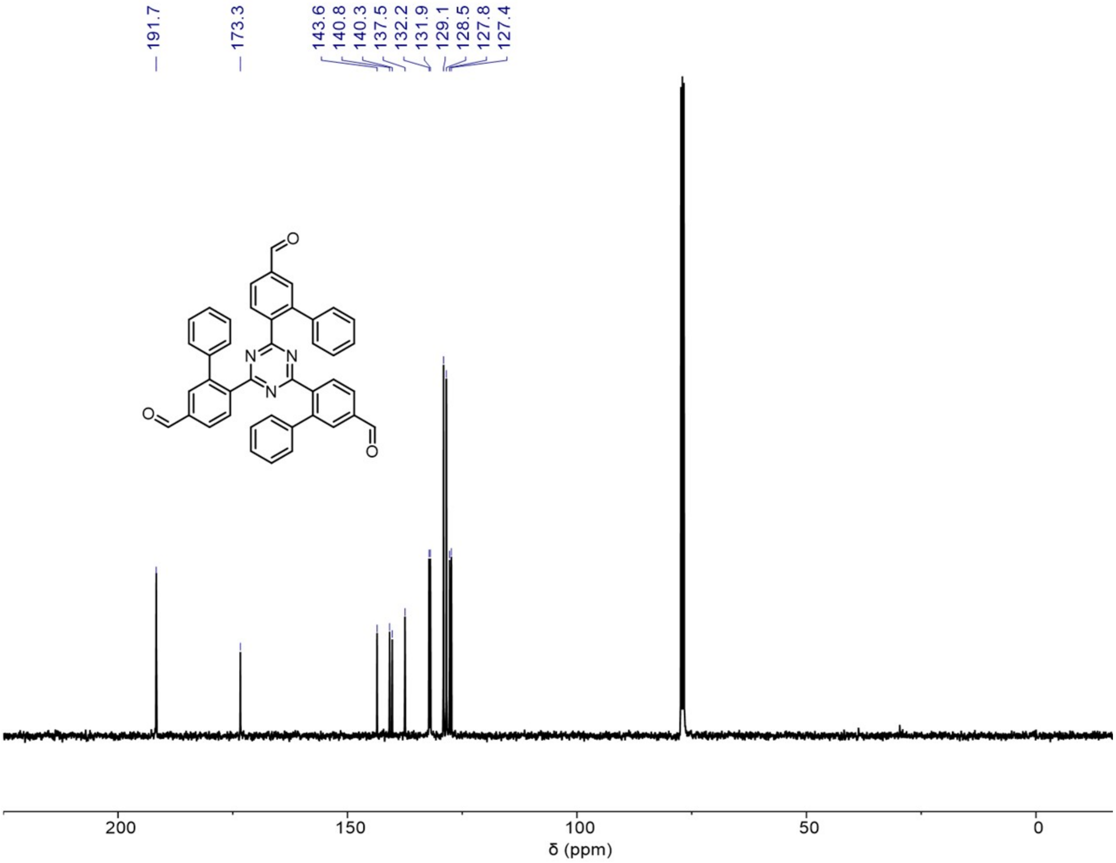

Figure S25. ^{13}C NMR spectrum of **1e** (100 MHz, $CDCl_3$, 298 K).

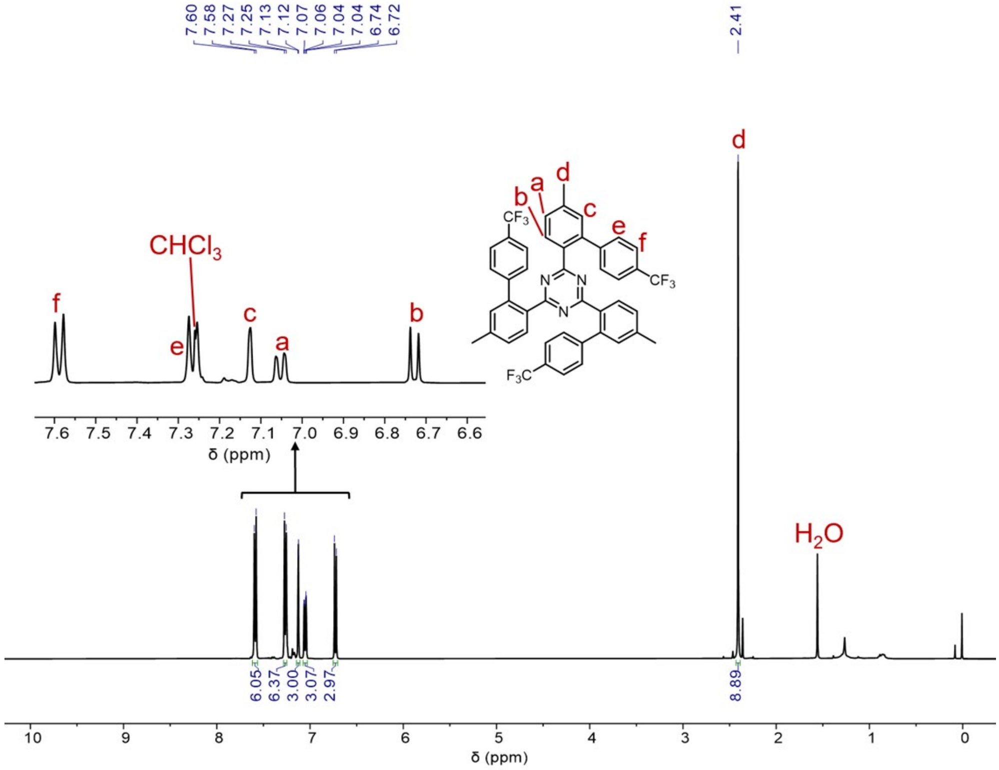

Figure S26. ^{1}H NMR spectrum of **S8** (400 MHz, $CDCl_3$, 298 K).

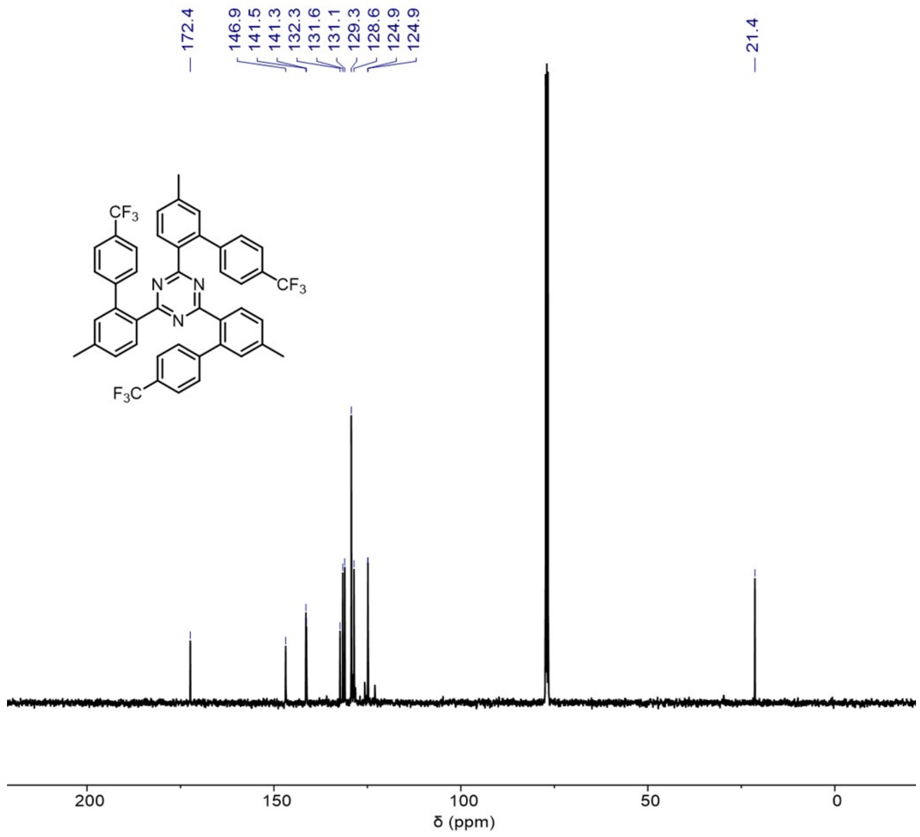

Figure S27. ^{13}C NMR spectrum of **S8** (100 MHz, $CDCl_3$, 298 K).

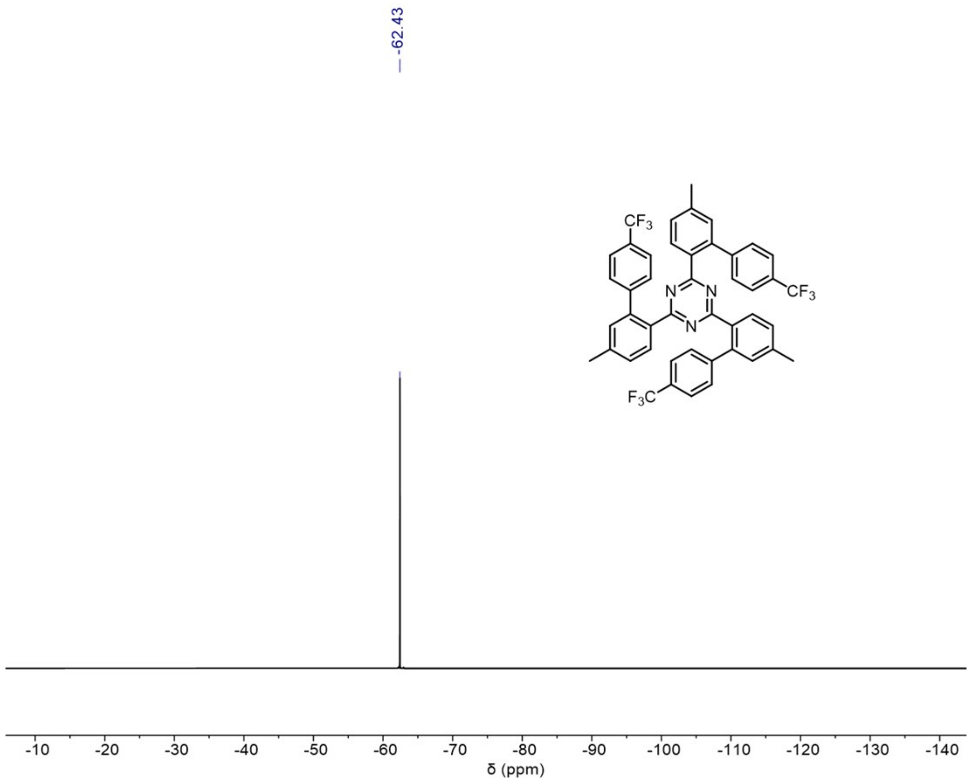

Figure S28. ^{19}F NMR spectrum of **S8** (376 MHz, $CDCl_3$, 298 K).

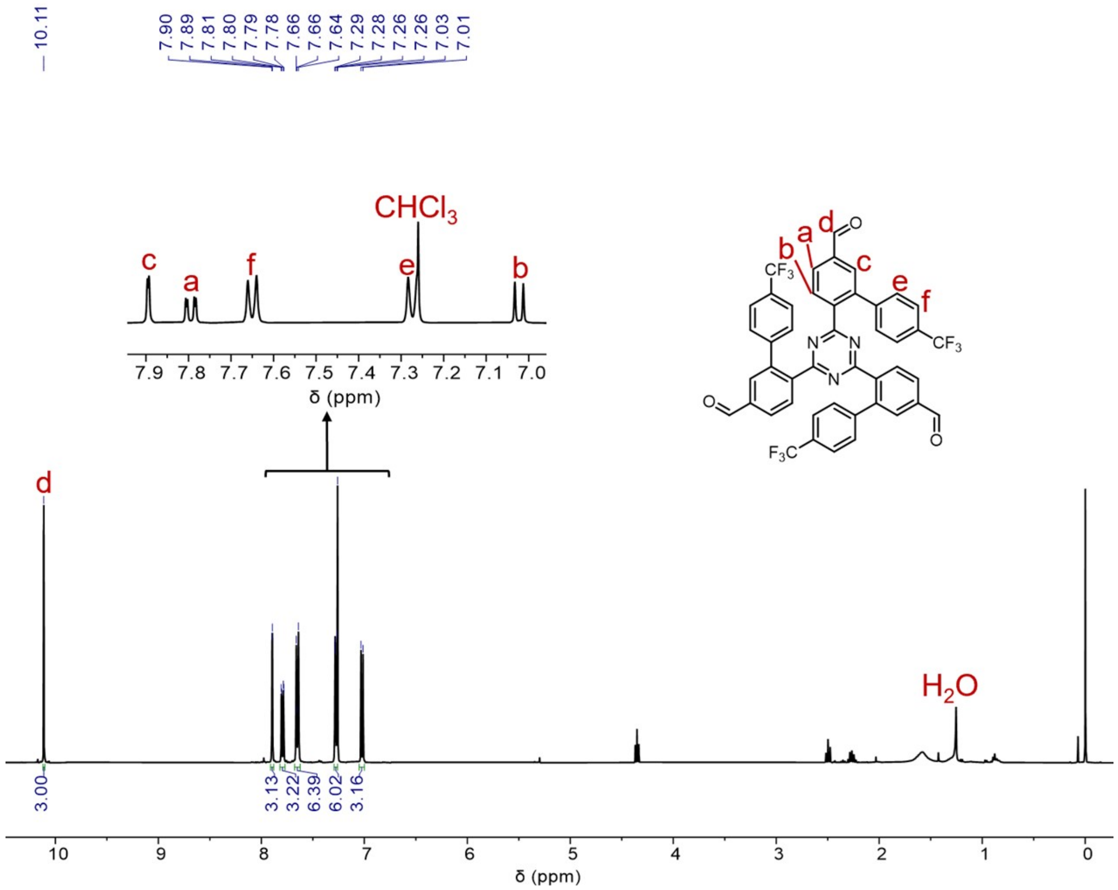

Figure S29. ^{1}H NMR spectrum of **1f** (400 MHz, $CDCl_3$, 298 K).

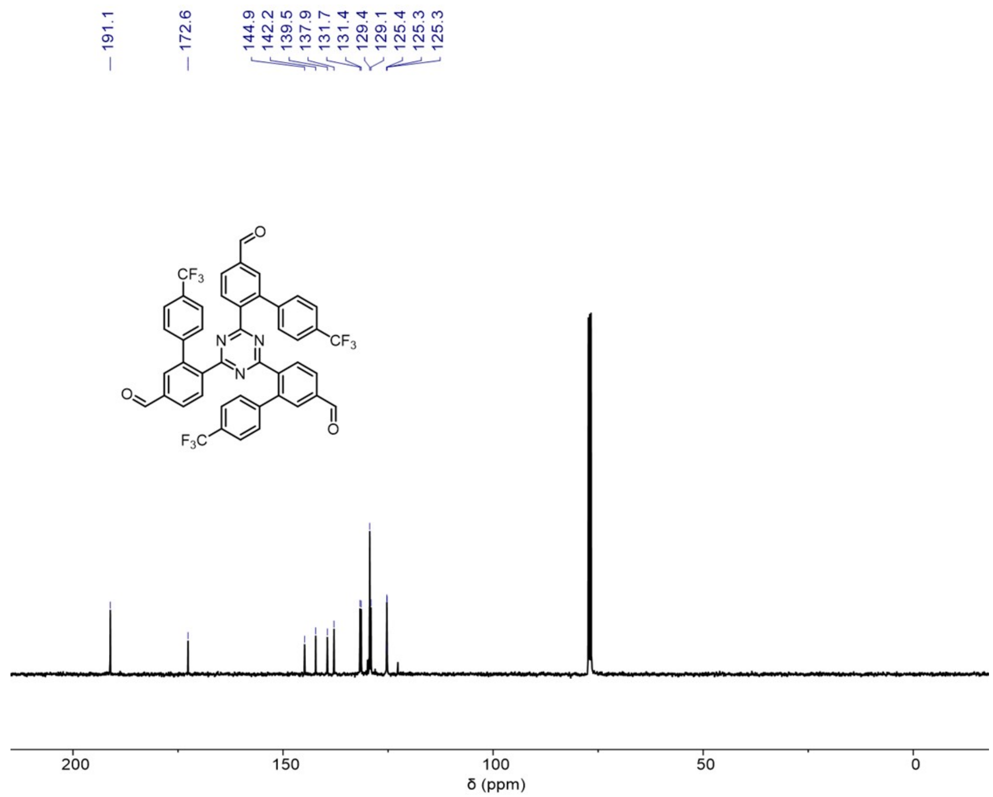

Figure S30. ^{13}C NMR spectrum of **1f** (100 MHz, $CDCl_3$, 298 K).

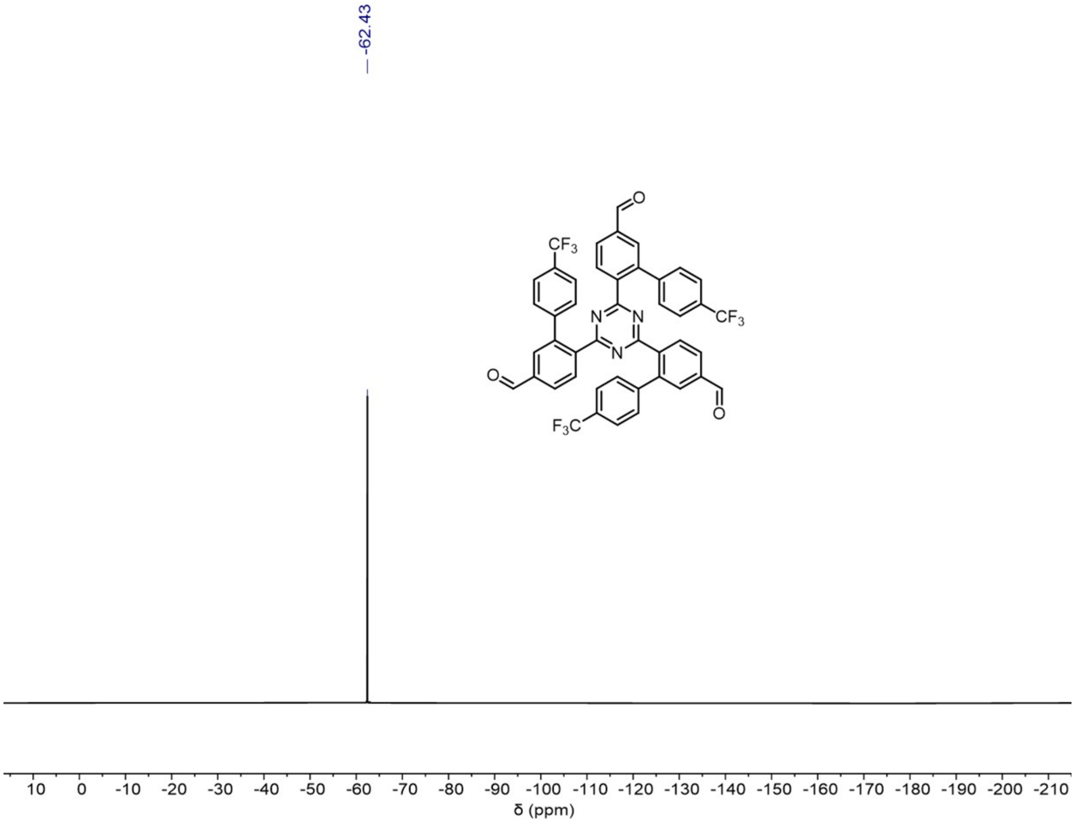

Figure S31. ^{19}F NMR spectrum of **1f** (376 MHz, $CDCl_3$, 298 K).

4. Synthesis and Characterization of the Cage Products

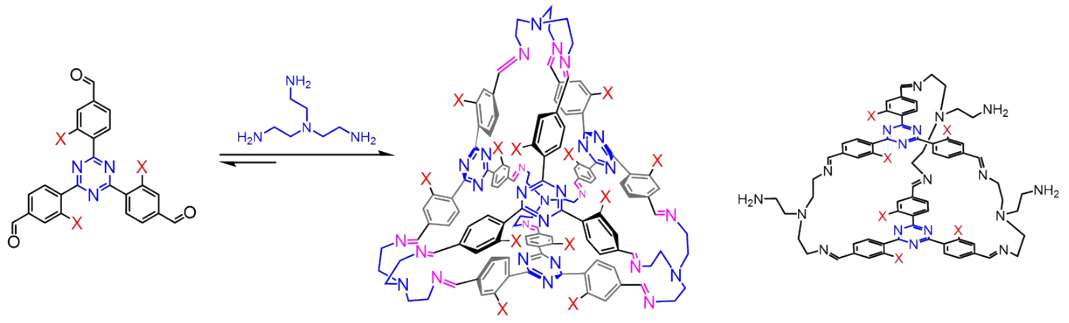

**1a** X = H	**2a** X = H	< 5%	**3a** X = H	47 %
**1b** X = F	**2b** X = F	29 %	**3b** X = F	8 %
**1c** X = Cl	**2c** X = Cl	82 %[a]	**3c** X = Cl	< 5 %
**1d** X = Br	**2d** X = Br	87 %[a]	**3d** X = Br	< 5 %
**1e** X = Ph	**2e** X = Ph	85 %[a]	**3e** X = Ph	< 5 %
**1f** X = 4-CF_3-Ph	**2f** X = 4-CF_3-Ph	45 %	**3f** X = 4-CF_3-Ph	< 5 %

Scheme S6. Condensation of **1a-1f** with TREN.

3.8 mg of **1b** (0.0085 mmol, 1 equiv.) were suspended in 0.6 mL of $CDCl_3$, after which tris(2-aminoethyl)amine (TREN) (1.25 mg, 0.0085 mmol, 1 equiv.) were added. The reaction was kept at 55 ºC for 12 h without stirring. The reaction progress was monitored by ^{1}H NMR spectroscopy, which indicates that the aldehyde-to-imine was completed and two sets of resonances were observed. 1,3,5-Trimethoxybenzene (1.68 mg, 0.01 mmol) was used as internal standard to calculate the yield of **2b** and **3b**.

The tetrahedral cage **2c** was prepared by condensing the **1c** (84.0 mg, 0.17 mmol, 1 equiv.) and tris(2-aminoethyl)amine (TREN) (25.0 mg, 0.17 mmol, 1 equiv.) in $CHCl_3$ (30 mL). The mixture was stirred at 55 ºC for 12 h to obtain the corresponding cage as major product. A small amount of insoluble polymeric byproducts were removed by filtration. The filtrate was collected and condensed under reduced pressure. Petroleum ether was added to precipitate the desired product **2c** as a yellow powder (82.0 mg, 82 %).

Similar procedure was used to prepared the tetrahedral cages **2d** and **2e**, whose yields were measured to be 87 % and 85 %, respectively.

2f was prepared by condensing **1f** (2.0 mg, 0.002 mmol, 1 equiv.) and tris(2-aminoethyl)amine (TREN) (0.3 mg, 0.002 mmol, 1 equiv.) in $CDCl_3$ (0.6 mL). The mixture was heated at 55 ºC for 12 h, which produced **2f** as the only observable product without further purification as indicated by the 1H NMR spectrum. The yield of **2f** was calculated by using 1,3,5-trimethoxybenzene as an internal standard in the ^{1}H NMR spectrum.

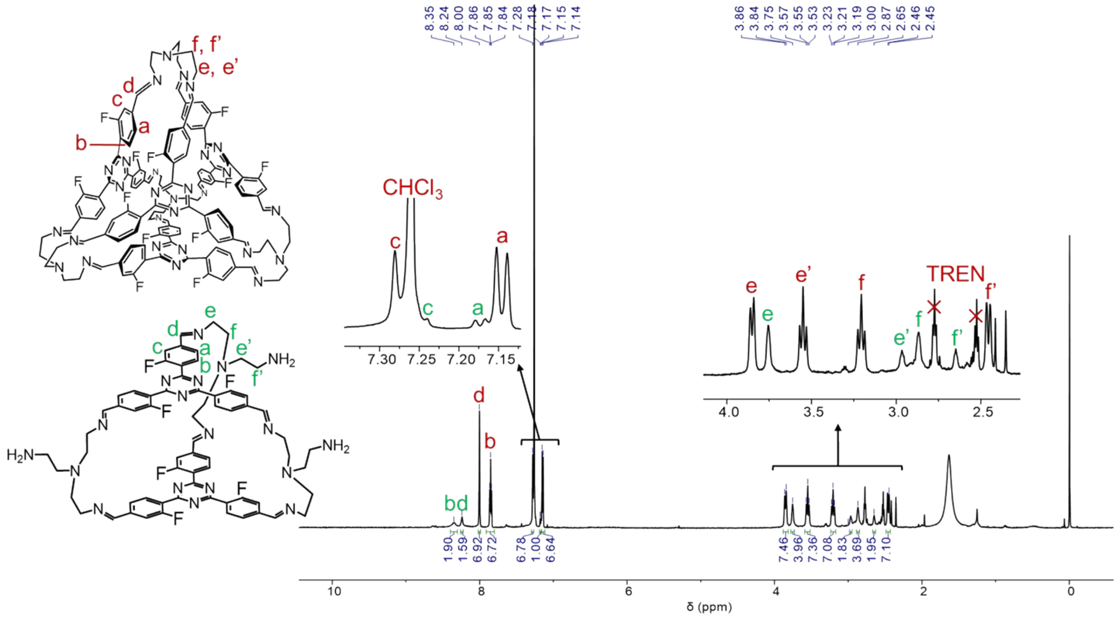

Figure S32. ^{1}H NMR spectra (600 MHz, $CDCl_3$, 298 K) of **2b** and **3b.** The molar ration of **2b** to **3b** was 2:1 by integration of ^{1}H NMR signals.

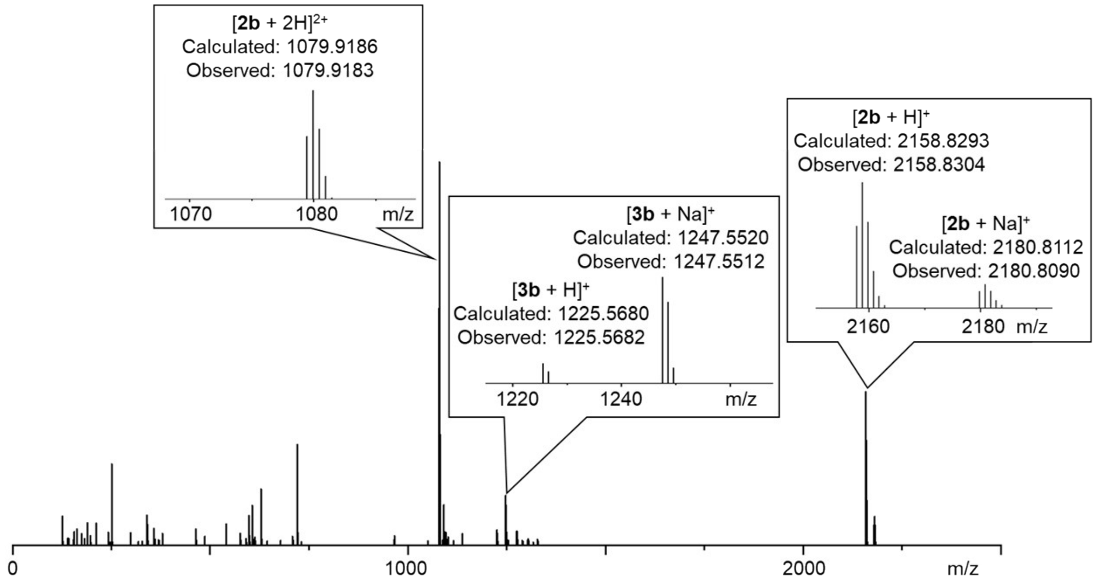

Figure S33. ESI-HRMS of **2b** and **3b**. Peaks corresponding to molecular ions of **2b** and **3b** bearing one or two positive charges by taking either or two protons, or Na^+ are observed.

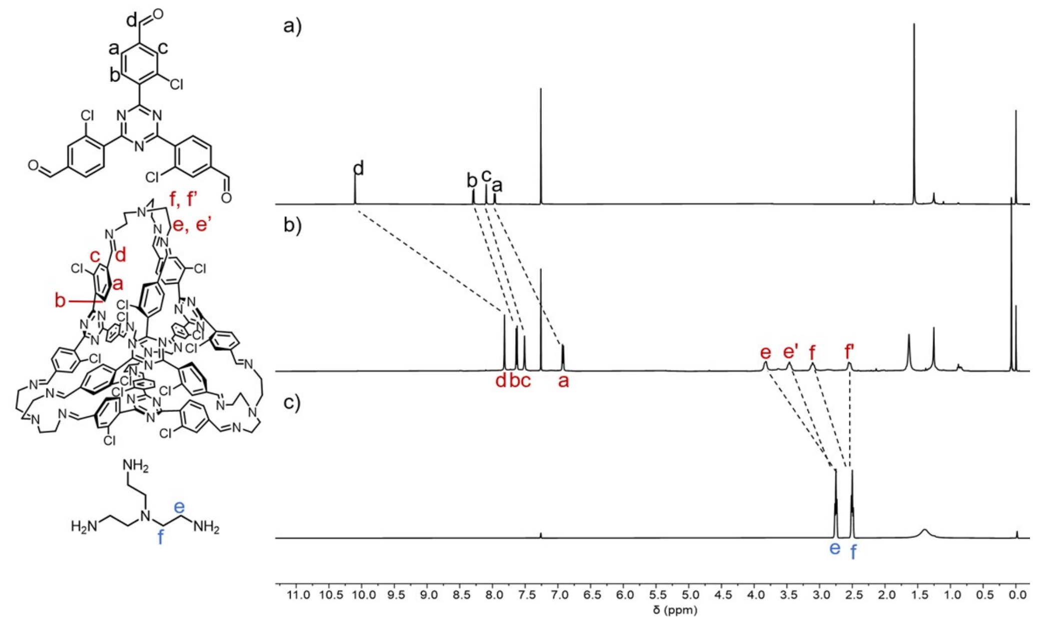

Figure S34. ^{1}H NMR spectra (500 MHz, $CDCl_3$, 298 K) of a) **1c**, b) **2c** and c) TREN.

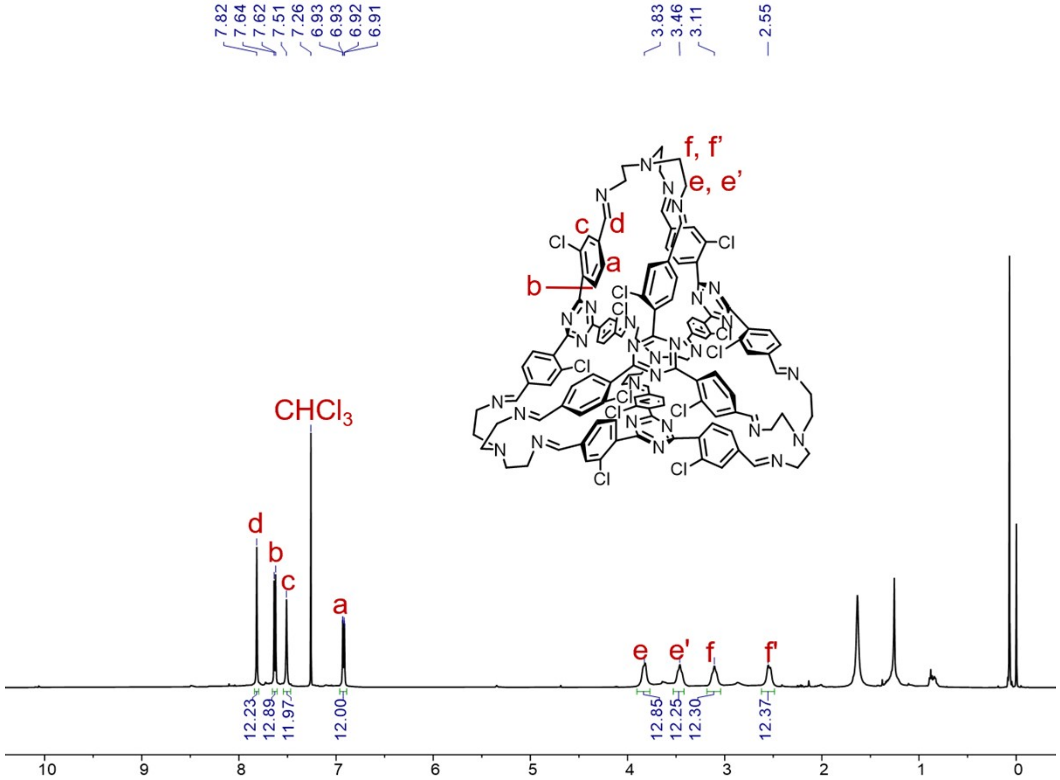

Figure S35. ^{1}H NMR spectra (500 MHz, $CDCl_3$, 298 K) of **2c**.

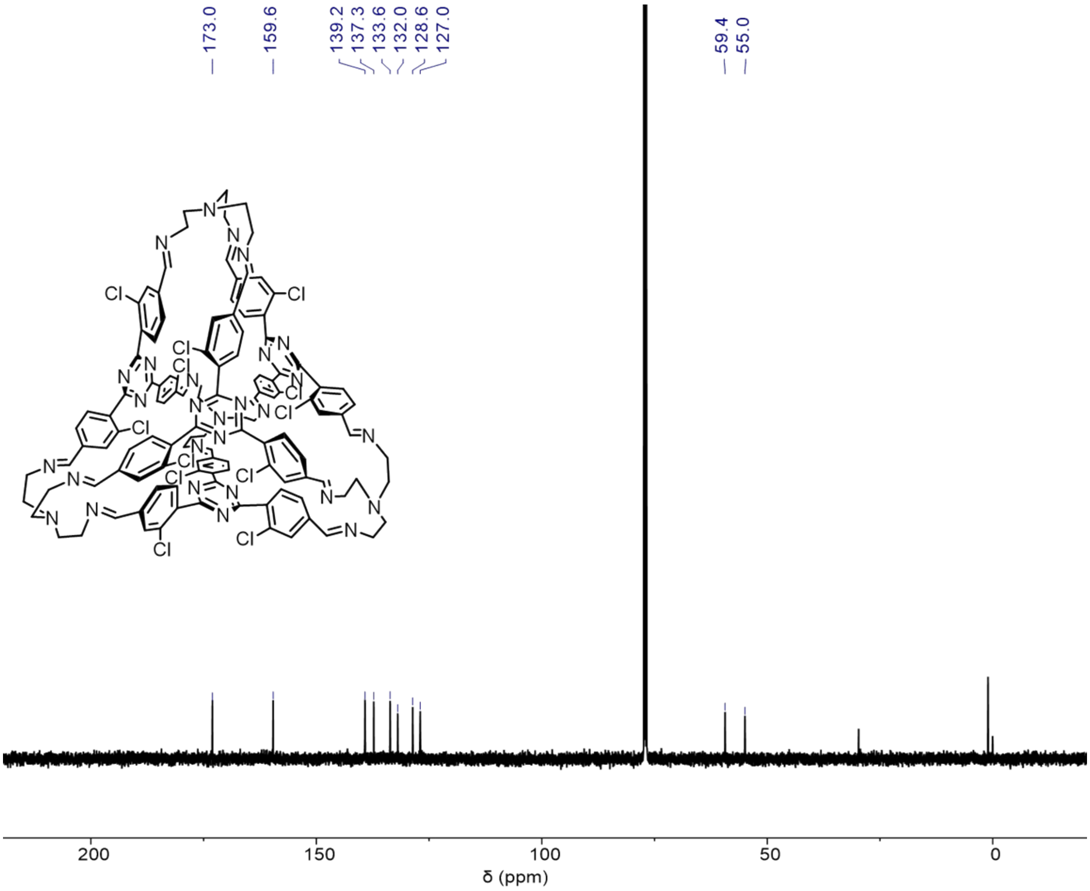

Figure S36. ^{13}C NMR spectra (125 MHz, $CDCl_3$, 298 K) of **2c**.

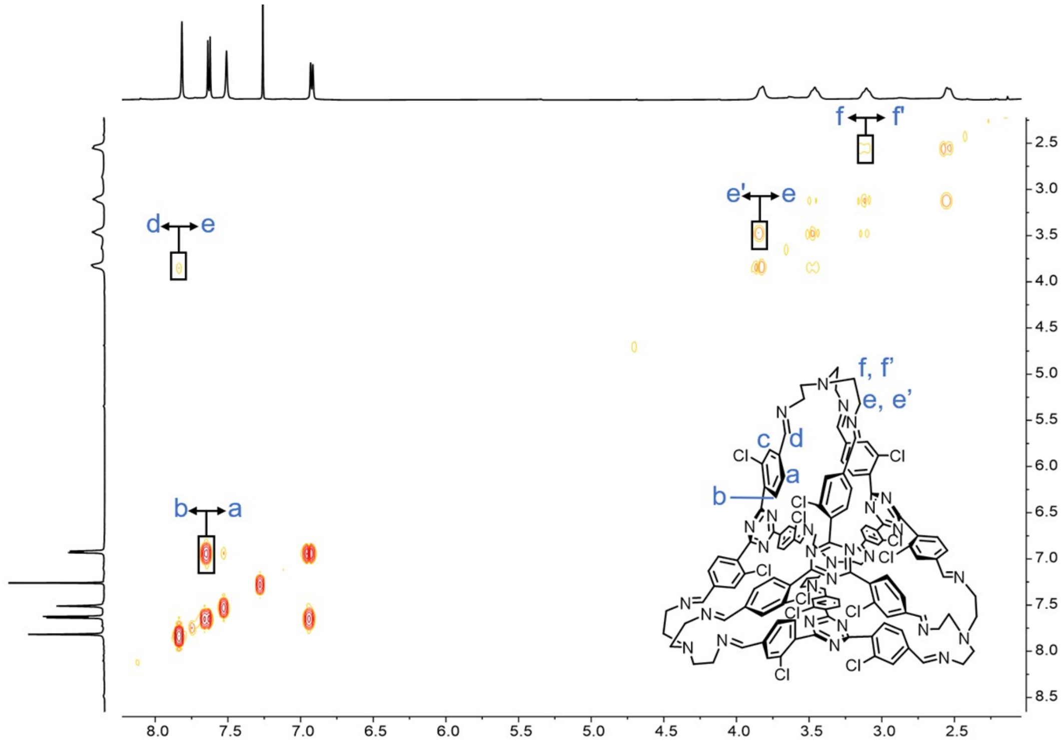

Figure S37. ^{1}H–^{1}H COSY spectrum of **2c** (500 MHz, $CDCl_3$, 298 K). The key correlation peaks are labeled in the spectrum.

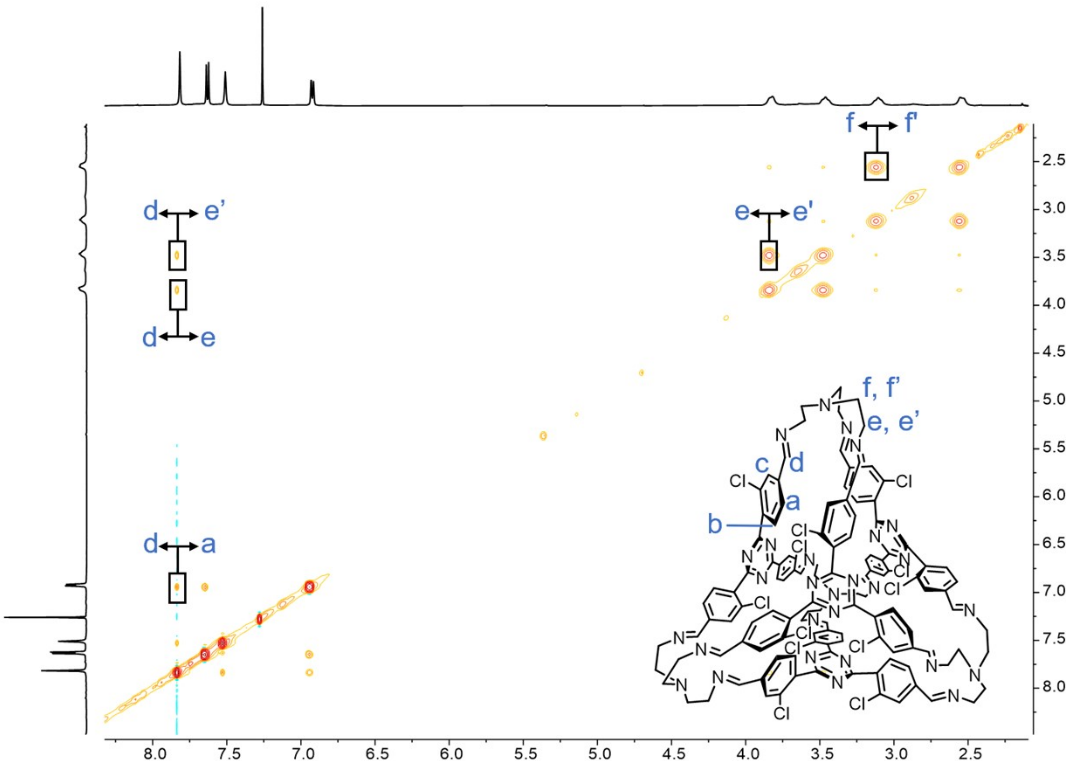

Figure S38. $^{1}H–^{1}H$ NOESY spectrum of cage **2c** (500 MHz, $CDCl_3$, 298K). Through-space proton couplings between some protons are labeled in the spectrum.

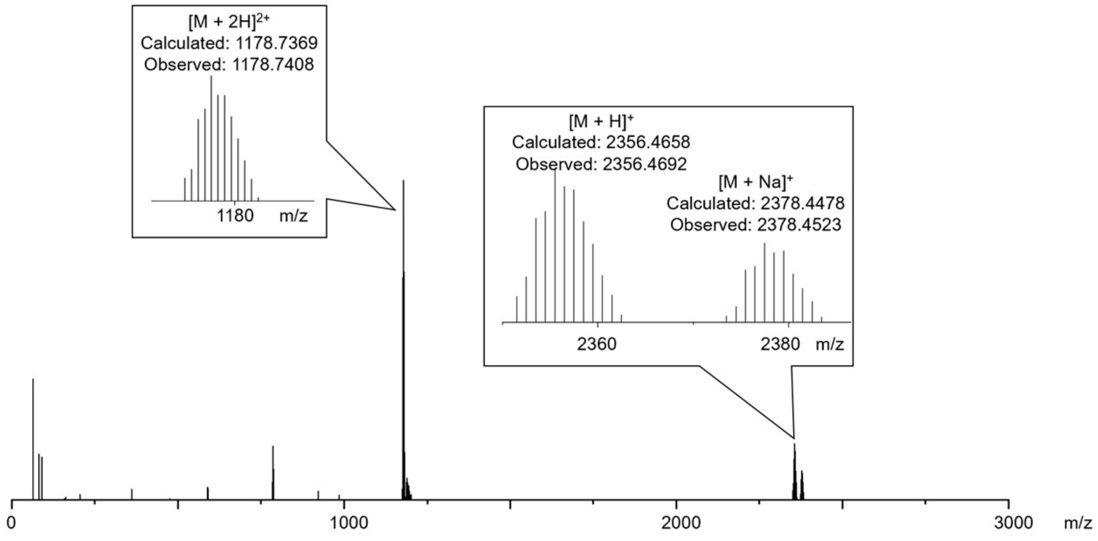

Figure S39. ESI-HRMS of **2c**. Peaks corresponding to molecular ions bearing one or two positive charges by taking protons or Na^{+} are observed.

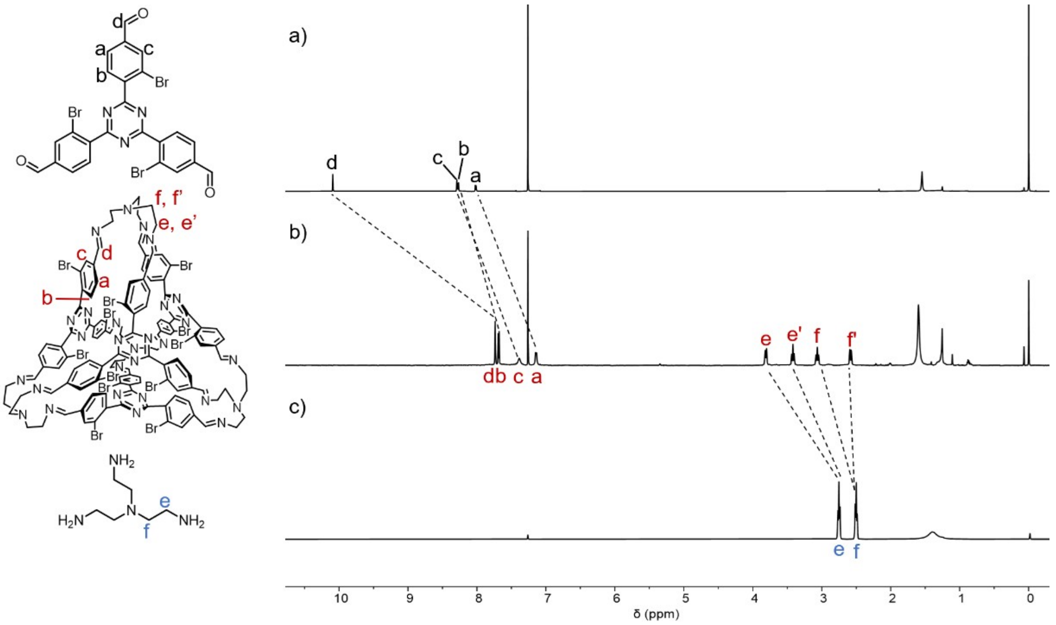

Figure S40. ^{1}H NMR spectra (600 MHz, $CDCl_3$, 298 K) of a) **1d**, b) **2d** and c) TREN.

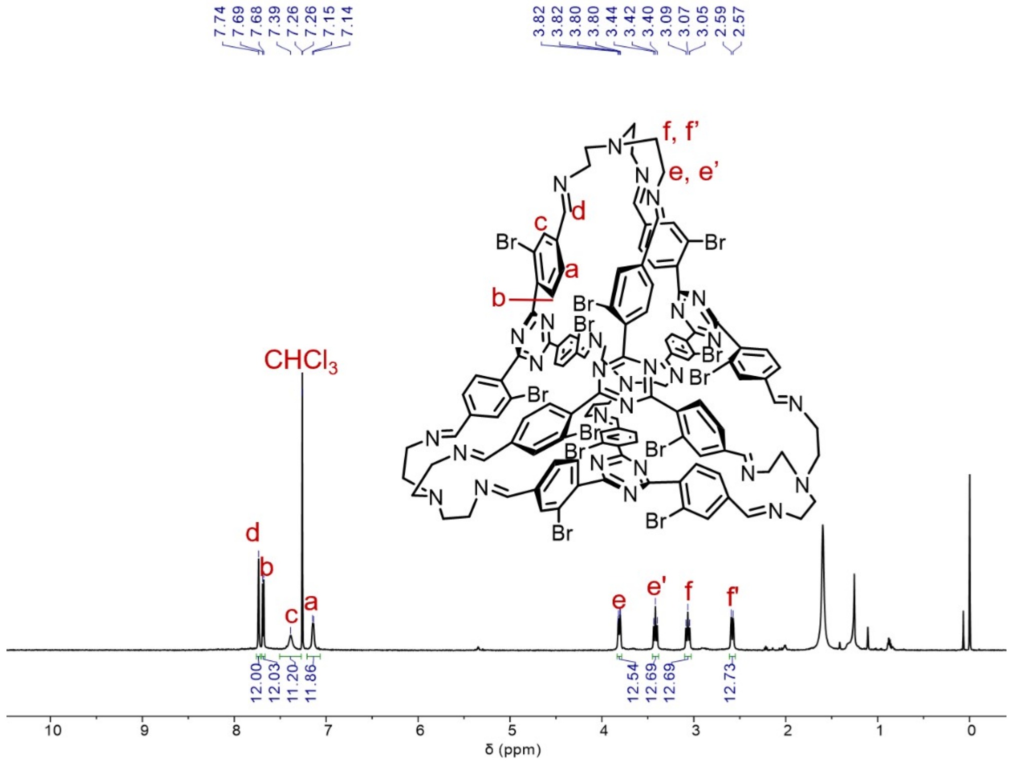

Figure S41. ^{1}H NMR spectra (600 MHz, $CDCl_3$, 298 K) of **2d**.

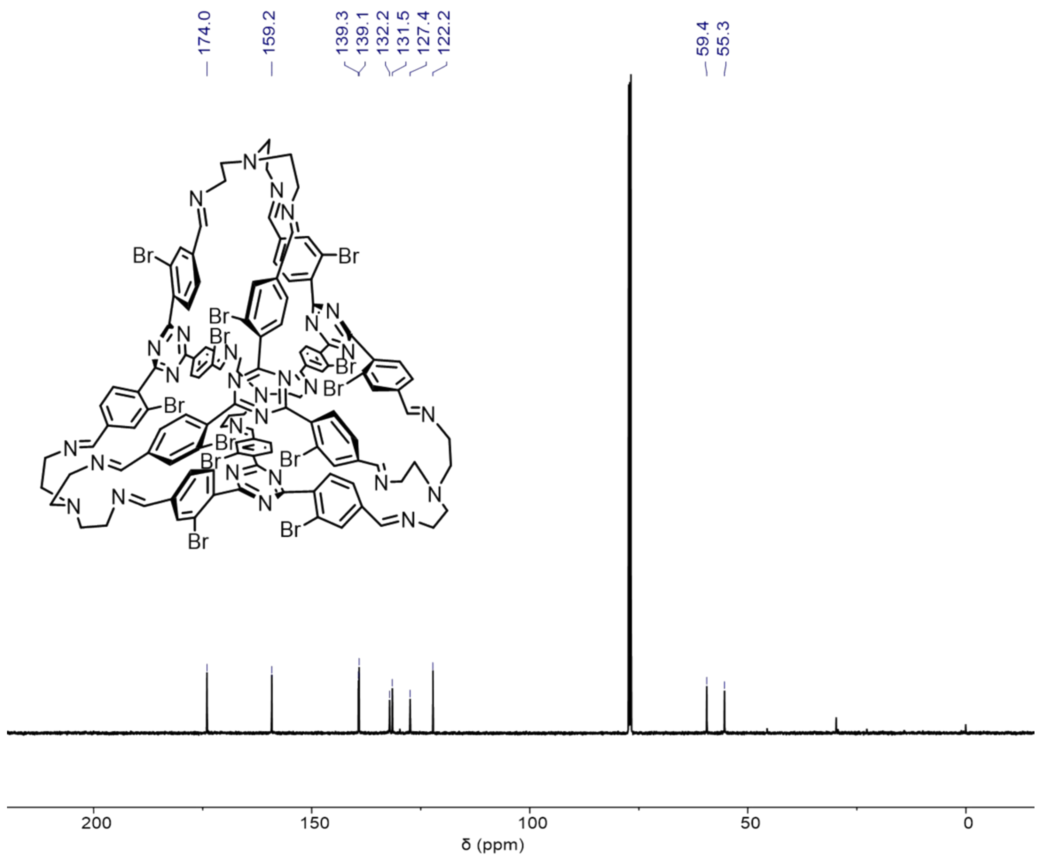

Figure S42. ^{13}C NMR spectra (125 MHz, $CDCl_3$, 298 K) of **2d**.

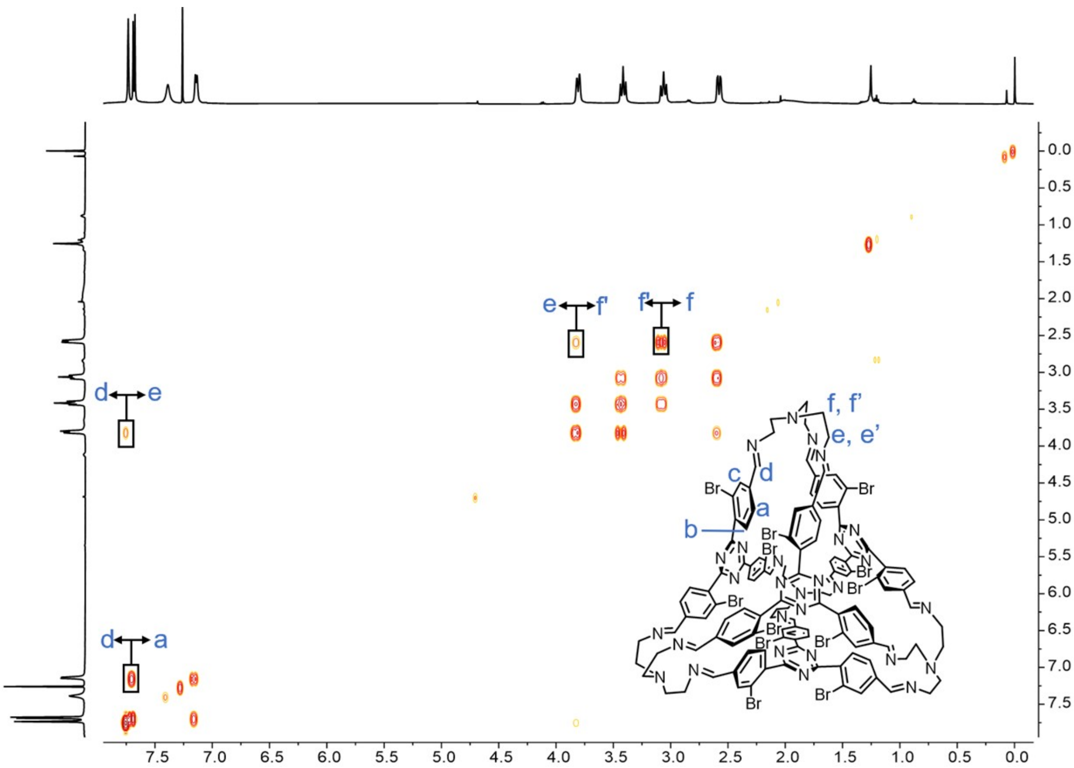

Figure S43. ^1H–^1H COSY spectrum of **2d** (500 MHz, $CDCl_3$, 298 K). The key correlation peaks are labeled in the spectrum.

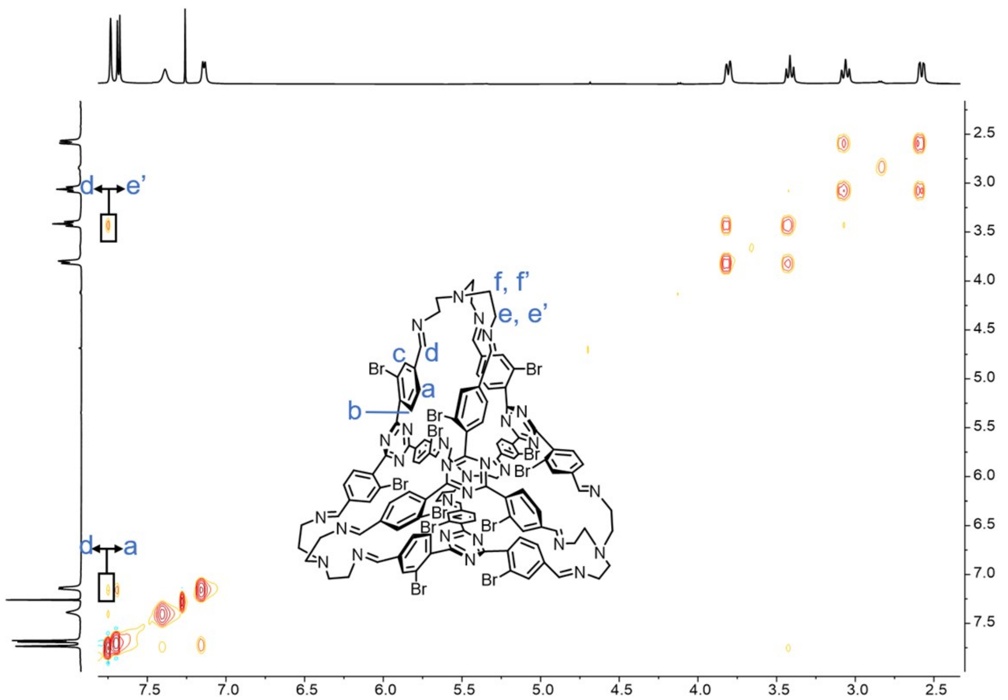

Figure S44. ^{1}H–^{1}H NOESY spectrum of cage **2d** (500 MHz, $CDCl_3$, 298K). Through-space proton couplings between some protons are labeled in the spectrum.

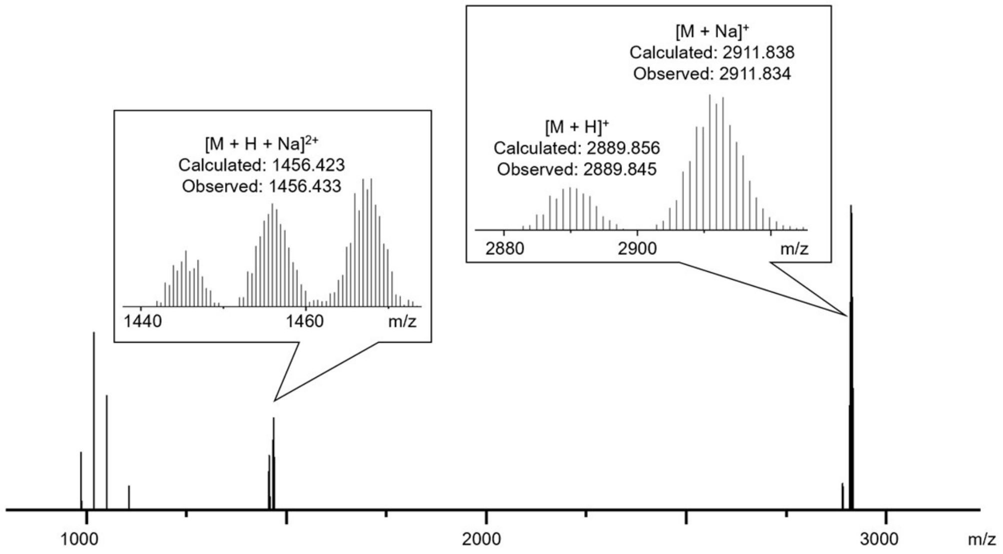

Figure S45. ESI-HRMS of **2d**. Peaks corresponding to molecular ions bearing one or two positive charges by taking proton or sodium cations are observed.

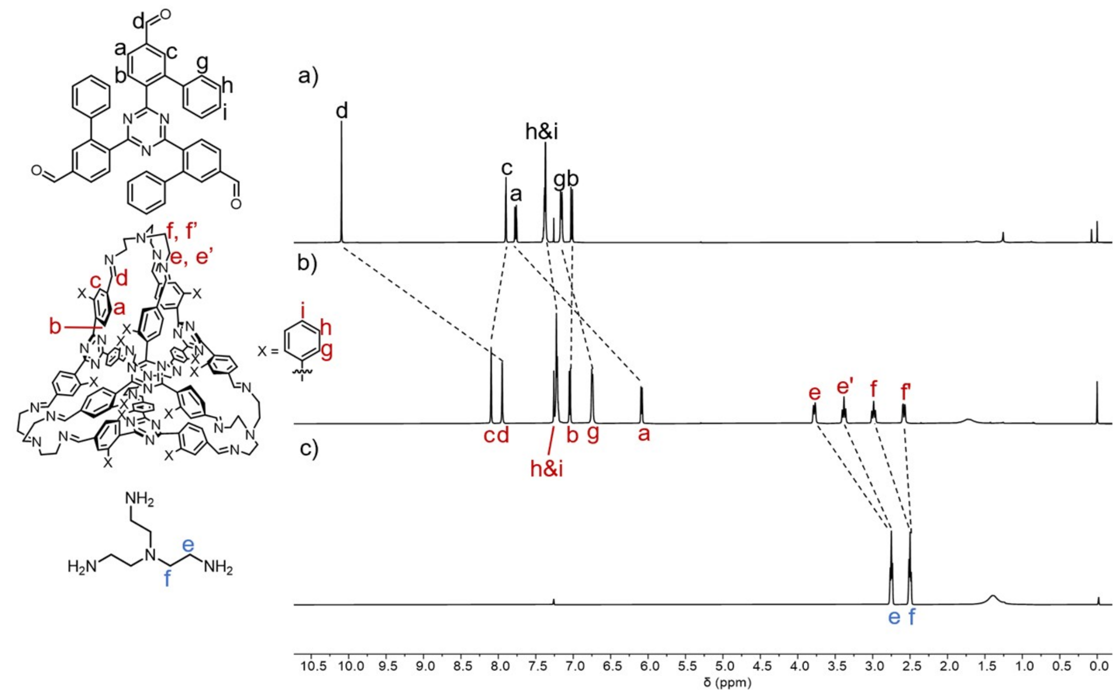

Figure S46. ^{1}H NMR spectra (500 MHz, $CDCl_3$, 298 K) of a) **1e**, b) **2e** and c) TREN.

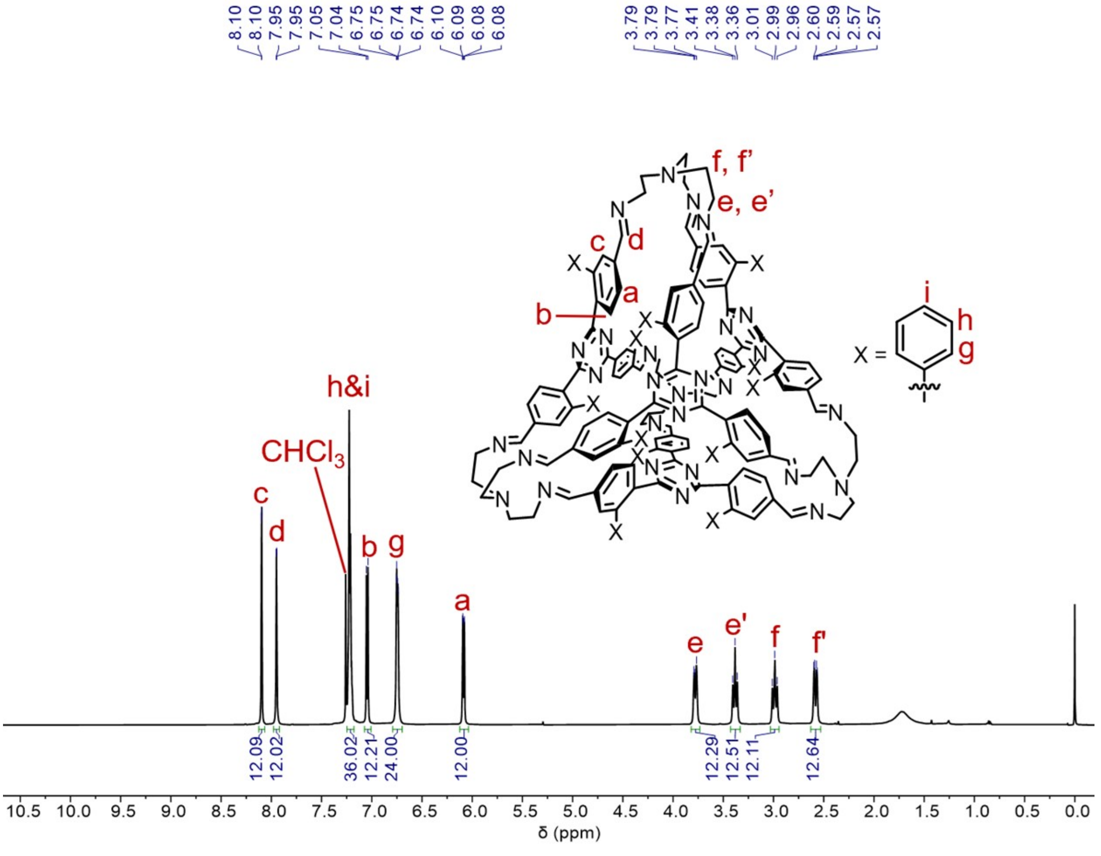

Figure S47. ^{1}H NMR spectra (500 MHz, $CDCl_3$, 298 K) of **2e**.

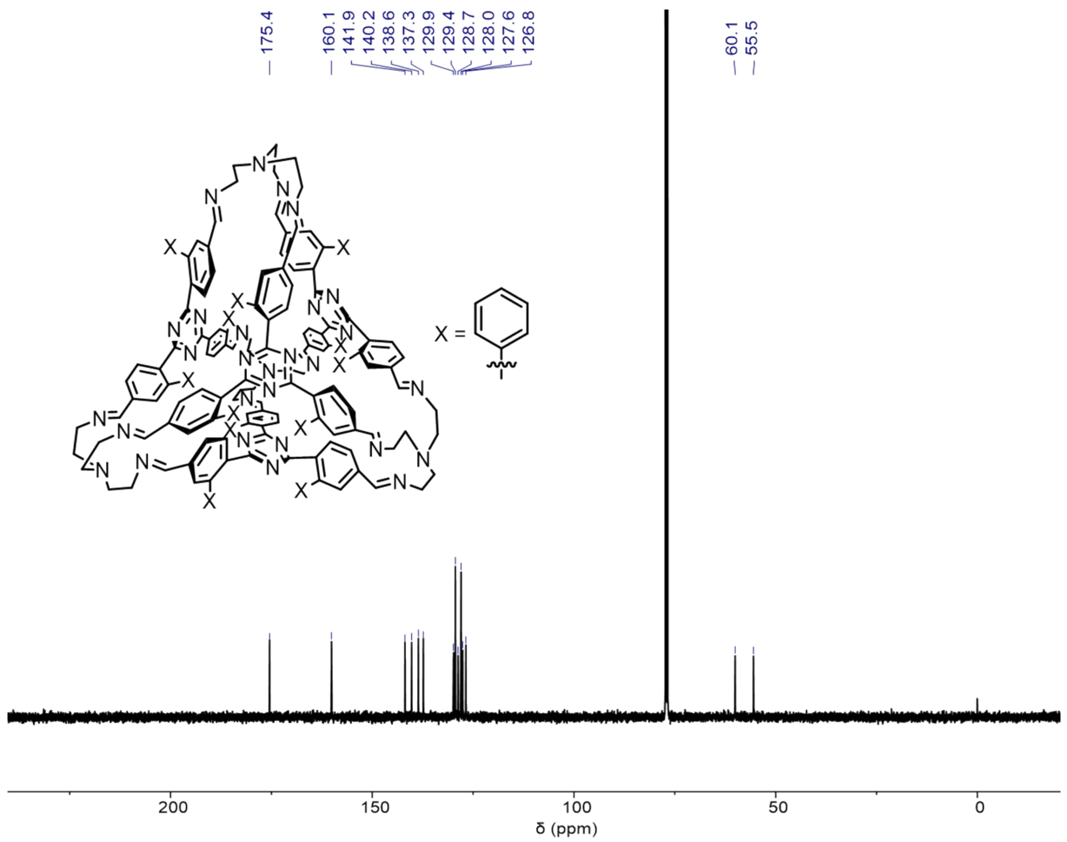

Figure S48. ^{13}C NMR spectra (125 MHz, $CDCl_3$, 298 K) of **2e**.

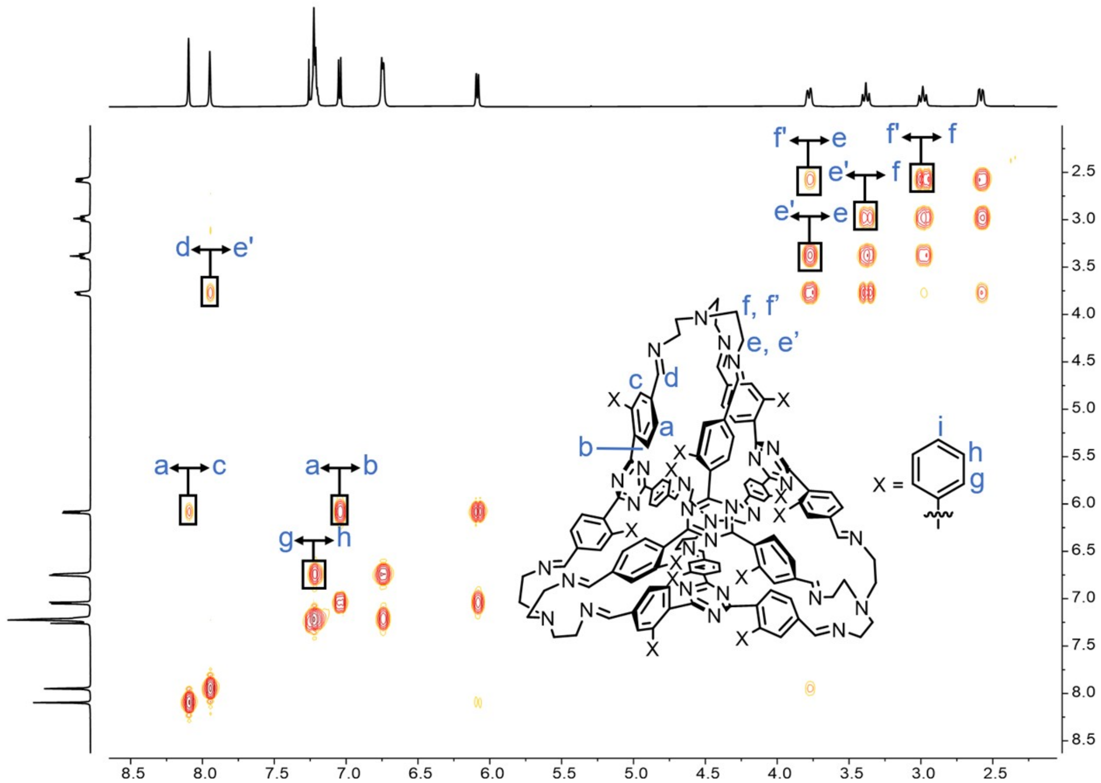

Figure S49. ^1H–^1H COSY spectrum of **2e** (500 MHz, $CDCl_3$, 298 K). The key correlation peaks are labeled in the spectrum.

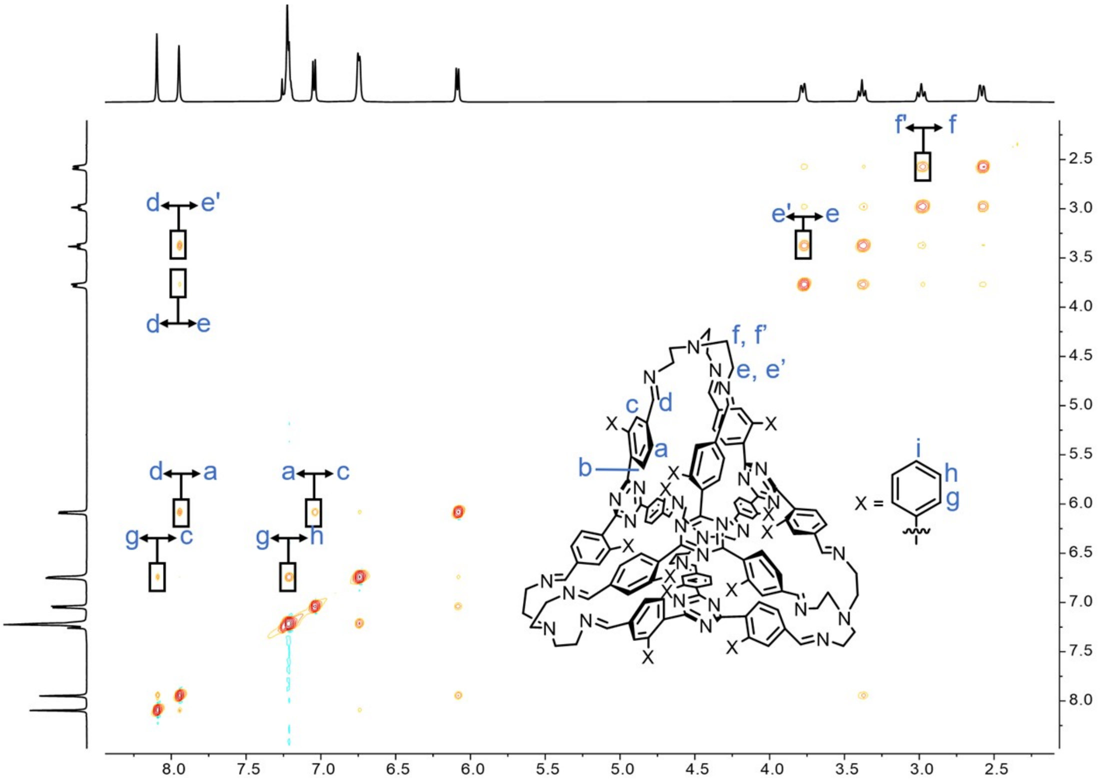

Figure S50. ^1H–^1H NOESY spectrum of cage **2e** (500 MHz, $CDCl_3$, 298K). Through-space proton couplings between some protons are labeled in the spectrum.

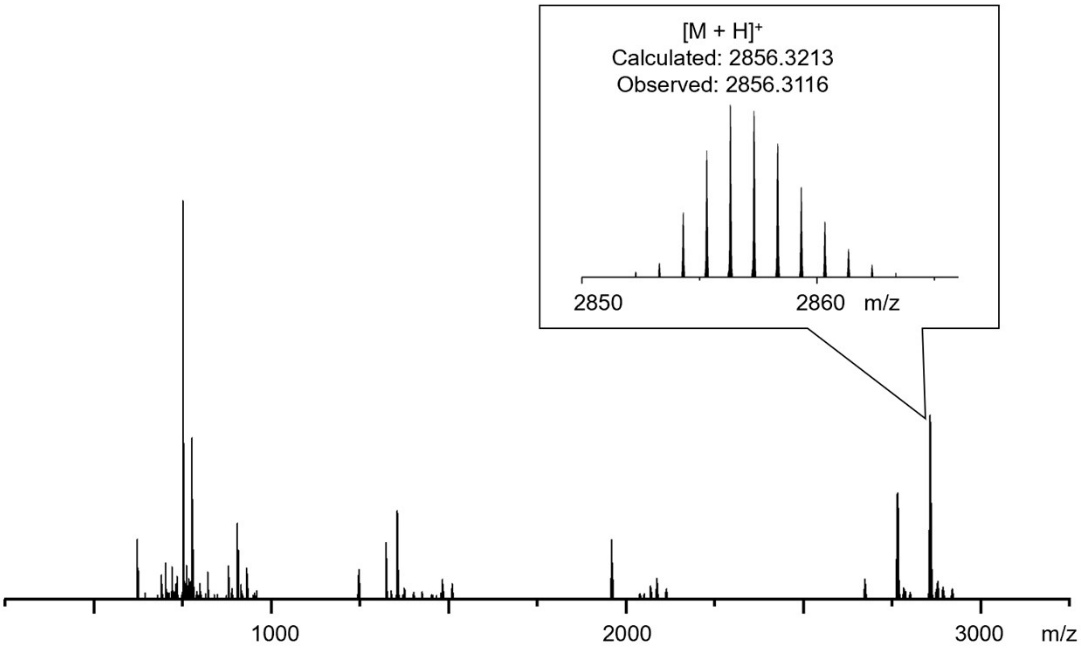

Figure S51. MALDI-QTOF MS of **2e**. Peaks corresponding to molecular ions bearing one positive charge by taking a proton are observed. M is referred to as the cage **2e**.

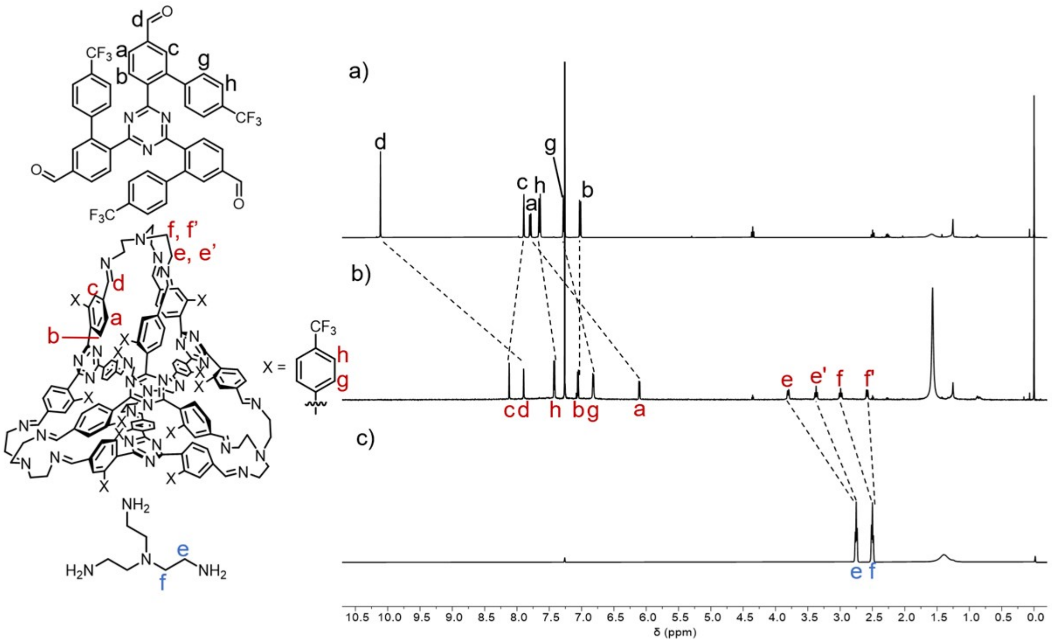

Figure S52. ^{1}H NMR spectra (600 MHz, $CDCl_3$, 298 K) of a) **1f**, b) **2f** and c) TREN.

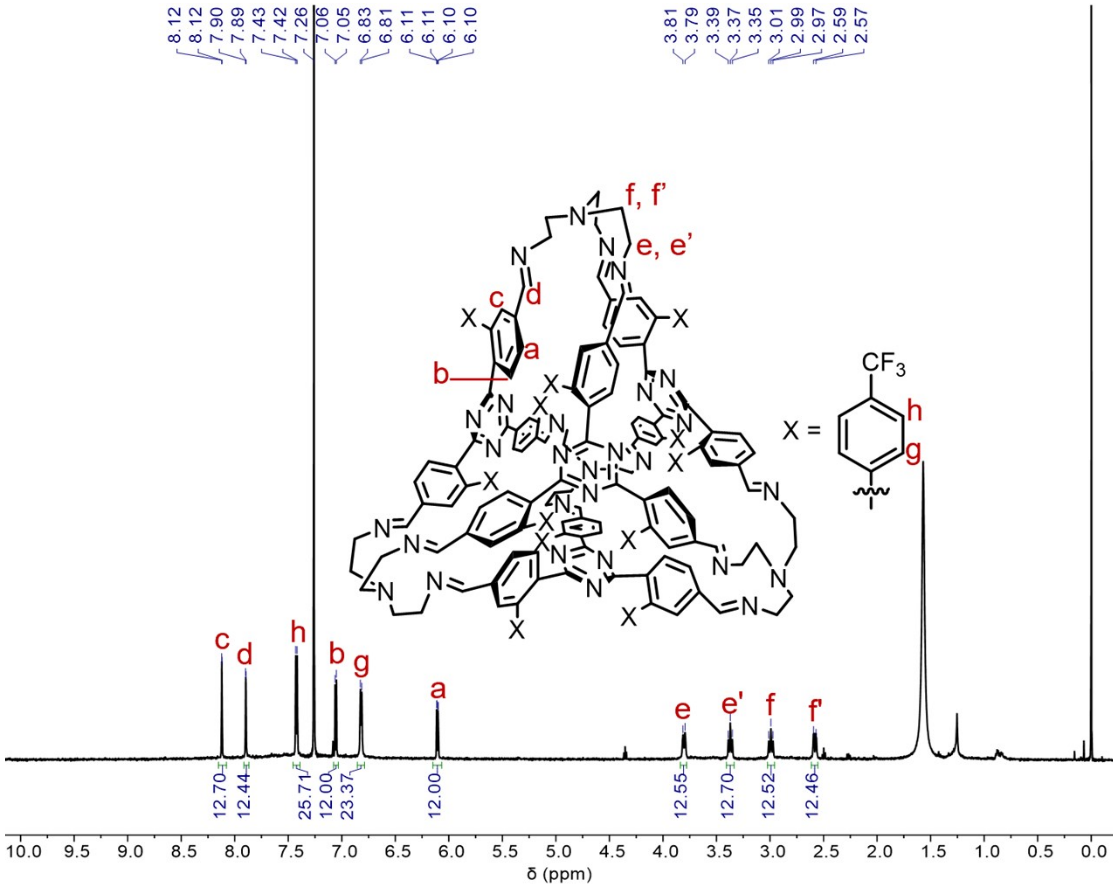

Figure S53. ^{1}H NMR spectra (600 MHz, $CDCl_3$, 298 K) of **2f**.

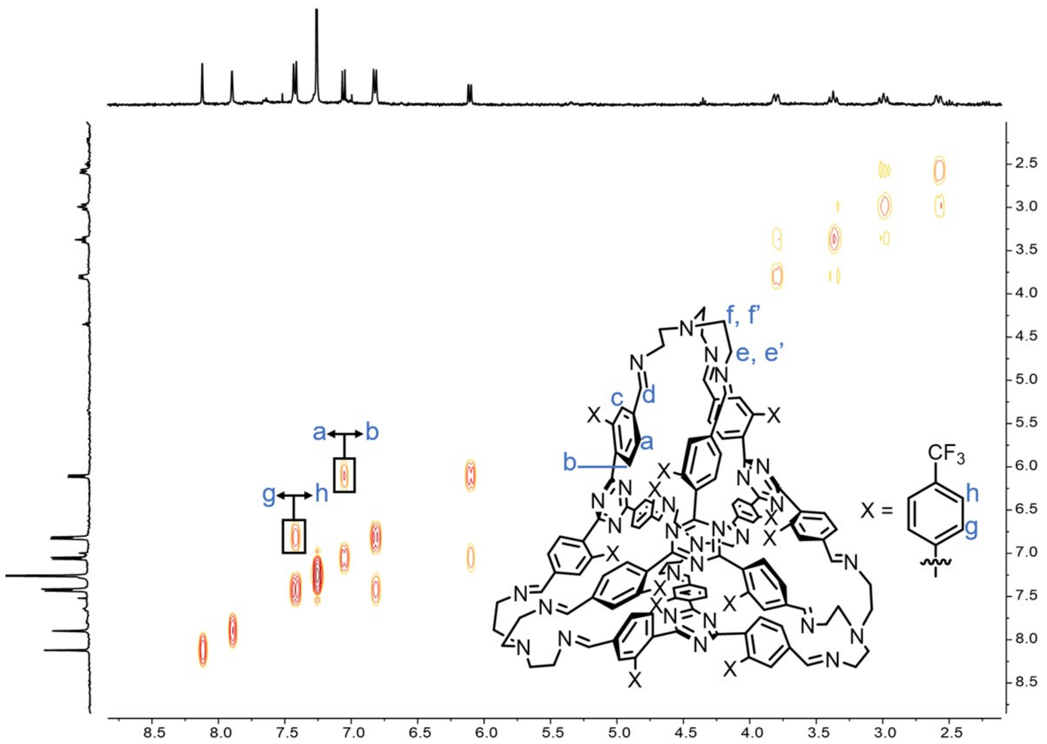

Figure S54. ^{1}H–^{1}H COSY spectrum of **2f** (400 MHz, $CDCl_3$, 298 K). The key correlation peaks are labeled in the spectrum.

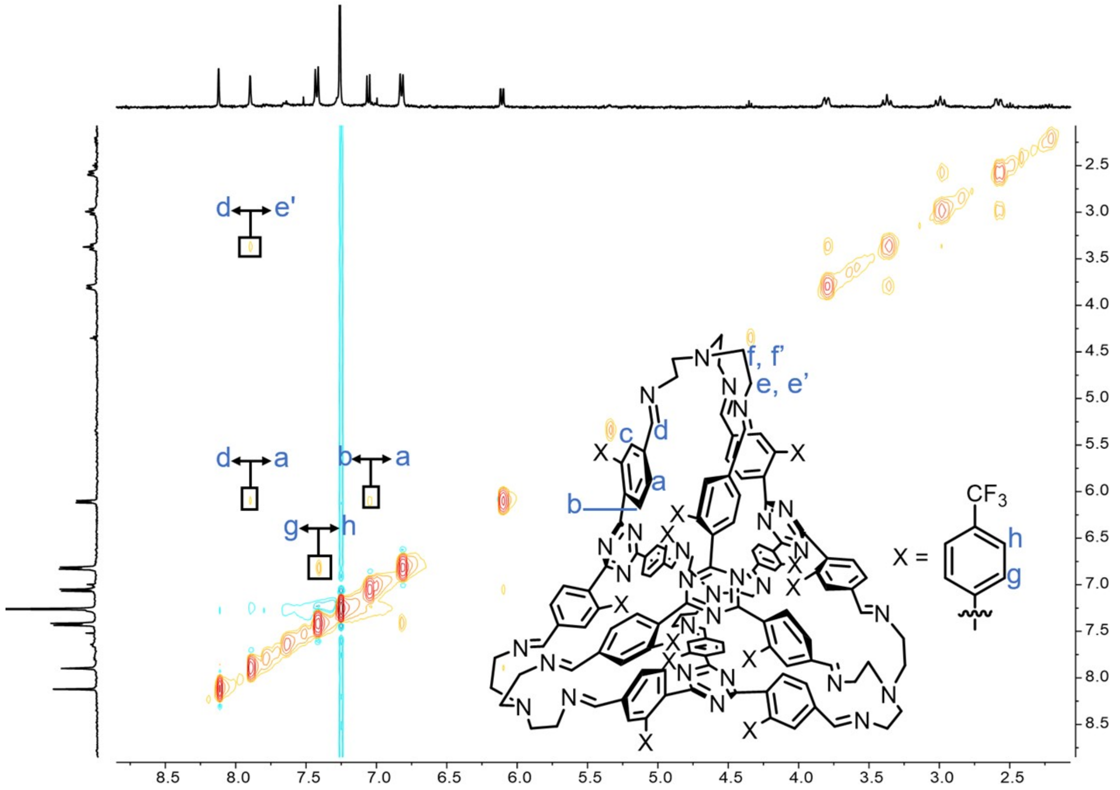

Figure S55. ^{1}H–^{1}H NOESY spectrum of cage **2f** (400 MHz, $CDCl_3$, 298K). Through-space proton couplings between some protons are labeled in the spectrum.

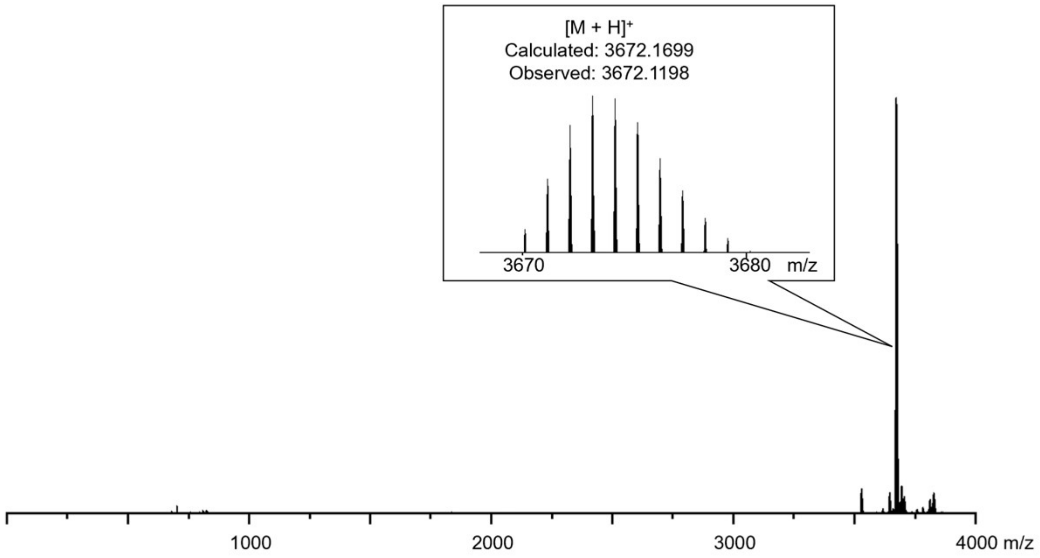

Figure S56. MALDI-QTOF MS of **2f**. Peaks corresponding to molecular ions bearing one positive charge by taking a proton are observed.

5. Kinetic and thermodynamic Studies of binding anionic guests based on ^{1}H NMR Spectroscopic results

5.1 Host-guest recognition investigation of 2c

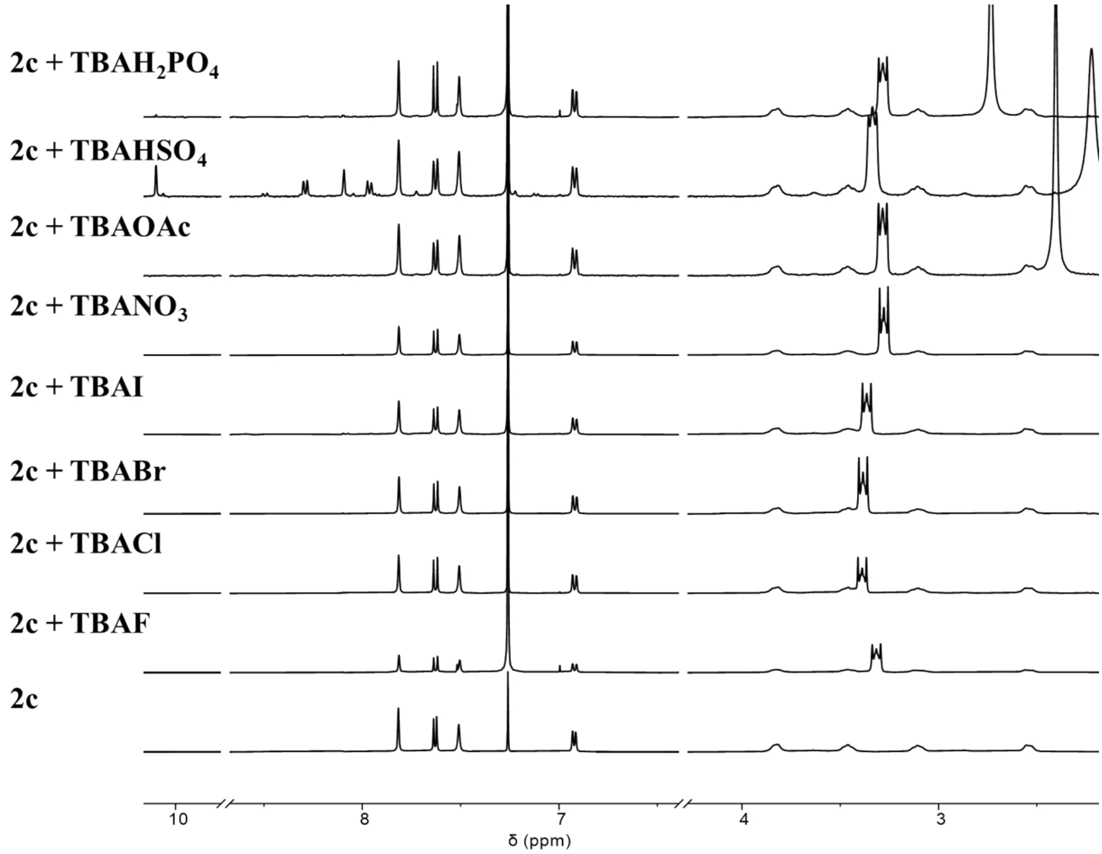

Figure S57. Partial ^{1}H NMR spectra (400 MHz, 298K) of **2c** in 500 µL $CDCl_3$ with 7.5 molar equivalents of different salts with TBA^+ cation. The spectra were recorded 24 h after the addition of salt. No signals corresponding to anion⊂**2c** complexes were found. Addition of $TBA^+{\cdot}HSO_4^-$ led to partial cage decomposition.

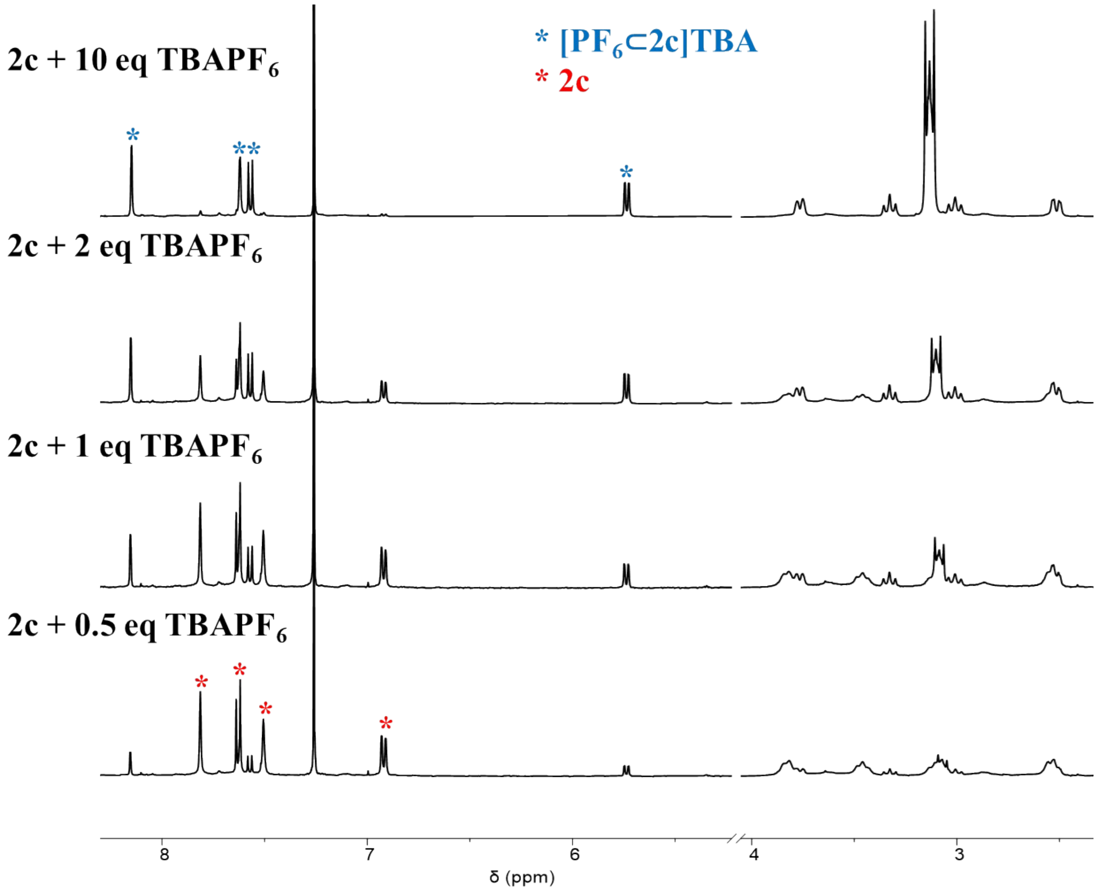

Figure S58. Partial ^{1}H NMR spectra (400 MHz, 298K) of 1 mM of **2c** in 500 μL $CDCl_3$ after addition of different amounts of $TBA^{+}\cdot PF_6^{-}$. The concentration of **2c**, $PF_6^{-}\subset$**2c** and PF_6^{-} were determined by integrating the corresponding resonances in the ^{1}H NMR spectra to calculate the binding constant. From the three independent measurements, K_a was measured to be 843 M^{-1}, 774 M^{-1} and 844 M^{-1}. The averaged value $K_a = (8.2 \pm 0.3) \times 10^{2}$ M^{-1} was thus calculated.

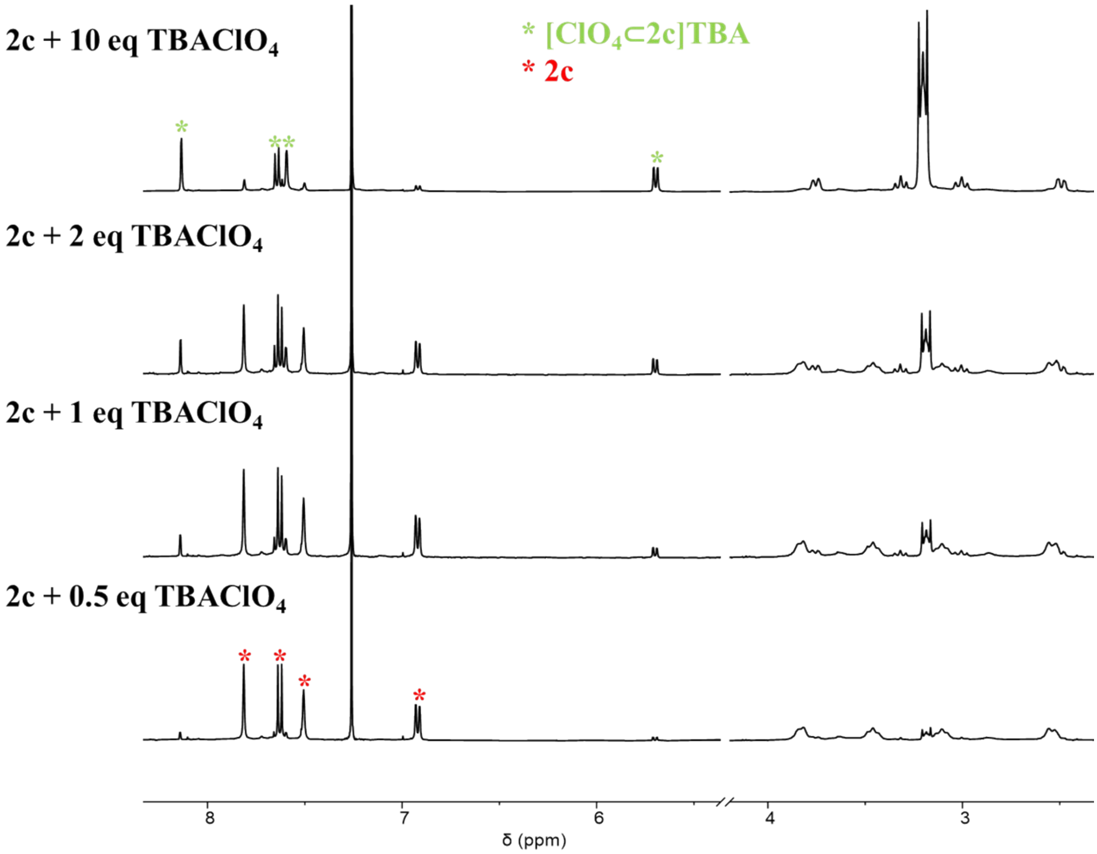

Figure S59. Partial ^{1}H NMR spectra (400 MHz, 298K) of 1 mM of **2c** in 500 μL $CDCl_3$ after addition of different amounts of $TBA^+ \cdot ClO_4^-$. The concentration of **2c**, $ClO_4^- \subset$**2c** and ClO_4^- were determined by integrating the corresponding resonances in the ^{1}H NMR spectra to calculate the binding constant. From the two independent measurements, K_a was found to be 223 M^{-1} and 217 M^{-1}. The averaged value $K_a = (2.2 \pm 0.03) \times 10^2\ M^{-1}$ was thus calculated.

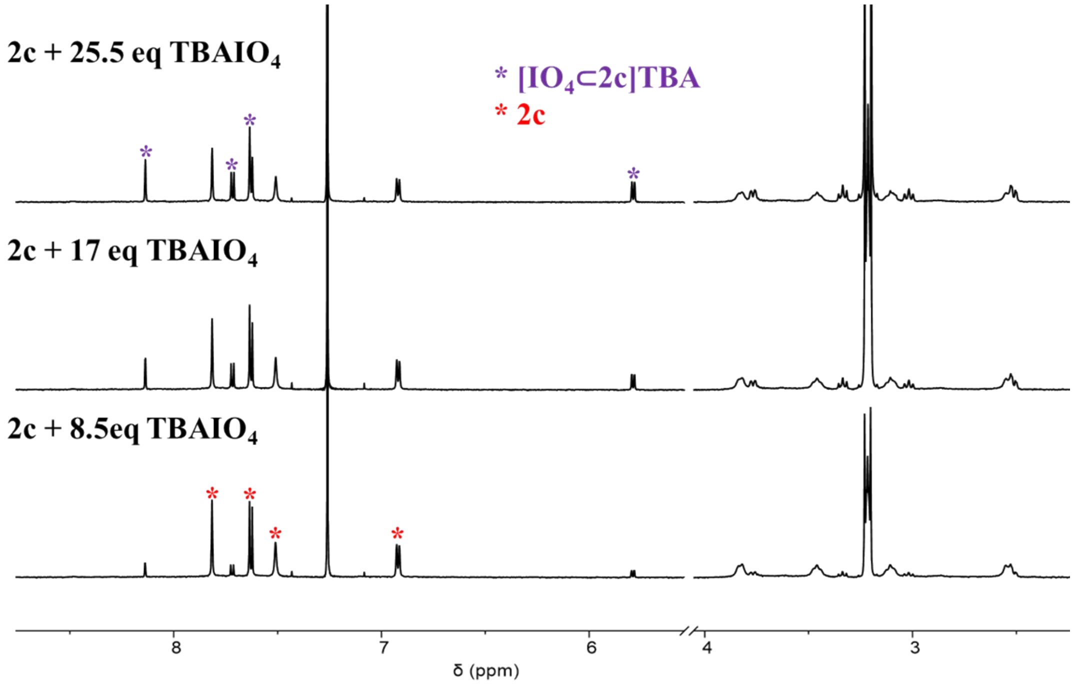

Figure S60. Partial ^{1}H NMR spectra (600 MHz, 298K) of 0.35 mM of **2c** in 600 μL $CDCl_3$ with different amounts of $TBA^+ \cdot IO_4^-$. The concentration of **2c**, IO_4^-⊂**2c** and IO_4^- were determined by integrating the corresponding resonances in the ^{1}H NMR spectra to calculate the binding constant. From the three independent measurements, K_a was found to be 61 M^{-1}, 74 M^{-1} and 78 M^{-1}. The averaged value $K_a = (0.7 \pm 0.07) \times 10^2$ M^{-1} was calculated.

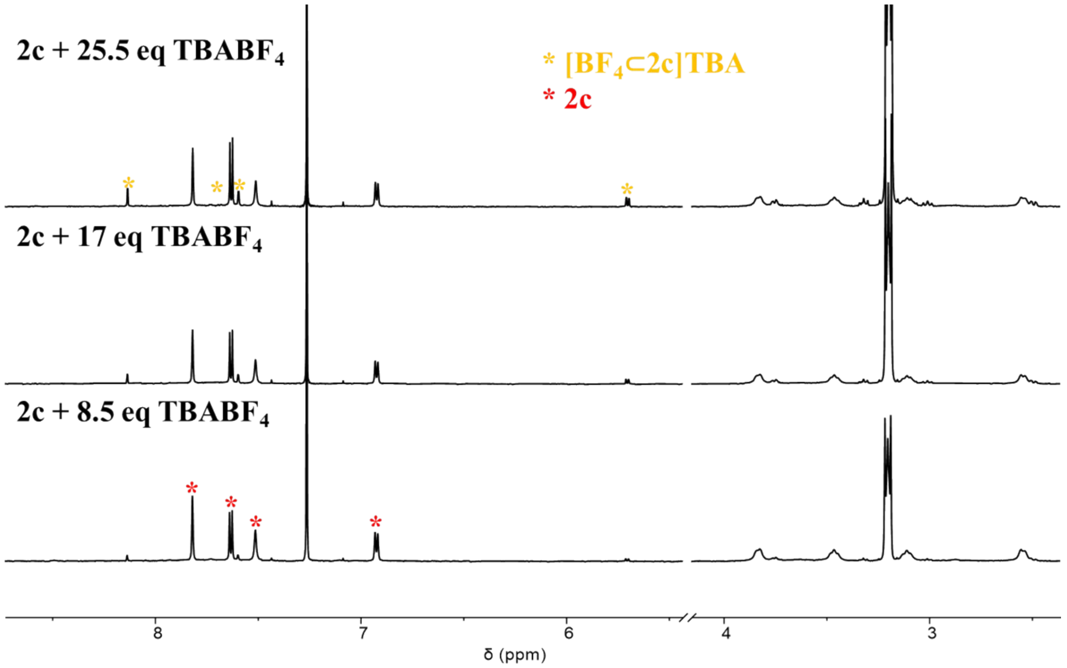

Figure S61. Partial ^{1}H NMR spectra (600 MHz, 298K) of 0.35 mM of **2c** in 600 μL $CDCl_3$ with different amounts of $TBA^+{\cdot}BF_4^-$. The concentration of **2c**, BF_4^-⊂**2c** and BF_4^- were determined by integrating the corresponding resonances in the ^{1}H NMR spectra to calculate the binding constant. From the three independent measurements, K_a was found to be 23 M^{-1}, 29 M^{-1} and 30 M^{-1}. The averaged value $K_a = (0.3 \pm 0.04) \times 10^2$ M^{-1} was calculated.

5.2 Thermodynamic studies of 2d

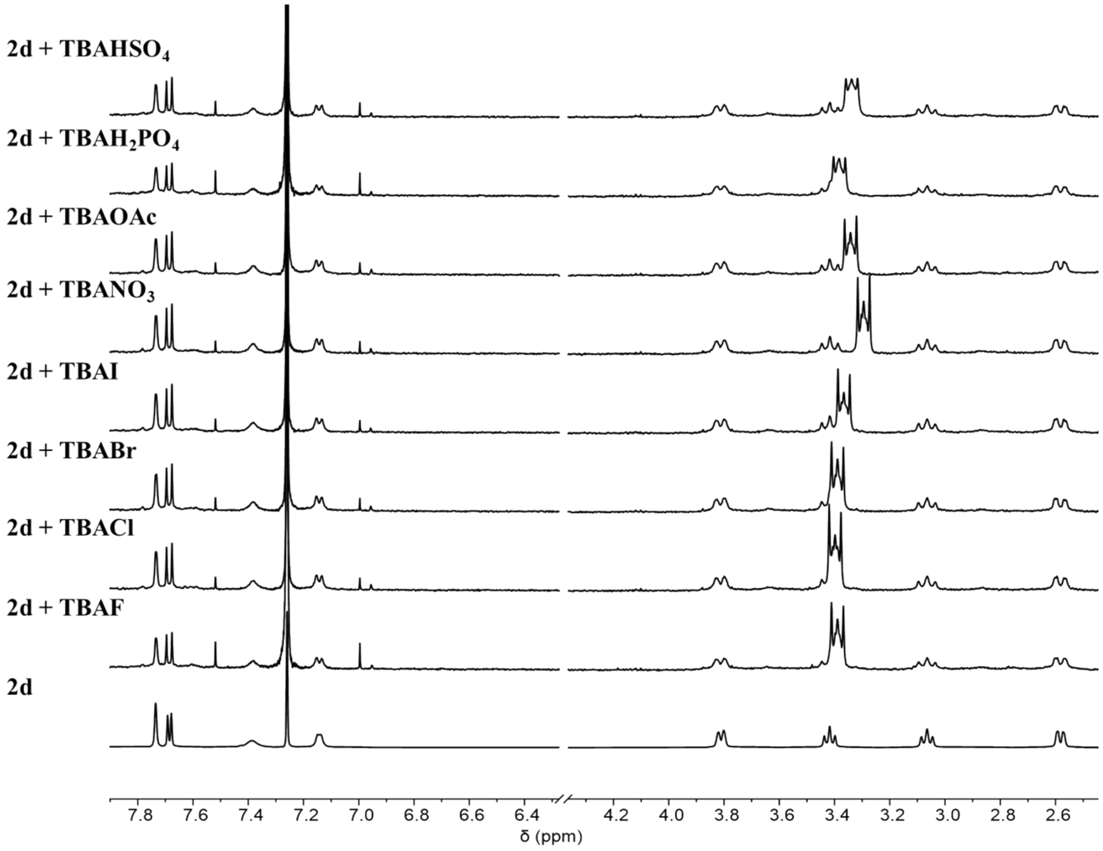

Figure S62. Partial ^{1}H NMR spectra (400 MHz, 298K) of **2d** in 500 μL $CDCl_3$ with 5 molar equivalents of different anions whose counterion are all TBA^+ cation. The spectra were taken 24 h after the salt addition, indicating no resonances corresponding to anion⊂**2d** complexes were found.

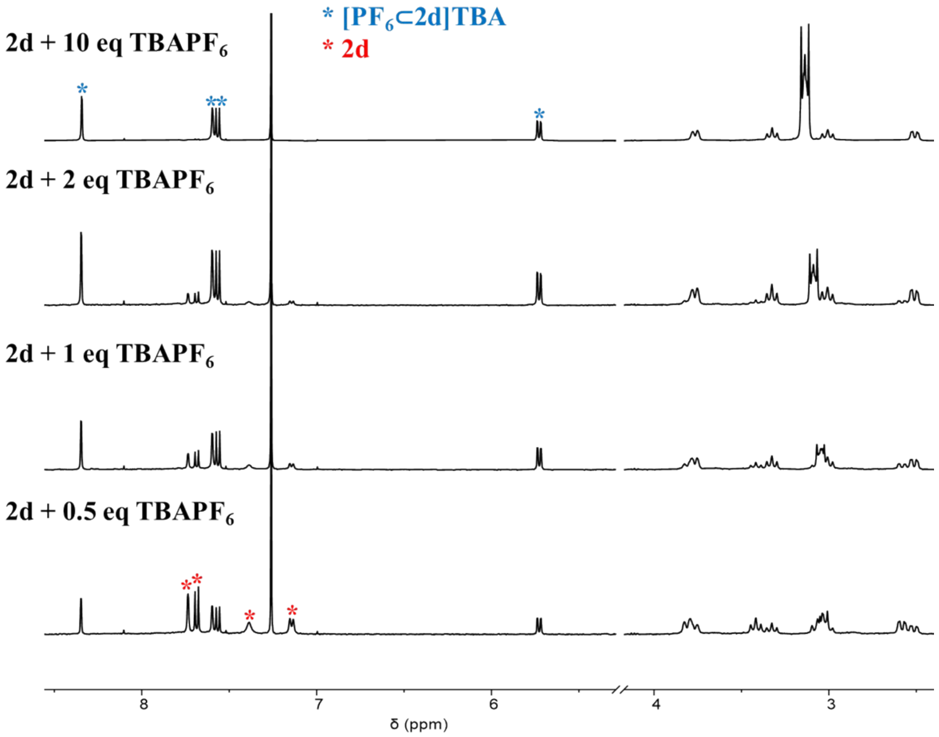

Figure S63. Partial ^{1}H NMR spectra (400 MHz, 298K, $CDCl_3$) of **2d** in $CDCl_3$ (500 μL) with different amounts of $TBA^+ \cdot PF_6^-$. The concentration of **2d**, PF_6^-⊂**2d** and PF_6^- were determined by integrating the resonances in ^{1}H NMR spectra to calculate the binding constant. From the three independent measurements, K_a was calculated to be 5812 M^{-1}, 6246 M^{-1} and 4928 M^{-1}. The average K_a was thus determined to be $(5.6 \pm 0.5) \times 10^3$ M^{-1}.

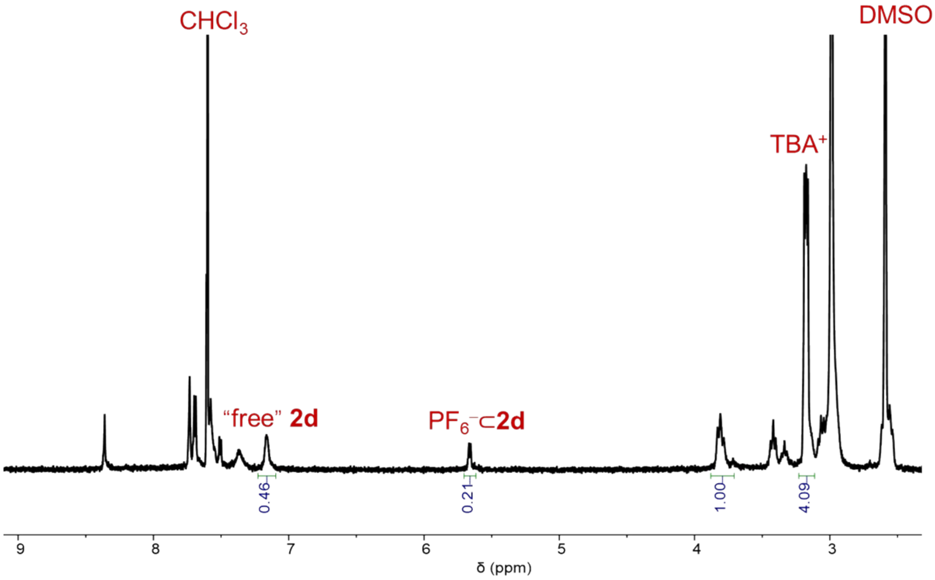

Figure S64. Partial ^{1}H NMR spectra (600 MHz, 298 K) of **2d** (0.5 mM) with $TBAPF_6$ (3.0 mM) in $CDCl_3/CD_3SOCD_3$ (5:1 v/v). K_a was thus determined to be 1.5×10^2 M^{-1}.

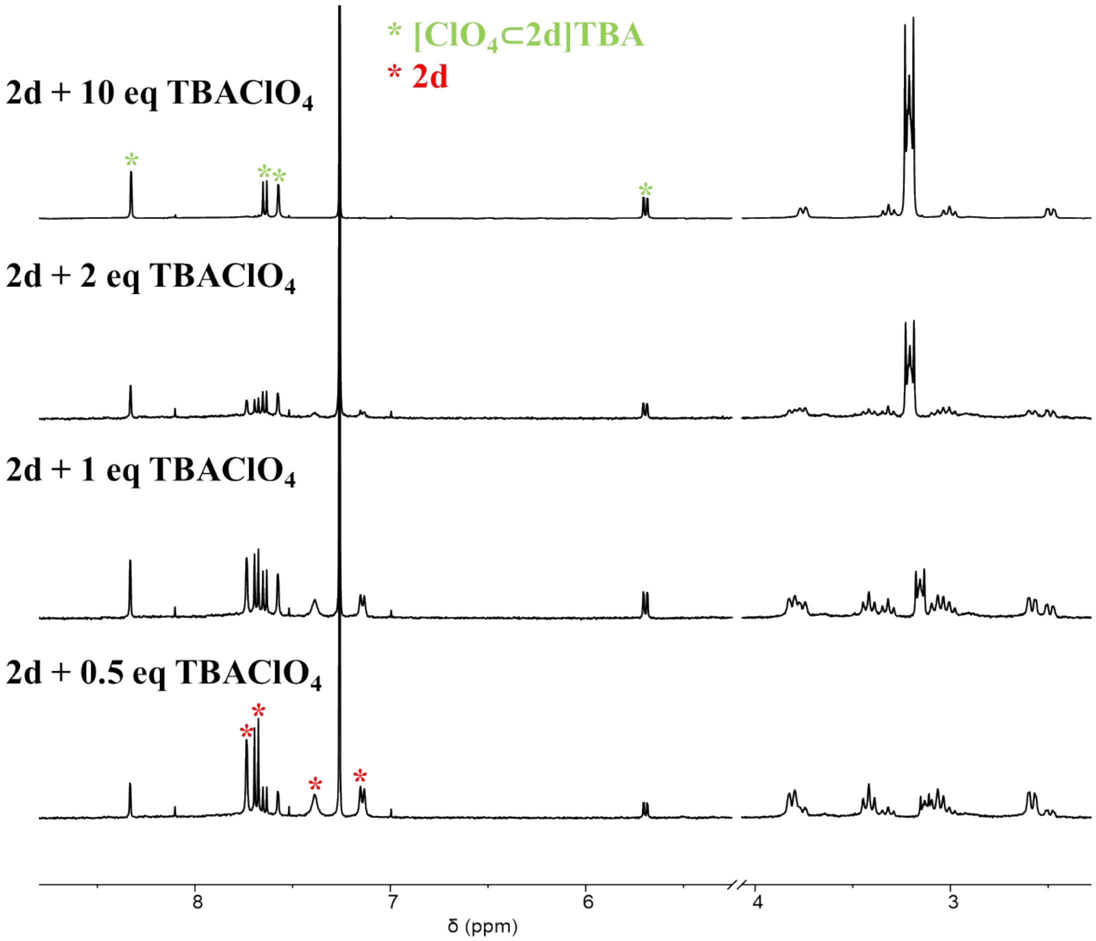

Figure S65. Partial ^{1}H NMR spectra (400 MHz, 298K, $CDCl_3$) of **2d** (1 mM) in $CDCl_3$ (500 μL) with different amounts of $TBA^+ \cdot ClO_4^-$. The concentration of **2d**, ClO_4^-⊂**2d** and ClO_4^- were determined by integrating the resonances in ^{1}H NMR spectra to calculate the binding constant. From the three independent measurements, K_a was calculated to be 1007, 1111 and 973 M^{-1}. The average K_a was thus determined to be $(1.0 \pm 0.06) \times 10^3$ M^{-1}.

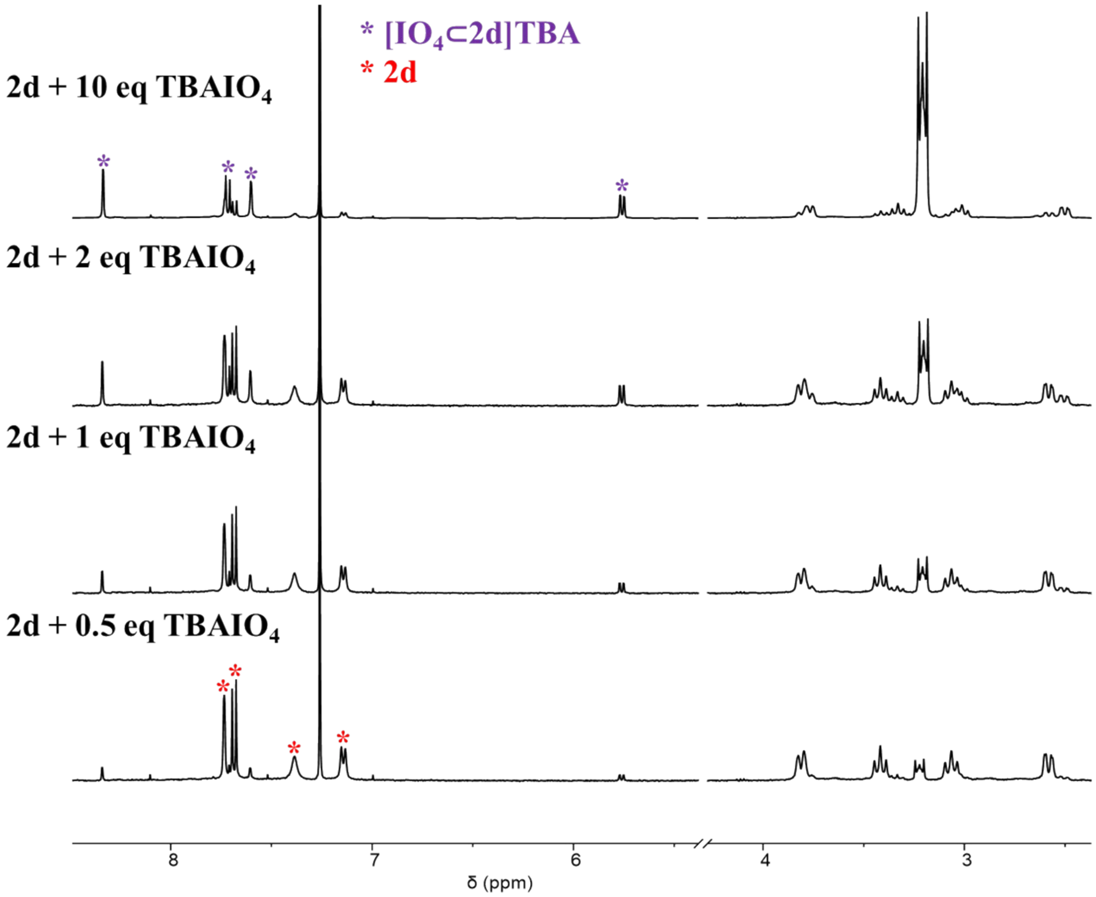

Figure S66. Partial ^{1}H NMR spectra (400 MHz, 298K, $CDCl_3$) of **2d** (1 mM) in $CDCl_3$ (500 μL) with different amounts of $TBA^{+}\cdot IO_4^{-}$. The concentration of **2d**, IO_4^{-}⊂**2d** and IO_4^{-} were determined by integrating the resonances in ^{1}H NMR spectra to calculate the binding constant. From the three independent measurements, K_a was calculated to be 241, 247 and 265 M^{-1}. The average K_a was thus determined to be $(2.5 \pm 0.1) \times 10^{2}$ M^{-1}.

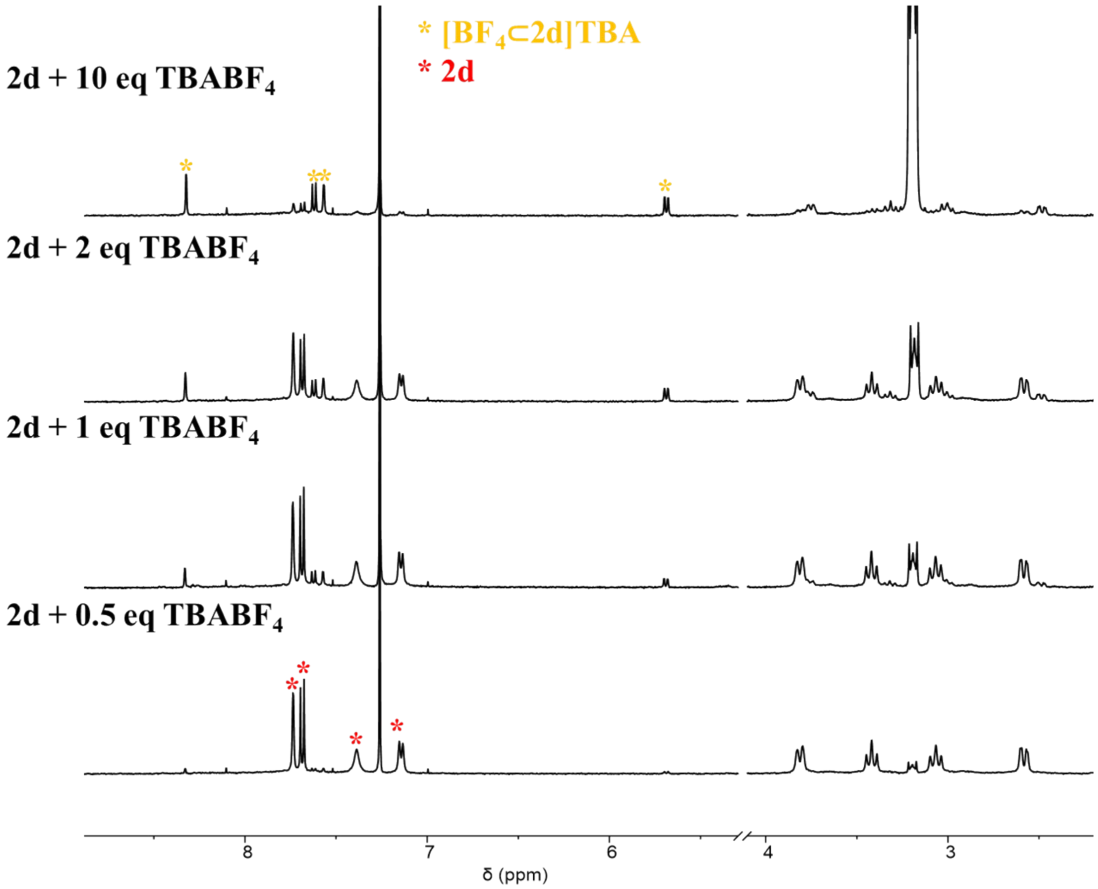

Figure S67. Partial ^{1}H NMR spectra (400 MHz, 298K, $CDCl_3$) of **2d** (1 mM) in $CDCl_3$ (500 μL) with different amounts of $TBA^+ \cdot BF_4^-$. The concentration of **2d**, $BF_4^- \subset$**2d** and BF_4^- were determined by integrating the resonances in ^{1}H NMR spectra to calculate the binding constant. From the three independent measurements, K_a was calculated to be 155, and 158 M^{-1}. The average K_a was thus determined to be $(1.6 \pm 0.02) \times 10^2$ M^{-1}.

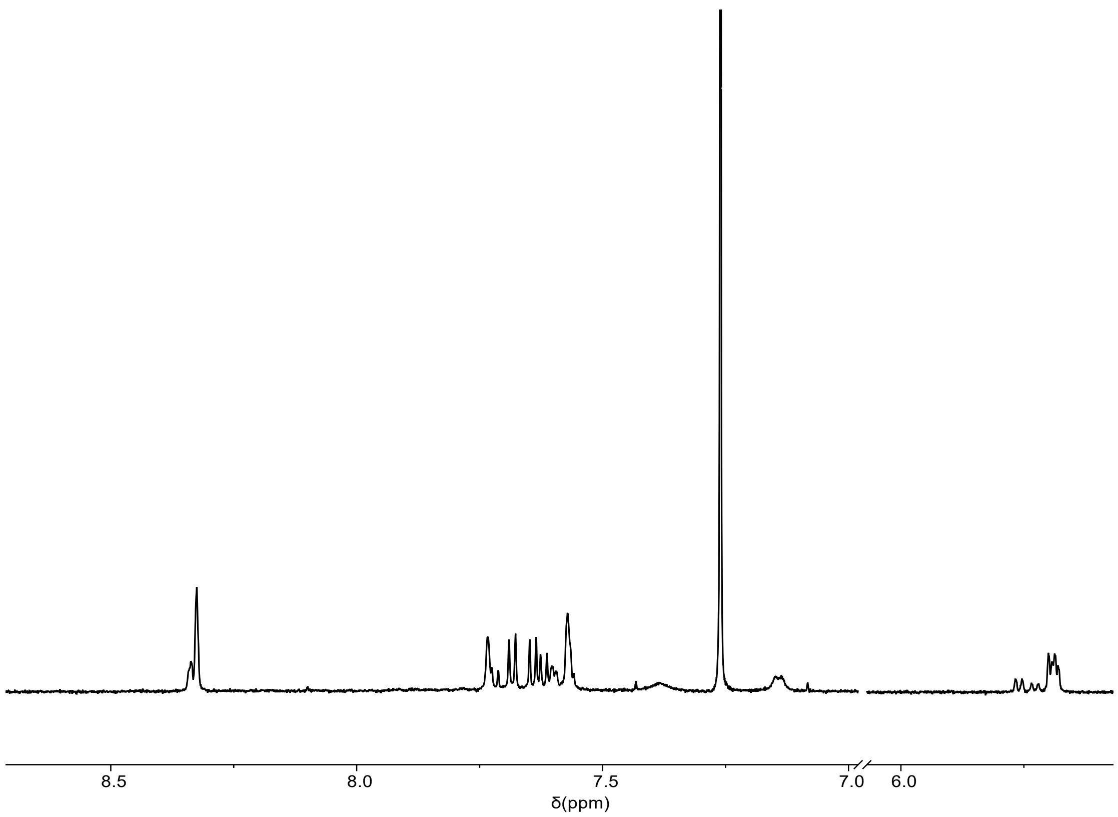

Figure S68. Partial ^{1}H NMR spectra (600 MHz, 298K, $CDCl_3$) of 0.5mM **2d** after addition of $TBA^{+}\cdot PF_6^{-}$, $TBA^{+}\cdot BF_4^{-}$, $TBA^{+}\cdot ClO_4^{-}$, and $TBA^{+}\cdot IO_4^{-}$. The concentration of each salt is 3 mM. The ^{1}H NMR spectrum was recorded 12 h after anion addition, in order to allow the cage-anion complexation reach the equilibrium.

5.3 Thermodynamic studies of 2b, 2e

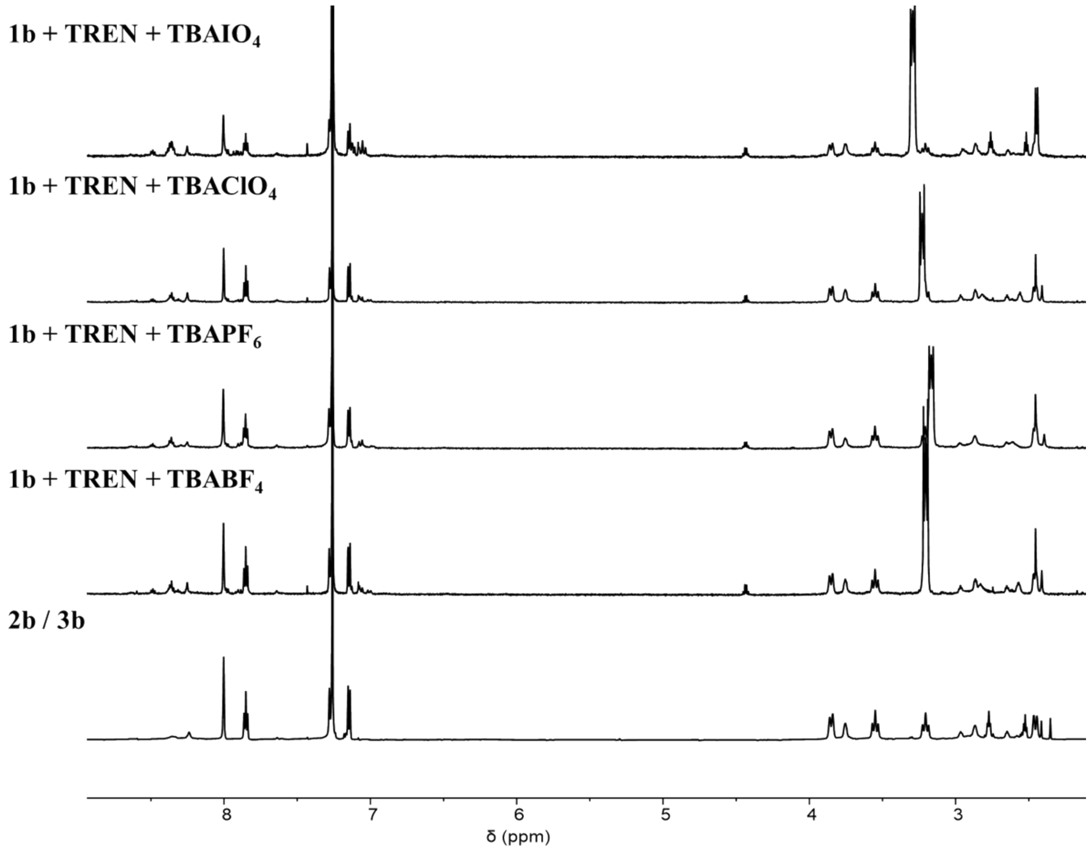

Figure S69. The spectra of mixtures each containing **1b** (0.002 mmol) and TREN (0.002 mmol) in 600 μL $CDCl_3$ in the presence of 0.6 equiv. of different TBA^+ salts. The spectra indicated that "free" cage **2b** was produced, without forming the putative cage-anion complexes.

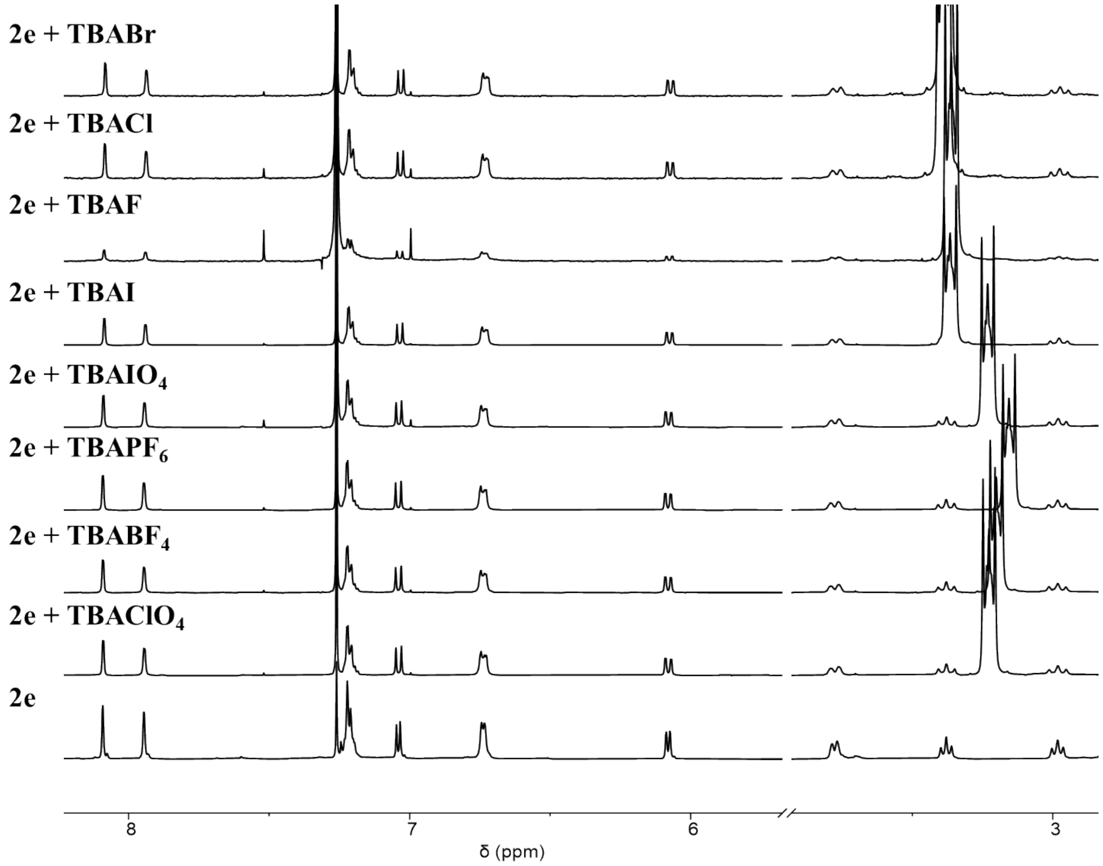

Figure S70. Partial ^{1}H NMR spectra (400 MHz, 298K) of **2e** in 500 μL $CDCl_3$ after adding 20 molar equivalents of TBA^+ salts. The spectra were taken 8 d after the salt addition. No resonances corresponding to anion⊂**2e** complexes were observed.

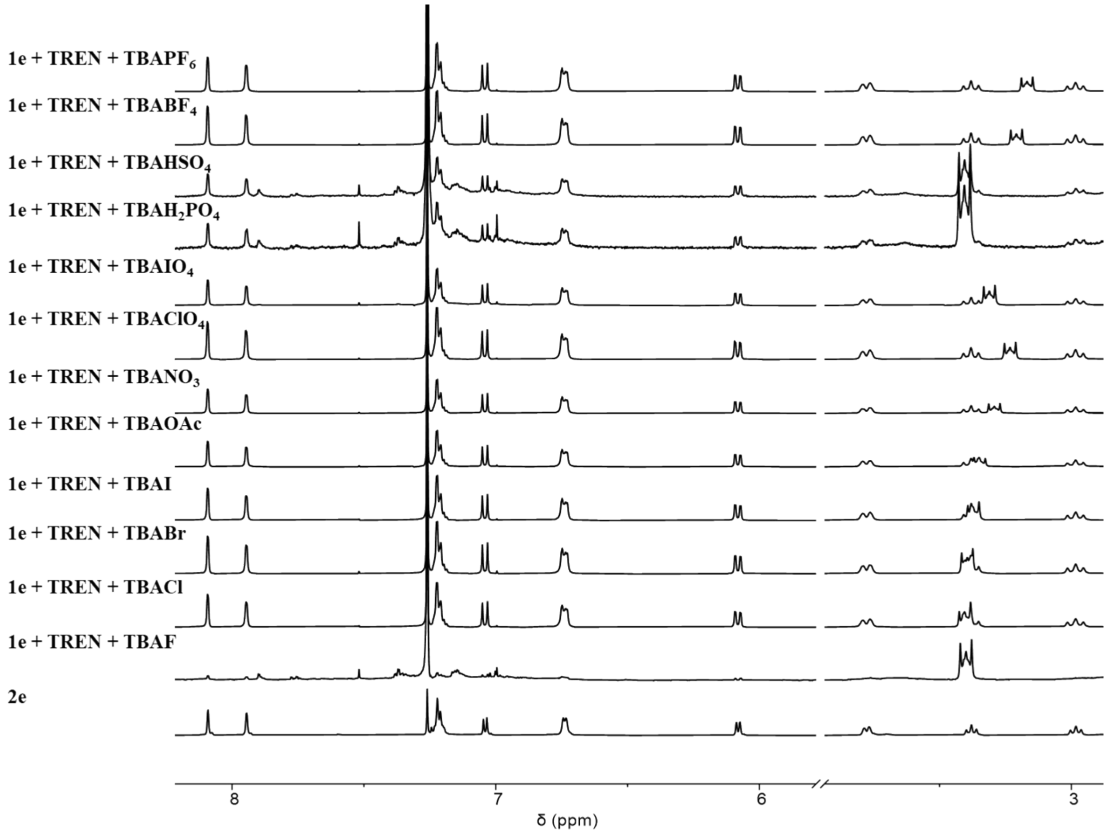

Figure S71. The ^{1}H NMR spectra of mixtures each containing **1e** (0.0016 mmol) and TREN (0.0016 mmol) in 600 μL $CDCl_3$ in the presence of 0.5 equiv. of TBA^+ salts. The spectra indicated that "free" cage **2e** was produced in most cases. In the presence of F^-, the self-assembly was unsuccessful.

5.4 Thermodynamic studies of 2f

The rates of association of **2f** with the anionic guests proved to be small, which often took days. The binding constants were thus determined by using the corresponding anionic guests to template the cage formation. General procedure: **1f** (0.002 mmol) and TREN (0.002 mmol) were condensed in 500 μL $CDCl_3$ at 55 °C for 12 h in the presence of the anionic guests that are 0.25 eq, 0.5eq, and 1 eq relative to **1f**. The overall concentration of the anions added were equal to the counterion namely TBA^+, whose concentration could be determined by integrating its resonances. The concentrations of **2f**, anion⊂**2f** could be determined by integrating the corresponding resonances in the ^1H NMR (400 MHz, 298 K) spectra. The concentrations of the free anions were calculated by using the equation, namley [anion] = [TBA^+]–[anion⊂**2f**].

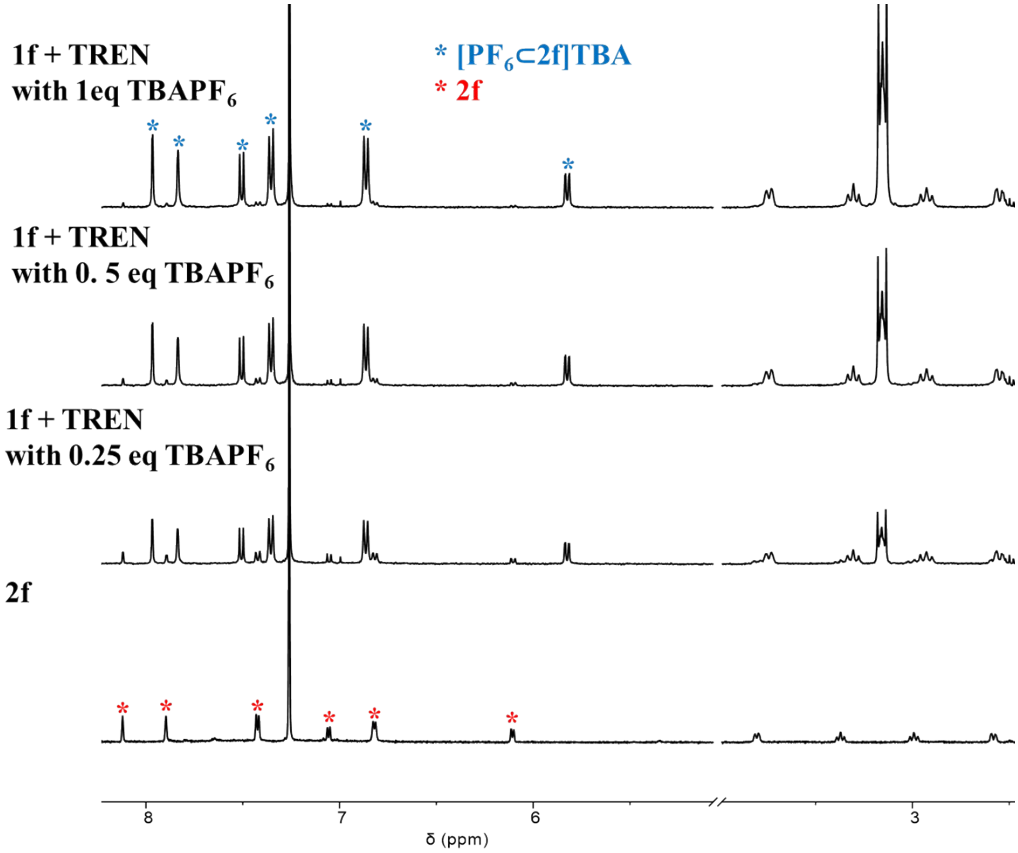

Figure S72. The ^1H NMR spectra of a mixture **1f** (0.002 mmol) and TREN (0.002 mmol) in 500 μL $CDCl_3$ in the presence of 0, 0.25, 0.5 and 1 equiv. of $TBA^+ \cdot PF_6^-$ relative to **1f**. The resonances corresponding to the free cage **2f** were labelled with red asterisks in the bottom spectrum, while those corresponding to the complex were labelled with blue asterisks in the top one. By using these spectra, K_a was determined to be $(5.7 \pm 0.4) \times 10^3$ M^{-1}.

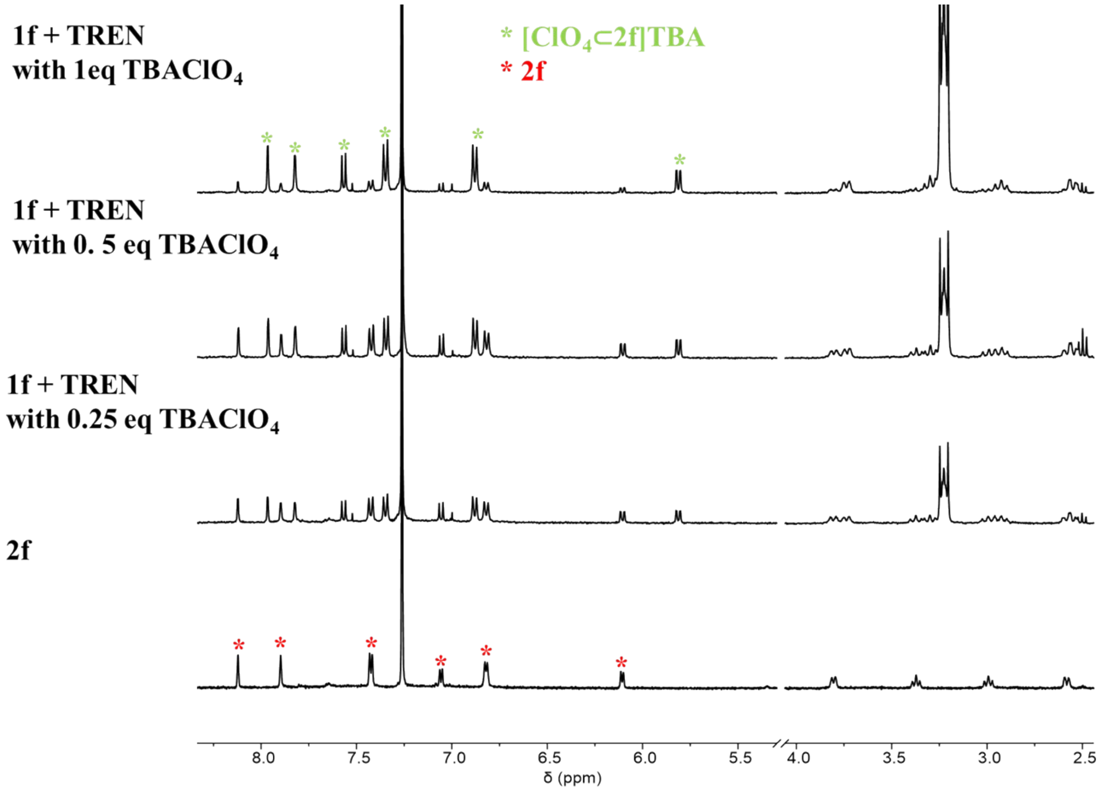

Figure S73. The ^{1}H NMR spectra of a mixture **1f** (0.002 mmol) and TREN (0.002 mmol) in 500 μL $CDCl_3$ in the presence of 0, 0.25, 0.5 and 1 equiv. of $TBA^+ \cdot ClO_4^-$ relative to **1f**. The resonances corresponding to the free cage **2f** were labelled with red asterisks in the bottom spectrum, while those corresponding to the complex $ClO_4^- \subset$**2f** were labelled with blue asterisks in the top one. By using these spectra, K_a of $ClO_4^- \subset$**2f** was determined to be $(1.1 \pm 0.3) \times 10^3$ M^{-1}.

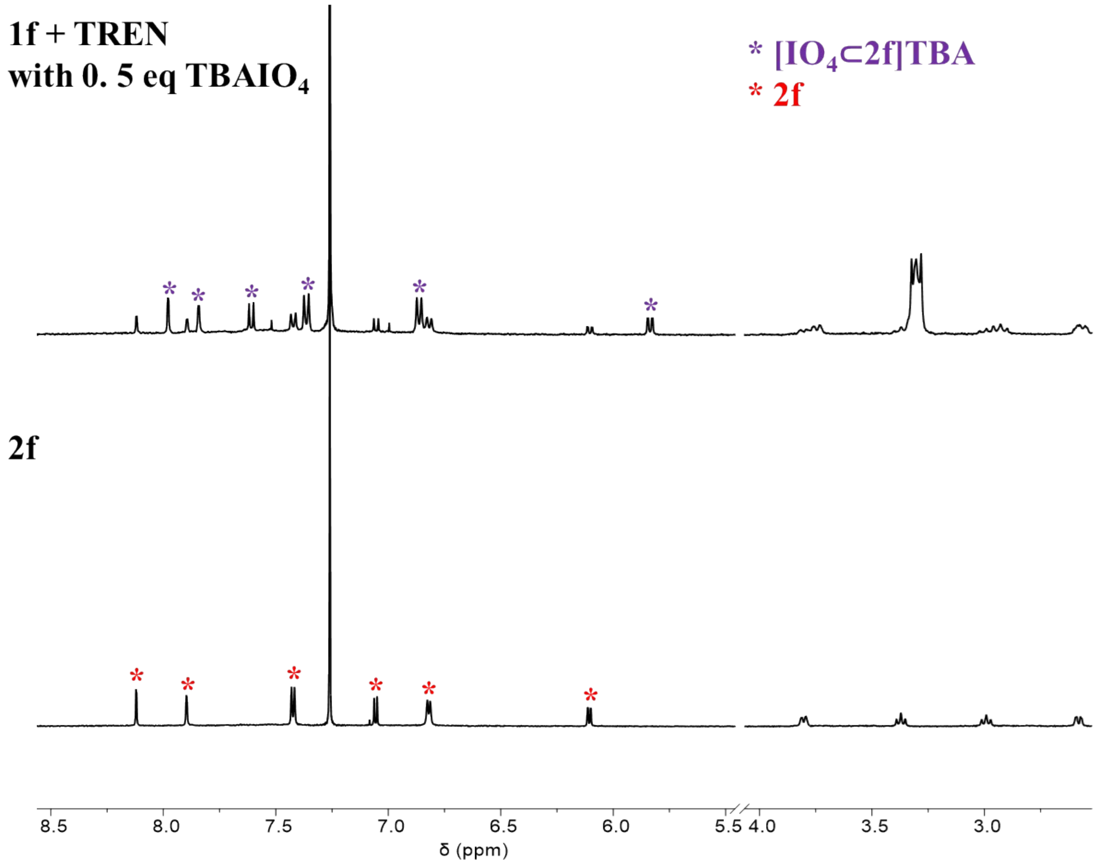

Figure S74. The ^{1}H NMR spectra of a mixture **1f** (0.002 mmol) and TREN (0.002 mmol) in 500 μL $CDCl_3$ in the presence of 0, and 0.5 equiv. of $TBA^+ \cdot IO_4^-$ relative to **1f**. The resonances corresponding to the free cage **2f** were labelled with red asterisks in the bottom spectrum, while those corresponding to the complex IO_4^-⊂**2f** were labelled with blue asterisks in the top one. By using these spectra, K_a of IO_4^-⊂**2f** was determined to be $(1.2 \pm 0.02) \times 10^3$ M^{-1}.

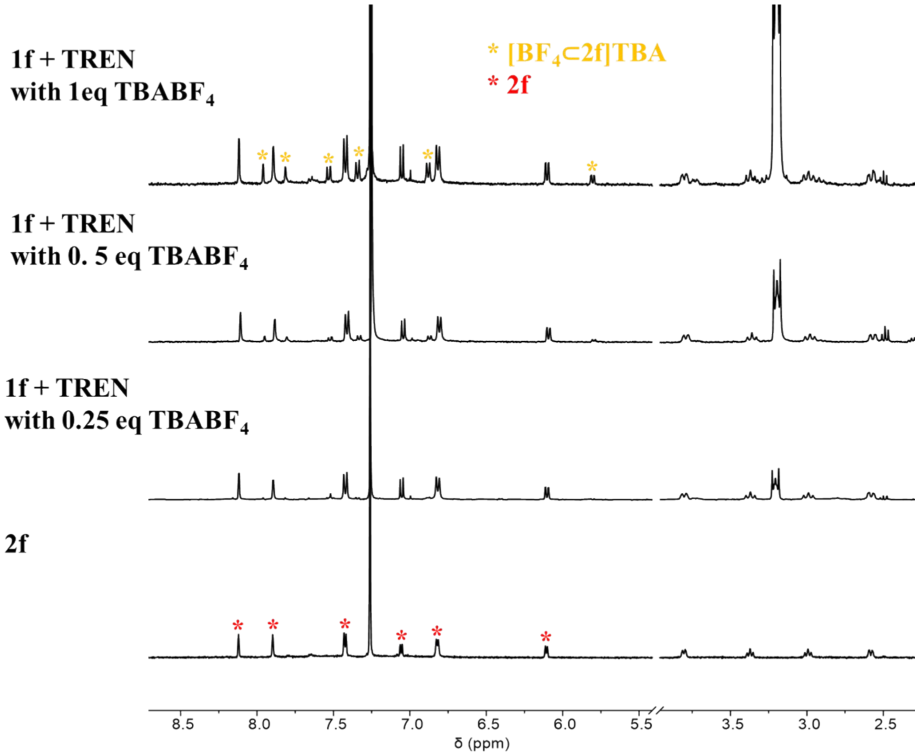

Figure S75. The ^{1}H NMR spectra of a mixture **1f** (0.002 mmol) and TREN (0.002 mmol) in 500 μL $CDCl_3$ in the presence of 0, and 0.5 equiv. of $TBA^{+}\cdot BF_4^{-}$ relative to **1f**. The resonances corresponding to the free cage **2f** were labelled with red asterisks in the bottom spectrum, while those corresponding to the complex BF_4^{-}⊂**2f** were labelled with blue asterisks in the top one. By using these spectra, K_a of BF_4^{-}⊂**2f** was determined to be $(0.9 \pm 0.07) \times 10^2$ M^{-1}.

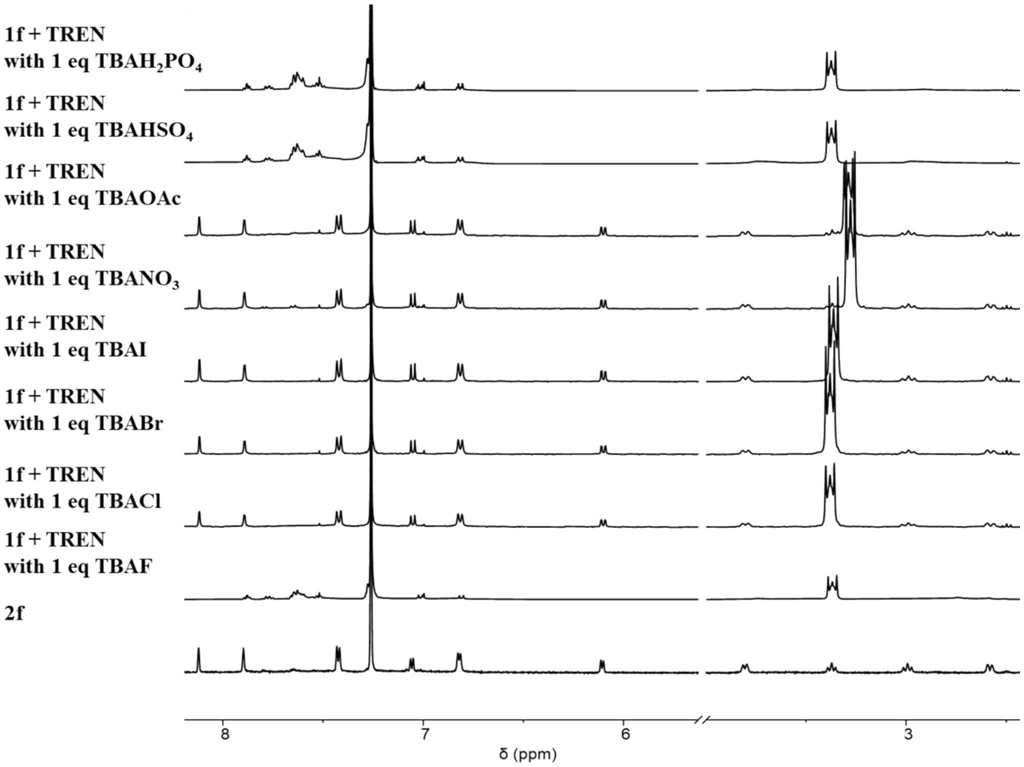

Figure S76. The ^{1}H NMR spectra of mixtures each containing **1f** (0.002 mmol) and TREN (0.002 mmol) in 500 μL $CDCl_3$ in the presence of 1 equiv. of TBA^+ salts. The spectra indicated that "free" cage **2e** was produced in most cases. In the presence of F^-, the self-assembly was unsuccessful. In the case of HSO_4^- and $H_2PO_4^-$, the cage was partially decomposed due to the acidity.

5.5 Kinetic studies of 2d to recognize anions

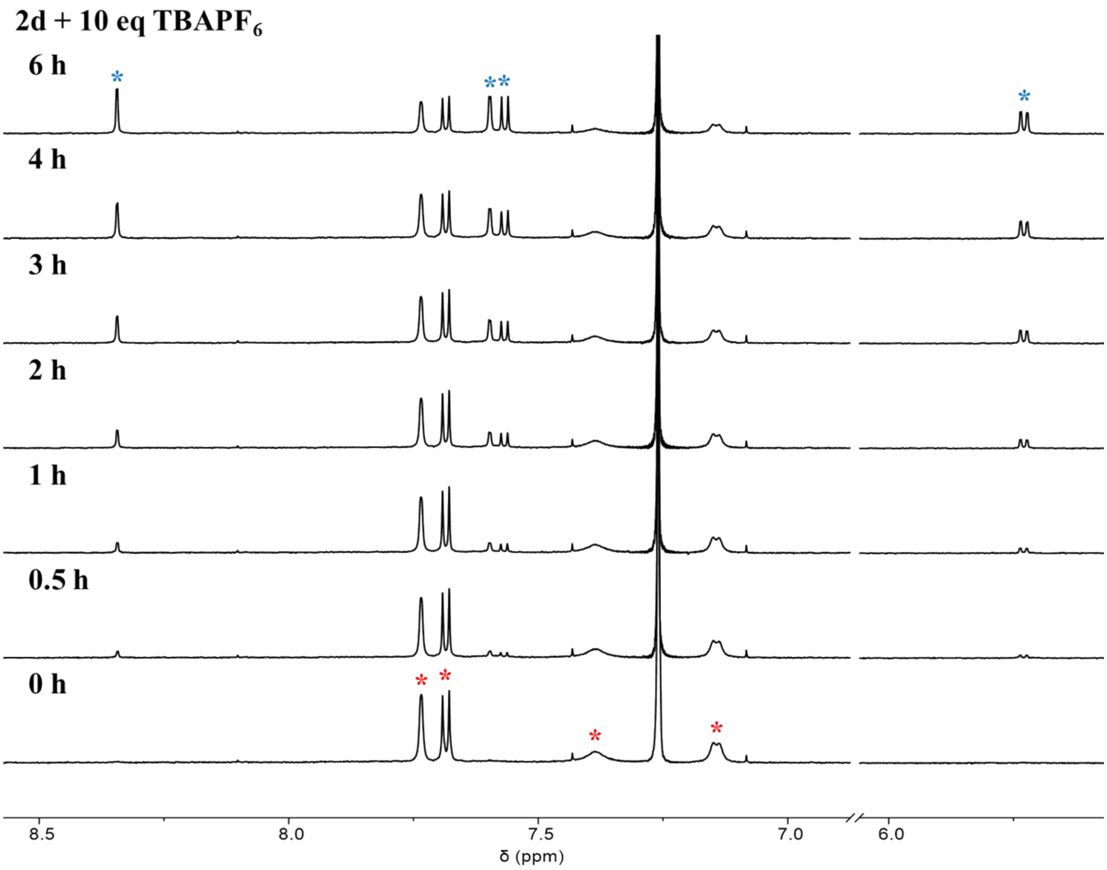

Figure S77. Partial ^{1}H NMR spectra (600 MHz, 298 K, $CDCl_3$) of a mixture of **2d** (0.3 mM) and $TBA^+ \cdot PF_6^-$ (3 mM), which were recorded for a certain amount of time including 0 (bottom) and 6 h (top). The resonances corresponding to the free cage and cage-anion complex were labelled with red and blue asterisks, respectively. The pseudo-first-order rate constant, k_{obs}, was calculated to be 11.3×10^{-2} h^{-1}, assuming that the concentration of free anion remained constant.

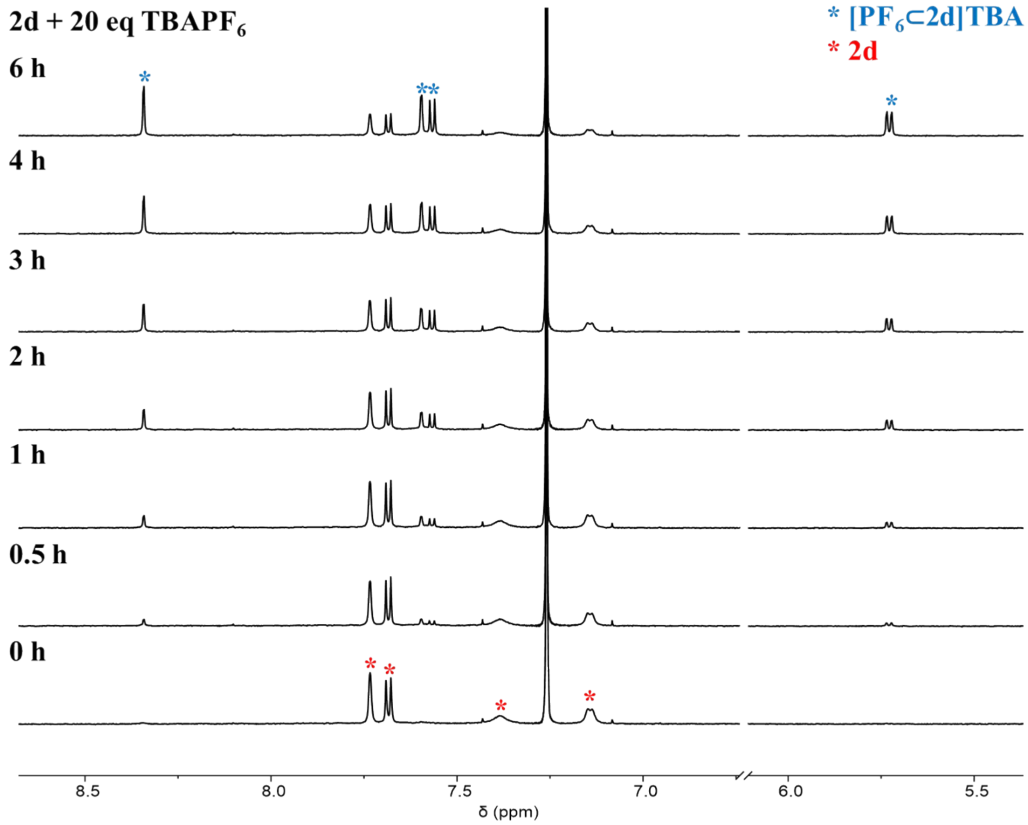

Figure S78. Partial ^{1}H NMR spectra (600 MHz, 298 K, $CDCl_3$) of a mixture of **2d** (0.3 mM) and $TBA^+ \cdot PF_6^-$ (6 mM), which were recorded for a certain amount of time including 0 (bottom) and 6 h (top). The resonances corresponding to the free cage and cage-anion complex were labelled with red and blue asterisks, respectively. The pseudo-first-order rate constant, k_{obs}, was calculated to be 16.2 × 10^{-2} h^{-1}, assuming that the concentration of free anion remained constant.

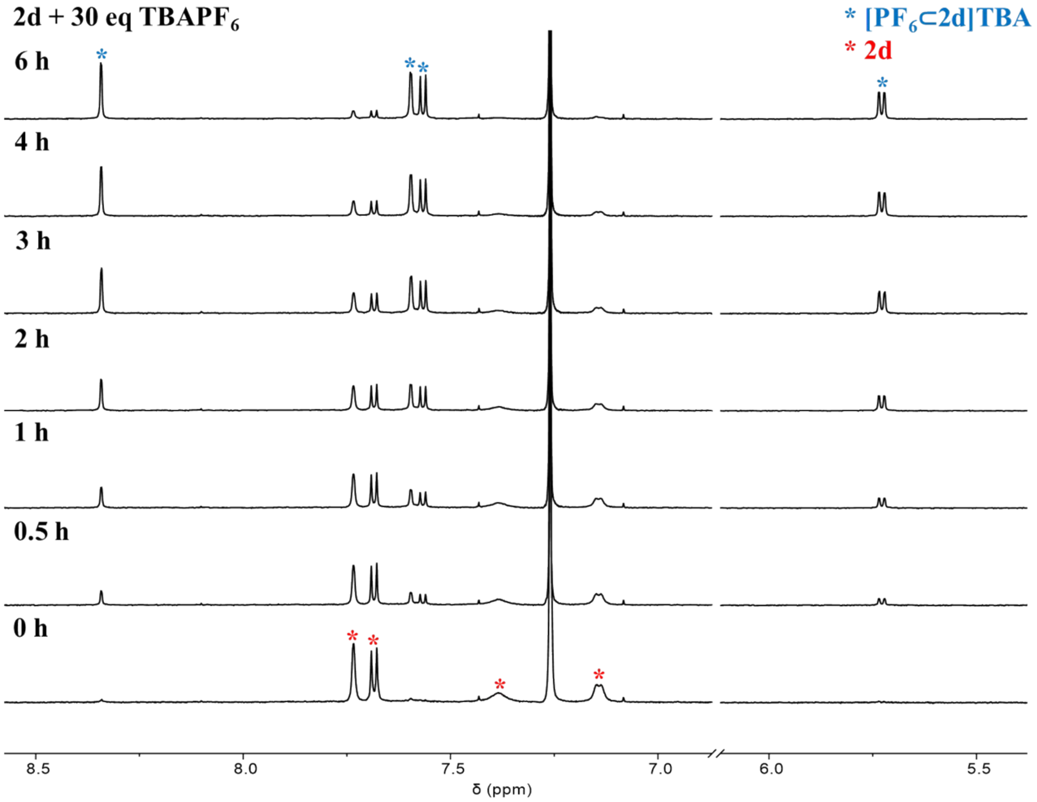

Figure S79. Partial ^{1}H NMR spectra (600 MHz, 298 K, $CDCl_3$) of a mixture of **2d** (0.3 mM) and $TBA^+ \cdot PF_6^-$ (9 mM), which were recorded for a certain amount of time including 0 (bottom) and 6 h (top). The resonances corresponding to the free cage and cage-anion complex were labelled with red and blue asterisks, respectively. The pseudo-first-order rate constant, k_{obs}, was calculated to be 29.8 × 10^{-2} h^{-1}, assuming that the concentration of free anion remained constant.

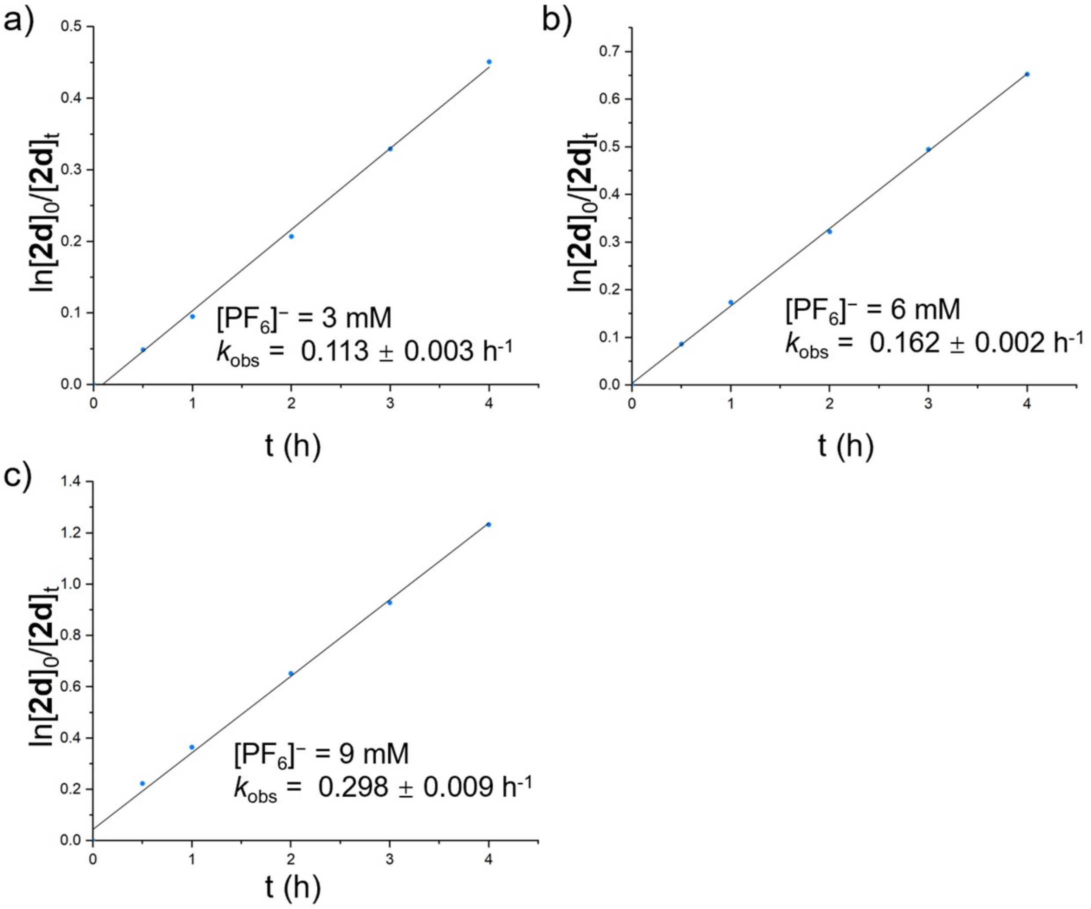

Figure S80. Plots of ln[**2d**]$_0$/[**2d**]$_t$ versus time (t) when the anion added is a) 3, b) 6 and c) 9 mM, based on the ^{1}H NMR spectroscopic results in Figure S76-S78. [**2d**]$_0$ and [**2d**]$_t$ are the relative concentration of cage **2d** in the beginning and a special time after addition of the anion, respectively, which was determined by integrating the corresponding resonances corresponding to the cage and complex respectively. Linear fitting was performed in each case, producing three straight lines. The rates of host-guest complexation namely k_{obs} in each case is the slope of the linear, by using the following equation ln[**2d**]$_0$/[**2d**]$_t$ = k_{obs}t.

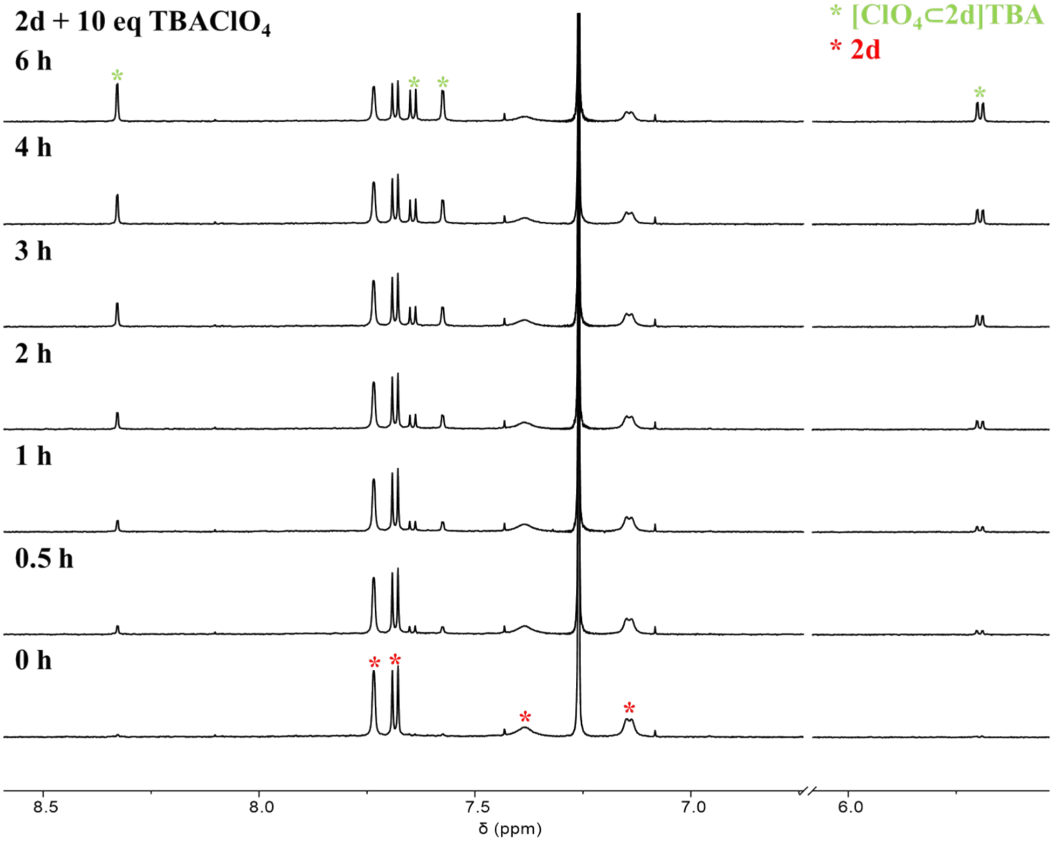

Figure S 81. Partial ^{1}H NMR spectra (600 MHz, 298 K, $CDCl_3$) of a mixture of **2d** (0.3 mM) and $TBA^+ \cdot ClO_4^-$ (3 mM), which were recorded for a certain amount of time including 0 (bottom) and 6 h (top). The resonances corresponding to the free cage and cage-anion complex were labelled with red and green asterisks, respectively. The pseudo-first-order rate constant, k_{obs}, was calculated to be 9.0×10^{-2} h^{-1}, assuming that the concentration of free anion remained constant.

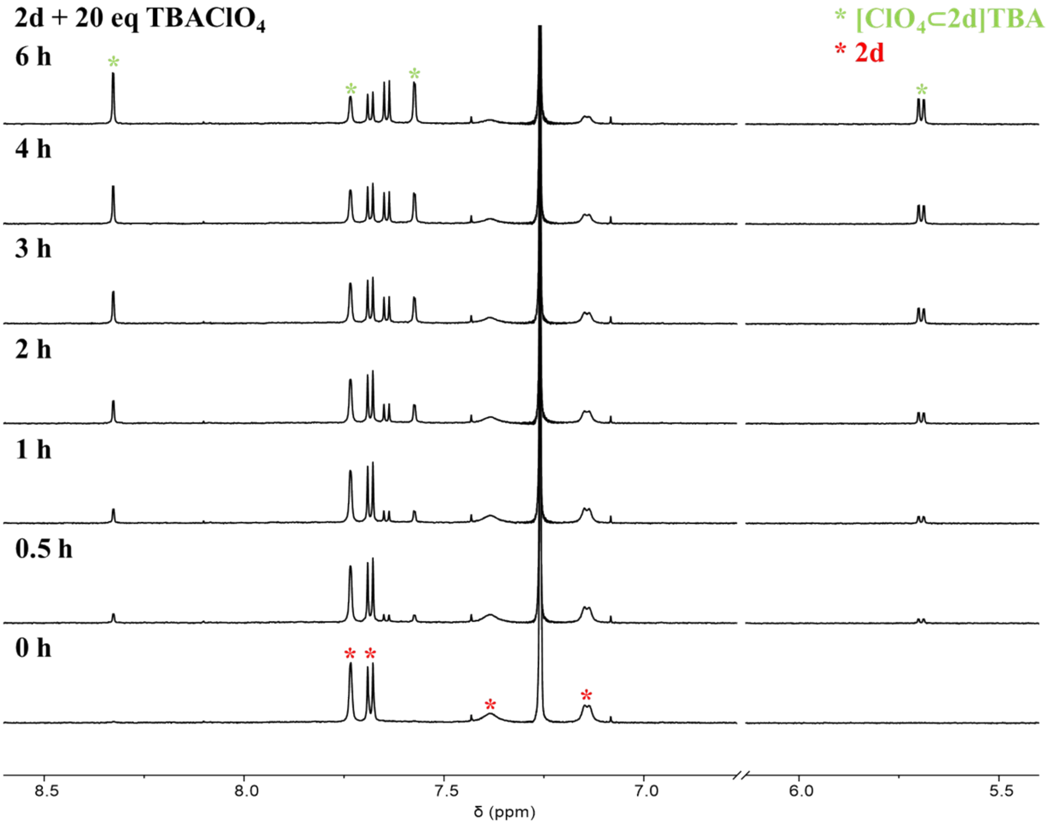

Figure S82. Partial ^{1}H NMR spectra (600 MHz, 298 K, $CDCl_3$) of a mixture of **2d** (0.3 mM) and $TBA^+ \cdot ClO_4^-$ (6 mM), which were recorded for a certain amount of time including 0 (bottom) and 6 h (top). The resonances corresponding to the free cage and cage-anion complex were labelled with red and green asterisks, respectively. The pseudo-first-order rate constant, k_{obs}, was calculated to be 14.1 × 10^{-2} h^{-1}, assuming that the concentration of free anion remained constant.

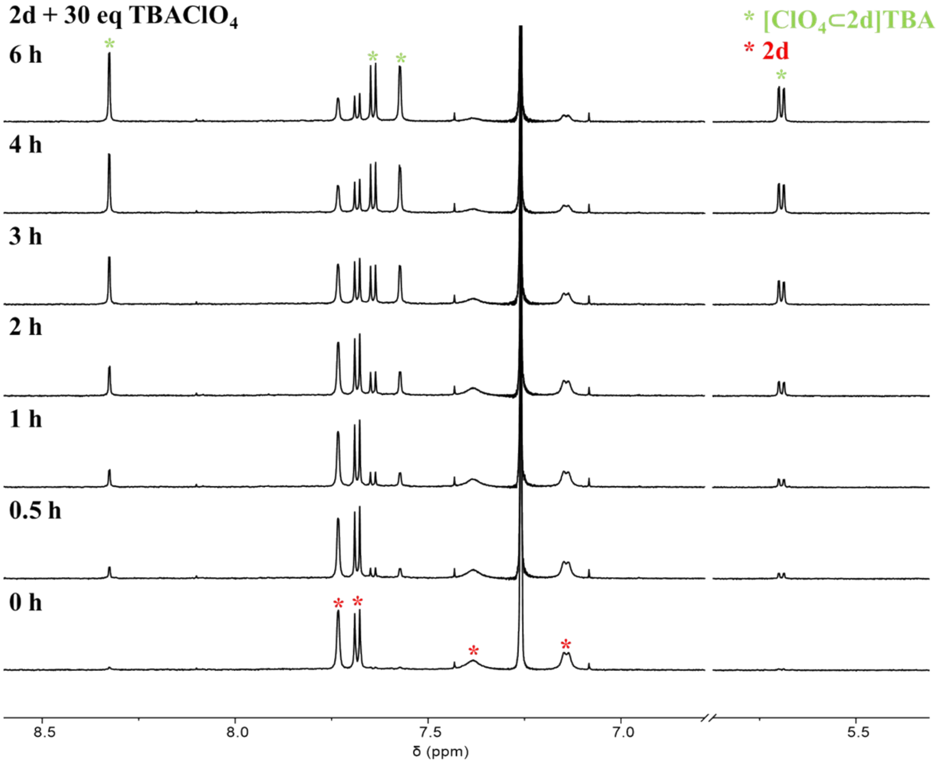

Figure S83. Partial ^{1}H NMR spectra (600 MHz, 298 K, $CDCl_3$) of a mixture of **2d** (0.3 mM) and $TBA^+ \cdot ClO_4^-$ (9 mM), which were recorded for a certain amount of time including 0 (bottom) and 6 h (top). The resonances corresponding to the free cage and cage-anion complex were labelled with red and green asterisks, respectively. The pseudo-first-order rate constant, k_{obs}, was calculated to be 20.0×10^{-2} h^{-1}, assuming that the concentration of free anion remained constant.

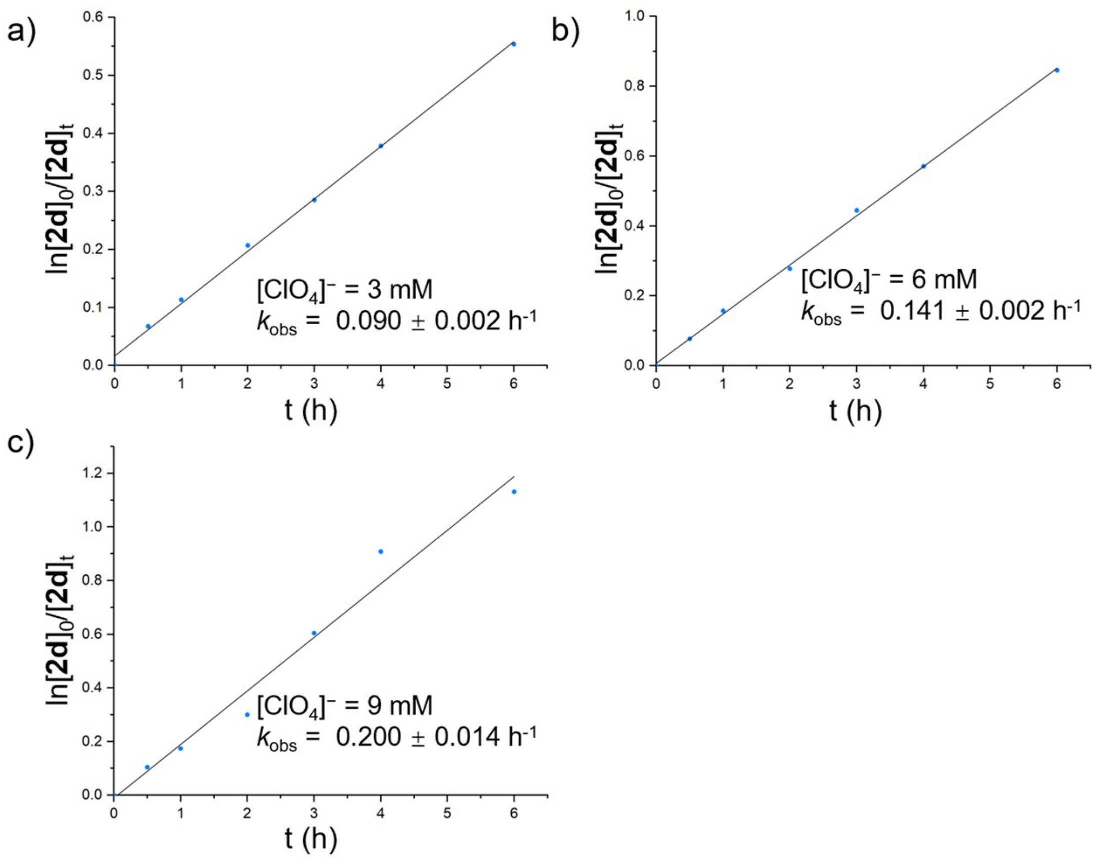

Figure S84. Plots of $\ln[\mathbf{2d}]_0/[\mathbf{2d}]_t$ versus time (t) when the anion added is a) 3, b) 6 and c) 9 mM, based on the ^{1}H NMR spectroscopic results in Figure S80-S82. $[\mathbf{2d}]_0$ and $[\mathbf{2d}]_t$ are the relative concentration of cage **2d** in the beginning and a special time after addition of the anion, respectively, which was determined by integrating the corresponding resonances corresponding to the cage and complex respectively. Linear fitting was performed in each case, producing three straight lines. The rates of host-guest complexation namely k_{obs} in each case is the slope of the linear, namely $\ln[\mathbf{2d}]_0/[\mathbf{2d}]_t = k_{obs}t$.

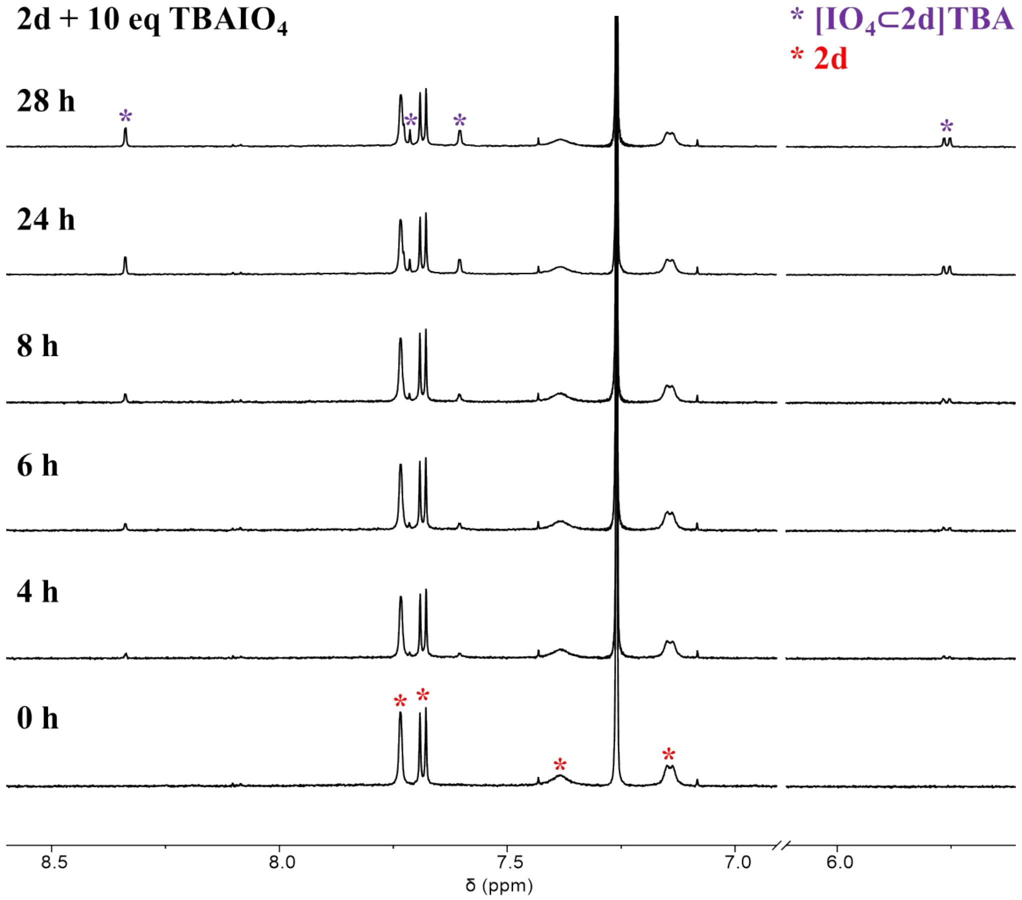

Figure S85. Partial ^{1}H NMR spectra (600 MHz, 298 K, $CDCl_3$) of a mixture of **2d** (0.3 mM) and $TBA^+ \cdot IO_4^-$ (3 mM), which were recorded for a certain amount of time including 0 (bottom) and 24 h (top). The resonances corresponding to the free cage and cage-anion complex were labelled with red and purple asterisks, respectively. The pseudo-first-order rate constant, k_{obs}, was calculated to be 8×10^{-3} h^{-1}, assuming that the concentration of free anion remained constant.

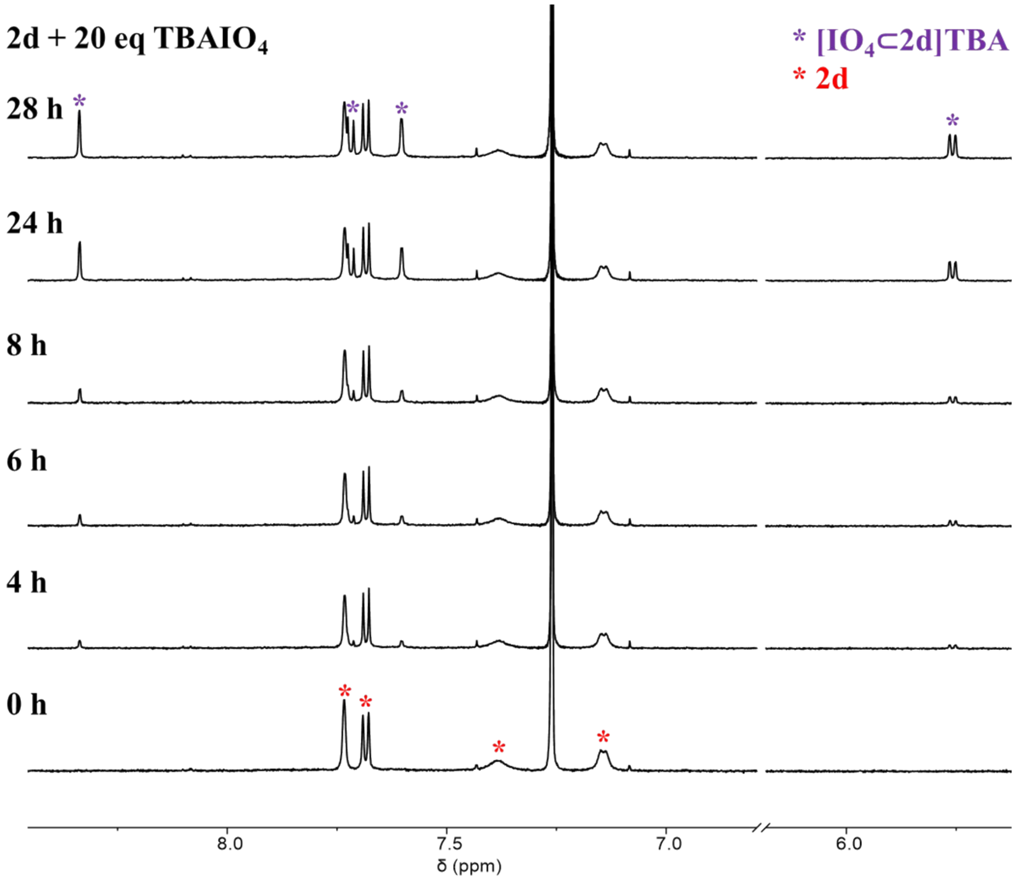

Figure S86. Partial ^{1}H NMR spectra (600 MHz, 298 K, $CDCl_3$) of a mixture of **2d** (0.3 mM) and $TBA^+ \cdot IO_4^-$ (6 mM), which were recorded for a certain amount of time including 0 (bottom) and 24 h (top). The resonances corresponding to the free cage and cage-anion complex were labelled with red and purple asterisks, respectively. The pseudo-first-order rate constant, k_{obs}, was calculated to be 18.3 × 10^{-3} h^{-1}, assuming that the concentration of free anion remained constant.

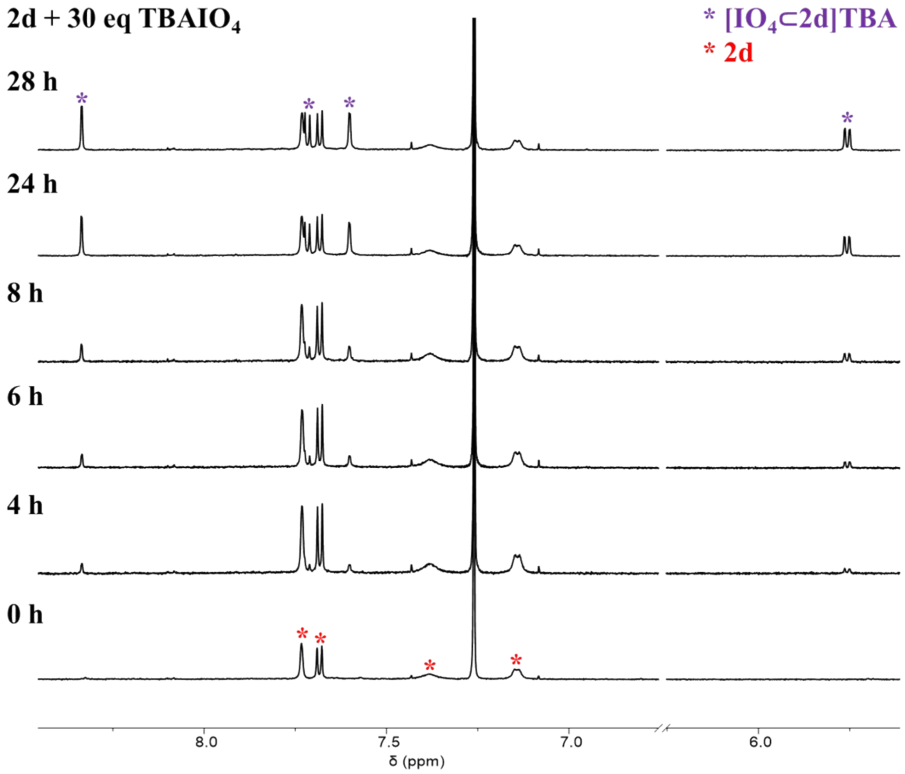

Figure S87. Partial ^{1}H NMR spectra (600 MHz, 298 K, $CDCl_3$) of a mixture of **2d** (0.3 mM) and $TBA^{+}\cdot IO_4^{-}$ (9 mM), which were recorded for a certain amount of time including 0 (bottom) and 24 h (top). The resonances corresponding to the free cage and cage-anion complex were labelled with red and purple asterisks, respectively. The pseudo-first-order rate constant, k_{obs}, was calculated to be 22×10^{-3} h^{-1}, assuming that the concentration of free anion remained constant.

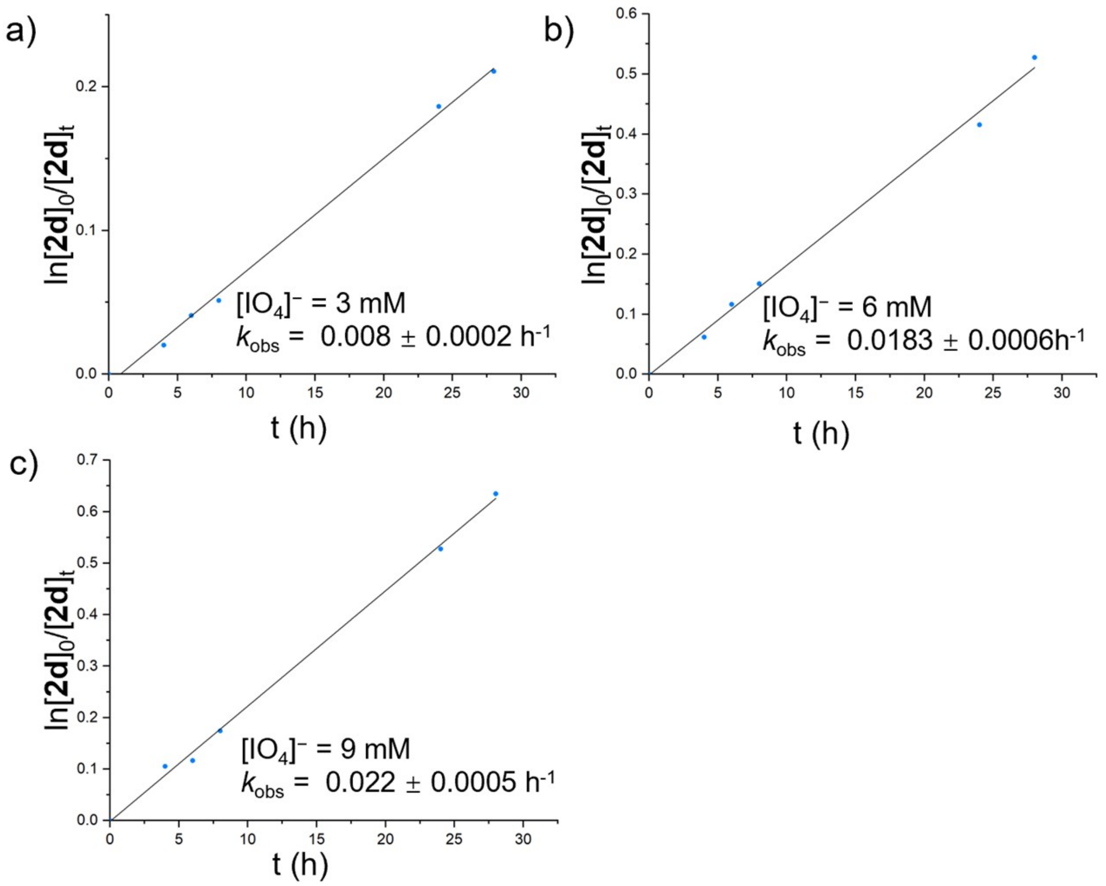

Figure S88. Plots of ln[**2d**]$_0$/[**2d**]$_t$ versus time (t) when the anion added is a) 3, b) 6 and c) 9 mM, based on the ^{1}H NMR spectroscopic results in Figure S84-S86. [**2d**]$_0$ and [**2d**]$_t$ are the relative concentration of cage **2d** in the beginning and a special time after addition of the anion, respectively, which was determined by integrating the corresponding resonances corresponding to the cage and complex respectively. Linear fitting was performed in each case, producing three straight lines. The rates of host-guest complexation namely k_{obs} in each case is the slope of the linear, namely ln[**2d**]$_0$/[**2d**]$_t$ = k_{obs}t.

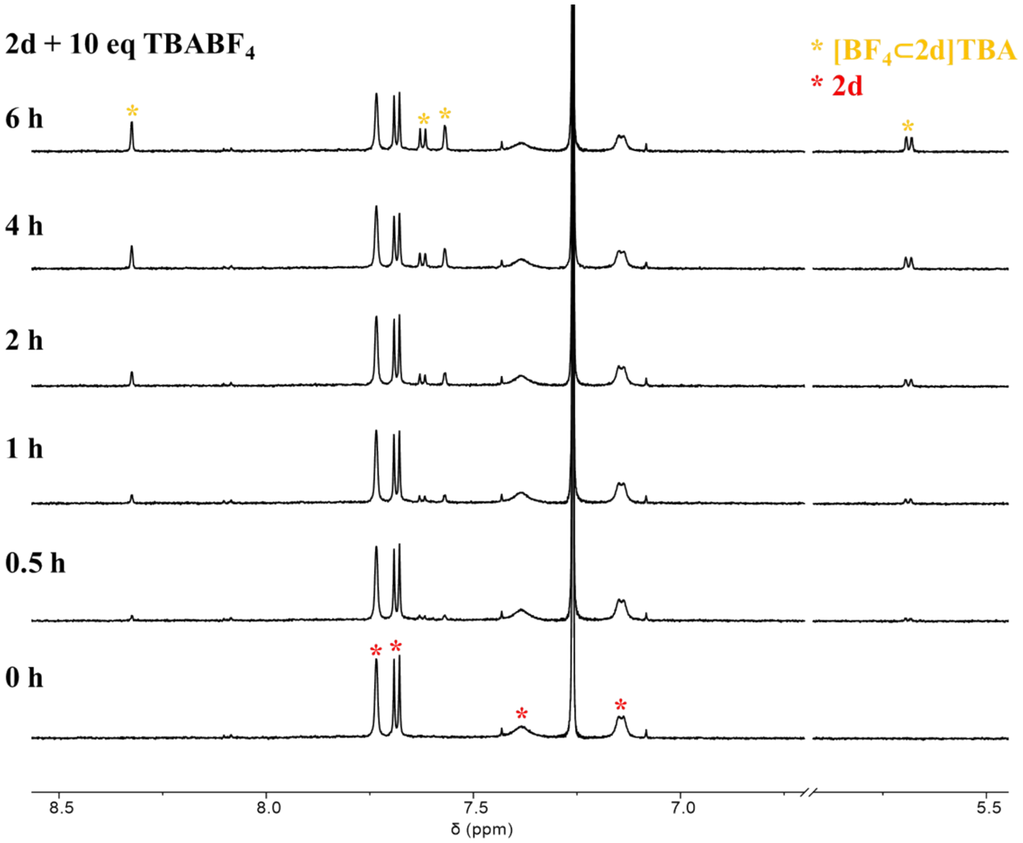

Figure S89. Partial ^{1}H NMR spectra (600 MHz, 298 K, $CDCl_3$) of a mixture of **2d** (0.3 mM) and $TBA^+{\cdot}BF_4^-$ (3 mM), which were recorded for a certain amount of time including 0 (bottom) and 6 h (top). The resonances corresponding to the free cage and cage-anion complex were labelled with red and yellow asterisks, respectively. The pseudo-first-order rate constant, k_{obs}, was calculated to be 8.1×10^{-2} h^{-1}, assuming that the concentration of free anion remained constant.

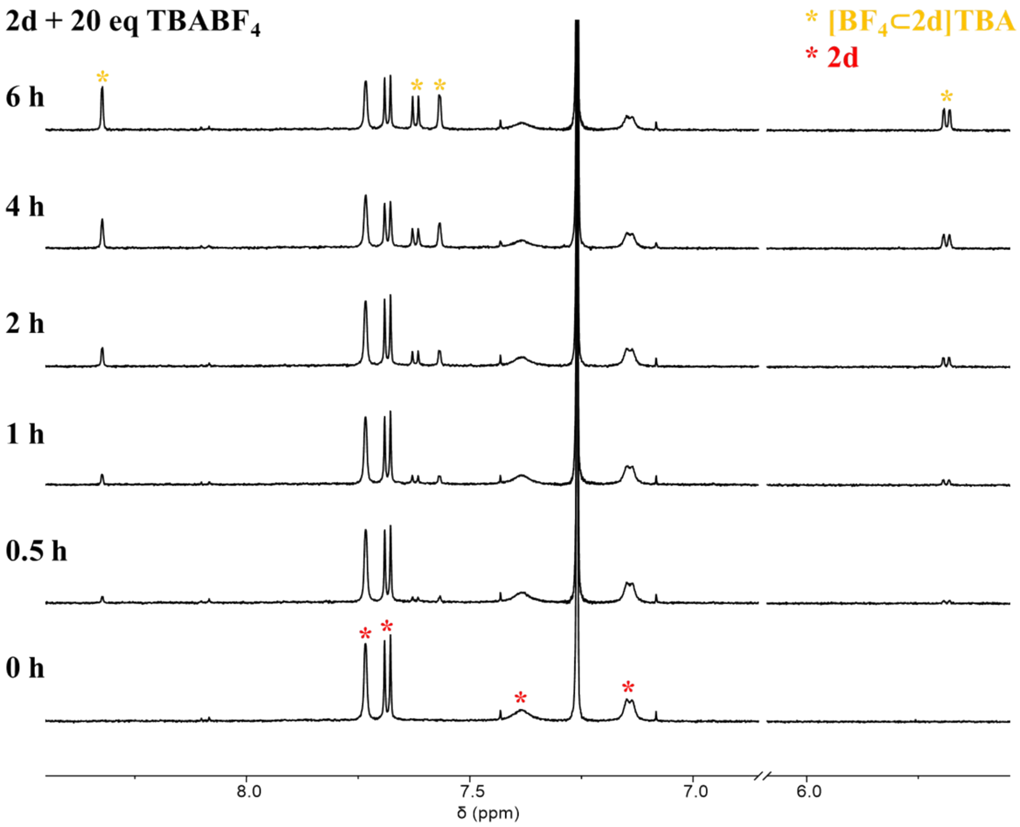

Figure S90. Partial ^{1}H NMR spectra (600 MHz, 298 K, $CDCl_3$) of a mixture of **2d** (0.3 mM) and $TBA^+ \cdot BF_4^-$ (6 mM), which were recorded for a certain amount of time including 0 (bottom) and 6 h (top). The resonances corresponding to the free cage and cage-anion complex were labelled with red and yellow asterisks, respectively. The pseudo-first-order rate constant, k_{obs}, was calculated to be 8.1×10^{-2} h^{-1}, assuming that the concentration of free anion remained constant.

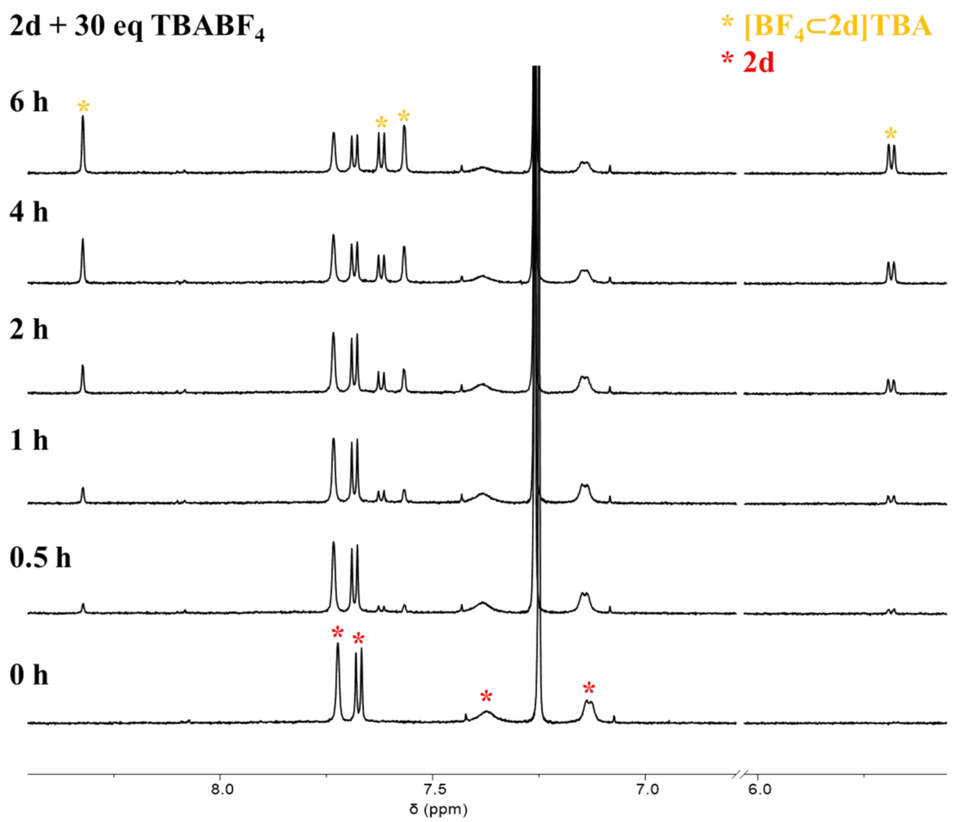

Figure S91. Partial ^{1}H NMR spectra (600 MHz, 298 K, $CDCl_3$) of a mixture of **2d** (0.3 mM) and $TBA^+ \cdot BF_4^-$ (9 mM), which were recorded for a certain amount of time including 0 (bottom) and 6 h (top). The resonances corresponding to the free cage and cage-anion complex were labelled with red and yellow asterisks, respectively. The pseudo-first-order rate constant, k_{obs}, was calculated to be 11.9×10^{-2} h^{-1}, assuming that the concentration of free anion remained constant.

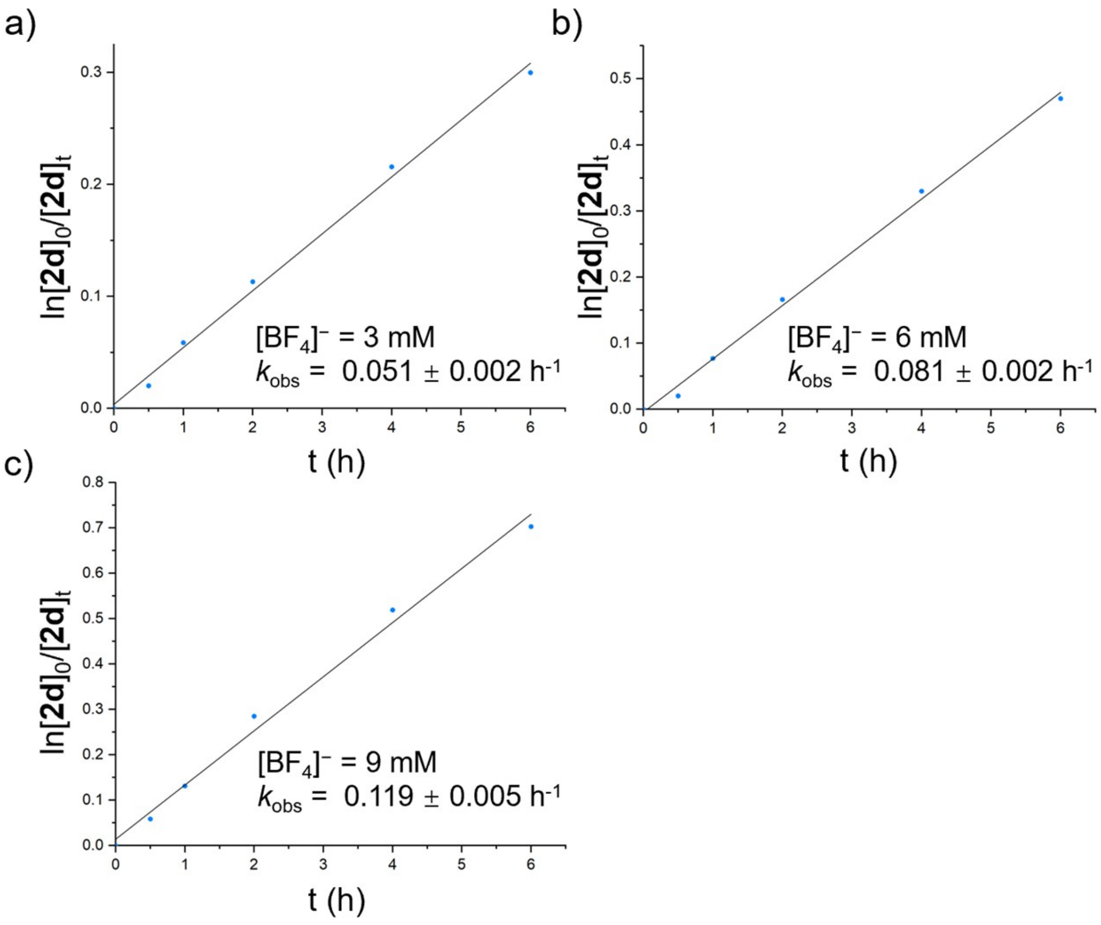

Figure S92. Plots of $\ln[\mathbf{2d}]_0/[\mathbf{2d}]_t$ versus time (t) when the anion added is a) 3, b) 6 and c) 9 mM, based on the ^{1}H NMR spectroscopic results in Figure S88-S90. $[\mathbf{2d}]_0$ and $[\mathbf{2d}]_t$ are the relative concentration of cage **2d** in the beginning and a special time after addition of the anion, respectively, which was determined by integrating the corresponding resonances corresponding to the cage and complex respectively. Linear fitting was performed in each case, producing three straight lines. The rates of host-guest complexation namely k_{obs} in each case is the slope of the linear, namely $\ln[\mathbf{2d}]_0/[\mathbf{2d}]_t = k_{obs}t$.

5.6 Kinetic studies of 2f to recognize anions

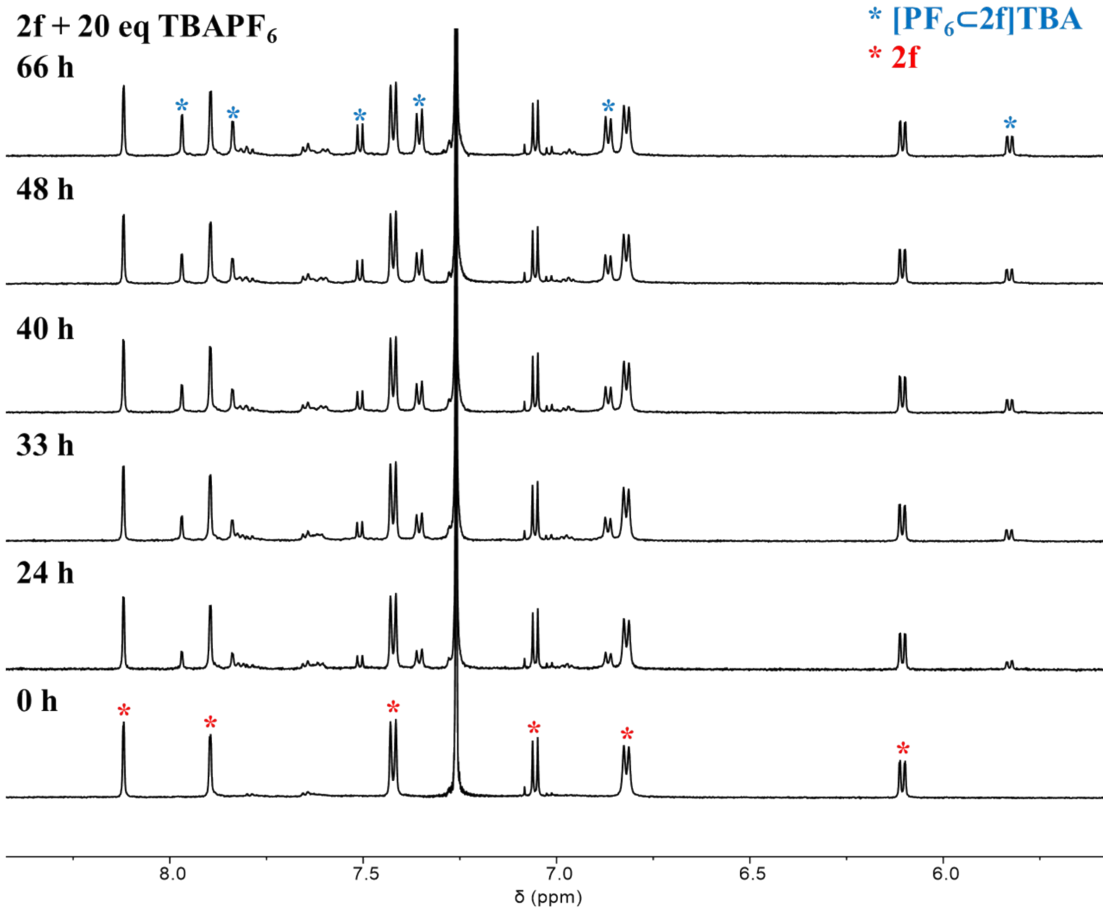

Figure S93. Partial ^{1}H NMR spectra (600 MHz, 298 K, $CDCl_3$) of a mixture of **2f** (0.15 mM) and $TBA^+ \cdot PF_6^-$ (3 mM), which were recorded for a certain amount of time including 0 (bottom) and 66 h (top). The resonances corresponding to the free cage and cage-anion complex were labelled with red and blue asterisks, respectively. The pseudo-first-order rate constant, k_{obs}, was calculated to be 8.14×10^{-3} h^{-1}, assuming that the concentration of free anion remained constant.

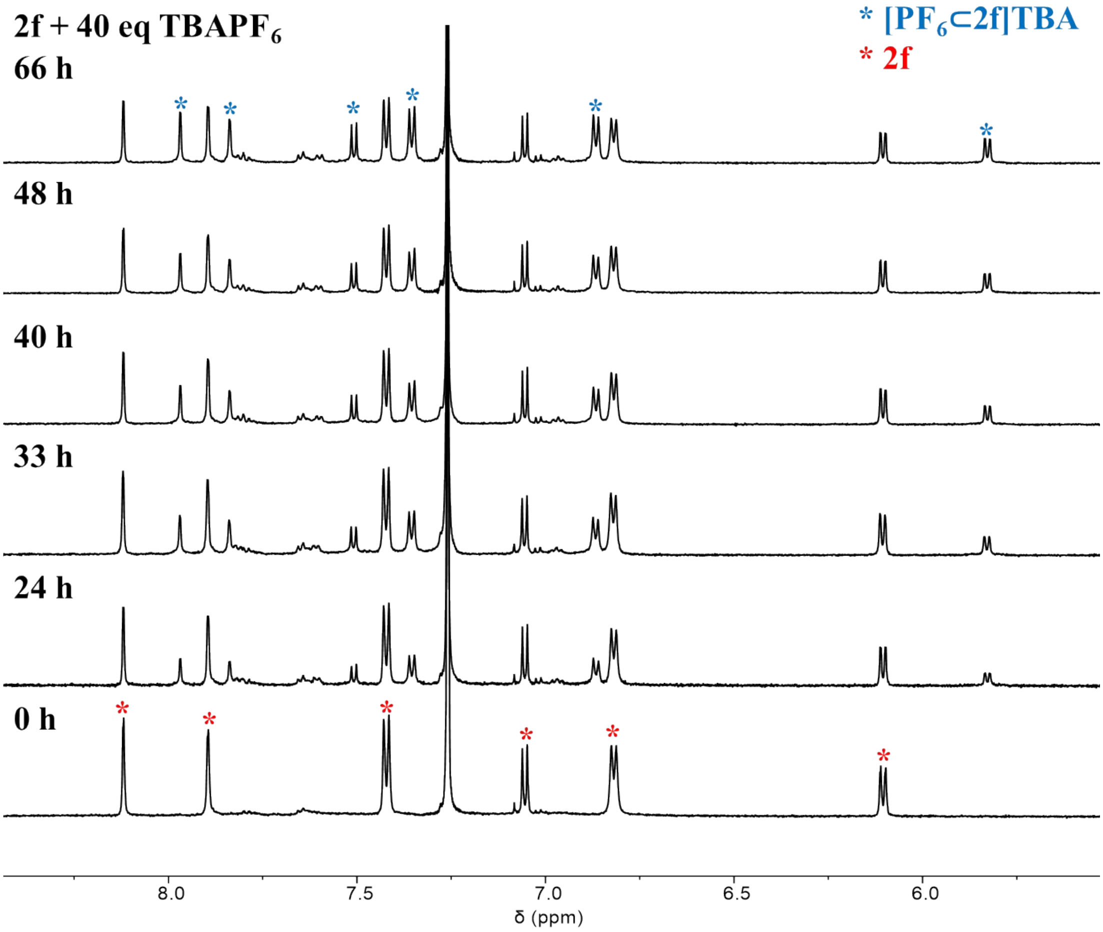

Figure S94. Partial ^{1}H NMR spectra (600 MHz, 298 K, $CDCl_3$) of a mixture of **2f** (0.15 mM) and $TBA^+ \cdot PF_6^-$ (6 mM), which were recorded for a certain amount of time including 0 (bottom) and 66 h (top). The resonances corresponding to the free cage and cage-anion complex were labelled with red and blue asterisks, respectively. The pseudo-first-order rate constant, k_{obs}, was calculated to be 10.68 × 10^{-3} h^{-1}, assuming that the concentration of free anion remained constant.

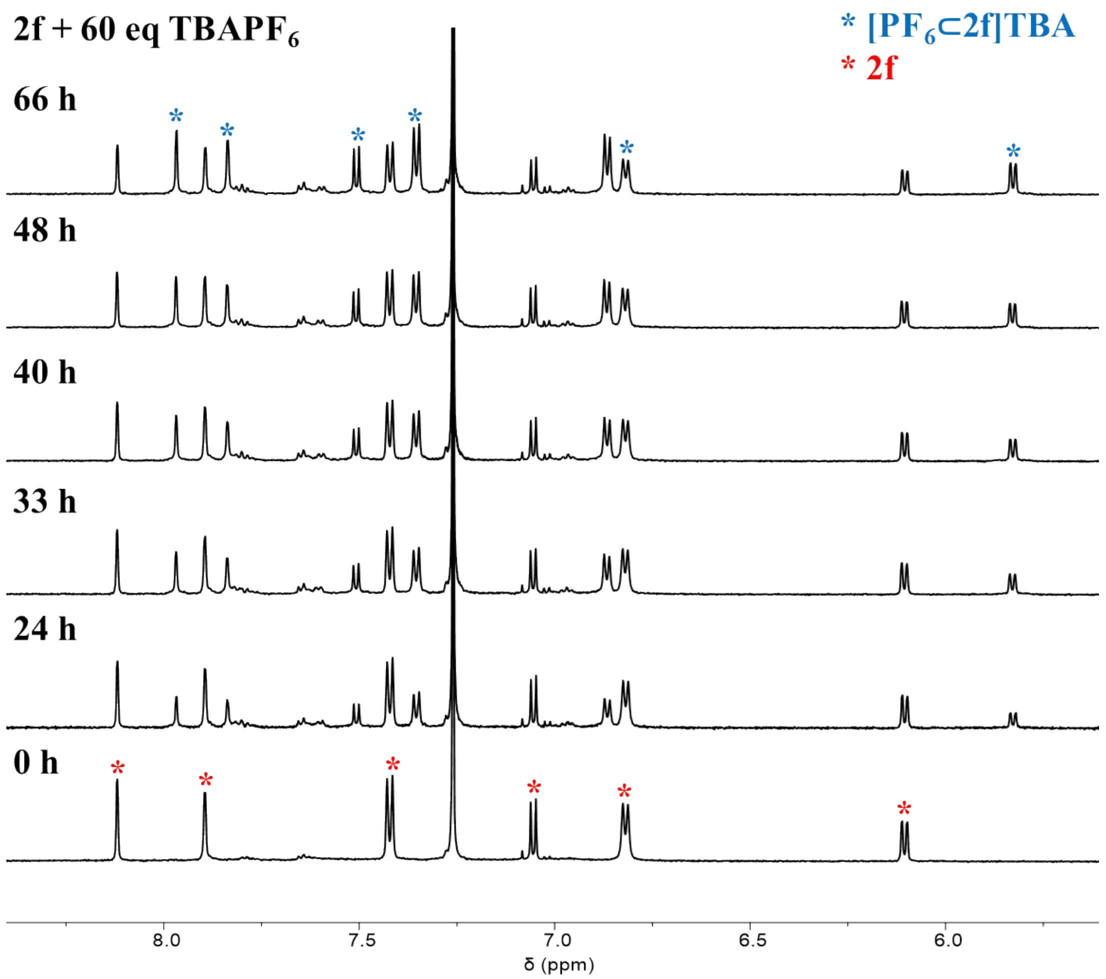

Figure S95. Partial ^{1}H NMR spectra (600 MHz, 298 K, $CDCl_3$) of a mixture of **2f** (0.15 mM) and $TBA^{+}\cdot PF_6^{-}$ (9 mM), which were recorded for a certain amount of time including 0 (bottom) and 66 h (top). The resonances corresponding to the free cage and cage-anion complex were labelled with red and blue asterisks, respectively. The pseudo-first-order rate constant, k_{obs}, was calculated to be 14.48×10^{-3} h^{-1}, assuming that the concentration of free anion remained constant.

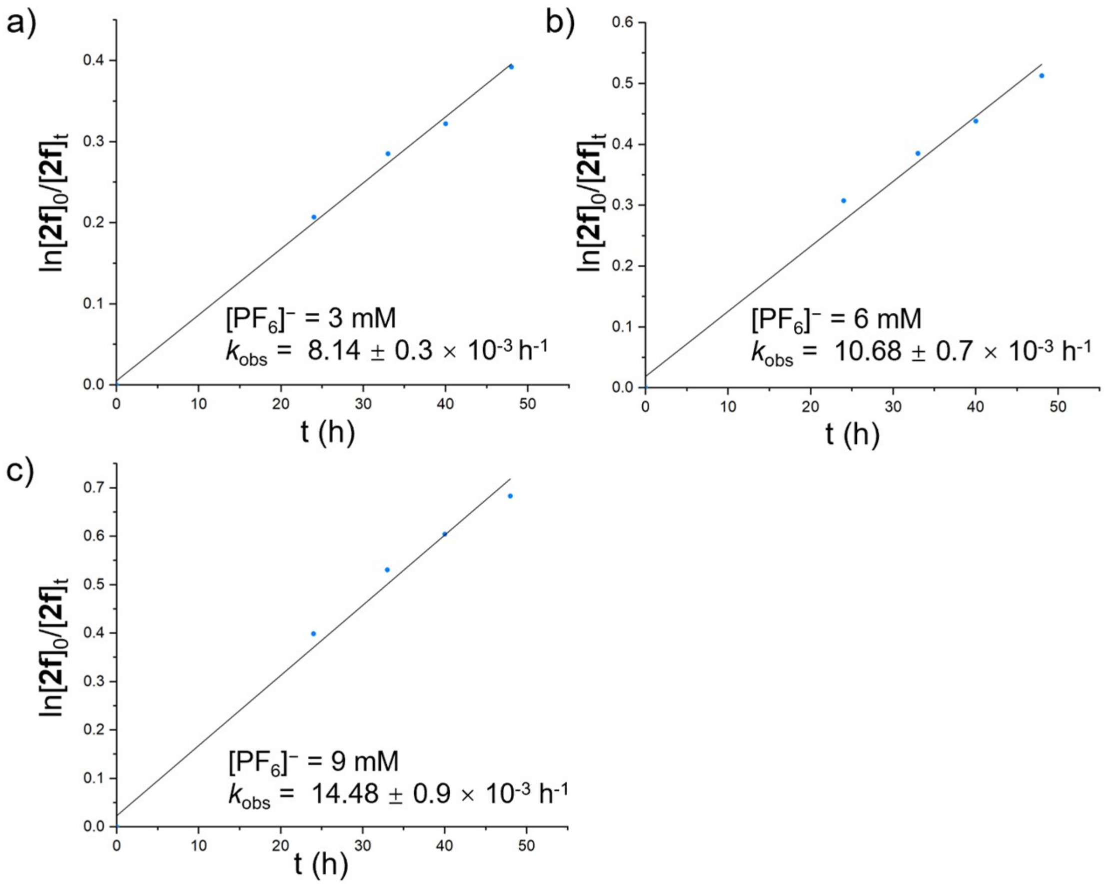

Figure S96. Plots of $\ln[\mathbf{2f}]_0/[\mathbf{2f}]_t$ versus time (t) when the anion added is a) 3, b) 6 and c) 9 mM, based on the ^{1}H NMR spectroscopic results in Figure S92-S94. $[\mathbf{2f}]_0$ and $[\mathbf{2f}]_t$ are the relative concentration of cage **2f** in the beginning and a special time after addition of the anion, respectively, which was determined by integrating the corresponding resonances corresponding to the cage and complex respectively. Linear fitting was performed in each case, producing three straight lines. The rates of host-guest complexation namely k_{obs} in each case is the slope of the linear, namely $\ln[\mathbf{2f}]_0/[\mathbf{2f}]_t = k_{obs}t$.

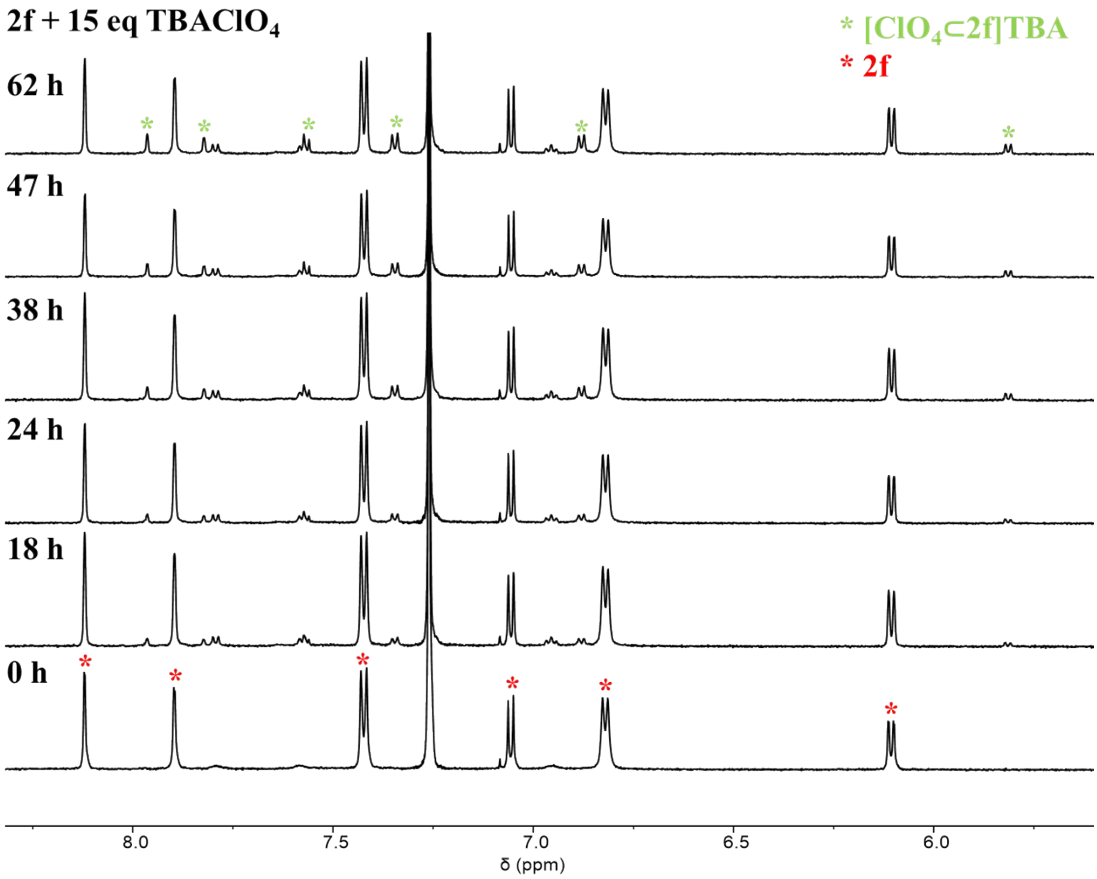

Figure S97. Partial ^{1}H NMR spectra (600 MHz, 298 K, $CDCl_3$) of a mixture of **2f** (0.2 mM) and $TBA^{+}\cdot ClO_4^{-}$ (3 mM), which were recorded for a certain amount of time including 0 (bottom) and 62 h (top). The resonances corresponding to the free cage and cage-anion complex were labelled with red and green asterisks, respectively. The pseudo-first-order rate constant, k_{obs}, was calculated to be 2.72×10^{-3} h^{-1}, assuming that the concentration of free anion remained constant.

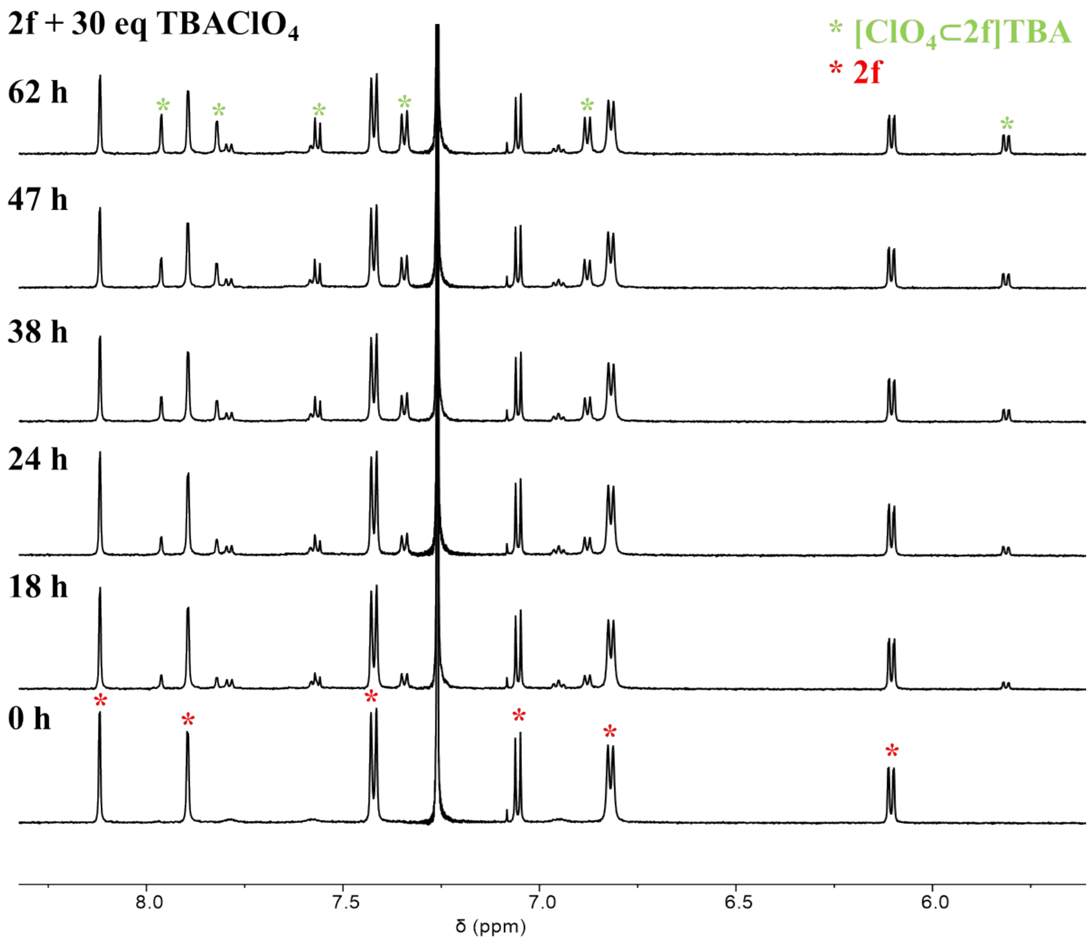

Figure S98. Partial ^{1}H NMR spectra (600 MHz, 298 K, $CDCl_3$) of a mixture of **2f** (0.2 mM) and $TBA^{+}\cdot ClO_4^{-}$ (6 mM), which were recorded for a certain amount of time including 0 (bottom) and 62 h (top). The resonances corresponding to the free cage and cage-anion complex were labelled with red and green asterisks, respectively. The pseudo-first-order rate constant, k_{obs}, was calculated to be 6.64×10^{-3} h^{-1}, assuming that the concentration of free anion remained constant.

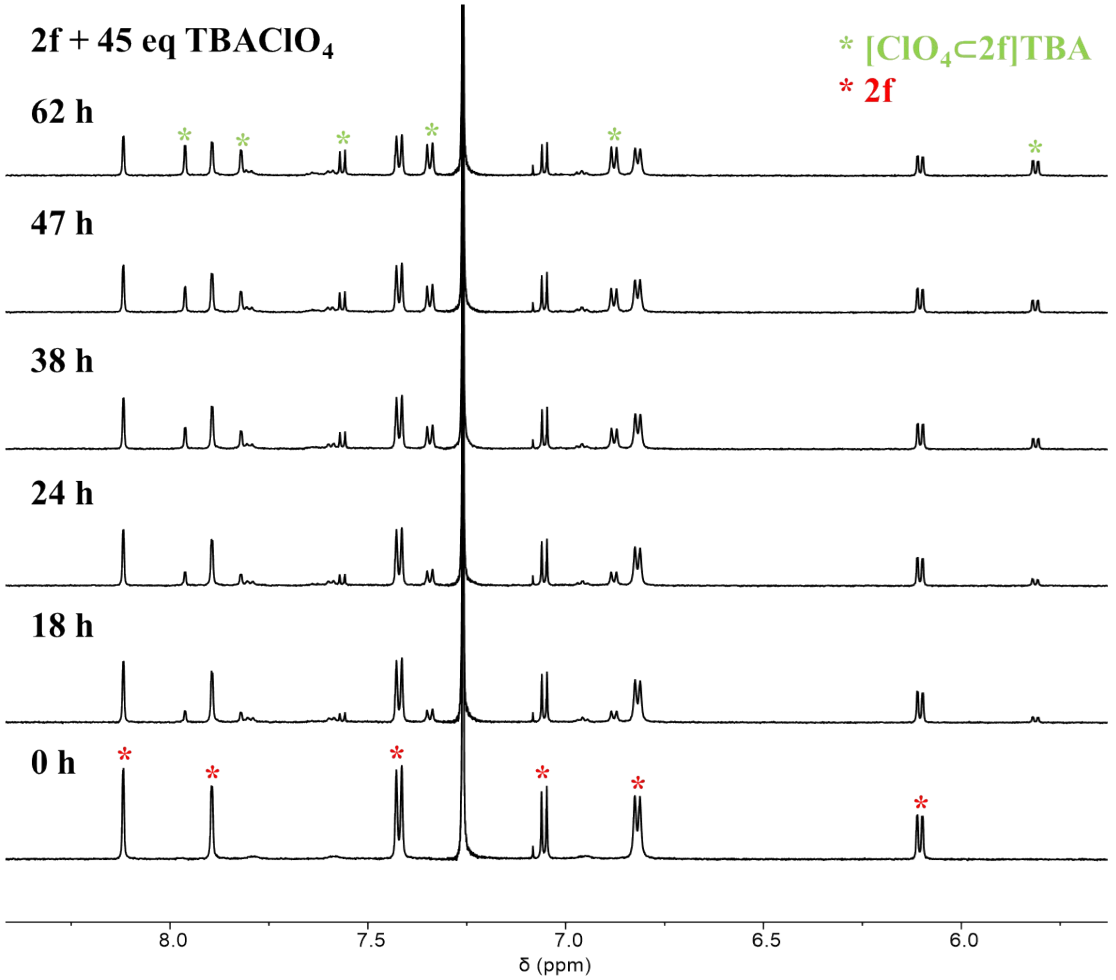

Figure S99. Partial ^{1}H NMR spectra (600 MHz, 298 K, $CDCl_3$) of a mixture of **2f** (0.2 mM) and $TBA^+ \cdot ClO_4^-$ (9 mM), which were recorded for a certain amount of time including 0 (bottom) and 62 h (top). The resonances corresponding to the free cage and cage-anion complex were labelled with red and green asterisks, respectively. The pseudo-first-order rate constant, k_{obs}, was calculated to be 9.36×10^{-3} h^{-1}, assuming that the concentration of free anion remained constant.

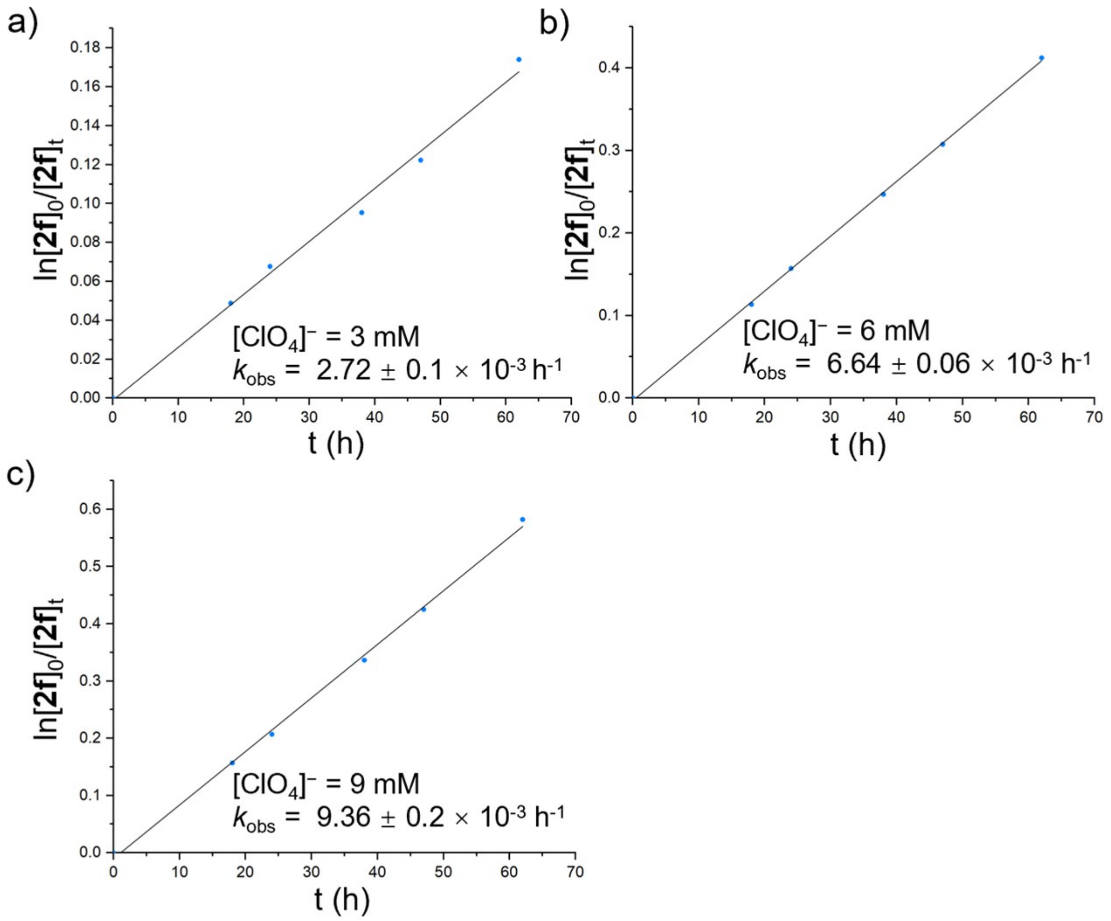

Figure S100. Plots of ln[**2f**]$_0$/[**2f**]$_t$ versus time (t) when the anion added is a) 3, b) 6 and c) 9 mM, based on the ^{1}H NMR spectroscopic results in Figure S96-S98. [**2f**]$_0$ and [**2f**]$_t$ are the relative concentration of cage **2f** in the beginning and a special time after addition of the anion, respectively, which was determined by integrating the corresponding resonances corresponding to the cage and complex respectively. Linear fitting was performed in each case, producing three straight lines. The rates of host-guest complexation namely k_{obs} in each case is the slope of the linear, namely ln[**2f**]$_0$/[**2f**]$_t$ = k_{obs}t.

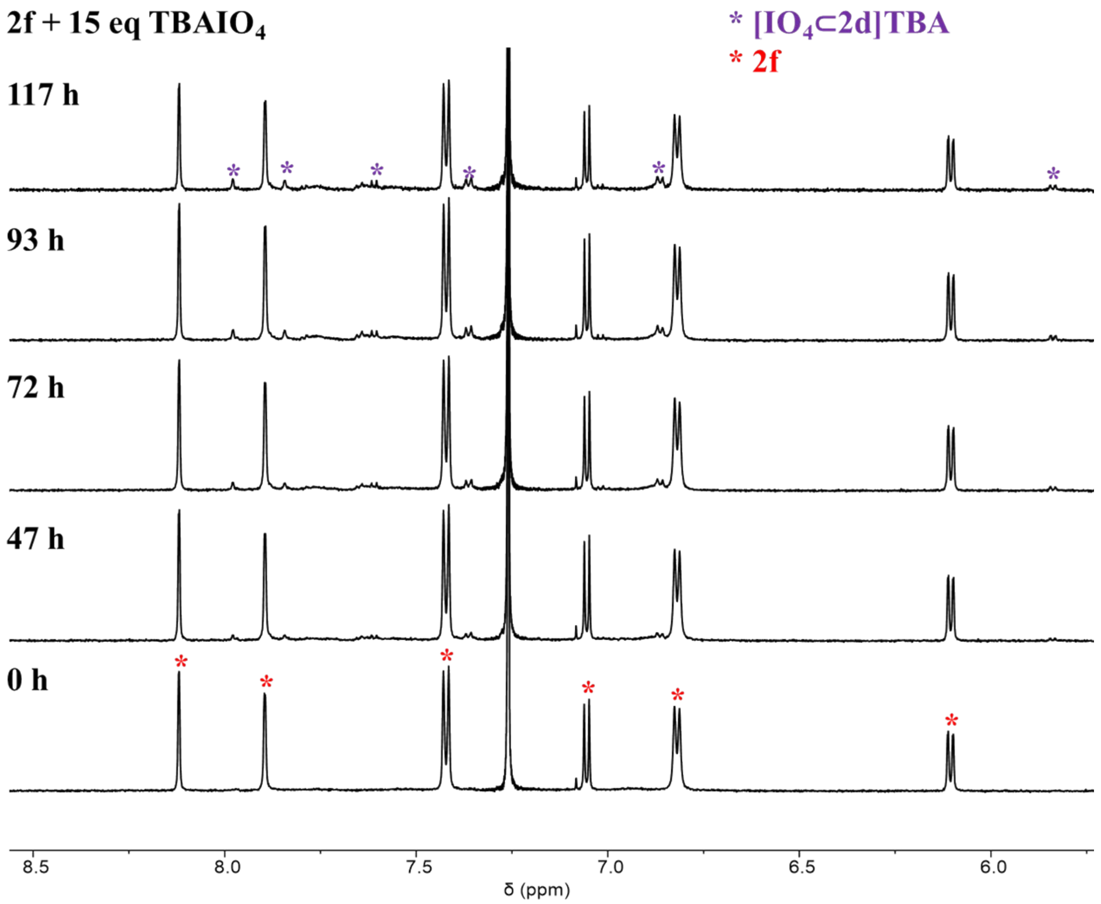

Figure S101. Partial ^{1}H NMR spectra (600 MHz, 298 K, $CDCl_3$) of a mixture of **2f** (0.2 mM) and $TBA^+ \cdot IO_4^-$ (3 mM), which were recorded for a certain amount of time including 0 (bottom) and 117 h (top). The resonances corresponding to the free cage and cage-anion complex were labelled with red and purple asterisks, respectively. The pseudo-first-order rate constant, k_{obs}, was calculated to be 0.6 × 10^{-3} h^{-1}, assuming that the concentration of free anion remained constant.

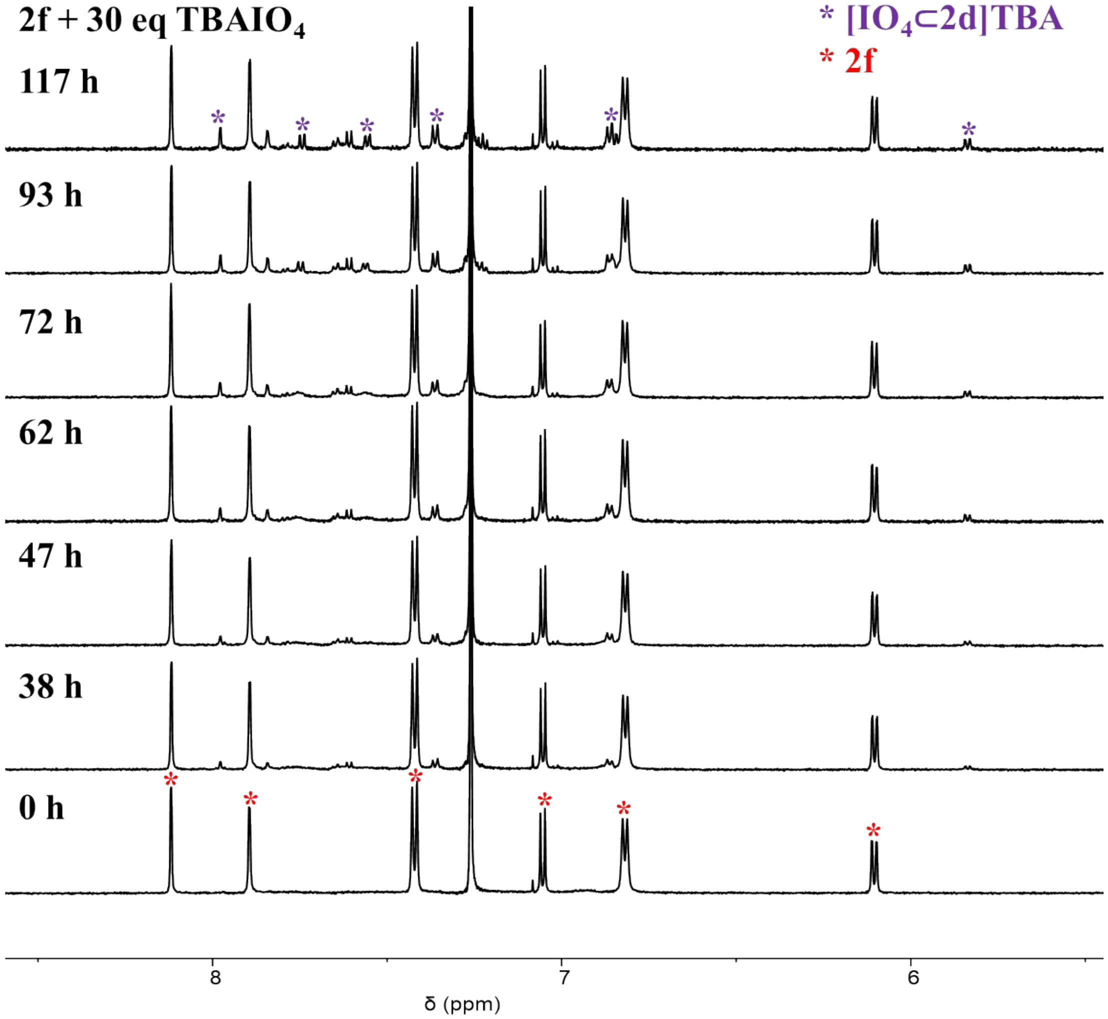

Figure S102. Partial ^{1}H NMR spectra (600 MHz, 298 K, $CDCl_3$) of a mixture of **2f** (0.2 mM) and $TBA^+ \cdot IO_4^-$ (6 mM), which were recorded for a certain amount of time including 0 (bottom) and 117 h (top). The resonances corresponding to the free cage and cage-anion complex were labelled with red and purple asterisks, respectively. The pseudo-first-order rate constant, k_{obs}, was calculated to be 1.6×10^{-3} h^{-1}, assuming that the concentration of free anion remained constant.

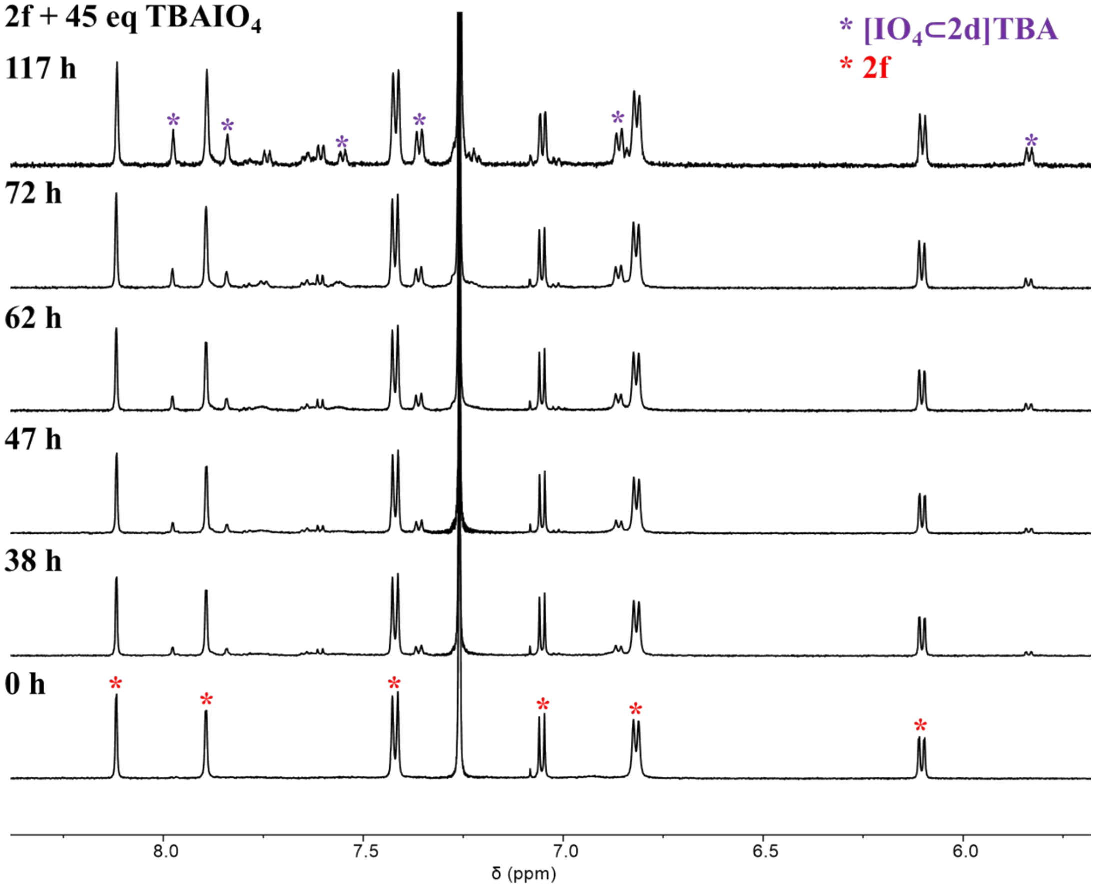

Figure S103. Partial ^{1}H NMR spectra (600 MHz, 298 K, $CDCl_3$) of a mixture of **2f** (0.2 mM) and $TBA^{+}\cdot IO_4^{-}$ (9 mM), which were recorded for a certain amount of time including 0 (bottom) and 117 h (top). The resonances corresponding to the free cage and cage-anion complex were labelled with red and purple asterisks, respectively. The pseudo-first-order rate constant, k_{obs}, was calculated to be 2.6×10^{-3} h^{-1}, assuming that the concentration of free anion remained constant.

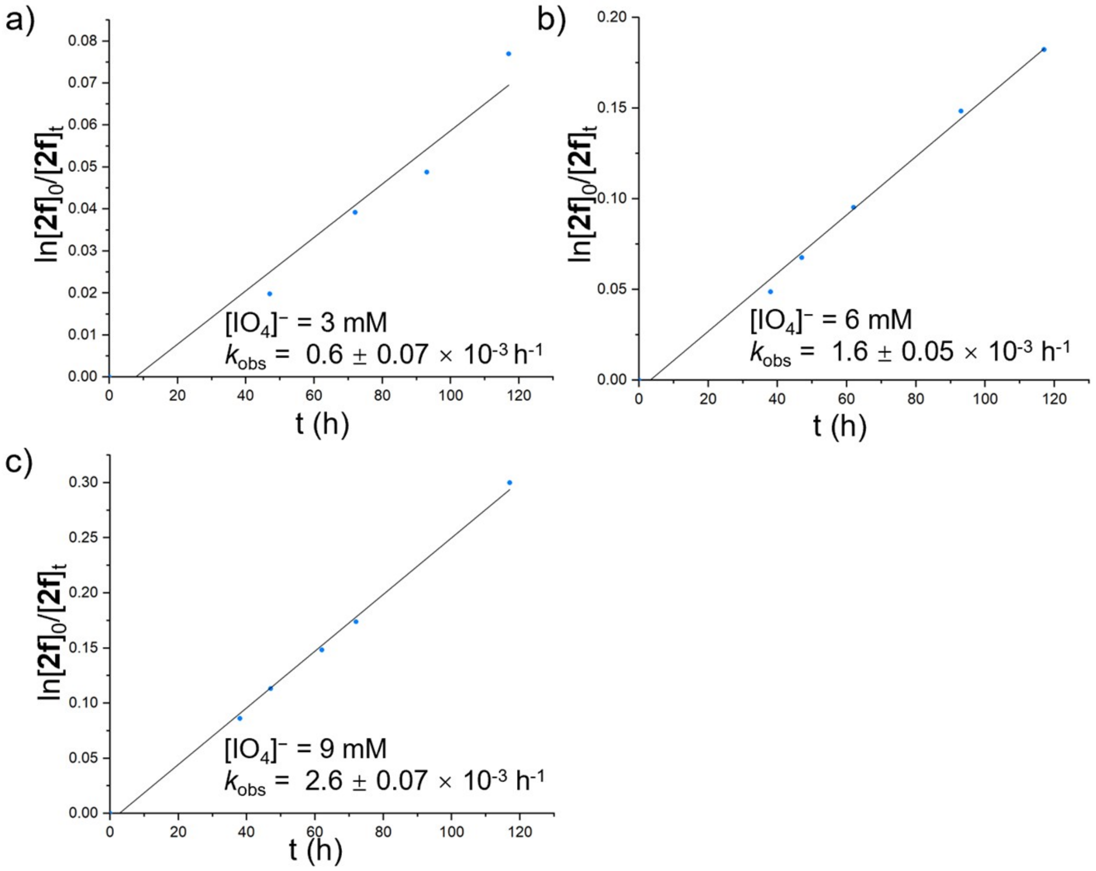

Figure S104. Plots of $\ln[\mathbf{2f}]_0/[\mathbf{2f}]_t$ versus time (t) when the anion added is a) 3, b) 6 and c) 9 mM, based on the ^{1}H NMR spectroscopic results in Figure S100-S102. $[\mathbf{2t}]_0$ and $[\mathbf{2f}]_t$ are the relative concentration of cage **2f** in the beginning and a special time after addition of the anion, respectively, which was determined by integrating the corresponding resonances corresponding to the cage and complex respectively. Linear fitting was performed in each case, producing three straight lines. The rates of host-guest complexation namely k_{obs} in each case is the slope of the linear, namely $\ln[\mathbf{2f}]_0/[\mathbf{2f}]_t = k_{obs}t$.

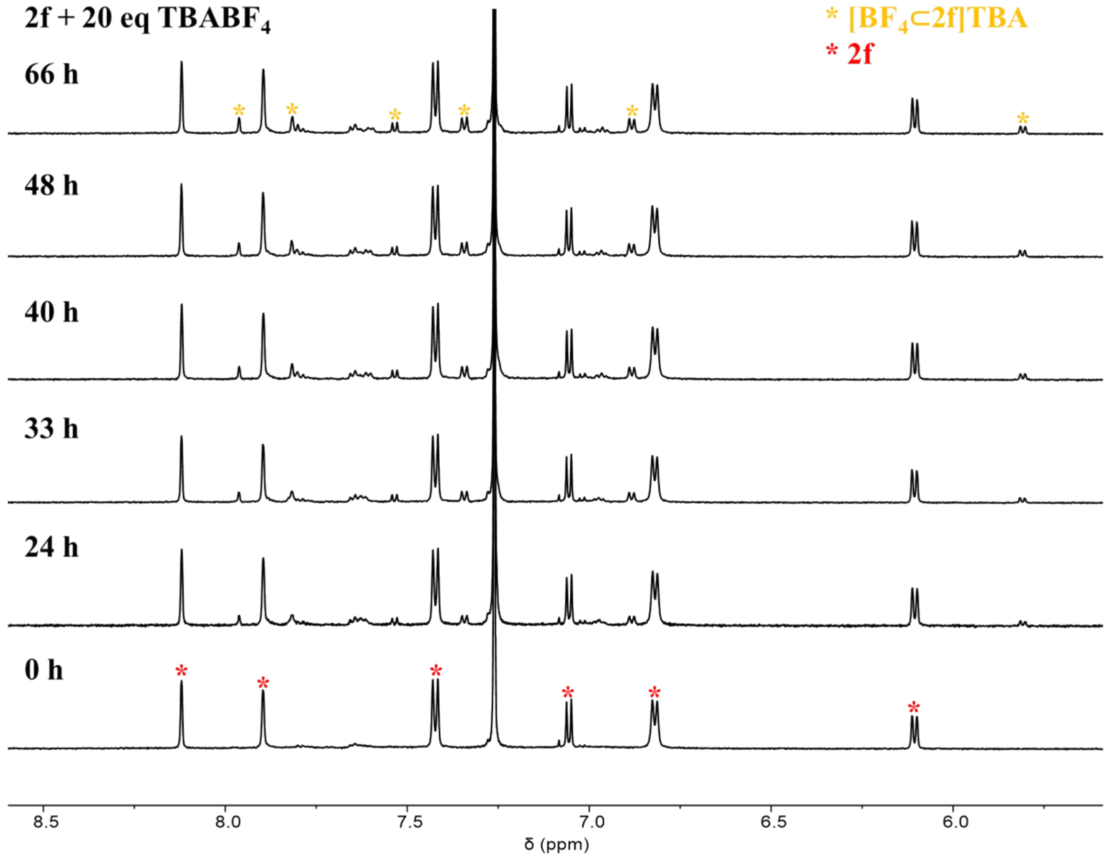

Figure S105. Partial ^{1}H NMR spectra (600 MHz, 298 K, $CDCl_3$) of a mixture of **2f** (0.15 mM) and $TBA^{+}\cdot BF_4^{-}$ (3 mM), which were recorded for a certain amount of time including 0 (bottom) and 66 h (top). The resonances corresponding to the free cage and cage-anion complex were labelled with red and yellow asterisks, respectively. The pseudo-first-order rate constant, k_{obs}, was calculated to be 3.24×10^{-3} h^{-1}, assuming that the concentration of free anion remained constant.

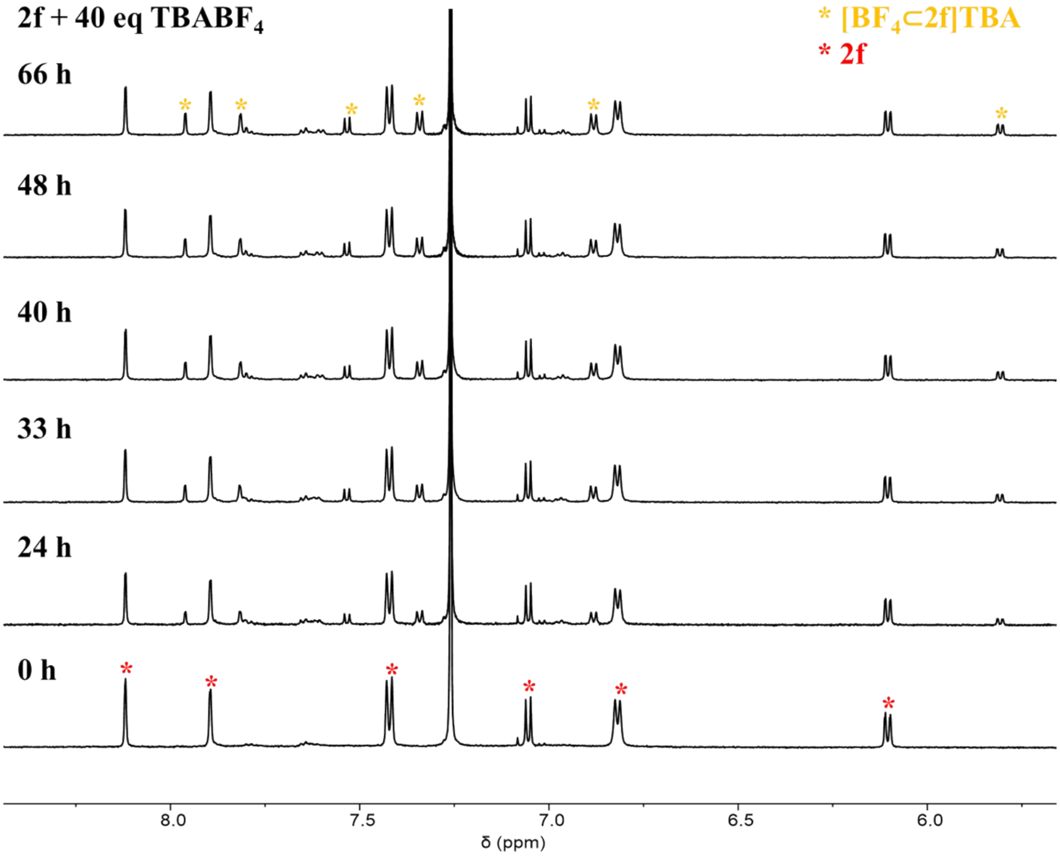

Figure S106. Partial ^{1}H NMR spectra (600 MHz, 298 K, $CDCl_3$) of a mixture of **2f** (0.15 mM) and $TBA^{+}\cdot BF_4^{-}$ (6 mM), which were recorded for a certain amount of time including 0 (bottom) and 66 h (top). The resonances corresponding to the free cage and cage-anion complex were labelled with red and yellow asterisks, respectively. The pseudo-first-order rate constant, k_{obs}, was calculated to be 6.73×10^{-3} h^{-1}, assuming that the concentration of free anion remained constant.

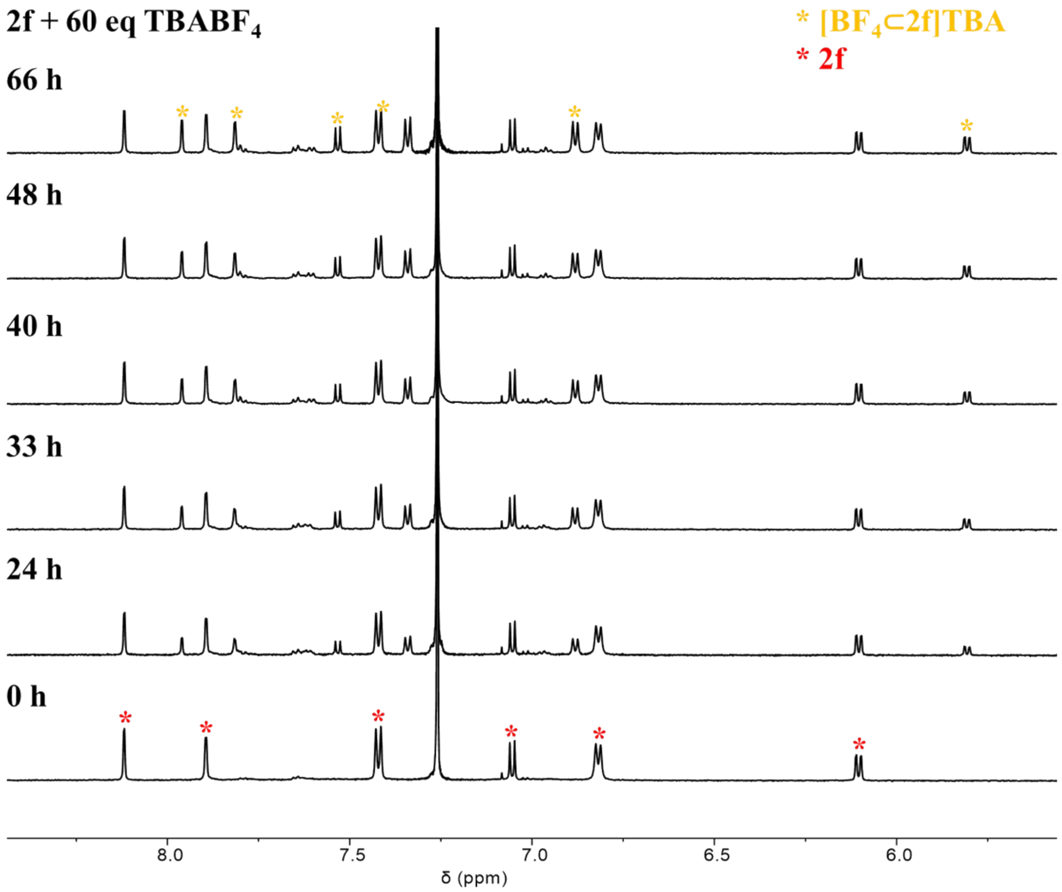

Figure S107. Partial ^{1}H NMR spectra (600 MHz, 298 K, $CDCl_3$) of a mixture of **2f** (0.15 mM) and $TBA^+ \cdot BF_4^-$ (9 mM), which were recorded for a certain amount of time including 0 (bottom) and 66 h (top). The resonances corresponding to the free cage and cage-anion complex were labelled with red and yellow asterisks, respectively. The pseudo-first-order rate constant, k_{obs}, was calculated to be 10.93 × 10^{-3} h^{-1}, assuming that the concentration of free anion remained constant.

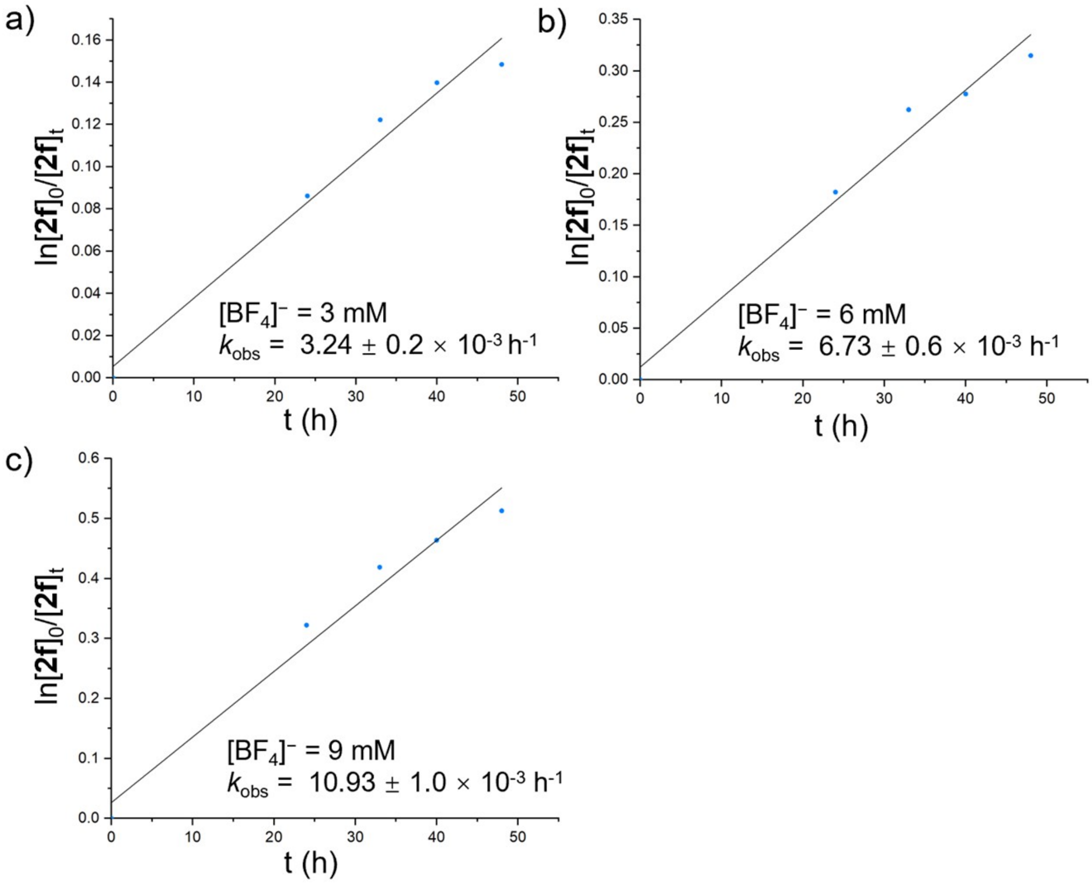

Figure S108. Plots of $\ln[\mathbf{2f}]_0/[\mathbf{2f}]_t$ versus time (t) when the anion added is a) 3, b) 6 and c) 9 mM, based on the ^{1}H NMR spectroscopic results in Figure 104-S106. $[\mathbf{2f}]_0$ and $[\mathbf{2f}]_t$ are the relative concentration of cage **2f** in the beginning and a special time after addition of the anion, respectively, which was determined by integrating the corresponding resonances corresponding to the cage and complex respectively. Linear fitting was performed in each case, producing three straight lines. The rates of host-guest complexation namely k_{obs} in each case is the slope of the linear, namely $\ln[\mathbf{2f}]_0/[\mathbf{2f}]_t = k_{obs}t$.

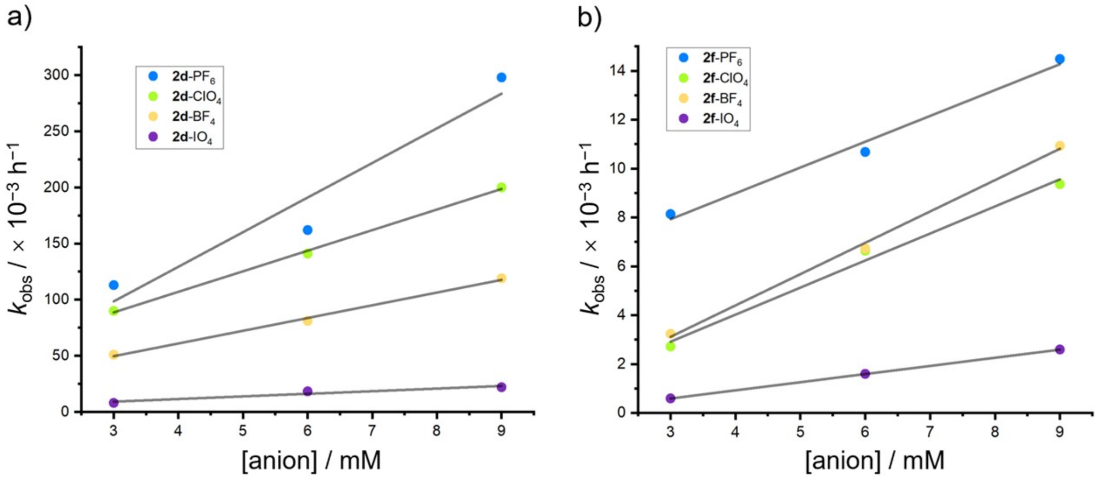

Figure S109. Plots of k_{obs} vs the concentration of anions for the formation of complexes namely a) anion⊂**2d** and b) anion⊂**2f**. Linear fitting was performed to yield the rate of association namely k_f, while the rates of dissociation (k_b) were determined from the equation $k_b = k_f/K_a$.

Table S1. Kinetic parameters for the complexation between cage **2d**, **2f** and four anions.

	**2d**				**2f**			
	PF_6^-	ClO_4^-	BF_4^-	IO_4^-	PF_6^-	ClO_4^-	BF_4^-	IO_4^-
K_a (10^3 M^{-1})	5.6	1.0	0.16	0.25	5.7	1.1	0.09	1.2
k_f (M^{-1} h^{-1})	30.8	18.3	11.3	2.3	1.06	1.11	1.28	0.33
k_b (10^{-3} h^{-1})	5.5	18.3	70.6	9.2	0.19	1.0	14.2	0.03

6. Computational Studies

Density functional theory (DFT) calculations

DFT calculations were performed using Gaussian 16[2] package. The geometries of all structures have been optimized at the wb97xd/def2-SVP[3-4] level of theory with the IEFPCM[5] solvation model (chloroform). Initial structures were obtained or modified based on the single crystal structures. Harmonic vibrational analyses have been performed to confirm each stationary point as an equilibrium structure (i.e., all real frequencies). Single-point energy calculations were carried out with the M06-2X-D3/6-311+G(2d,p)[6-10] level in the SMD[5] implicit solvent model (chloroform). Gibbs free energies were corrected with the quasi-rigid-rotor-harmonic-oscillator (quasi-RRHO)[11] method. Energy decomposition analysis (EDA) was conducted at the BHandHLYP-D3(BJ)/6-311+G(2d,p)[12] w.CB level using the sobEDA[13] method. Wave function analyses including IGMH[14] and ESP[15] were obtained using the Multiwfn 3.8(dev) program.[16] The corresponding plots and maps were rendered using the VMD software[17] with the plotting scripts included in the Multiwfn package.

Energy decomposition analysis (EDA)

We first of all employed EDA for the interactions between ClO_4^- and the tetrahedral cage using the sobEDAw method. This approach provides an efficient and universal protocol for quantifying contributions to interfragment interaction energies. Calculations were performed on the optimized complex geometries and the results are summarized in Table S2.

The EDA results reveal a total interaction energy trend that aligns well with the experimental results. For the halogen-substituted series (**2b**, **2c** and **2d**), a closer inspection of the energy components shows that while the exchange-repulsion (ΔE_{xrep}), orbital interaction (ΔE_{orb}), and dispersion (ΔE_{disp}) terms each exhibit monotonic variations across the series, their differences are modest compared to that of the electrostatic term and are largely countervailing. This analysis underpins the primacy of electrostatic contribution in governing the overall binding affinity. Similarly, a

significant drop of the electrostatic interaction accounts for the low affinity observed in **2e**, which features electron-donating phenyl substituents. While in the case of **2f**, the high anion affinity arises not only from reasonable electrostatic attractions but also from a significant reduction in Pauli repulsion.

Table S2. Physical components of the interaction energy (kcal mol^{-1}) for ClO_4^- complexes in the gas state calculated by sobEDAw method. ΔE_{els}, ΔE_x, ΔE_{rep}, ΔE_{orb} and ΔE_{disp} represent the electrostatic energy, exchange energy, Pauli repulsion energy, orbital interaction energy and dispersion energy, respectively. ΔE_x and ΔE_{rep} are merged into one term, as exchange-repulsion term, namely, $\Delta E_{xrep} = \Delta E_x + \Delta E_{rep}$. Total interaction energy $\Delta E_{int} = \Delta E_{els} + \Delta E_{xrep} + \Delta E_{orb} + \Delta E_{disp}$.

complexes	ΔE_{int}	ΔE_{els}	ΔE_{xrep}	ΔE_{orb}	ΔE_{disp}	ΔE_x	ΔE_{rep}
$ClO_4^-\subset$**2b**	−56.67	−37.59	46.63	−25.79	−39.92	−31.90	82.06
$ClO_4^-\subset$**2c**	−61.33	−42.29	47.79	−26.40	−40.42	−32.80	83.97
$ClO_4^-\subset$**2d**	−61.62	−43.01	49.39	−26.97	−41.02	−34.13	86.95
$ClO_4^-\subset$**2e**	−51.78	−31.80	45.45	−27.01	−38.42	−31.14	80.28
$ClO_4^-\subset$**2f**	−65.25	−39.83	39.58	−26.48	−38.51	−25.81	68.55

Independent gradient model based on Hirshfeld partition of molecular density (IGMH) Analysis

In order to reveal the noncovalent interactions in the host-guest complex, we employed a real-space function, named the interaction region indicator independent gradient model based on the Hirshfeld partition of molecular density (IGMH) method to investigate noncovalent interactions. The complex was partitioned into two fragments: the guest ClO_4^- anion as one fragment and the tetrahedral cage as the other. Thereby, the resulting isosurfaces reveal exclusively host-guest interactions. It can be observed that the IGMH analysis clearly visualizes all types of interactions between the two fragments, including hydrogen-bonding (H-bond) interactions and anion–π

interactions. In the IGMH analyses, the isosurfaces correspond to δginter = 0.003 a.u., and the generated cube files represent high-quality data.

Quantitative assessment of anion-π and hydrogen bond interactions

It is well-established that the electrostatic component of the anion-π interaction is correlated to the quadrupole moment of the electron deficient aromatic ring. We therefore computed the average magnitudes of traceless quadrupole moments for the triazine rings in each cage (Table S3). The result shows that all the triazine rings exhibit positive traceless quadrupole moments, consistent with their electron-deficient nature. The average values, however, showed neither significant difference nor a discernible trend across the halide series. Furthermore, higher value was observed for **2e** than those for **2c** and **2d** which is inconsistent with the experimental results, suggesting that differential anion-π interactions are unlikely to be the key determinant of the observed trend in binding affinity.

Table S3. Calculated average magnitudes of quadrupole moments (Debye·Å) for the four triazine rings in each cage.

	**2b**	**2c**	**2d**	**2e**	**2f**
$Q_{\text{traceless}}$	1.77	1.46	1.49	2.72	3.82
$\|Q\|$	2.55	1.96	2.17	4.61	6.00

The strength of CH–O hydrogen bonds can be estimated[18] as the half value of the potential energy density $V(\mathbf{r})$ at the bond critical point (BCP) for the hydrogen bonds. Data derived from the same level of theory with the EDA analyses reveal (Table S4) a minor progression of the sum of hydrogen bond interactions between the cage and ClO_4^-, that the hydrogen bond interactions within complex $ClO_4^-\subset$**2d** is larger than that of the $ClO_4^-\subset$**2b** by merely 1.0 kcal/mol. This difference accounts for only 20% of the distinction of the total interaction energy between $ClO_4^-\subset$**2d** and $ClO_4^-\subset$**2b**. As a

result, the observed differential binding trend should be attributed to a more dominant electrostatic contribution.

Table S4. Sum of hydrogen bond interaction energies (kcal mol^{-1}) within the ClO_4^- complexes estimated as the half value of *V*(r) at the BCP for the hydrogen bonds.

	ClO_4^-⊂**2b**	ClO_4^-⊂**2c**	ClO_4^-⊂**2d**	ClO_4^-⊂**2e**	ClO_4^-⊂**2f**
E_{HB}	−17.63	−18.06	−18.68	−12.78	−16.77

Electrostatic potential (ESP) Analysis

To validate that the anion and the cage tend to interact through electrostatic complementarity, we analyzed the electrostatic potential (ESP) distribution both over the entire vdW surface of the cages and across a defined cross-sectional plane within the cage. This plane was selected parallel to a triazine plane of the tetrahedral cage (Figure S109 f) and positioned 3 Å above it. We selected (150, 150) grid points to generate the .cube file. The plane selected for each of the five molecular cages was defined by three points, with their coordinates provided in the table below:

Table S5. The coordinates (Å) of the three points selected to define the plane within the cage.

Name	**2b**	**2c**	**2d**	**2e**	**2f**
Point1	(−1.9609716, −0.2465801, 0.1461176)	(0.0199476, −1.2945553, −1.1136127)	(−1.5987199, −0.9065082, −0.4487543)	(−1.5639241, −0.9827590, −0.0247870)	(−1.2678594, −0.4435849, 1.0656113)
Point2	(0.2700272, −0. 6316981, −0.6979060)	(−1.6123706, −0.3197284, 0.3774232)	(0.6528679, −1.1461509, −1.2918809)	(−0.6800759, 0.5713110, −1.6501258)	(−0.1979343, 1.6839671, 0.6569803)
Point3	(−0.2962742, 1.4985254, 0.2921583)	(0.6083530, −0.8279752, 1.1829346)	(−0.2874632, 1.0557544, −0.9667587)	(−0.9799766, 1.2642624, 0.6450466)	(1.1430890, −0.2846621, 1.0625282)

The results demonstrate that, among the five cages studied, both the global and local ESP analyses consistently reveal the same trend: the **2b** and **2e** exhibit the least electron-deficient character, followed by the **2c**, while the **2d** and **2f** show the most pronounced electron-deficient properties (Figure S109).

Restrained electrostatic potential charge (RESP)[19] analysis

The above ESP analysis surprisingly showed that both the electron-withdrawing fluorine groups and the electron-donating phenyl groups result in the least electron-deficient cavity. In order to clarify this counterintuitive effect, we derived RESP for each atom. The average values of the net atomic charge for key fragments are summarized in Table S6.

Table S6. The average values of the net RESP charge (a.u.) for key fragments in the empty cages.

Fragments	**2b**	**2c**	**2d**	**2e**	**2f**
Triazines	0.33	0.12	0.07	0.18	0.20
HB donor hydrogens	0.13	0.16	0.19	0.16	0.16
Halogen atoms	−0.22	−0.083	−0.054	–	–

RESP charge analysis reveals a progressive increase in the net positive charge on the triazine fragments across the series **2d** to **2b**, consistent with the enhanced inductive electron-withdrawing effect of the increasingly electronegative halogen substituents. Correspondingly, the halogen atoms bear substantial negative charges, with average values decreasing monotonically: **2d** (−0.054 a.u.) > **2c** (−0.083 a.u.) >> **2b** (−0.22 a.u.). This charge localization is visually corroborated by the ESP maps (Figure S110), which show pronounced electron-rich regions over the halogen surfaces.

The significant negative charge localized on the halogen atoms prompted a quantitative assessment of their electrostatic influence. This was achieved by computing the interaction energy between the key fragments and an imaginary unit (−1) charge positioned at the cage center. The result are summarized in Table S7.

Table S7. The electrostatic potential energies (kcal mol^{-1}) exerted by key fragments towards an imaginary unit (−1) charge positioned at the cage center.

Fragments	**2b**	**2c**	**2d**	**2e**	**2f**
Triazines	−110.85	−40.97	−23.75	−56.44	−64.63
Halogen atoms	135.15	47.02	29.67	–	1.59*
Triazines and halogens	24.30	6.05	5.92	–	–
All atoms	−9.78	−10.96	−21.14	−6.45	−25.48

* Electrostatic potential energies exerted by twelve CF_3 groups.

The four triazine units induce attractive electrostatic potentials of −110.8 kcal/mol, −41.0 kcal/mol, and −23.8 kcal/mol for **2b**, **2c**, and **2d**, respectively. These attractions, however, are effectively overshadowed by the repulsive potentials arising from the halogen atoms, calculated as 135.2 kcal/mol for **2b**, 47.0 kcal/mol for **2c**, and 29.7 kcal/mol for **2d**, respectively. Consequently, the strongest net electrostatic repulsion is observed for **2b** (24.3 kcal/mol) which aligns with the experimental anion affinity series and rationalize the existence of repulsive field effect from the halogen atoms.

Electric Field Gradient Diagram

The external electric field (EEF) is conventionally defined as the negative of the first-order derivative vector with respect to the coordinate, which is the gradient vector of the electrostatic potential (ESP) of Figure S109. Hence, by generating a gradient line or vector field representation of the ESP, the characteristics and behavior of the electric field can be illustrated. We selected (100, 100) grid points. In all the electric field gradient distribution diagrams, we set the upper limit of the absolute value for scaling arrows to 0.05.

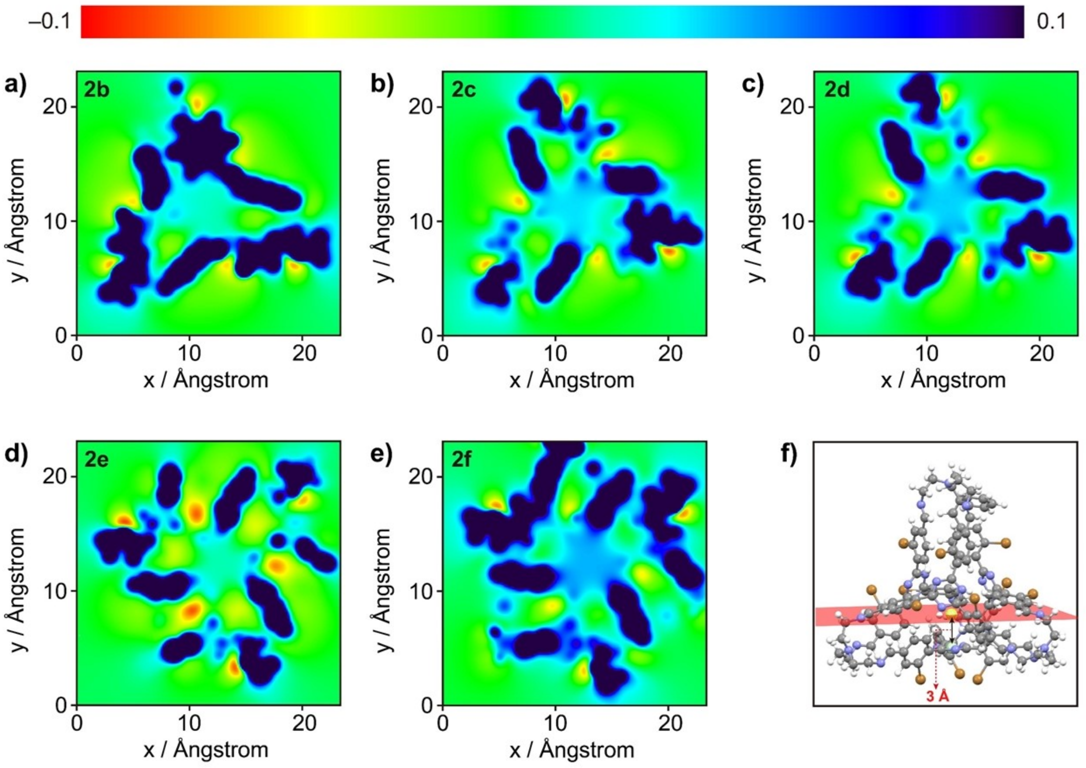

Figure S110. The 2D electrostatic potential map of **2b** (a), **2c** (b), **2d** (c), **2e** (d), **2f** (e). The selected plane was constructed based on the coordinates of the three points shown in Table S2.

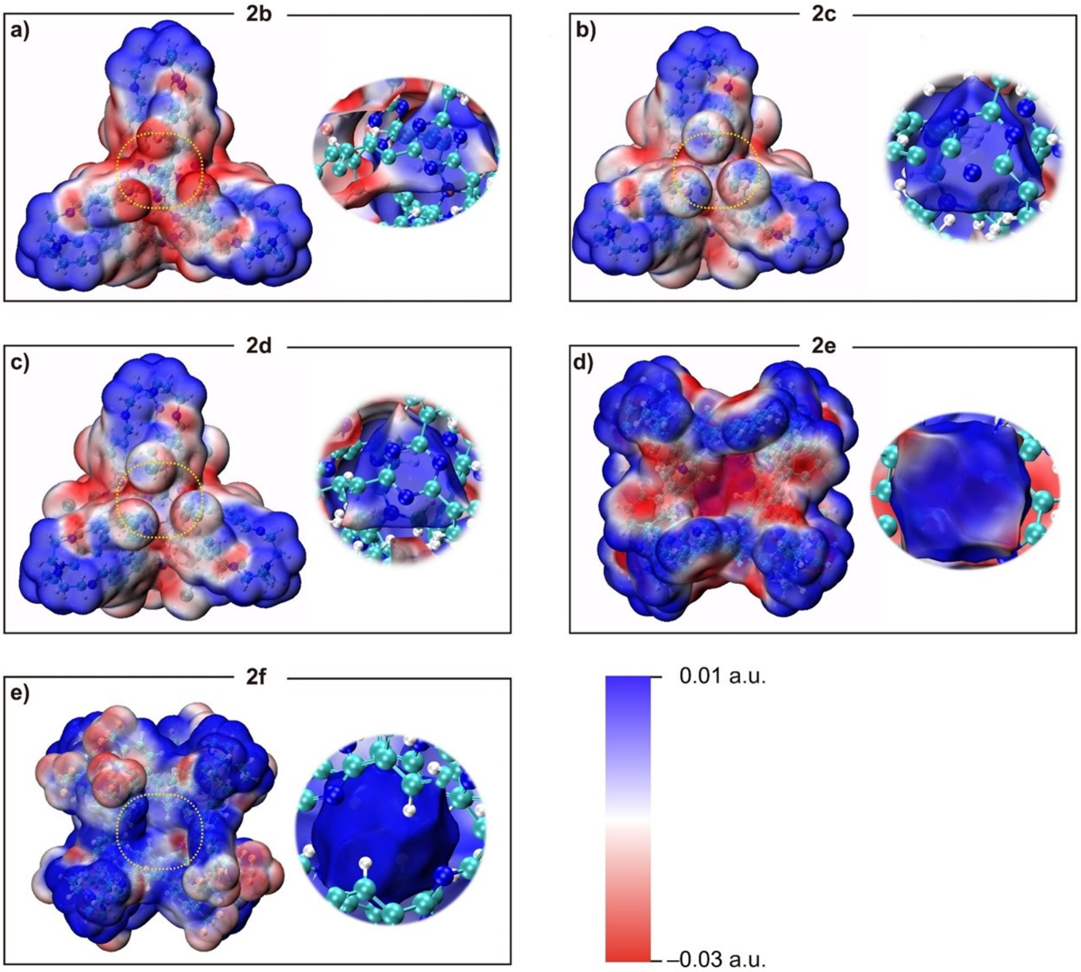

Figure S111. Surface electrostatic potential distribution of **2b** (a), **2c** (b), **2d** (c), **2e** (d), **2f** (e).

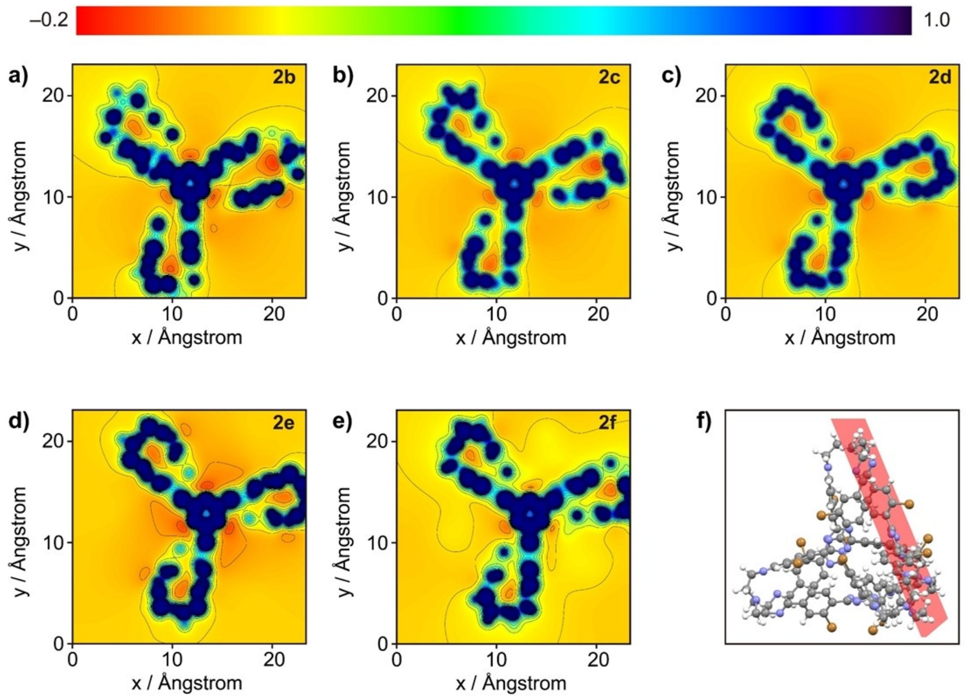

Figure S112. The 2D electrostatic potential map of **2b** (a), **2c** (b), **2d** (c), **2e** (d), **2f** (e). The selected plane was constructed based on the coordinates of the three nitrogen atoms located on the triazine ring, as shown in (f).

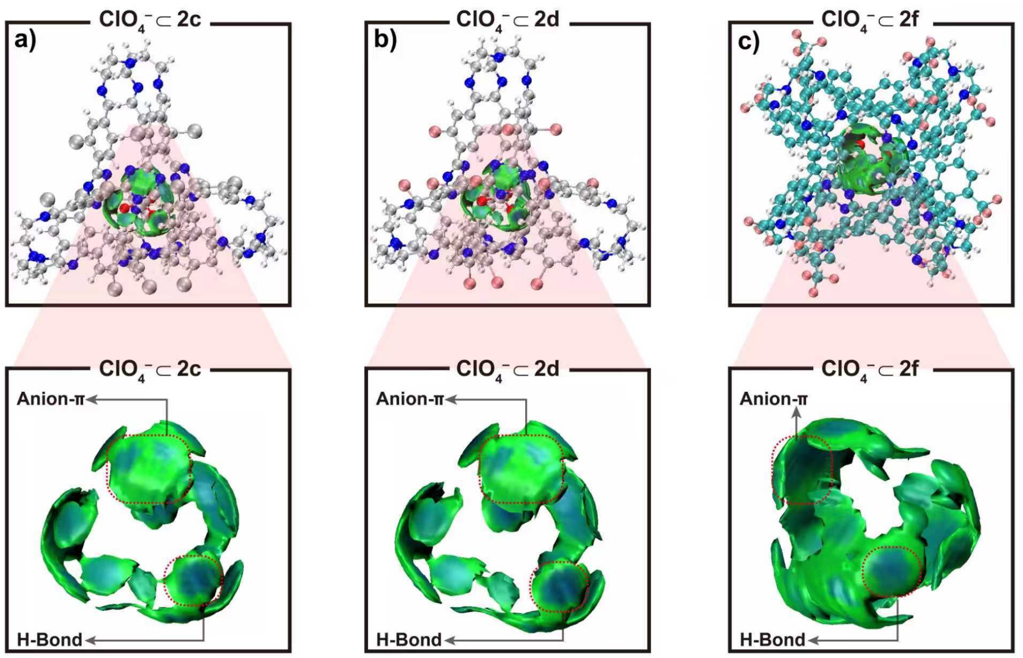

Figure S113. The reduced density gradient isosurface map of the **ClO_4^-⊂2c** (a), **ClO_4^-⊂2c** (b), **ClO_4^-⊂2f** (c), with the isosurfaces corresponding to $\delta g^{inter} = 0.003$ a.u. For clarity, only the noncovalent interaction between Cage and anion was displayed.

7. X-Ray Crystallography

Single crystal X-ray diffraction data were performed on Bruker D8 Venture metaljet diffractometer (Bruker, Ettlingen, Germany) with GaKα (λ = 1.34139 Å) radiation. Using Olex2[20], the crystal structure was solved using intrinsic phasing and refined with full-matrix least-squares methods with anisotropic thermal parameters for all nonhydrogen atoms on F^2 using SHELXL[21] package. Hydrogen atoms were found from the difference Fourier map but placed in the calculated positions and refined isotropically.

Solid-state structure of complex BF_4^-⊂2c·TBA$^+$

Methods

Single crystals of BF_4^-⊂**2c**·TBA$^+$, suitable for X-ray crystallography, were grown by slow solvent diffusion of isopropyl ether into a solution of **2c** with 10 equiv. of TBA$^+$·BF_4^- in chloroform after three days. Data were collected at 170 K on a Bruker D8 Venture Diffractometer equipped with a GaKα IµS source and MX optic.

Crystal parameters of BF_4^-⊂2c·TBA$^+$

[$C_{141.9}H_{137.9}BCl_{29.7}F_4N_{29}$], 0.17×0.17×0.05 mm^3, M = 3389.18, Triclinic, space group P-1, a = 17.5969(6) Å, b = 21.9736(7) Å, c = 22.7357(9) Å, α = 102.3817(19)°, β = 93.1489(18)°, γ = 94.7641(16)°. V = 8533.1(5) Å^3, Z = 2, T = 170 K, ρ_{calc} = 1.319 mg/m^3, μ(GaKα) = 3.249 mm^{-1}. 136792 reflections collected, 32470 independent reflections, R(int) = 0.0856. Final R_1 (I > 2σ(I)) = 0.0760 and wR_2 = 0.2067. The structure was solved by intrinsic phasing and different Fourier syntheses. Using Olex2, the structure was solved with the ShelXT structure solution program using Intrinsic Phasing and refined with the ShelXL refinement package using Least Squares minimization. The SQUEEZE procedure was done (see details in the cif file). CCDC number: 2483576.

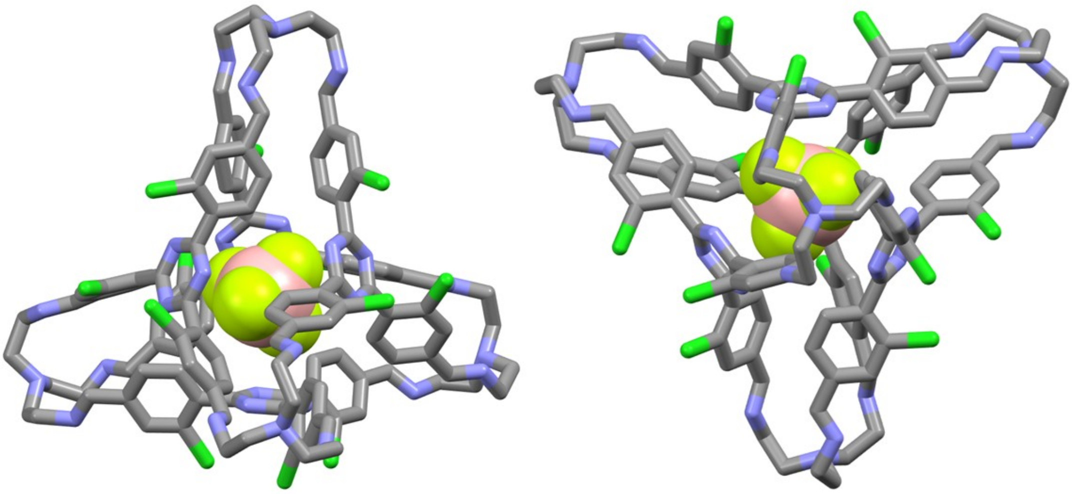

Figure S114. Different views of the solid-state structure of $BF_4^-\subset$**2c**$\cdot$TBA$^+$. Color code: carbon, grey; boron, pink; nitrogen, blue; fluorine, cyan; chlorine, green. Disordered solvent molecules, other counterions, and hydrogen atoms are omitted for clarity.

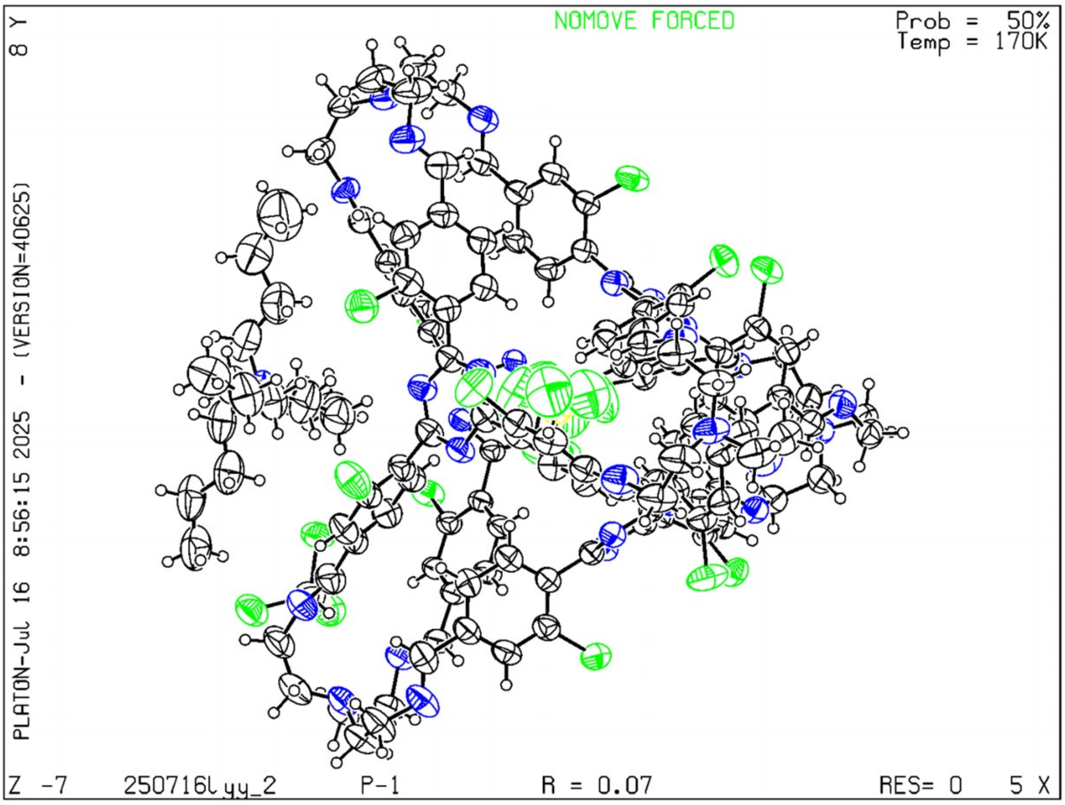

Figure S115. Thermal ellipsoid plot for $BF_4^-\subset$**2c**$\cdot$TBA$^+$ crystal. Color code: carbon, grey; boron, pink; nitrogen, blue; fluorine, cyan; chlorine, green. And displacement ellipsoids are drawn at the 50% probability level.

Solid-state structure of complex ClO_4^-⊂2c·TBA$^+$

Methods

Single crystals of ClO_4^-⊂**2c**·TBA$^+$, suitable for X-ray crystallography, were grown by slow solvent diffusion of isopropyl ether into a solution of **2c** with 10 equiv. of TBA$^+$·ClO_4^- in chloroform after three days. Data were collected at 170 K on a Bruker D8 Venture Diffractometer equipped with a GaKα IμS source and MX optic.

Crystal parameters of ClO_4^-⊂2c·TBA$^+$

[$C_{141.29}H_{137.29}Cl_{28.86}N_{29}O_4$], 0.17×0.17×0.05 mm^3, M = 3328.73, Triclinic, space group P-1, a = 17.6131(10) Å, b = 22.0166(14) Å, c = 22.7591(14) Å, α = 102.389(3)°, β = 93.067(3)°, γ = 94.661(2)°. V = 8568.7(9) Å^3, Z = 2, T = 170 K, ρ_{calc} = 1.290 mg/m^3, μ(GaKα) = 3.140 mm^{-1}. 133242 reflections collected, 25510 independent reflections, R(int) = 0.1274. Final R_1 (I > 2σ(I)) = 0.0743 and wR_2 = 0.1904. The structure was solved by intrinsic phasing and different Fourier syntheses. Using Olex2, the structure was solved with the ShelXT structure solution program using Intrinsic Phasing and refined with the ShelXL refinement package using Least Squares minimization. The SQUEEZE procedure was done (see details in the cif file). CCDC number: 2483577.

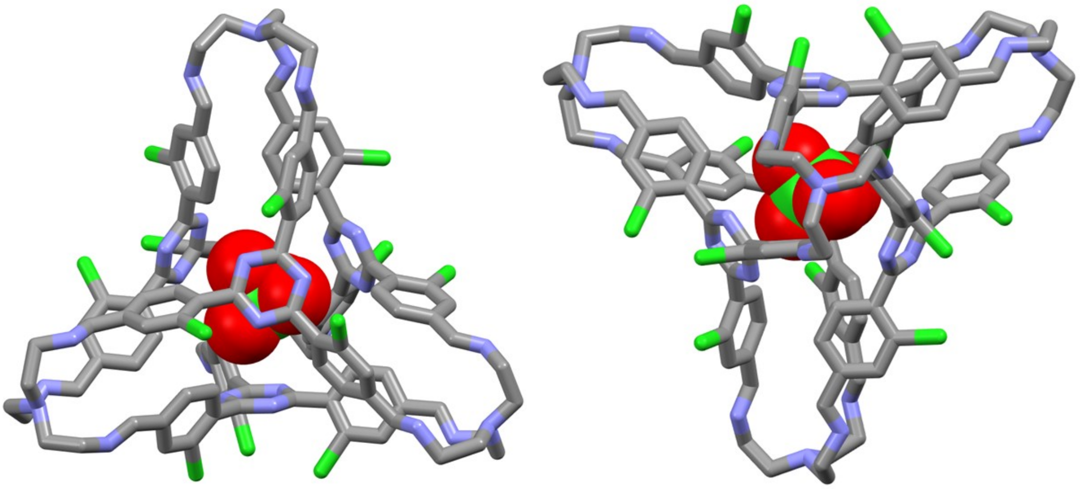

Figure S116. Different views of the solid-state structure of ClO_4^-⊂**2c**·TBA$^+$. Color code: carbon, grey; nitrogen, blue; oxygen, red; chlorine, green. Disordered solvent molecules, other counterions, and hydrogen atoms are omitted for clarity.

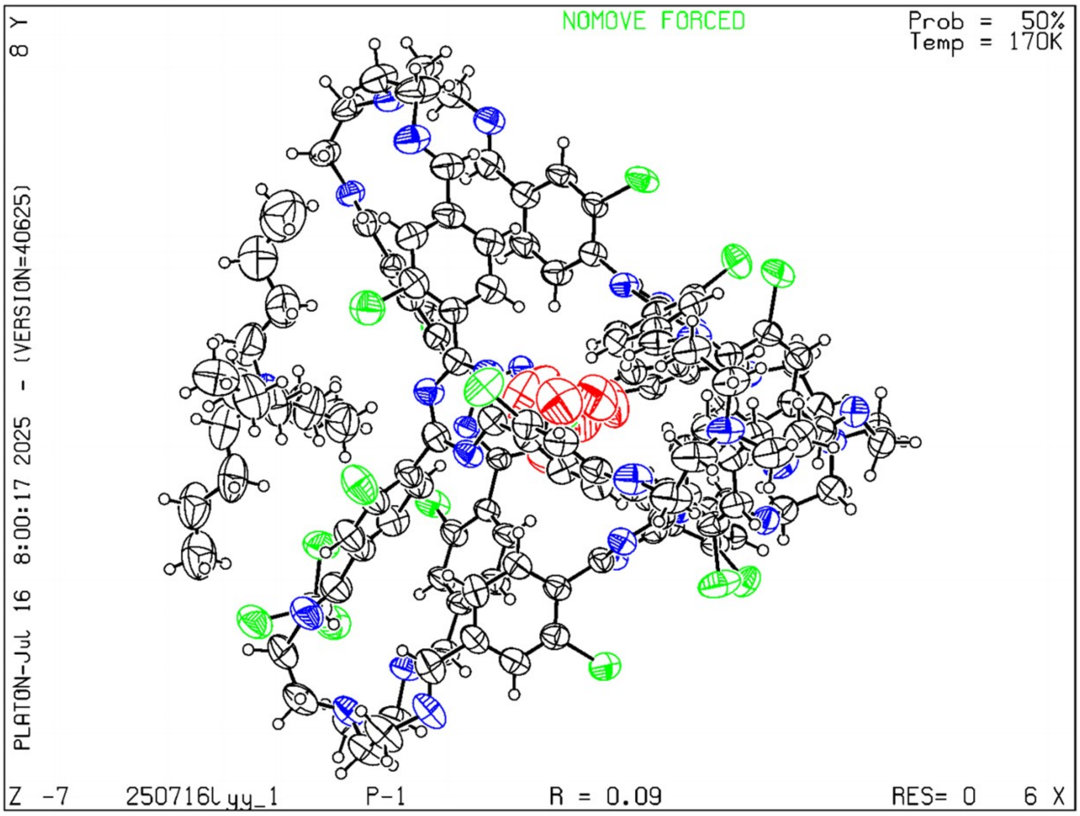

Figure S117. Thermal ellipsoid plot for ClO_4^{-}⊂**2c**·TBA^{+} crystal. Color code: carbon, grey; nitrogen, blue; oxygen, red; chlorine, green. And displacement ellipsoids are drawn at the 50% probability level.

8. References

[S1] Li, S.-H.; Huang, H.-P.; Yu, S.-Y.; Li, X.-P., Design and Synthesis of Polypyrazolyl Compounds as a New Type of Versatile Building Blocks†. *Chin. J. Chem.* **2006,** *24*, 1225-1229.

[S2] Frisch, M. J.; Trucks, G. W.; Schlegel, H. B.; Scuseria, G. E.; Robb, M. A.; Cheeseman, J. R.; Scalmani, G.; Barone, V.; Petersson, G. A.; Nakatsuji, H.; Li, X.; Caricato, M.; Marenich, A. V.; Bloino, J.; Janesko, B. G.; Gomperts, R.; Mennucci, B.; Hratchian, H. P.; Ortiz, J. V.; Izmaylov, A. F.; Sonnenberg, J. L.; Williams; Ding, F.; Lipparini, F.; Egidi, F.; Goings, J.; Peng, B.; Petrone, A.; Henderson, T.; Ranasinghe, D.; Zakrzewski, V. G.; Gao, J.; Rega, N.; Zheng, G.; Liang, W.; Hada, M.; Ehara, M.; Toyota, K.; Fukuda, R.; Hasegawa, J.; Ishida, M.; Nakajima, T.; Honda, Y.; Kitao, O.; Nakai, H.; Vreven, T.; Throssell, K.; Montgomery Jr., J. A.; Peralta, J. E.; Ogliaro, F.; Bearpark, M. J.; Heyd, J. J.; Brothers, E. N.; Kudin, K. N.; Staroverov, V. N.; Keith, T. A.; Kobayashi, R.; Normand, J.; Raghavachari, K.; Rendell, A. P.; Burant, J. C.; Iyengar, S. S.; Tomasi, J.; Cossi, M.; Millam, J. M.; Klene, M.; Adamo, C.; Cammi, R.; Ochterski, J. W.; Martin, R. L.; Morokuma, K.; Farkas, O.; Foresman, J. B.; Fox, D. J. *Gaussian 16 Rev. C.01*, Wallingford, CT, 2016.

[S3] Chai, J.-D.; Head-Gordon, M., Long-range Corrected Hybrid Density Functionals with Damped Atom–Atom Dispersion Corrections. *Phys. Chem. Chem. Phys.* **2008,** *10*, 6615-6620.

[S4] Weigend, F.; Ahlrichs, R., Balanced Basis Sets of Split Valence, Triple Zeta Valence and Quadruple Zeta Valence Quality for H to Rn: Design and Assessment of Accuracy. *Phys. Chem. Chem. Phys.* **2005,** *7*, 3297-3305.

[S5] Marenich, A. V.; Cramer, C. J.; Truhlar, D. G., Universal Solvation Model Based on Solute Electron Density and on a Continuum Model of the Solvent Defined by the Bulk Dielectric Constant and Atomic Surface Tensions. *J. Phys. Chem. B* **2009,** *113*, 6378-6396.

[S6] Grimme, S.; Ehrlich, S.; Goerigk, L., Effect of the Damping Function in Dispersion Corrected Density Functional Theory. *J. Comput. Chem.* **2011,** *32*, 1456-1465.

[S7] Krishnan, R.; Binkley, J. S.; Seeger, R.; Pople, J. A., Self-Consistent Molecular Orbital Methods. XX. A Basis Set for Correlated Wave Functions. *J. Chem. Phys.* **1980,** *72*, 650-654.

[S8] Clark, T.; Chandrasekhar, J.; Spitznagel, G. W.; Schleyer, P. V. R., Efficient Diffuse Function-Augmented Basis Sets for Anion Calculations. III. The 3-21+G Basis Set for First-Row Elements, Li–F. *J. Comput. Chem.* **1983,** *4*, 294-301.

[S9] Zhao, Y.; Truhlar, D. G., The M06 Suite of Density Functionals for Main Group Thermochemistry, Thermochemical Kinetics, Noncovalent Interactions, Excited States, and Transition Elements: Two New Functionals and Systematic Testing of Four M06-Class Functionals and 12 Other Functionals. *Theor. Chem. Acc.* **2008,** *120*, 215-241.

[S10] Frisch, M. J.; Pople, J. A.; Binkley, J. S., Self-Consistent Molecular Orbital Methods 25. Supplementary Functions for Gaussian Basis Sets. *J. Chem. Phys.* **1984,** *80*, 3265-3269.

[S11] Grimme, S., Supramolecular Binding Thermodynamics by Dispersion-Corrected Density Functional Theory. *Chem. - Eur. J.* **2012,** *18*, 9955-9964.

[S12] Lee, C.; Yang, W.; Parr, R. G., Development of the Colle-Salvetti Correlation-Energy Formula into a Functional of the Electron Density. *Phys. Rev. B* **1988,** *37*, 785-789.

[S13] Lu, T.; Chen, Q., Simple, Efficient, and Universal Energy Decomposition Analysis Method Based on Dispersion-Corrected Density Functional Theory. *J. Phys. Chem. A* **2023,** *127*, 7023-7035.

[S14] Lu, T.; Chen, Q., Independent Gradient Model Based on Hirshfeld Partition: A New Method for

Visual Study of Interactions in Chemical Systems. *J. Comput. Chem.* **2022,** *43*, 539-555.

[S15] Zhang, J.; Lu, T., Efficient Evaluation of Electrostatic Potential with Computerized Optimized Code. *Phys. Chem. Chem. Phys.* **2021,** *23*, 20323-20328.

[S16] Lu, T.; Chen, F., Multiwfn: A Multifunctional Wavefunction Analyzer. *J. Comput. Chem.* **2012,** *33*, 580-592.

[S17] Humphrey, W.; Dalke, A.; Schulten, K., VMD: Visual Molecular Dynamics. *J. Mol. Graphics* **1996,** *14*, 33-38.

[S18] Espinosa, E.; Molins, E.; Lecomte, C., Hydrogen Bond Strengths Revealed by Topological Analyses of Experimentally Observed Electron Densities. *Chem. Phys. Lett.* **1998,** *285*, 170-73.

[S19] Bayly, C. I.; Cieplak, P.; Cornell, W.; Kollman, P. A., A Well-behaved Electrostatic Potential Based Method Using Charge Restraints for Deriving Atomic Charges: The RESP Model. *J. Phys. Chem.* **1993,** *97*, 10269-10280.

[S20] Dolomanov, O.V., Bourhis, L.J., Gildea, R.J, Howard, J.A.K. & Puschmann, H, *OLEX2*: a Complete Structure Solution, Refinement and Analysis Program. *J. Appl. Cryst.* **2009,** 42, 339-341.

[S21] Sheldrick, G.M., Crystal Structure Refinement with *SHELXL*. Acta Cryst. **2015,** A71, 3-8.
